# Efficient Palladium‐Catalyzed Aerobic Oxidative Carbocyclization to Seven‐Membered Heterocycles

**DOI:** 10.1002/chem.202004265

**Published:** 2020-10-22

**Authors:** Jie Liu, Jan‐E. Bäckvall

**Affiliations:** ^1^ Department of Organic Chemistry Arrhenius Laboratory Stockholm University 10691 Stockholm Sweden; ^2^ Department of Natural Sciences Mid Sweden University Holmgatan 10 85170 Sundsvall Sweden; ^3^ College of Chemistry and Chemical Engineering Hunan University 410082 Changsha P. R. China

**Keywords:** aerobic oxidation, carbocyclization, heterocycles, palladium, regioseletivity

## Abstract

The use of molecular oxygen in palladium‐catalyzed oxidation reactions is highly widespread in organic chemistry. However, the direct reoxidation of palladium by O_2_ is often kinetically unfavored, thus leading the deactivation of the palladium catalyst during the catalytic cycle. In the present work, we report a highly selective palladium‐catalyzed carbocyclization of bisallenes to seven‐membered heterocycles under atmospheric pressure of O_2_. The use of a homogenous hybrid catalyst (Co(salophen)‐HQ, HQ=hydroquinone) significantly promotes efficient electron transfer between the palladium catalyst and O_2_ through a low‐energy pathway. This aerobic oxidative transformation shows broad substrate scope and functional group compatibility and allowed the preparation of *O*‐containing seven‐membered rings in good yields in most cases.

## Graphical abstract

**Heterocycles**: We report a Pd^II^‐catalyzed oxidative carbocyclization of the rationally designed bisallenes using molecular oxygen. By applying a bifunctional catalyst (Co(salophen)‐HQ) as an efficient electron‐transfer mediator, bisallenes are effectively converted to the corresponding seven‐membered carbocycles in good yields (see scheme).

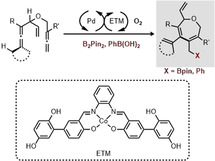

Over the past decades, palladium‐catalyzed oxidations have emerged as powerful and valuable tools in modern organic synthesis.^[1]^ Various protocols have been developed for the assembly of carbon‐carbon and carbon‐heteroatom bonds that provide useful applications in biology, medicine, and materials science.^[2]^ However, in most cases the use of stoichiometric oxidants such as Cu^II^, Ag^I^, and peroxides often leads to poor selectivity, low atom economy, and considerable amounts of undesired waste. In the perspective of synthetic organic chemistry, molecular oxygen is an inexpensive, abundant, and highly atom‐efficient oxidant, which does not generate any toxic byproducts, thus fulfilling the requirements of “green chemistry”.^[3]^ Despite significant advances in palladium‐catalyzed aerobic oxidations, a severe problem is that fast aggregation of palladium black from the active palladium species (Pd‐H or Pd^0^) slows down and finally stops the homogenous reaction.^[4]^ Extensive endeavors for solving this problem have focused on the use of air‐stable ligands to restrain Pd^0^ precipitation during the catalytic cycle (Scheme 1 a).^[5]^ Moreover, Jiang and co‐workers recently developed an elegant protocol that utilizes an efficient metal‐organic framework (MOF) for the stabilization of the palladium catalyst in aerobic functionalizations (Scheme 1 b).^[6]^ Although a plethora of strategies to circumvent this oxidation problem have been reported, the development of highly active catalytic systems that enable mild Pd‐catalyzed aerobic oxidations continues to be an important topic in this area.

**Scheme 1:**
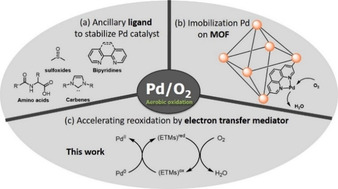
Strategies to improve the efficiency of palladium‐catalyzed aerobic oxidative reactions.

Our research group has a long‐standing interest in palladium‐catalyzed oxidations where molecular oxygen is used as a green oxidant.^[7]^ However, as stated above, the direct reoxidation of palladium by molecular oxygen is often kinetically unfavored.^[4]^ To solve this problem, we and others have employed coupled catalytic systems with electron transfer mediators (metal‐macrocycle and quinone) to facilitate the relay of electrons between the palladium catalyst and O_2_ (Scheme 1 c).^[8]^ These coupled catalytic systems have been demonstrated to be highly efficient in Wacker oxidations[^8b^, ^9^] alcohol oxidations,^[10]^ oxidative olefin functionalizations,^[11]^ and oxidative C−H activations^[12]^ where molecular oxygen is the oxidant. In 1993, a more efficient hybrid catalyst, involving a cobalt‐porphyrin with pendant hydroquinone groups in one molecule, was reported by our group as well.^[13]^ Later on, an improved hybrid Schiff base‐hydroquinone [Co(salophen)‐HQ] as a redox relay catalyst for aerobic oxidation was reported.^[14]^ The latter bifunctional catalyst led to a faster reaction rate compared to the system with the quinone and metal‐macrocycle as separate molecules in Pd‐catalyzed aerobic carbocyclization of enallenynes and dienallenes.[^8h^, ^15^]

During the past decade, our research group has been involved in the development of palladium‐catalyzed oxidative carbocyclizations of allenes bearing an additional unsaturated moiety, such as an olefin, alkyne, or allene.^[16]^ Interestingly, we found that an assisting group (AG),^[17]^ such as an olefin, alkyne or hydroxyl group, is required to make 6‐membered rings[^8e^, ^8h^, ^15^, ^18^] from enallenes and allenynes and to make 7‐membered rings from bisallenes due to the initial allenic C−H cleavage by Pd^II^ (Scheme 2 a). Afterward, cascade carbocyclization and transmetallation with a nucleophile can occur and give the cyclic product. However, without an activating group, no reaction takes place with the distal π‐bond to give the larger rings. This interesting neighboring group effect allows for palladium‐catalyzed carbocyclization in a highly selective manner. We recently reported an efficient approach for the synthesis of seven‐membered rings via olefin‐assisted palladium‐catalyzed carbonylative carbocyclization of bisallenes.^[19]^ However, O‐containing seven‐membered rings were not explored in the previous study. Also, there is no example on the use of oxidative carbocyclization of bisallenes for the synthesis of useful organoboron molecules.^[20]^ Herein we report a general and efficient catalytic system for the synthesis of O‐containing seven‐membered heterocycles via cascade borylative carbocyclizations (Scheme 2 b). This catalytic system involves the use of a Pd^II^ catalyst with a hybrid ETM catalyst for the biomimetic aerobic oxidations.

**Scheme 2:**
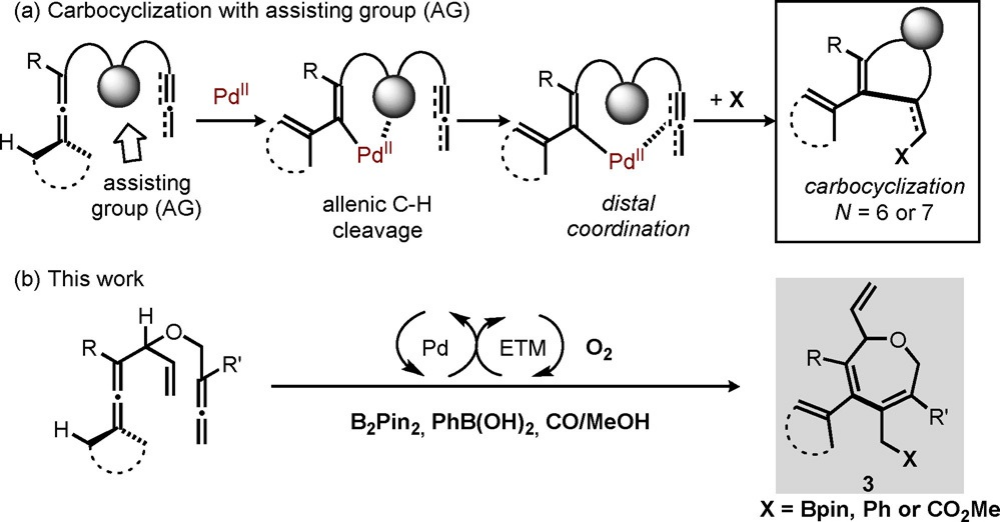
(a) Palladium‐catalyzed oxidative carbocyclization with an assisting group; (b) Current work.

Initially, a class of bifunctional Schiff base‐hydroquinone catalysts were prepared from the ligand precursor salicylaldehyde‐hydroquinone, which was covalently synthesized according to our previous report.^[14]^ As shown in Scheme 3, the synthetic route to these oxidation catalysts commences with condensation of two equivalents of salicylaldehyde‐hydroquinone with various diamines such as *o*‐phenylenediamine, 4‐chloro and 4‐methoxyl‐*o*‐phenylenediamine, ethylenediamine and *N‐(*3‐aminopropyl)‐*N*‐methylpropane‐1,3‐diamine. The resulting salen‐hydroquinone ligands could be directly used for the next step without further purification. Subsequently, coordination of these ligands with *3d* metal salts (Co, Fe, Ni, and Cu) afforded the corresponding hybrid catalysts (**Cat**. **1**–**9**) in general yields of 78–95 %.

**Scheme 3:**
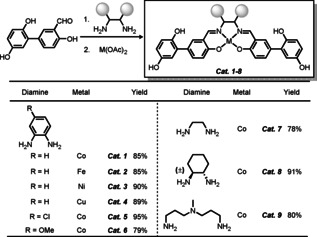
Synthesis of Schiff base‐hydroquinone hybrid catalysts.

At the outset of our investigations, the palladium‐catalyzed aerobic oxidation of a readily accessible bisallene **1 a** with B_2_pin_2_ **2 a** was chosen as the benchmark reaction (Table 1). In the absence of electron transfer mediators (ETMs), we did not observe any formation of the desired borylation product under aerobic conditions and the starting material **1 a** was recovered in 93 % yield (entry 1). When catalytic amounts of *p*‐benzoquinone (BQ) was added, the seven‐membered carbocycle **3 a** was selectively formed in low yield (15 %, entry 2). Notably, the use of cobalt‐based salophen complex with *p*‐benzoquinone (BQ) or hydroquinone (HQ) as separate ETMs led to increased but moderate yields of **3 a** (48 and 35 % yields, entries 3 and 4). To our delight, the bifunctional cobalt catalyst (Co(salophen)‐HQ), combining a metal‐macrocycle and a quinone moieties, was found to be a highly active catalyst and afforded the desired product **3 a** in 78 % yield (entry 5). Importantly, this aerobic catalytic system appeared to be equally effective in comparison to the application of stoichiometric amounts of BQ as oxidant (78 %, entry 6), therefore highlighting the advancement of this efficient and sustainable method.^[21]^

**Table 1:** Evaluation of different electron transfer mediators (ETMs) for borylative carbocyclization of bisallene **1 a**.^[a]^

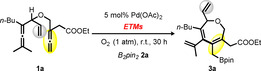		
Entry	ETMs	Yield of **3 a** [%]^[b]^
1	none	0^[c]^
2	20 mol % BQ	15
3	10 mol % Co(salophen), 20 mol % BQ	48
4	10 mol % Co(salophen), 20 mol % HQ	35
**5**	**10 mol % Co(salophen)‐HQ (Cat. 1)**	**78** ^[d]^
6	100 mol % BQ	78^[d]^

[a] Unless otherwise noted, the following reaction condition were employed with **1 a** (0.1 mmol, 1.0 equiv), B_2_pin_2_ **2 a** (0.13 mmol, 1.3 equiv), Pd(OAc)_2_ (5 mol %), ETMs (10–20 mol %) in 0.1 m acetone, O_2_ (1 atm) at room temperature (25 °C) for 30 h. [b] Yields were determined by ^1^H NMR using anisole as internal standard. [c] 93 % of starting material **1 a** was recovered. [d]<1 % of starting material **1 a** was recovered.

It is noteworthy that the Schiff base‐hydroquinone hybrid catalyst (Co(salophen)‐HQ, **Cat. 1**) leads to significant improvement of the transformation of **1 a** to **3 a** (78 % yield). This interesting result motivated us to further explore the catalytic performance of various hybrid catalysts synthesized above (vide supra). As shown in Figure 1, compared to Co‐based catalyst (**Cat. 1**), other metal‐based catalysts with Fe, Ni and Cu (**Cat. 2**–**4**) were less effective in this reaction. This result indicates that the metal center of the salen catalyst has a significant influence in the oxygen activation step.^[22]^ Notably, functional groups such as Cl (**Cat. 5**) or OMe (**Cat. 6**) on the diamine backbone resulted in little change of the reaction yields (77 % and 74 %, respectively). The hybrid catalysts with aliphatic diamine backbones, such as ethylenediamine (**Cat. 7**), (±)‐ *trans*‐1,2‐diaminocyclohexane (**Cat. 8**) and *N‐(*3‐aminopropyl)‐*N*‐methylpropane‐1,3‐diamine (**Cat. 9**) exhibited slightly lower reactivity (58–63 %). Therefore, the bifunctional catalyst Co(salophen)‐HQ was found to be the best performing catalyst in this aerobic carbocyclization to a seven‐membered heterocycle.

**Figure 1:**
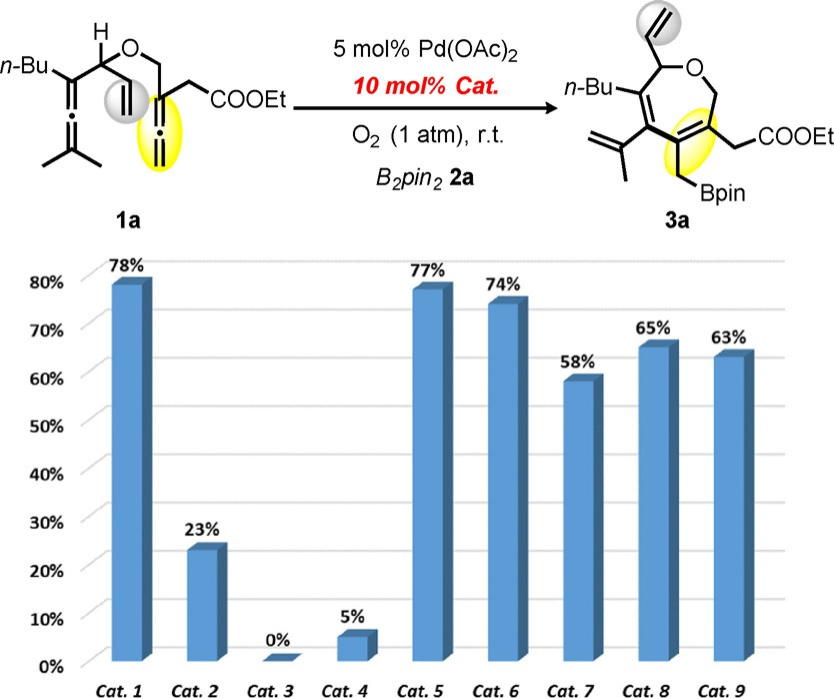
Evaluation of the bifunctional catalysts for aerobic carbocyclization of bisallene **1 a** with B_2_pin_2_ to seven membered cycle **3 a**. Unless otherwise noted, the following reaction condition were employed with **1 a** (0.1 mmol, 1.0 equiv), B_2_pin_2_ **2 a** (0.13 mmol, 1.3 equiv), Pd(OAc)_2_ (5 mol %), Schiff base‐hydroquinone catalyst (10 mol %) in 0.1 m acetone, O_2_ (1 atm) at room temperature (25 °C) for 30 h. Yields were determined by ^1^H NMR using anisole as internal standard.

After optimizing of the reaction conditions, we explored the substrate scope with the optimal oxidation catalyst Co(salophen)‐HQ (Scheme 4). The benchmark borylative carbocyclization gave the corresponding product **3 a** in 75 % isolated yield. With R^1^ being a benzyl or a cyclohexyl group, the corresponding products **3 b** and **3 c** were obtained in 78 % and 60 % yields, respectively. Under the optimal aerobic reaction conditions, cyclohexylidene bisallene **1 d** afforded **3 d** in only 30 % yield. We attribute this diminished reactivity to the increased steric bulk of the cyclohexyl group. Not only can a terminal olefin act as an assisting group, but also internal olefins were found to promote the reaction as shown by the formation of products **3 e** and **3 f** in 59 % and 65 % yield, respectively. In addition to an ester group on R^2^, various functional groups at R^2^ such as alkyl **1 g**, hydroxyl **1 h**, silyl **1 i**, acetate **1 j**, sulfonamide **1 k**, and imide **1 l** were compatible with the reaction conditions, highlighting the broad substrate scope of this protocol (52–75 % yields). As an example of late‐stage oxidative functionalization, an estrone‐derived substrate **1 m** efficiently participated in this reaction to afford a functionalized complex molecule **3 m** in a useful yield (78 %). Unfortunately, attempts to synthesize the *N*‐containing seven‐membered heterocycle using the nitrogen analogue of **3 g** (where oxygen in the ring had been replaced by NTs) were unsuccessful (See Supporting Information). Bis(neopentyl glycolato)diboron and bis(hexylene glycolato)diboron also worked as the borylating reagent and afforded the corresponding borylation products **3 n** and **3 o** in moderate yields (40 % and 52 %, respectively). The seven‐membered borylation product from **1 s** was not observed due to the steric hindrance of substrate **1 s**.

**Scheme 4:**
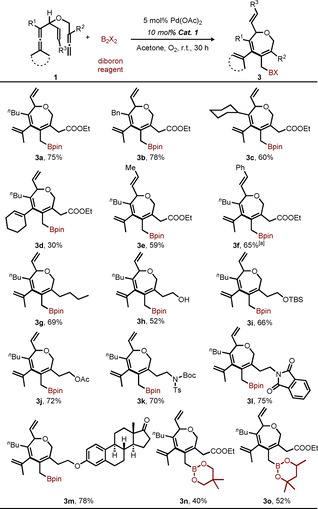
Substrate scope for aerobic borylative carbocyclization to seven‐membered rings. Reaction conditions: bisallene **1** (1.0 equiv), B_2_X_2_ **2** (1.3 equiv), Pd(OAc)_2_ (5 mol %), **Cat. 1** (10 mol %) in 0.1 m acetone, O_2_ (1 atm) at room temperature (25 °C) for 30 h; all yields are Isolated yields. [a] 15 mol % **Cat. 1** was added.

To further confirm the effect of the pending olefin group in bisallene **1**, we carried out control experiments with **1 a’** as the substrate, in which the vinyl group in **1 a** had been replaced by an ethyl group [Eq. 1]. Attempted reaction of **1 a’** under standard conditions did not give any product. This result shows that the pending olefin is an indispensable element for this oxidative carbocyclization, which is in accordance with our previous work.^[17]^

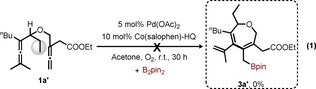



In addition to borylative oxidative carbocyclization, the use of phenylboronic acid as a transmetallating agent under similar reaction conditions also afforded the carbocyclization‐arylation product **4** in 48 % yield (Scheme 5 a). Furthermore, an efficient cascade reaction of bisallene **1 a** via an oxidative carbocyclization‐methoxycarbonylation is demonstrated here (Scheme 5 b) and gave the seven‐membered carbocycle **5** in 78 % yield. In the absence of a trapping reagent, an intramolecular oxidative coupling ending with β‐H elimination produced a highly conjugated seven‐membered ring **6** in 79 % yield (Scheme 5 c).

**Scheme 5:**
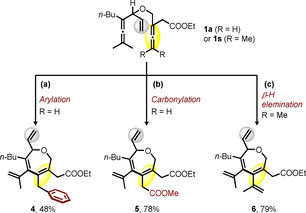
Synthesis of seven membered rings via arylative, carbonylative and beta‐H eliminative carbocyclization. Reaction conditions: (a) bisallene **1 a** (1.0 equiv), PhB(OH)_2_ (1.3 equiv), Pd(OAc)_2_ (5 mol %), **Cat. 1** (10 mol %), in 0.1 m acetone, O_2_ (1 atm) at 25 °C for 30 h; (b) bisallene **1 a** (1.0 equiv), Pd(OAc)_2_ (5 mol %), DMSO (20 mol %), BQ (1.5 equiv), MeOH (5.0 equiv), CO (1 atm) in 0.1 m DCE at 25 °C for 30 h; (c) bisallene **1 s** (1.0 equiv), Pd(OAc)_2_ (5 mol %), **Cat. 1** (10 mol %) in 0.1 m THF, O_2_ (1 atm) at 25 °C for 30 h.

In analogy with our previous report,^[15]^ there was a rate enhancement with the hybrid catalyst compared to the use of Co(salophen) and quinone as separate molecules as shown in Figure 2. Co(salophen)‐HQ hybrid (**Cat. 1**) resulted in fast borylative carbocyclization of **1 g** to **3 g** (Figure 2 a), as well as the direct carbocyclization of **1 s** to **6** ending with β‐H elimination (Figure 2 b). These results indicate that the intramolecular electron transfer between the hydroquinone and the oxidized metal‐macrocycle of this bifunctional catalyst leads to a more efficient palladium reoxidation process under aerobic conditions.^[23]^

**Figure 2:**
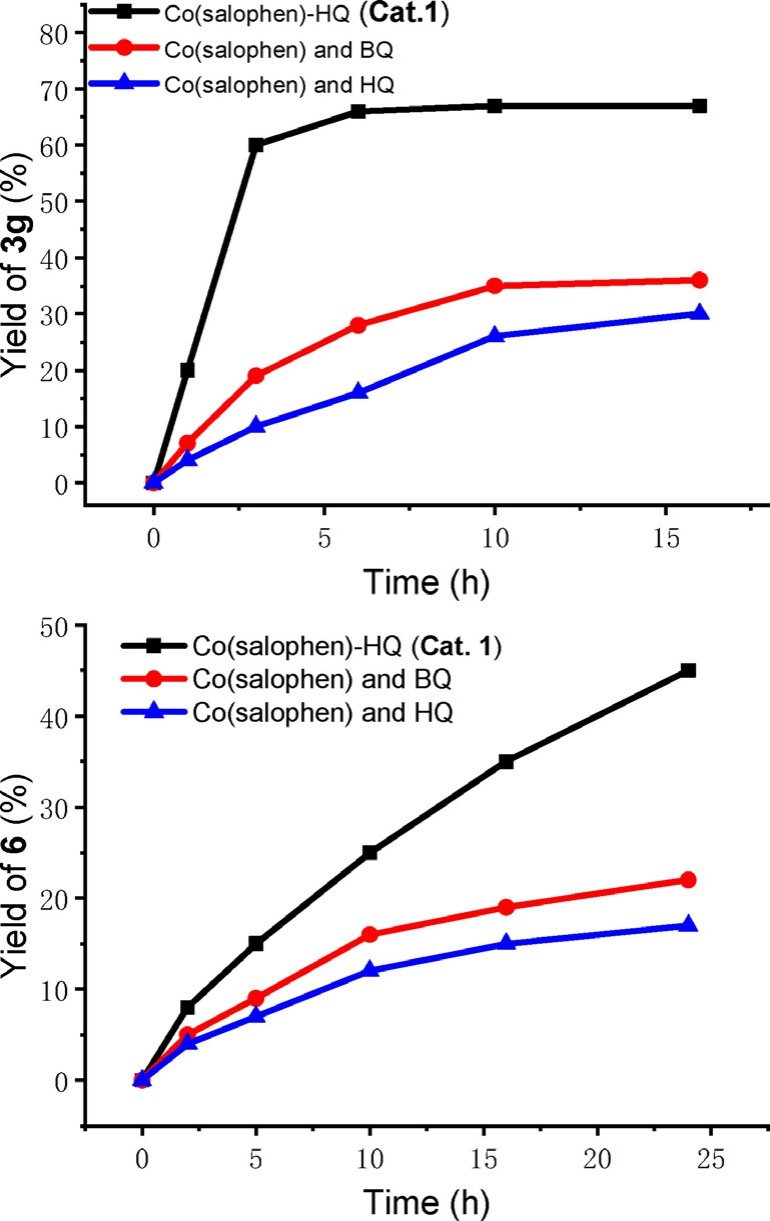
Reaction profiles with different ETMs: (a) Borylative carbocyclization of **1 g** to **3 g**; (b) Direct intramolecular carbocyclization of **1 s** to **6**. For details, please see the supporting information.

On the basis of these experimental findings, a possible mechanism for the oxidative coupling reaction is proposed in Scheme 6. Initially, the coordination of allene and olefin units to the Pd^II^ center leads to a chelate palladium complex ***Int‐I***. This special coordination of the close‐by olefin to Pd^II^ is essential for triggering the allenic C(sp^3^)−H cleavage and generating a vinylpalladium intermediate ***Int‐II***.^[17]^ Next, the envisioned ligand exchange of olefin by the distant allene moiety takes place to give ***Int‐III***. Subsequent carbocyclization of ***Int‐III*** by the second allene insertion gives a seven‐membered (π‐allyl)palladium intermediate ***Int‐IV***. Reaction of ***Int‐IV*** with a trapping reagent such as B_2_pin_2_, PhB(OH)_2_ or CO/MeOH provides the target product and a Pd^0^ species, respectively. Finally, with the assistance of the cobalt hybrid catalyst, aerobic oxidation of Pd^0^ regenerates Pd^II^ to close the catalytic cycle.

**Scheme 6:**
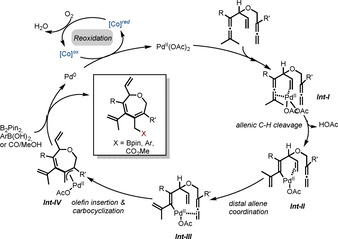
Proposed catalytic cycle.

In summary, we have developed an efficient and selective palladium‐catalyzed carbocyclization of bisallenes under aerobic oxidative conditions. This reaction avoids overstoichiometric amounts of non‐environmentally friendly oxidants (Cu^II^, Ag^I^, peroxide etc.) for the activation of allenic C−H bonds. The use of molecular oxygen as a green oxidant allows for synthesis of important seven‐membered heterocycles in moderate to good yields. The key to success of this transformation is the application of a special hybrid electron transfer mediator [Co(salophen)‐HQ]—a bifunctional catalyst consisting of a metal‐macrocycle and quinone moieties. This hybrid catalyst significantly facilitates the reoxidation of Pd^0^ to Pd^II^ using molecular oxygen as the terminal oxidant. In view of the high reaction efficiency and selectivity, this protocol is expected to complement the current approach for oxidative functionalization in the synthesis of natural products and pharmacologically active substances.

## Conflict of interest

The authors declare no conflict of interest.

## Supporting information

As a service to our authors and readers, this journal provides supporting information supplied by the authors. Such materials are peer reviewed and may be re‐organized for online delivery, but are not copy‐edited or typeset. Technical support issues arising from supporting information (other than missing files) should be addressed to the authors.

Supplementary
