# Supplementary material for: Efficient Palladium‐Catalyzed Aerobic Oxidative Carbocyclization to Seven‐Membered Heterocycles
Source: Chemistry. 2020 Oct 22;26(67):15513–8. doi: 10.1002/chem.202004265 (PMC7894550; doi:10.1002/chem.202004265)

Chemistry–A European Journal

Supporting Information

Efficient Palladium-Catalyzed Aerobic Oxidative Carbocyclization to Seven-Membered Heterocycles

Jie Liu*[a, c] and Jan-E. Bäckvall*[a, b]

Table of Content

General considerations..S1
1. Synthesis of starting materials 1..S2
2. Synthesis of bifunctional oxidation catalysts..S10
3. General procedure for Pd-catalyzed aerobic carbocyclization of bisallenes..S15
4. Experimental procedure for the reaction progress with different ETMs..S17
5. Characterization of products 3a to 6..S19
6. Spectra of catalysts, starting materials and products..S26

General considerations

Unless otherwise noted, all reagents were used as received from the commercial suppliers. $Pd(OAc)_2$, B_2pin_2 and $PhB(OH)_2$ were commercial available from Sigma Aldrich. Acetone was purchased from VWR Chemicals (≥99.8%) and used without further purification. Reactions were monitored using thin-layer chromatography (SiO_2). TLC plates were visualized with UV light (254 nm) or $KMnO_4$ stain. Flash chromatography was carried out with 60 Å (particle size 35-70 µm) normal flash silica gel. NMR spectra were recorded at 400 MHz (^{1}H) and at 100 MHz (^{13}C), respectively. Chemical shifts (δ) are reported in ppm, using the residual solvent peak in *$CDCl_3$* (H = 7.26 and C = 77.0 ppm) or in *d^6-DMSO* (H = 2.50 and C = 40.0 ppm) as the internal standard, and coupling constants (*J*) are given in Hz. Multiplets were assigned as s (singlet), d (doublet), t (triplet), dd (doublet of doublet), m (multiplet) and br (broad peak). All measurements were carried out at room temperature (25 °C) unless otherwise stated. UV characterization was recorded using a Varian Cary 50 UV-Vis spectrophotometer**.** HRMS were recorded using ESI-TOF techniques.

1. Synthesis of starting materials 1

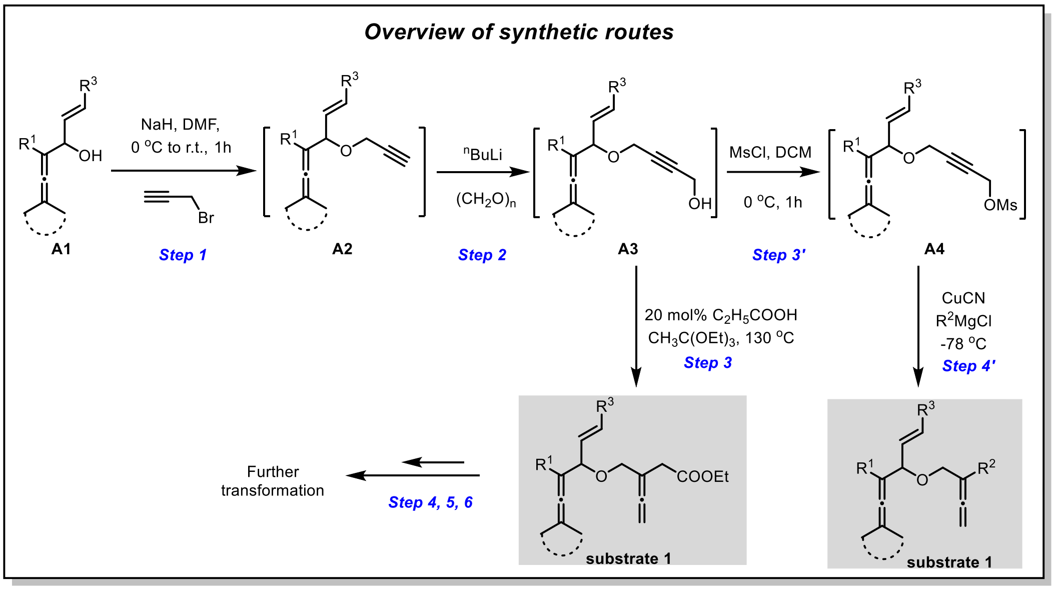

Step 1

To a dry round flask were added a solution of **A1**[1] (10 mmol) in DMF (20 mL) and NaH (60% purity, 12 mmol) sequentially at 0 °C. After the mixture was stirred at room temperature for 10 min, propargyl bromide (80% in toluene, 15 mmol) were added sequentially. After the reaction was stirred at room temperature for 1 h, H_2O (10 mL) was carefully added to quench the reaction and diluted with 50 ml Et_2O. The organic layer was washed with H_2O three times (3 × 20 mL). The organic layers were dried over Na_2SO_4, evaporated *in vacuo* to give the **A2**. This material was used directly in the next reaction.

Step 2

To a solution of **A2** in THF (20 mL) were added ^{n}BuLi (4.4 mL, 2.5 M in hexane, 11 mmol) sequentially at -78 °C. After the mixture was stirred at room temperature for 10 min, paraformaldehyde (30 mmol) was added at -78 °C. The reaction was allowed to warm to room temperature and stirred for additional 5 h. H_2O (1 mL) was added to quench the reaction and the solution was filtered through a pad of silica gel, and evaporated *in vacuo* to give the **A3** without further purification.

Step 3

To a dry round bottom flask was added the above corresponding propargylic alcohol **A3**, triethyl orthoacetate (0.3 M), and propanoic acid (20 mol%) sequentially. After the reaction was refluxed for 2 h, the mixture was cooled down to 0 °C. Then Et_2O (15 mL) and HCl (aq., 1 M, 20 mL) were added. The organic layer was separated, and the aqueous layer was extracted with Et_2O. The combined organic layers were dried over Na_2SO_4, filtered, evaporated, and purified via column chromatography on silica gel to afford the desired bisallene **1**.

[1] M.-B. Li, D. Posevins, K. P. J. Gustafson, C.-W. Tai, A. Shchukarev, Y. Qiu and J.-E. Bäckvall, *Chem. Eur. J.*, 2019, **25**, 210.

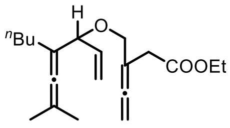

1a

Yield: 35% for 3 steps, yellow oil, R_f = 0.7 (petroleum ether/Et_2O = 10/1).
^{1}H NMR (400 MHz, $CDCl_3$) δ 5.84-5.76 (m, 1H), 5.24-5.12 (m, 2H), 4.80-4.79 (m, 2H), 4.23-4.21 (m, 1H), 4.14 (q, *J* = 7.1 Hz, 2H), 4.10-4.01 (m, 2H), 3.08-3.07 (m, 2H), 1.90-1.86 (m, 2H), 1.69 (s, 3H), 1.68 (s, 3H), 1.35-1.32 (m, 4H), 1.26 (t, *J* = 7.1 Hz, 3H), 0.90-0.87 (m, 3H);
^{13}C NMR (100 MHz, $CDCl_3$) δ 207.9, 200.1, 171.1, 137.5, 115.7, 101.4, 97.0, 94.8, 81.0, 75.9, 67.8, 60.6, 35.2, 29.9, 27.2, 22.4, 20.8, 20.6, 14.2, 14.1.
HRMS (ESI) $[C_{20}H_{30}O_3+Na]^+$ calculated mass 341.2087, measured mass 341.2090.

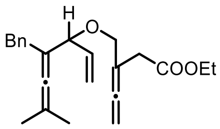

1b

Yield: 28% for 3 steps, yellow oil, R_f = 0.7 (petroleum ether/Et_2O = 10/1).
^{1}H NMR (400 MHz, $CDCl_3$) δ 7.26-7.23 (m, 2H), 7.18-7.16 (m, 3H), 5.85-5.76 (m, 1H), 5.25-5.15 (m, 2H), 4.78-4.77 (m, 2H), 4.24-4.22 (m, 1H), 4.15 (q, *J* = 7.1 Hz, 2H), 4.06-3.99 (m, 2H), 3.26 (s, 2H), 3.08-3.06 (m, 2H), 1.58 (s, 3H), 1.57 (s, 3H), 1.26 (t, *J* = 7.2 Hz, 3H);
^{13}C NMR (100 MHz, $CDCl_3$) δ 207.9, 201.5, 171.0, 140.2, 137.1, 129.1, 127.9, 125.7, 116.2, 101.5, 97.6, 94.7, 80.1, 75.9, 67.9, 60.6, 35.2, 35.0, 20.4, 20.2, 14.2.
HRMS (ESI) $[C_{23}H_{28}O_3+Na]^+$ calculated mass 375.1931, measured mass 375.1931.

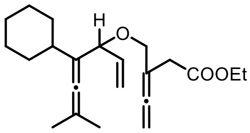

1c

Yield: 27% for 3 steps, yellow oil, R_f = 0.7 (petroleum ether/Et_2O = 10/1).
^{1}H NMR (400 MHz, $CDCl_3$) δ 5.82-5.74 (m, 1H), 5.22-5.10 (m, 2H), 4.79-4.78 (m, 2H), 4.22-4.20 (m, 1H), 4.16-4.11 (m, 2H), 4.08-4.01 (m, 2H), 3.07-3.06 (m, 2H), 1.79-1.67 (m, 11H), 1.27-1.02 (m, 9H);
^{13}C NMR (100 MHz, $CDCl_3$) δ 207.9, 200.1, 171.1, 137.7, 115.7, 107.4, 98.0, 94.9, 80.1, 75.9, 67.8, 60.6, 37.4, 35.1, 33.5, 33.2, 26.6, 26.5, 26.2, 20.8, 20.6, 14.2.
HRMS (ESI) $[C_{22}H_{32}O_3+Na]^+$ calculated mass 367.2244, measured mass 367.2238.

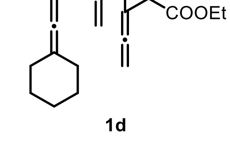

1d

Yield: 28% for 3 steps, yellow oil, R_f = 0.7 (petroleum ether/Et_2O = 10/1).

^{1}H NMR (400 MHz, CDCl$_3$) δ 5.84-5.76 (m, 1H), 5.23-5.11 (m, 2H), 4.79-4.78 (m, 2H), 4.23-4.22 (m, 1H), 4.14 (q, J = 7.1 Hz, 2H), 4.09-4.00 (m, 2H), 3.08 (s, 2H), 2.10-2.08 (m, 4H), 1.91-1.87 (m, 2H), 1.58-1.53 (m, 6H), 1.37-1.32 (m, 4H), 1.25 (t, J = 7.1 Hz, 3H), 0.88 (t, J = 7.0 Hz, 3H);
^{13}C NMR (100 MHz, CDCl$_3$) δ 207.8, 196.7, 171.1, 137.6, 115.6, 104.5, 101.2, 94.8, 81.1, 75.9, 67.7, 60.6, 35.2, 32.0, 31.7, 29.9, 27.8, 27.7, 27.1, 26.2, 22.4, 14.2, 14.1.
HRMS (ESI) $[C_{23}H_{34}O_3+Na]^+$ calculated mass 381.2400, measured mass 381.2398.

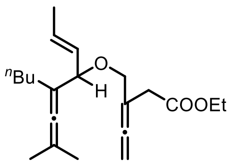

1e

Yield: 27% for 3 steps, yellow oil, R_f = 0.7 (petroleum ether/Et$_2$O = 10/1).
^{1}H NMR (400 MHz, CDCl$_3$) δ 5.68-5.58 (m, 1H), 5.48-5.42 (m, 1H), 4.79-4.78 (m, 2H), 4.18-4.11 (m, 3H), 4.07-3.97 (m, 2H), 3.08-3.07 (m, 2H), 1.90-1.85 (m, 2H), 1.71-1.67 (m, 9H), 1.34-1.32 (m, 4H), 1.25 (t, J = 7.1 Hz, 3H), 0.88 (t, J = 7.0 Hz, 3H);
^{13}C NMR (100 MHz, CDCl$_3$) δ 207.8, 199.9, 171.1, 130.4, 127.5, 101.9, 96.8, 94.9, 80.7, 75.9, 67.6, 60.6, 35.1, 29.9, 27.4, 22.5, 20.9, 20.6, 17.8, 14.2, 14.1.
HRMS (ESI) $[C_{21}H_{32}O_3+Na]^+$ calculated mass 355.2244, measured mass 355.2237.

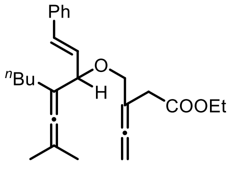

1f

Yield: 30% for 3 steps, yellow oil, R_f = 0.7 (petroleum ether/Et$_2$O = 10/1).
^{1}H NMR (400 MHz, CDCl$_3$) δ 7.39-7.37 (m, 2H), 7.33-7.29 (m, 2H), 7.24-7.21 (m, 1H), 6.56 (d, J = 15.9 Hz, 1H), 6.18 (dd, J = 15.9, 6.4 Hz, 1H), 4.82-4.81 (m, 2H), 4.41-4.39 (m, 1H), 4.18-4.08 (m, 4H), 3.11-3.10 (m, 2H), 1.98-1.92 (m, 2H), 1.71 (s, 3H), 1.70 (s, 3H), 1.38-1.33 (m, 4H), 1.24 (t, J = 7.1 Hz, 3H), 0.88 (t, J = 7.0 Hz, 3H);
^{13}C NMR (100 MHz, CDCl$_3$) δ 207.9, 200.2, 171.1, 137.1, 130.8, 129.2, 128.5, 127.4, 126.4, 101.7, 97.1, 94.8, 80.7, 76.0, 67.9, 60.7, 35.2, 30.0, 27.4, 22.4, 20.9, 20.6, 14.2, 14.1.
HRMS (ESI) $[C_{26}H_{34}O_3+Na]^+$ calculated mass 417.2400, measured mass 417.2406.

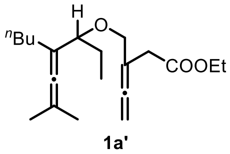

1a'

Yield: 35% for 3 steps, yellow oil, R_f = 0.7 (petroleum ether/Et$_2$O = 10/1).
^{1}H NMR (400 MHz, CDCl$_3$) δ 4.78-4.77 (m, 2H), 4.16-4.11 (m, 2H), 4.09-3.91 (m, 2H), 3.66-3.62 (m, 1H), 3.07-3.06 (m, 2H), 1.85-1.81 (m, 2H), 1.68 (s, 3H), 1.65 (s, 3H), 1.63-1.50 (m, 2H), 1.35-1.32 (m, 4H), 1.25 (t, J = 7.2 Hz, 3H), 0.90-0.85 (m, 6H);
^{13}C NMR (100 MHz, CDCl$_3$) δ 207.8, 200.4, 171.1, 100.7, 95.8, 95.0, 82.3, 75.8, 67.6, 60.6, 35.2, 29.9, 26.5, 26.0, 22.5, 20.9, 20.7, 14.2, 14.1, 10.3.

HRMS (ESI) $[C_{20}H_{32}O_3+Na]^+$ calculated mass 343.2244, measured mass 343.2237.

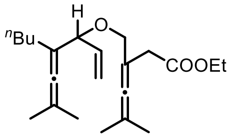

1s

Yield: 41% for 3 steps (3 equiv. acetone instead of $(CH_2O)_n$ in step 2), yellow oil, R_f = 0.7 (petroleum ether/Et_2O = 10/1).

^{1}H NMR (400 MHz, $CDCl_3$) δ 5.84-5.75 (m, 1H), 5.22-5.18 (m, 1H), 5.13-5.10 (m, 1H), 4.25-4.23 (m, 1H), 4.11 (q, *J* = 7.1 Hz, 2H), 4.02-3.94 (m, 2H), 3.02-3.01 (m, 2H), 1.91-1.87 (m, 2H), 1.68 (s, 6H), 1.67 (s, 6H), 1.36-1.30 (m, 4H), 1.24 (t, *J* = 7.1 Hz, 3H), 0.88 (t, *J* = 7.1 Hz, 3H);

^{13}C NMR (100 MHz, $CDCl_3$) δ 201.4, 200.1, 171.7, 137.7, 115.5, 101.6, 96.8, 96.4, 93.0, 80.2, 68.6, 60.4, 36.1, 29.9, 27.3, 22.4, 20.7, 20.6, 20.4, 20.4, 14.2, 14.0.

HRMS (ESI) $[C_{20}H_{32}O_3+Na]^+$ calculated mass 369.2400, measured mass 369.2409.

Step 3′ and 4′

To a solution of substrate **A3** (2 mmol) in DCM (20 mL) was added Et_3N (2.2 mmol) and MsCl (2.2 mmol) sequentially. After the reaction was stirred at 0 °C for 2h, the reaction was quenched with water (1 mL). The solution was filtered through a pad of silica gel, and evaporated *in vacuo* to give the **A4** which can be used directly in the next step without further purification.

A solution of ^{n}BuMgCl (5 mmol, 2.0 M in THF) was added dropwise to a stirred suspension of **A4** and CuCN (2.4 mmol) in dry Et_2O (10 mL) at -78 °C under Ar. The mixture was stirred for 5 h at this temperature, and then carefully quenched with citric acid aqueous solution (5 mL, 10%). The organic layer was separated, and the aqueous layer was extracted with Et_2O (2 × 20 mL). The combined organic layers were dried over Na_2SO_4, concentrated *in vacuo* and purified via column chromatography on silica gel to afford **1g**.

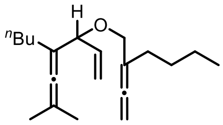

1g

Yield: 40% for 3 steps, yellow oil, R_f = 0.7 (petroleum ether/Et_2O = 10/1).

^{1}H NMR (400 MHz, $CDCl_3$) δ 5.86-5.77 (m, 1H), 5.24-5.12 (m, 2H), 4.70-4.69 (m, 2H), 4.23-4.22 (m, 1H), 4.00-3.92 (m, 2H), 2.04-2.01 (m, 2H), 1.91-1.89 (m, 2H), 1.69 (s, 3H), 1.68 (s, 3H), 1.46-1.34 (m, 8H), 0.92-0.89 (m, 6H);

^{13}C NMR (100 MHz, $CDCl_3$) δ 206.8, 200.1, 137.7, 115.6, 101.7, 100.8, 96.8, 80.7, 75.2, 68.5, 30.0, 29.6, 28.8, 27.4, 22.5, 22.5, 20.8, 20.6, 14.1, 13.9.

HRMS (ESI) $[C_{20}H_{32}O+Na]^+$ calculated mass 311.2345, measured mass 311.2336.

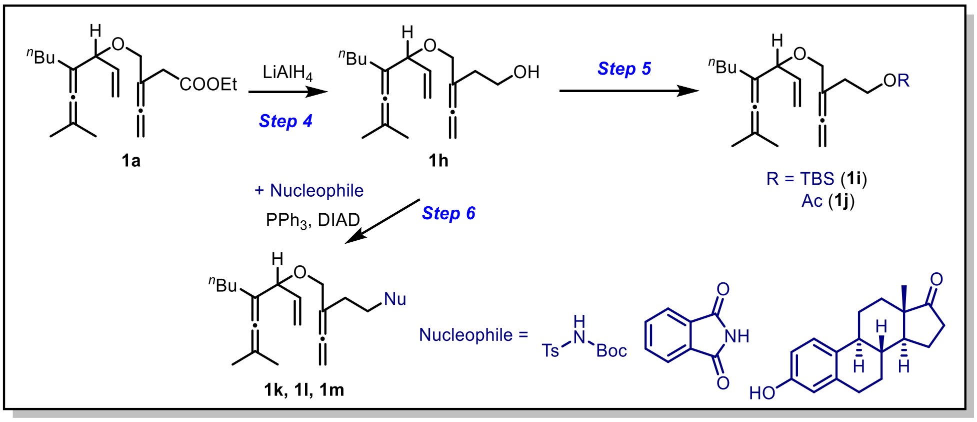

Step 4

Synthesis of **1h**:

A solution of **1a** (5 mmol) in dry Et_2O (50 mL) was added dropwise to a stirred suspension of $LiAlH_4$ (3 mmol) at 0 °C under Ar atmosphere. The mixture was stirred for 2 h at 0 °C, and then carefully quenched with H_2O (2 mL). The solution was filtered through a pad of silica gel and evaporated, and purified via column chromatography on silica gel to afford **1h**.

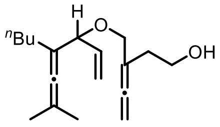

1h

Yield: 99% from **1a**, yellow oil, R_f = 0.6 (petroleum ether/Et_2O = 3/1).

^{1}H NMR (400 MHz, $CDCl_3$) δ 5.85-5.76 (m, 1H), 5.25-5.15 (m, 2H), 4.76-4.75 (m, 2H), 4.25-4.23 (m, 1H), 4.00-3.94 (m, 2H), 3.75-3.74 (m, 2H), 2.46-2.43 (m, 1H), 2.35-2.32 (m, 2H), 1.91-1.87 (m, 2H), 1.69 (s, 3H), 1.68 (s, 3H), 1.35-1.32 (m, 4H), 0.90-0.87 (m, 3H);

^{13}C NMR (100 MHz, $CDCl_3$) δ 207.7, 200.1, 137.2, 116.1, 101.3, 97.9, 97.3, 81.5, 75.2, 68.9, 61.4, 34.0, 29.9, 27.3, 22.4, 20.8, 20.6, 14.1.

HRMS (ESI) $[C_{18}H_{28}O_2+Na]^+$ calculated mass 299.1982, measured mass 299.1976.

Step 5

Synthesis of **1i**:

A solution of **1h** (0.5 mmol) in THF (5 mL) was added NaH (60%, 1.2 equiv.) and tert-butyldimethylsilyl chloride (1.05 mmol, 1.05 equiv) under Ar. The reaction mixture was stirred at room temperature for 2 h. Then the reaction was quenched with H_2O (10 mL). The organic layer was separated, and the aqueous layer was extracted with Et_2O (2 × 20 mL). The combined organic layers were dried over Na_2SO_4, filtered, and evaporated. The crude mixture was purified via column chromatography on silica gel to afford the product **1i**.

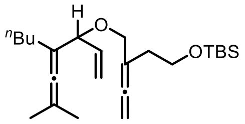

1i

Yield: 95% from **1h**, yellow oil, R_f = 0.8 (petroleum ether/Et_2O = 20/1).

^{1}H NMR (400 MHz, $CDCl_3$) δ 5.85-5.76 (m, 1H), 5.24-5.12 (m, 2H), 4.70-4.69 (m, 2H), 4.23-4.22 (m, 1H), 4.00-3.92 (m, 2H), 3.74-3.71 (m, 2H), 2.28-2.24 (m, 2H), 1.92-1.90 (m, 2H), 1.68 (s, 6H), 1.35-1.32 (m, 4H), 0.89 (s, 9H), 0.05 (s, 6H);

^{13}C NMR (100 MHz, $CDCl_3$) δ 207.1, 200.1, 137.7, 115.6, 101.6, 97.5, 96.9, 80.8, 75.3, 68.6, 61.8, 32.5, 29.9, 27.3, 25.9, 22.5, 20.8, 20.6, 18.3, 14.1, -5.3.

HRMS (ESI) $[C_{24}H_{42}SiO_2+Na]^+$ calculated mass 413.2846, measured mass 413.2848.

Synthesis of **1j**:

To a round bottom flask were added substrate **1h** (0.5 mmol), DMAP (0.1 mmol), DCM (10 mL), Et_3N (0.6 mmol), and Ac_2O (0.6 mmol). The reaction mixture was stirred at room temperature for 12 h and then quenched with water (5 mL). The organic layer was separated, and the aqueous layer was extracted with Et_2O (2 × 20 mL). The combined organic layers were dried over Na_2SO_4 and concentrated *in vacuo*. The crude product was purified via column chromatography on silica gel to afford **1j**.

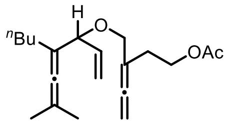

1j

Yield: 95% from **1h**, yellow oil, R_f = 0.7 (petroleum ether/Et_2O = 10/1).

^{1}H NMR (400 MHz, $CDCl_3$) δ 5.85-5.76 (m, 1H), 5.24-5.13 (m, 2H), 4.76-4.74 (m, 2H), 4.22-4.17 (m, 3H), 4.02-3.94 (m, 2H), 2.38-2.35 (m, 2H), 2.04 (s, 3H), 1.89-1.87 (m, 2H), 1.69 (s, 6H), 1.36-1.32 (m, 4H), 0.90-0.87 (m, 3H);

^{13}C NMR (100 MHz, $CDCl_3$) δ 206.8, 200.1, 171.0, 137.5, 115.8, 101.4, 97.0, 80.9, 76.1, 68.4, 62.6, 29.9, 28.3, 27.3, 22.4, 20.9, 20.8, 20.6, 14.1.

HRMS (ESI) $[C_{20}H_{30}O_3+Na]^+$ calculated mass 341.2087, measured mass 341.2081.

Step 6

Synthesis of **1k, 1l, 1m**:

A mixture of PPh_3 (1.2 mmol), di-isopropyl azodicarboxylate (DIAD, 1.2 mmol), and nucleophile (1.2 mmol) was added a solution of **1h** (0.8 mmol) in THF (5 mL) at 0 °C. The reaction was stirred at room temperature for 12 h. After full consumption of starting material **1h** as monitored by TLC, the reaction mixture was concentrated *in vacuo* and purified via column chromatography on silica gel to afford the corresponding product **1k**, **1l**, **1m**.

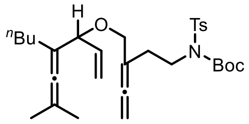

1k

Yield: 75% from **1h**, yellow oil, R_f = 0.4 (petroleum ether/Et_2O = 5/1).

^{1}H NMR (400 MHz, $CDCl_3$) δ 7.77 (d, *J* = 8.4 Hz, 2H), 7.26 (d, *J* = 7.7 Hz, 2H), 5.85-5.77 (m, 1H), 5.26-5.11 (m, 2H), 4.73-4.72 (m, 2H), 4.24-4.23 (m, 1H), 4.04-3.91 (m, 4H), 2.46-2.43 (m, 2H), 2.41 (s, 3H), 1.91-1.88 (m, 2H), 1.67 (s, 3H), 1.66 (s, 3H), 1.33-1.31 (m, 13H), 0.88-0.84 (m, 3H);

^{13}C NMR (100 MHz, $CDCl_3$) δ 206.9, 200.0, 150.7, 143.9, 137.5, 137.4, 129.1, 127.8, 115.6, 101.4, 97.4, 96.8, 83.9, 80.8, 76.0, 68.2, 45.3, 29.8, 29.3, 27.8, 27.2, 22.3, 21.5, 20.7, 20.5, 14.0.

HRMS (ESI) $[C_{30}H_{43}NSO_5+Na]^+$ calculated mass 552.2754, measured mass 552.2758.

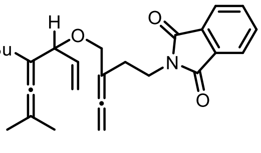

1l

Yield: 73% from **1h**, yellow oil, R_f = 0.6 (petroleum ether/Et_2O = 5/1).

^{1}H NMR (400 MHz, $CDCl_3$) δ 7.85-7.83 (m, 2H), 7.72-7.70 (m, 2H), 5.85-5.76 (m, 1H), 5.26-5.12 (m, 2H), 4.65-4.64 (m, 2H), 4.24-4.23 (m, 1H), 4.07-3.97 (m, 2H), 3.85-3.82 (m, 2H), 2.48-2.42 (m, 2H), 1.91-1.88 (m, 2H), 1.69 (s, 3H), 1.68 (s, 3H), 1.35-1.32 (m, 4H), 0.88 (t, *J* = 7.1 Hz, 3H);

^{13}C NMR (100 MHz, $CDCl_3$) δ 206.9, 200.1, 168.2, 137.5, 133.8, 132.1, 123.1, 115.7, 101.4, 97.3, 96.9, 80.9, 75.9, 68.0, 36.2, 29.9, 27.7, 27.2, 22.4, 20.8, 20.6, 14.0.

HRMS (ESI) $[C_{26}H_{31}NO_3+Na]^+$ calculated mass 428.2196, measured mass 428.2202.

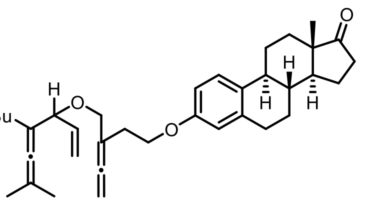

1m

Yield: 70% from **1h**, yellow oil, R_f = 0.75 (petroleum ether/Et_2O = 10/1).

^{1}H NMR (400 MHz, $CDCl_3$) δ 7.20-7.17 (m, 1H), 6.73-6.70 (m, 1H), 6.65-6.64 (m, 1H), 5.87-5.79 (m, 1H), 5.27-5.14 (m, 2H), 4.78-4.77 (m, 2H), 4.27-4.26 (m, 1H), 4.10-4.02 (m, 4H), 2.90-2.87 (m, 2H), 2.53-2.51 (m, 3H), 2.41-2.37 (m, 1H), 2.25-2.24 (m, 1H), 2.16-1.90 (m, 6H), 1.70 (s, 6H), 1.63-1.35 (m, 10H), 0.91-0.88 (m, 6H);

^{13}C NMR (100 MHz, $CDCl_3$) δ 220.9, 206.9, 200.1, 156.9, 137.6, 137.6, 131.9, 126.2, 115.7, 114.7, 112.2, 101.5, 97.3, 97.0, 80.9, 75.9, 68.7, 66.2, 50.4, 48.0, 44.0, 38.4, 35.9, 31.6, 29.9, 29.6, 29.1, 27.3, 26.6, 25.9, 22.4, 21.6, 20.8, 20.6, 14.1, 13.8.

HRMS (ESI) $[C_{36}H_{48}O_3+Na]^+$ calculated mass 551.3496, measured mass 551.3492.

Synthesis of **1-NTs**:

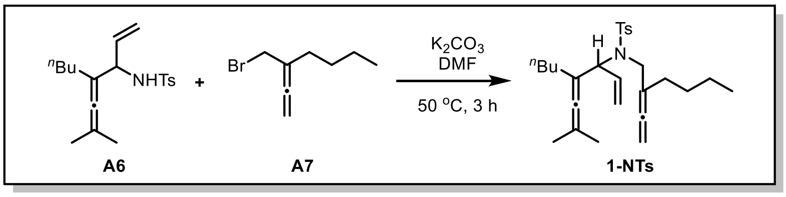

To a solution of **A6** (0.5 mmol) and **A7** (0.6 mmol) was added K_2CO_3 (1.0 mmol) in 10 ml DMF. At room temperature. The reaction was heated to 50 °C and stirred for 3 h. Then the reaction was quenched with H_2O (10 mL) and diluted with 20 ml Et_2O. The organic layer was separated, and washed with water three times (3 × 10 mL). The organic layers were dried over Na_2SO_4, filtered, and evaporated. The crude mixture was purified via column chromatography on silica gel to afford the product **1-NTs**.

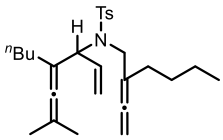

1-NTs

Yield: 51%, yellow oil, R_f = 0.6 (petroleum ether/Et_2O = 5/1).

^{1}H NMR (400 MHz, $CDCl_3$) δ 7.69 (d, *J* = 8.2 Hz, 2H), 7.27 (d, *J* = 8.2 Hz, 2H), 5.95-5.91 (m, 1H), 5.14-5.06 (m, 1H), 4.63-4.62 (m, 2H), 3.84-3.81 (m, 2H), 3.76-3.75 (m, 2H), 2.41 (s, 3H), 1.93-1.90 (m, 2H), 1.87-1.83 (m, 2H), 1.66 (s, 6H), 1.38-1.26 (m, 8H), 0.90-0.86 (m, 6H);

^{13}C NMR (100 MHz, $CDCl_3$) δ 207.2, 204.4, 142.9, 137.7, 134.5, 129.5, 127.2, 119.7, 101.9, 99.1, 95.7, 76.2, 49.2, 49.1, 29.7, 29.4, 28.5, 28.4, 22.4, 22.3, 21.4, 20.4, 13.9, 13.9.

HRMS (ESI) $[C_{27}H_{39}SNO_2+Na]^+$ calculated mass 464.2594, measured mass 464.2591.

2. Synthesis of bifunctional oxidation catalysts

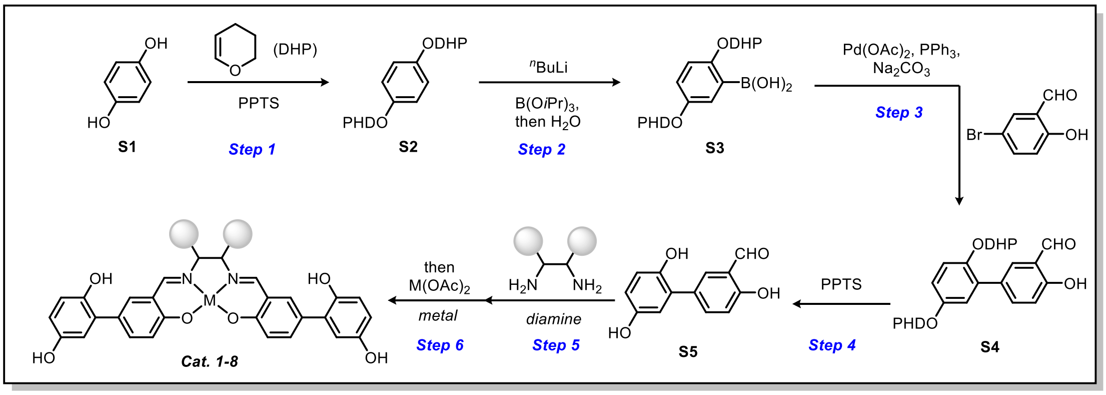

Step 1

Hydroquinone **S1** (200 mmol) was suspended in dichloromethane (200 mL). 3,4-Dihydro-2*H*-pyran (500 mmol, 2.5 equiv.) and PPTS (1.0 mmol) were added, and the mixture was stirred at room temperature for 2 h. The solution was filtered through a pad of silica gel, and concentrated to give **S2** which is pure enough for next step.

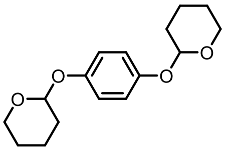

S2

Yield: quant., white solid, R_f = 0.7 (petroleum ether/EA = 10/1).[2]

^{1}H NMR (400 MHz, d^6-DMSO) δ 6.94 (s, 4H), 5.32 (t, *J* = 3.5 Hz, 2H), 3.80-3.75 (m, 2H), 3.54-3.49 (m, 2H), 1.88-1.52 (m, 12H);

^{13}C NMR (100 MHz, d^6-DMSO) δ 151.7, 151.7, 118.0, 97.0, 96.9, 61.9, 30.4, 25.2, 19.2.

Step 2 and 3

Compound **S2** (50 mmol) was dissolved in dry THF (100 mL) under argon. *n*-Butyllithium in hexanes (20 mL, 2.5 M, 50 mmol) was added dropwise by syringe at room temp. After stirring at room temp. for 2 h, the reaction was cooled to –78 °C, and B(O*i*Pr)$_3$ (100 mmol) was added dropwise. After stirring at –78 °C for 30 min, the reaction mixture was warmed to r.t. for 2 h. The reaction mixture was quenched with water (100 mL) and stirring for 30 min. The reaction mixture was extracted with ethyl acetate (2 × 100 mL). The organic layer was dried with Na_2SO_4, concentrated under vacuum, and the resulting crude product **S3** was used directly without further purification in the Suzuki coupling.

Compound **S3** and 5-bromo-2-hydroxybenzaldehyde (50 mmol) were dissolved in toluene (90 mL). To this mixture was added a solution of Na_2CO_3 (100 mmol) and $BnEt_3NCl$ (2 mmol) in water (30 mL). Ethanol (30 mL) was then added, followed by a solution of of $Pd(OAc)_2$ (0.2 mmol) and PPh_3 (0.8 mmol) in toluene (30 mL). The mixture was purged with argon for 10 min, and then stirred under argon at 100 °C for 4 h. After cooling to room temp., the mixture was diluted with brine (100 mL) and extracted with ethyl acetate (3 × 100 mL). The combined organic layers were concentratedand purified by flash chromatography to afford the product **S4**.

---

2 E. V. Johnston, E. A. Karlsson, L.-H. Tran, B. Aakermark and J.-E. Baeckvall, *Eur. J. Org. Chem.*, 2009, 3973.

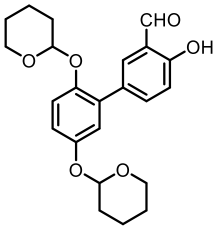

S4

Yield: 95% for 2 steps, yellow oil, R_f = 0.5 (petroleum ether/EA = 5/1).

^{1}H NMR (400 MHz, $CDCl_3$) δ 11.01 (s, 1H), 9.93 (s, 1H), 7.79-7.77 (m, 2H), 7.16 (d, J = 8.9 Hz, 1H), 7.04-7.01 (m, 2H), 6.97-6.96 (m, 1H), 5.38-5.37 (m, 1H), 5.28-5.26 (m, 1H), 3.98-3.91 (m, 1H), 3.81-3.76 (m, 1H), 3.62-3.57 (m, 2H), 2.07-1.50 (m, 12H);

^{13}C NMR (100 MHz, $CDCl_3$) δ 196.7, 160.6, 152.1, 148.5, 138.5, 134.5, 130.5, 130.4, 120.3, 118.5, 117.5, 117.4, 117.0, 116.7, 97.6, 97.1, 62.0, 62.0, 30.4, 30.4, 25.2, 25.2, 18.8, 18.7.

Step 4

Compound **S4** was dissolved in ethanol (90 mL), followed by addition of water (10 mL). Pyridinium *p*-toluenesulfonate (0.4 mmol) was then added, and the mixture was stirred at 60 °C for 2 h. After cooling to room temp., the reaction mixture was quenched with pyridine (0.32 mL, 4 mmol). The mixture was concentrated in vacuo to afford a yellow oil and recrystallization in $CHCl_3$ to give hydroquinone–salicylaldehyde **S5** (95 %).

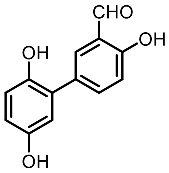

S5

Yield: 95%, yellow solid, R_f = 0.3 (petroleum ether/EA = 2/1).

^{1}H NMR (400 MHz, d^{6}-DMSO) δ 10.71 (s, 1H), 10.29 (s, 1H), 8.81 (s, 1H), 8.78 (s, 1H), 7.82 (d, J = 2.4 Hz, 1H), 7.70 (dd, J = 8.6, 2.4 Hz, 1H), 7.02 (d, J = 8.6 Hz, 1H), 6.73 (d, J = 8.6 Hz, 1H), 6.65 (d, J = 3.0 Hz, 1H), 6.56 (dd, J = 8.6, 3.0 Hz, 1H);

^{13}C NMR (100 MHz, d^{6}-DMSO) δ 192.3, 160.0, 150.6, 147.1, 137.5, 130.5, 129.7, 127.2, 122.3, 117.3, 117.3, 116.4, 115.4.

Step 5 and 6

Hydroquinone–salicylaldehyde **S5** (2 mmol) and diamine (1 mmol) were placed in a round-bottomed flask and methanol (10 ml) was added. The reaction mixture was allowed to stir for 16 h at room temperature. Metal acetate salt ($Co(OAc)_2$, $Fe(OAc)_2$, $Ni(OAc)_2$ or $Cu(OAc)_2$, 1.2 equiv. to diamine) was added to the above solution under argon. The reaction was then heated at 62 ^{o}C for 2 h and cooled to room temperature. Degassed water (10 ml) was added to complete the precipitation, and the product was collected by filtration, washing with degassed water (2 × 5 mL) to remove excess metal acetate. The pure product was dried under vacuum at 100 ^{o}C for 10 h to give the **Cat. 1**-**9**.

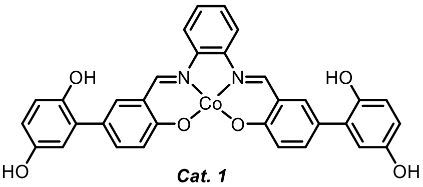

Cat. 1

Yield: 85%, dark brown solid.[3]

UV-visible (MeOH): λ_{max} = 498 nm (ε_{max} = 7.6 × 10^4 $M^{-1}cm^{-1}$);

HRMS (ESI) $[C_{32}H_{18}CoN_2O_6]^+$ (oxidized form) calculated mass 585.0491, measured mass 585.0483.

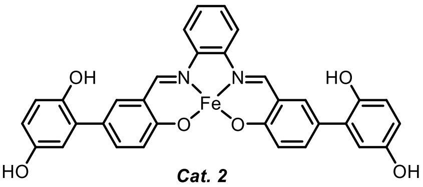

Cat. 2

Yield: 85%, dark brown solid.

UV-visible (MeOH): λ_{max} = 435 nm (ε_{max} = 3.1 × 10^4 $M^{-1}cm^{-1}$);

HRMS (ESI) $[C_{32}H_{18}FeN_2O_6]^+$ (oxidized form) calculated mass 582.0509, measured mass 582.0512.

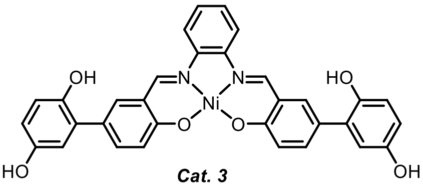

Cat. 3

Yield: 90%, brown solid.

^{1}H NMR (400 MHz, d^6-DMSO) δ 8.96 (s, 2H), 8.74 (d, *J* = 7.2 Hz, 4H), 8.19 (dd, *J* = 6.4, 3.2 Hz, 2H), 7.78 (d, *J* = 2.4 Hz, 2H), 7.57 (dd, *J* = 8.9, 2.4 Hz, 2H), 7.35 (dd, *J* = 6.4, 3.2 Hz, 2H), 6.93 (d, *J* = 8.9 Hz, 2H), 6.74 (d, *J* = 8.6 Hz, 2H), 6.69 (d, *J* = 3.0 Hz, 2H), 6.54 (dd, *J* = 8.6, 3.0 Hz, 2H);

^{13}C NMR (100 MHz, d^6-DMSO) δ 164.9, 157.1, 150.6, 147.2, 142.9, 137.2, 134.2, 128.1, 127.8, 126.5, 120.4, 120.0, 117.2, 116.7, 116.4, 114.7.

UV-visible (MeOH): λ_{max} = 376 nm (ε_{max} = 2.4 × 10^4 $M^{-1}cm^{-1}$), 480 nm (ε_{max} = 1.8 × 10^4 $M^{-1}cm^{-1}$);

HRMS (ESI) $[C_{32}H_{22}NiN_2O_6+Na]^+$ calculated mass 611.0724, measured mass 611.0719.

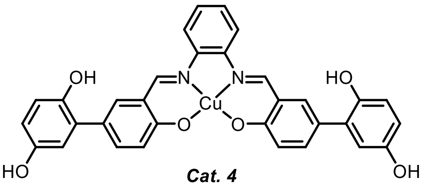

Cat. 4

Yield: 89%, dark brown solid.

UV-visible (MeOH): λ_{max} = 496 nm (ε_{max} = 2.5 × 10^4 $M^{-1}cm^{-1}$);

HRMS (ESI) $[C_{32}H_{18}CuN_2O_6]^+$ (oxidized form) calculated mass 589.0455, measured mass 589.0454.

[3] J. Liu, A. Ricke, B. Yang and J.-E. Bäckvall, *Angew. Chem. Int. Ed.*, 2018, **57**, 16842.

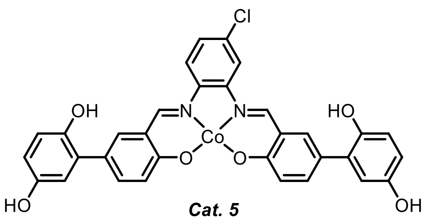

Cat. 5

Yield: 95%, dark brown solid.
UV-visible (MeOH): λ_{max} = 496 nm (ε_{max} = 7.9 × 10^4 $M^{-1}cm^{-1}$);
HRMS (ESI) $[C_{32}H_{17}CoClN_2O_6]^+$ (oxidized form) calculated mass 619.0102, measured mass 619.0093.

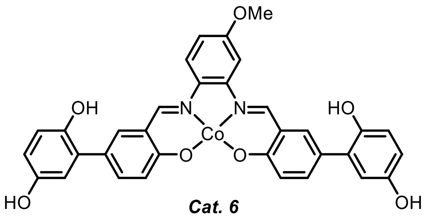

Cat. 6

Yield: 79%, dark brown solid.
UV-visible (MeOH): λ_{max} = 490 nm (ε_{max} = 7.6 × 10^4 $M^{-1}cm^{-1}$);
HRMS (ESI) $[C_{33}H_{20}CoN_2O_7]^+$ (oxidized form) calculated mass 615.0607, measured mass 615.0597.

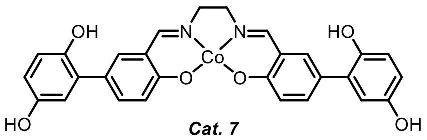

Cat. 7

Yield: 78%, dark brown solid.
UV-visible (MeOH): λ_{max} = 521 nm (ε_{max} = 3.2 × 10^4 $M^{-1}cm^{-1}$);
HRMS (ESI) $[C_{28}H_{18}CoN_2O_6]^+$ (oxidized form) calculated mass 537.0491, measured mass 537.0487.

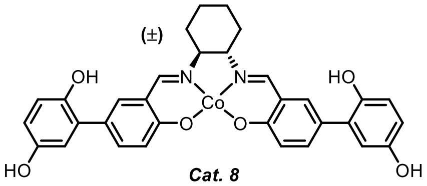

Cat. 8

Yield: 91%, dark brown solid.
UV-visible (MeOH): λ_{max} = 524 nm (ε_{max} = 3.6 × 10^4 $M^{-1}cm^{-1}$);
HRMS (ESI) $[C_{32}H_{24}CoN_2O_6]^+$ (oxidized form) calculated mass 589.0961, measured mass 589.0947.

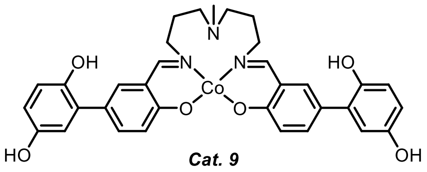

Cat. 9

Yield: 80%, dark brown solid.[4]

[4] B. W. Purse, L.-H. Tran, J. Piera, B. Åkermark and J.-E. Bäckvall, *Chem. Eur. J.*, 2008, **14**, 7500

UV-visible (MeOH): λ_{max} = 502 nm (ε_{max} = 3.6 × 10^4 $M^{-1}cm^{-1}$);

HRMS (ESI) $[C_{33}H_{29}CoN_3O_6]^+$ (oxidized form) calculated mass 622.1383, measured mass 622.1386.

3. General procedure for Pd-catalyzed aerobic carbocyclization of bisallenes

1. Borylation

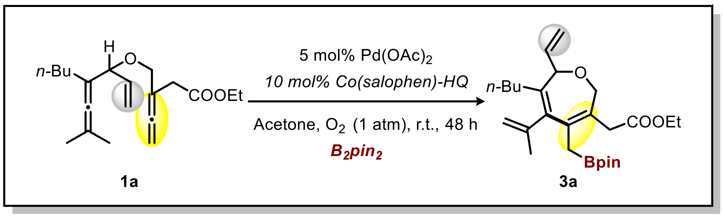

A microwave tube (10 mL) was charged with $Pd(OAc)_2$ (0.005 mmol, 5 mol%), Co(salophen)-HQ (0.01 mmol, 10 mol%), B_2pin_2 **2a** (0.13 mmol, 1.3 equiv. to **1a**) and a stirring bar was added. Then, a solution of bisallene **1a** (0.1 mmol) in acetone (1 mL, 0.1 M) were injected by syringe. The reaction mixture was purged briefly with oxygen, and equipped with an O_2 balloon then stirred at room temperature (25 °C) for 30 h. After full consumption of bisallene **1a** monitored by TLC, the reaction mixture was diluted with Et_2O (5 mL), and quickly filtered via a short column of silica gel (5 cm, eluent: 30 mL of Et_2O). The collected filtrate solution was evaporated and determined by ^{1}H-NMR of the crude product mixture. Purification via column chromatography on silica gel afforded the desired product **3a**.

2. Arylation

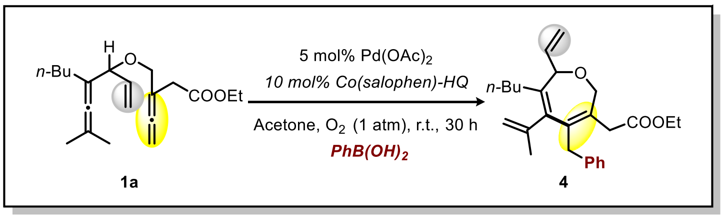

A microwave tube (10 mL) was charged with $Pd(OAc)_2$ (0.005 mmol, 5 mol%), Co(salophen)-HQ (0.01 mmol, 10 mol%), $PhB(OH)_2$ (0.13 mmol, 1.3 equiv. to **1a**) and a stirring bar was added. Then, a solution of bisallene **1a** (0.1 mmol) in acetone (1 mL, 0.1 M) were injected by syringe. The reaction mixture was purged briefly with oxygen, and equipped with an O_2 balloon then stirred at room temperature (25 °C) for 30 h. After full consumption of bisallene **1a** monitored by TLC, the reaction mixture was diluted with Et_2O (5 mL), and quickly filtered via a short column of silica gel (5 cm, eluent: 30 mL of Et_2O). The collected filtrate solution was evaporated and determined by ^{1}H-NMR of the crude product mixture. Purification via column chromatography on silica gel afforded the desired product **4**.

3. Alkoxycarbonylation

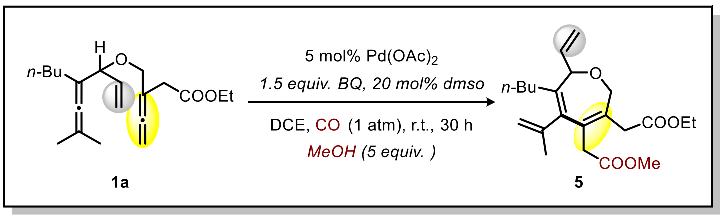

A microwave tube (10 mL) was charged with $Pd(OAc)_2$ (0.005 mmol, 5 mol%), BQ (0.15 mmol, 1.5 equiv. to **1a**) and a stirring bar was added. Then, a solution of bisallene **1a** (0.1 mmol) in DCE (1 mL, 0.1 M) were injected by syringe and followed by the addition of MeOH (5.0 equiv. to **1a**) and DMSO (20 mol%). The reaction mixture was purged briefly with CO, and equipped with a CO balloon then stirred at room temperature (25 ^oC) for 30 h. After full consumption of bisallene **1a** monitored by TLC, the reaction mixture was diluted with Et_2O (5 mL), and quickly filtered via a short column of silica gel (5 cm, eluent: 30 mL of Et_2O). The collected filtrate solution was evaporated and determined by ^1H-NMR of the crude product mixture. Purification via column chromatography on silica gel afforded the desired product **5**.

4. Carbocyclization via β-H elimination

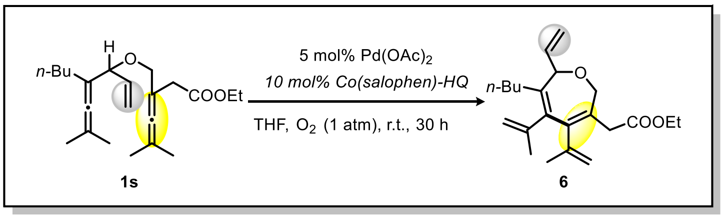

A microwave tube (10 mL) was charged with $Pd(OAc)_2$ (0.005 mmol, 5 mol%), Co(salophen)-HQ (0.01 mmol, 10 mol%) and a stirring bar was added. Then, a solution of bisallene **1s** (0.1 mmol) in THF (1 mL, 0.1 M) were injected by syringe. The reaction mixture was purged briefly with oxygen, and equipped with an O_2 balloon then stirred at room temperature (25 ^oC) for 30 h. After full consumption of bisallene **1s** monitored by TLC, the reaction mixture was diluted with Et_2O (5 mL), and quickly filtered via a short column of silica gel (5 cm, eluent: 30 mL of Et_2O). The collected filtrate solution was evaporated and determined by ^1H-NMR of the crude product mixture. Purification via column chromatography on silica gel afforded the desired product **6**.

4. Experimental procedure for the reaction progress with different ETMs

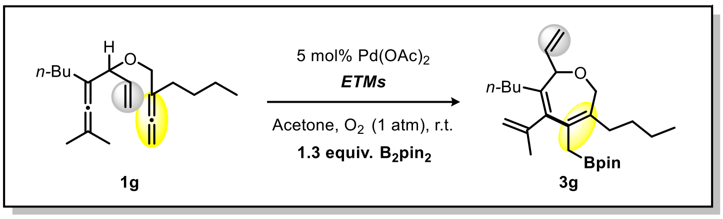

ETM employing ***5 mol% Co(salophen)-HQ***:

A microwave tube (10 mL) was charged with $Pd(OAc)_2$ (5 mol%), B_2pin_2 (1.3 equiv.) and **Co(salophen)-HQ** (5 mol%). Then, a solution of bisallene **1g** (0.1 mmol) in acetone (1 mL) were injected by syringe, respectively. The reaction mixture was stirred at 25 °C under atmosphere of O_2. After a specific time, the reaction mixture was diluted with Et_2O (5 mL), and quickly filtered via a short column of silica gel (5 cm, eluent: 30 mL of Et_2O). The collected filtrate solution was evaporated and the yield of **3g** was determined by ^{1}H-NMR using anisole as the internal standard.

ETMs employing ***5 mol% Co(salophen) and 10 mol% BQ***:

A microwave tube (10 mL) was charged with $Pd(OAc)_2$ (5 mol%), B_2pin_2 (1.3 equiv.) and **Co(salophen)** (5 mol%). Then, a solution of bisallene **1g** (0.1 mmol) and **BQ** (10 mol%) in acetone (1 mL) were injected by syringe, respectively. The reaction mixture was stirred at 25 °C under atmosphere of O_2. After a specific time, the reaction mixture was diluted with Et_2O (5 mL), and quickly filtered via a short column of silica gel (5 cm, eluent: 30 mL of Et_2O). The collected filtrate solution was evaporated and the yield of **3g** was determined by ^{1}H-NMR using anisole as the internal standard.

ETMs employing ***5 mol% Co(salophen) and 10 mol% HQ***:

A microwave tube (10 mL) was charged with $Pd(OAc)_2$ (5 mol%), B_2pin_2 (1.3 equiv.) and **Co(salophen)** (5 mol%). Then, a solution of bisallene **1g** (0.1 mmol) and **HQ** (10 mol%) in acetone (1 mL) were injected by syringe, respectively. The reaction mixture was stirred at 25 °C under atmosphere of O_2. After a specific time, the reaction mixture was diluted with Et_2O (5 mL), and quickly filtered via a short column of silica gel (5 cm, eluent: 30 mL of Et_2O). The collected filtrate solution was evaporated and the yield of **3g** was determined by ^{1}H-NMR using anisole as the internal standard.

Results:

time	1h	3h	6h	10h	16h
5 mol% Co(salophen)-HQ	20%	60%	66%	67%	67%
5 mol% Co(salophen) and 10 mol% BQ	7%	19%	28%	35%	36%
5 mol% Co(salophen) and 10 mol% HQ	4%	10%	16%	26%	30%

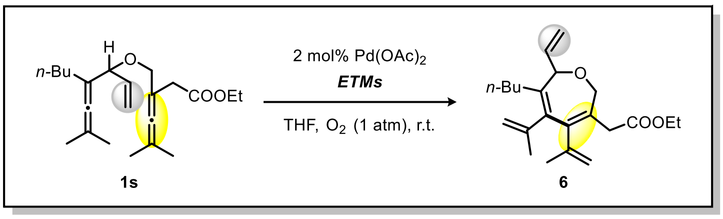

ETM employing ***5 mol% Co(salophen)-HQ***:

A microwave tube (10 mL) was charged with $Pd(OAc)_2$ (2 mol%) and **Co(salophen)-HQ** (5 mol%). Then, a solution of bisallene **1s** (0.1 mmol) in THF (1 mL) were injected by syringe, respectively. The reaction mixture was stirred at 25 °C under atmosphere of O_2. After a specific time, the reaction mixture was diluted with Et_2O (5 mL), and quickly filtered via a short column of silica gel (5 cm, eluent: 30 mL of Et_2O). The collected filtrate solution was evaporated and the yield of **3s** was determined by ^{1}H-NMR using anisole as the internal standard.

ETMs employing ***5 mol% Co(salophen) and 10 mol% BQ***:

A microwave tube (10 mL) was charged with $Pd(OAc)_2$ (2 mol%) and **Co(salophen)** (5 mol%). Then, a solution of bisallene **1s** (0.1 mmol) and **BQ** (10 mol%) in THF (1 mL) were injected by syringe, respectively. The reaction mixture was stirred at 25 °C under atmosphere of O_2. After a specific time, the reaction mixture was diluted with Et_2O (5 mL), and quickly filtered via a short column of silica gel (5 cm, eluent: 30 mL of Et_2O). The collected filtrate solution was evaporated and the yield of **3s** was determined by ^{1}H-NMR using anisole as the internal standard.

ETMs employing ***5 mol% Co(salophen) and 10 mol% HQ***:

A microwave tube (10 mL) was charged with $Pd(OAc)_2$ (2 mol%) and **Co(salophen)** (5 mol%). Then, a solution of bisallene **1s** (0.1 mmol) and **HQ** (10 mol%) in THF (1 mL) were injected by syringe, respectively. The reaction mixture was stirred at 25 °C under atmosphere of O_2. After a specific time, the reaction mixture was diluted with Et_2O (5 mL), and quickly filtered via a short column of silica gel (5 cm, eluent: 30 mL of Et_2O). The collected filtrate solution was evaporated and the yield of **3s** was determined by ^{1}H-NMR using anisole as the internal standard.

Results:

time	2h	5h	10h	16h	24h
5 mol% Co(salophen)-HQ	8%	15%	25%	35%	45%
5 mol% Co(salophen) and 10 mol% BQ	5%	9%	16%	19%	22%
5 mol% Co(salophen) and 10 mol% HQ	4%	7%	12%	15%	17%

5. Characterization of products 3a to 6

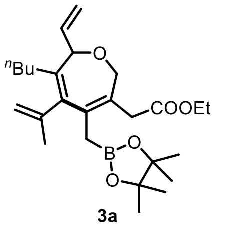

3a

Yield: 75%, colorless oil, R_f = 0.6 (petroleum ether/Et_2O = 5/1).

^1H NMR (400 MHz, $CDCl_3$) δ 6.11-6.02 (m, 1H), 5.27-5.17 (m, 2H), 5.09-5.08 (m, 1H), 4.78-4.76 (m, 1H), 4.49-4.47 (m, 1H), 4.14-4.08 (m, 2H), 3.97-3.88 (m, 2H), 3.35-3.26 (m, 2H), 2.40-2.34 (m, 1H), 2.07-2.01 (m, 1H), 1.87-1.77 (m, 2H), 1.76-1.75 (m, 3H), 1.48-1.26 (m, 4H), 1.23 (t, *J* = 7.1 Hz, 3H), 1.19 (s, 12H), 0.87 (t, *J* = 7.2 Hz, 3H);

^{13}C NMR (100 MHz, $CDCl_3$) δ 171.4, 145.5, 143.4, 141.0, 137.3, 137.1, 127.7, 116.4, 115.8, 83.2, 76.2, 66.7, 60.5, 38.7, 33.2, 30.6, 24.8, 24.7, 23.7, 23.2, 14.2, 13.9.

HRMS (ESI) $[C_{26}H_{41}BO_5+Na]^+$ calculated mass 467.2944, measured mass 467.2941.

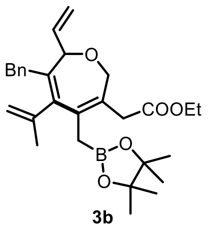

3b

Yield: 78%, yellow oil, R_f = 0.6 (petroleum ether/Et_2O = 5/1).

^1H NMR (400 MHz, $CDCl_3$) δ 7.23-7.14 (m, 5H), 5.86-5.77 (m, 1H), 5.15-5.11 (m, 1H), 5.05-5.04 (m, 1H), 4.97-4.94 (m, 1H), 4.88-4.87 (m, 1H), 4.61-4.59 (m, 1H), 4.16-4.10 (m, 2H), 3.98 (s, 2H), 3.76-3.62 (m, 2H), 3.37-3.29 (m, 2H), 1.96-1.84 (m, 2H), 1.73-1.72 (m, 3H), 1.25-1.21 (m, 15H);

^{13}C NMR (100 MHz, $CDCl_3$) δ 171.3, 147.8, 143.0, 141.2, 140.9, 136.8, 133.8, 128.7, 128.0, 127.9, 125.5, 116.2, 115.7, 83.3, 75.8, 66.6, 60.6, 38.6, 36.4, 24.8, 23.6, 14.2.

HRMS (ESI) $[C_{29}H_{39}BO_5+Na]^+$ calculated mass 501.2788, measured mass 501.2786.

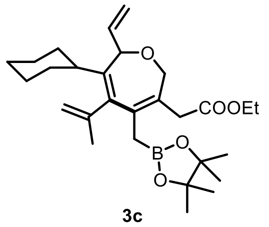

3c

Yield: 60%, colorless oil, R_f = 0.6 (petroleum ether/Et_2O = 5/1).

^1H NMR (400 MHz, $CDCl_3$) δ 6.05-5.97 (m, 1H), 5.04-4.98 (m, 2H), 4.88-4.85 (m, 1H), 4.73-4.72 (m, 1H), 4.65-4.63 (m, 1H), 4.13-4.07 (m, 2H), 4.00-3.85 (m, 2H), 3.25-3.17 (m, 2H), 2.64-2.61 (m, 1H), 1.76-1.75 (m, 3H), 1.68-1.49 (m, 6H), 1.24-1.18 (m, 21H);

^{13}C NMR (100 MHz, $CDCl_3$) δ 171.3, 144.5, 144.1, 142.4, 141.8, 140.4, 129.8, 115.1, 113.1, 83.1, 74.6, 66.1, 60.4, 42.9, 38.7, 32.2, 31.9, 26.5, 26.2, 26.0, 24.7, 23.8, 14.1, 14.0.

HRMS (ESI) $[C_{28}H_{43}BO_5+Na]^+$ calculated mass 493.3101, measured mass 493.3098.

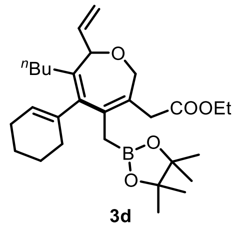

3d

Yield: 30%, yellow oil, R_f = 0.6 (petroleum ether/Et_2O = 5/1).

^1H NMR (400 MHz, $CDCl_3$) δ 6.11-6.02 (m, 1H), 5.47-5.46 (m, 1H), 5.25-5.15 (m, 2H), 4.48-4.47 (m, 1H), 4.14-4.08 (m, 2H), 3.95-3.87 (m, 2H), 3.35-3.25 (m, 2H), 2.38-2.32 (m, 1H), 2.06-2.01 (m, 3H), 1.91-1.89 (m, 2H), 1.85-1.75 (m, 2H), 1.67-1.64 (m, 4H), 1.35-1.25 (m, 4H), 1.23 (t, *J* = 7.1 Hz, 3H), 1.19 (s, 12H), 0.88 (t, *J* = 7.2 Hz, 3H);

^{13}C NMR (100 MHz, $CDCl_3$) δ 171.6, 146.3, 141.8, 137.3, 136.5, 127.4, 126.8, 116.2, 83.2, 76.3, 66.6, 60.5, 38.6, 33.2, 30.4, 29.5, 25.3, 24.8, 24.8, 23.1, 23.0, 22.2, 14.2, 13.9.

HRMS (ESI) $[C_{29}H_{45}BO_5+Na]^+$ calculated mass 507.3257, measured mass 507.3252.

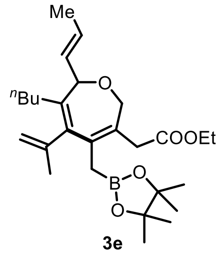

3e

Yield: 59%, yellow oil, R_f = 0.6 (petroleum ether/Et_2O = 5/1).

^1H NMR (400 MHz, $CDCl_3$) δ 5.81–5.62 (m, 2H), 5.10-5.09 (m, 1H), 4.77-4.77 (m, 1H), 4.45-4.43 (m, 1H), 4.17-4.09 (m, 2H), 3.95-3.88 (m, 2H), 3.38-3.27 (m, 2H), 2.41-2.34 (m, 1H), 2.10-2.03 (m, 1H), 1.88-1.82 (m, 2H), 1.77-1.76 (m, 3H), 1.75 (d, *J* = 5.1 Hz, 3H), 1.49-1.30 (m, 4H), 1.24 (t, *J* = 7.1 Hz, 3H), 1.21 (s, 12H), 0.90 (t, *J* = 7.2 Hz, 3H);

^{13}C NMR (100 MHz, $CDCl_3$) δ 171.4, 145.1, 143.5, 140.9, 137.7, 129.7, 128.6, 127.6, 115.6, 83.2, 75.9, 66.7, 60.5, 38.6, 33.3, 30.3, 24.8, 24.7, 23.7, 23.2, 17.9, 14.1, 13.9.

HRMS (ESI) $[C_{27}H_{43}BO_5+Na]^+$ calculated mass 481.3100, measured mass 481.3091.

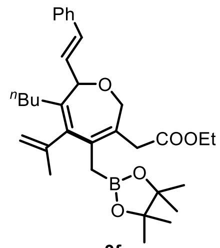

3f

Yield: 51% (10 mol% Cat.1), 65% (15 mol% Cat.2), yellow oil, R_f = 0.6 (petroleum ether/Et_2O = 5/1).

^1H NMR (400 MHz, $CDCl_3$) δ 7.41-7.39 (m, 2H), 7.35-7.31 (m, 2H), 7.26-7.22 (m, 1H), 6.65-6.60 (m, 1H), 6.47-6.41 (m, 1H), 5.14-5.13 (m, 1H), 4.81-4.80 (m, 1H), 4.72-4.70 (m, 1H), 4.18-4.12 (m, 2H), 4.04-3.97 (m, 2H), 3.40-3.31 (m, 2H), 2.48-2.41 (m, 1H), 2.16-2.09 (m, 1H), 1.90-1.82 (m, 2H), 1.81-1.80 (m, 3H), 1.55-1.30 (m, 4H), 1.28-1.24 (m, 15H), 0.88 (t, *J* = 7.3 Hz, 3H);

^{13}C NMR (100 MHz, $CDCl_3$) δ 171.4, 145.6, 143.3, 141.2, 137.5, 137.4, 131.6, 128.6, 128.5, 127.7, 127.3, 126.4, 115.8, 83.2, 75.7, 66.8, 60.6, 38.7, 33.2, 30.6, 24.8, 24.8, 23.7, 23.2, 14.2, 13.9.
HRMS (ESI) $[C_{32}H_{45}BO_5+Na]^+$ calculated mass 543.3258, measured mass 543.3251.

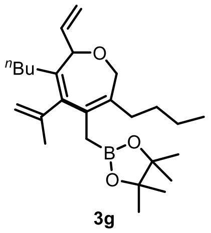

3g

Yield: 69%, yellow oil, R_f = 0.8 (petroleum ether/Et_2O = 5/1).
^{1}H NMR (400 MHz, $CDCl_3$) δ 6.15-6.06 (m, 1H), 5.28-5.18 (m, 2H), 5.09-5.08 (m, 1H), 4.76-4.75 (m, 1H), 4.50-4.48 (m, 1H), 3.90-3.82 (m, 2H), 2.42-2.05 (m, 4H), 1.88-1.84 (m, 2H), 1.77 -1.76 (m, 3H), 1.50-1.39 (m, 8H), 1.21 (s, 12H), 0.95-0.88 (m, 6H);
^{13}C NMR (100 MHz, $CDCl_3$) δ 146.0, 143.8, 137.4, 136.6, 136.2, 135.7, 116.3, 115.3, 83.0, 76.4, 66.5, 33.3, 33.2, 31.3, 30.5, 24.8, 24.7, 23.8, 23.2, 22.8, 14.1, 13.9.
HRMS (ESI) $[C_{26}H_{43}BO_3+Na]^+$ calculated mass 437.3202, measured mass 437.3194.

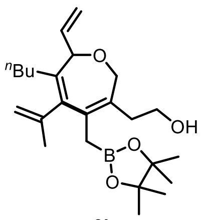

3h

Yield: 52%, colorless oil, R_f = 0.5 (petroleum ether/Et_2O = 2/1).
^{1}H NMR (400 MHz, $CDCl_3$) δ 6.09-6.01 (m, 1H), 5.27-5.16 (m, 2H), 5.09-5.08 (m, 1H), 4.73 (s, 1H), 4.44 (d, *J* = 7.0 Hz, 1H), 3.87 (s, 2H), 3.81-3.75 (m, 2H), 2.85 (br, 1H), 2.67-2.61 (m, 1H), 2.42-2.30 (m, 2H), 2.08-2.01 (m, 1H), 1.92-1.78 (m, 2H), 1.77-1.76 (m, 3H), 1.31-1.25 (m, 4H), 1.20 (s, 12H), 0.87 (t, *J* = 7.1 Hz, 3H);
^{13}C NMR (100 MHz, $CDCl_3$) δ 145.8, 143.4, 139.5, 137.0, 136.8, 132.2, 116.8, 115.7, 83.6, 76.6, 66.5, 62.0, 36.8, 33.2, 30.7, 24.7, 24.6, 23.8, 23.2, 13.9.
HRMS (ESI) $[C_{24}H_{39}BO_4+Na]^+$ calculated mass 425.2838, measured mass 425.2837.

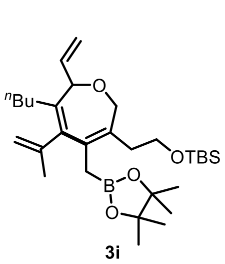

3i

Yield: 66%, colorless oil, R_f = 0.8 (petroleum ether/Et_2O = 6/1).
^{1}H NMR (400 MHz, $CDCl_3$) δ 6.11-6.02 (m, 1H), 5.25-5.15 (m, 2H), 5.07-5.06 (m, 1H), 4.73-4.72 (m, 1H), 4.42-4.40 (m, 1H), 3.90-3.79 (m, 2H), 3.73-3.66 (m, 2H), 2.52-2.46 (m, 2H), 2.36-2.31 (m, 1H), 2.06-1.99 (m, 1H), 1.89-1.73 (m, 2H), 1.74-1.73 (m, 3H), 1.46-1.27 (m, 4H), 1.18 (d, *J* = 2.8 Hz, 12H), 0.88 (s, 9H), 0.04 (s, 6H);

^{13}C NMR (100 MHz, $CDCl_3$) δ 145.9, 143.6, 138.9, 137.2, 136.6, 131.9, 116.4, 115.5, 83.1, 76.3, 66.9, 62.3, 37.2, 33.3, 30.6, 25.9, 24.8, 24.7, 23.8, 23.2, 18.3, 13.9, -5.3.
HRMS (ESI) $[C_{30}H_{53}BSiO_4+Na]^+$ calculated mass 539.3704, measured mass 539.3704.

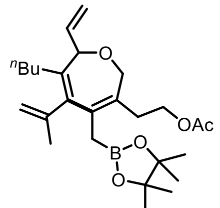

3j

Yield: 72%, yellow oil, R_f = 0.6 (petroleum ether/Et_2O = 5/1).
^1H NMR (400 MHz, $CDCl_3$) δ 6.09-6.07 (m, 1H), 5.25-5.15 (m, 2H), 5.08-5.07 (m, 1H), 4.73-4.72 (m, 1H), 4.41 (d, *J* = 7.0 Hz, 1H), 4.21-4.13 (m, 2H), 3.88-3.80 (m, 2H), 2.60-2.54 (m, 2H), 2.36-2.31 (m, 1H), 2.02-1.99 (m, 4H), 1.88-1.74 (m, 2H), 1.73-1.72 (m, 3H), 1.46-1.27 (m, 4H), 1.19-1.18 (m, 12H), 0.86 (t, *J* = 7.1 Hz, 3H);
^{13}C NMR (100 MHz, $CDCl_3$) δ 171.0, 145.6, 143.4, 140.0, 137.1, 137.0, 130.5, 116.4, 115.7, 83.2, 76.2, 66.5, 63.2, 33.2, 32.6, 30.6, 24.8, 24.7, 23.7, 23.2, 21.0, 13.9.
HRMS (ESI) $[C_{26}H_{41}BO_5+Na]^+$ calculated mass 467.2944, measured mass 467.2951.

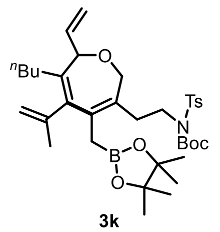

3k

Yield: 70%, yellow oil, R_f = 0.5 (petroleum ether/Et_2O = 5/1).
^1H NMR (400 MHz, $CDCl_3$) δ 7.79 (d, *J* = 8.2 Hz, 2H), 7.28 (d, *J* = 8.2 Hz, 2H), 6.12-6.03 (m, 1H), 5.28-5.17 (m, 2H), 5.08-5.07 (m, 1H), 4.73-4.72 (m, 1H), 4.46 (d, *J* = 6.9 Hz, 1H), 3.98-3.84 (m, 4H), 2.73-2.67 (m, 2H), 2.42 (s, 3H), 2.37-2.31 (m, 1H), 2.06-2.03 (m, 1H), 1.99-1.80 (m, 2H), 1.77-1.75 (m, 3H), 1.47-1.27 (m, 13H), 1.19 (d, *J* = 5.1 Hz, 12H), 0.87 (t, *J* = 7.1 Hz, 3H);
^{13}C NMR (100 MHz, $CDCl_3$) δ 150.8, 145.7, 143.9, 143.5, 140.2, 137.5, 137.2, 137.0, 130.9, 129.1, 127.8, 116.4, 115.6, 83.9, 83.2, 76.3, 66.6, 45.8, 34.5, 33.2, 30.6, 27.9, 24.9, 24.7, 23.8, 23.2, 21.5, 13.9.
HRMS (ESI) $[C_{36}H_{54}BNSO_7+Na]^+$ calculated mass 678.3612, measured mass 678.3606.

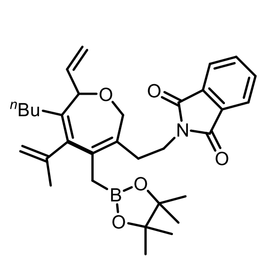

3l

Yield: 75%, yellow oil, R_f = 0.5 (petroleum ether/Et_2O = 5/1).
^1H NMR (400 MHz, $CDCl_3$) δ 7.82-7.80 (m, 2H), 7.70-7.68 (m, 2H), 6.09-6.01 (m, 1H), 5.25-5.15 (m, 2H), 5.02-5.01 (m, 1H), 4.69-4.68 (m, 1H), 4.45 (d, *J* = 6.8 Hz, 1H), 3.99-3.82 (m, 4H), 2.74-2.62 (m,

2H), 2.38-2.31 (m, 1H), 2.04-1.98 (m, 1H), 1.86-1.67 (m, 2H), 1.64-1.63 (m, 3H), 1.47-1.24 (m, 4H), 1.20 (d, J = 2.1 Hz, 12H), 0.88 (t, J = 7.2 Hz, 3H);

^{13}C NMR (100 MHz, $CDCl_3$) δ 168.1, 145.4, 143.4, 140.2, 137.0, 137.0, 133.7, 132.2, 130.9, 123.1, 116.3, 115.6, 83.2, 76.1, 66.1, 36.9, 33.2, 31.7, 30.5, 24.8, 24.7, 23.6, 23.2, 13.8.

HRMS (ESI) $[C_{32}H_{42}BNO_5+Na]^+$ calculated mass 554.3054, measured mass 554.3059.

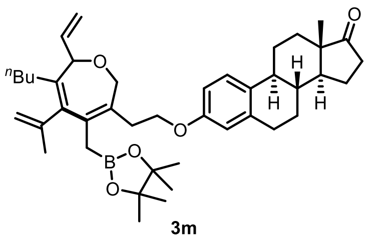

3m

Yield: 78%, oil, R_f = 0.6 (petroleum ether/Et_2O = 5/1).

^{1}H NMR (400 MHz, $CDCl_3$) δ 7.16 (d, J = 8.6 Hz, 1H), 6.71 (dd, J = 8.6, 2.7 Hz, 1H), 6.64-6.63 (m, 1H), 6.12-6.03 (m, 1H), 5.26-5.16 (m, 2H), 5.09-5.08 (m, 1H), 4.76-4.75 (m, 1H), 4.45 (d, J = 6.9 Hz, 1H), 4.07-4.04 (m, 2H), 3.96-3.85 (m, 2H), 2.88-2.86 (m,2H), 2.80-2.65 (m, 2H), 2.53-2.46 (m, 1H), 2.40-2.34 (m, 2H), 2.27-2.22 (m, 1H), 2.18-1.94 (m, 4H), 1.94-1.85 (m, 2H), 1.76-1.75 (m, 3H), 1.62-1.43 (m, 7H), 1.32-1.29 (m, 4H), 1.19 (d, J = 2.2 Hz, 12H), 0.91-0.88 (m, 6H);

^{13}C NMR (100 MHz, $CDCl_3$) δ 220.9, 156.9, 145.8, 143.5, 139.8, 137.6, 137.1, 136.9, 131.8, 130.9, 126.2, 116.5, 115.6, 114.7, 114.7, 112.2, 112.2, 83.2, 76.4, 66.7, 66.7, 66.7, 50.4, 48.0, 44.0, 38.3, 35.8, 33.5, 33.2, 31.6, 30.6, 29.6, 26.5, 25.9, 24.8, 24.7, 23.8, 23.2, 21.5, 13.9, 13.8.

HRMS (ESI) $[C_{42}H_{59}BO_5+Na]^+$ calculated mass 677.4355, measured mass 677.4348.

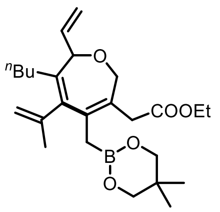

3n

Yield: 40%, yellow oil, R_f = 0.6 (petroleum ether/Et_2O = 5/1).

^{1}H NMR (400 MHz, $CDCl_3$) δ 6.10-6.01 (m, 1H), 5.23-5.18 (m, 2H), 5.09-5.08 (m, 1H), 4.75-4.74 (m, 1H), 4.47 (d, J = 6.8 Hz, 1H), 4.13-4.08 (m, 2H), 3.97-3.88 (m, 2H), 3.51 (s, 4H), 3.32-3.30 (m, 2H), 2.38-2.34 (m, 1H), 2.07-2.03 (m, 1H), 1.83-1.81 (m, 2H), 1.77-1.75 (m, 3H), 1.30-1.25 (m, 4H), 1.22 (t, J = 7.1 Hz, 3H), 0.91 (s, 6H), 0.87 (t, J = 7.1 Hz, 3H);

^{13}C NMR (100 MHz, $CDCl_3$) δ 171.6, 145.9, 143.7, 141.9, 137.2, 136.9, 127.4, 116.3, 115.5, 76.3, 72.0, 66.7, 60.5, 38.7, 33.2, 31.6, 30.6, 23.7, 23.2, 21.8, 14.2, 13.9.

HRMS (ESI) $[C_{25}H_{39}BO_5+Na]^+$ calculated mass 453.2787, measured mass 453.2796.

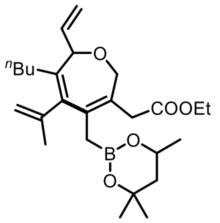

3o

Yield: 52%, yellow oil, R_f = 0.6 (petroleum ether/Et_2O = 5/1).

^{1}H NMR (400 MHz, $CDCl_3$) δ 6.10-6.02 (m, 1H), 5.28-5.27 (m, 2H), 5.07-5.06 (m, 1H), 4.75-4.73 (m, 1H), 4.49-4.46 (m, 1H), 4.13-4.08 (m, 3H), 3.96-3.89 (m, 2H), 3.36-3.26 (m, 2H), 2.40-2.33 (m, 1H), 2.07-2.00 (m, 1H), 1.75-1.74 (m, 3H), 1.72-1.69 (m, 2H), 1.42-1.27 (m, 4H), 1.22-1.20 (m, 8H), 1.17 (d, *J* = 6.2 Hz, 3H), 0.87 (t, *J* = 7.2 Hz, 3H);

^{13}C NMR (100 MHz, $CDCl_3$) δ 171.7, 146.1, 146.1, 143.8, 142.4, 142.3, 137.2, 136.6, 127.1, 115.9, 115.9, 115.5, 115.4, 76.0, 70.7, 70.7, 66.6, 66.6, 64.7, 60.4, 45.8, 45.8, 38.6, 38.6, 33.2, 31.2, 30.7, 28.0, 28.0, 23.7, 23.7, 23.2, 23.1, 14.2, 13.9.

HRMS (ESI) $[C_{26}H_{41}BO_5+Na]^+$ calculated mass 467.2944, measured mass 467.2939.

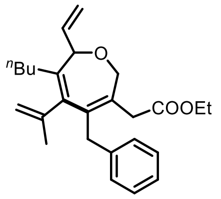

4

Yield: 48%, yellow oil, R_f = 0.8 (petroleum ether/Et_2O = 10/1).

^{1}H NMR (400 MHz, $CDCl_3$) δ 7.24-7.21 (m, 2H), 7.17-7.13 (m, 3H), 6.00-5.91 (m, 1H), 5.17-5.16 (m, 1H), 5.14-5.12 (m, 2H), 4.69-4.68 (m, 1H), 4.34-4.32 (m, 1H), 4.14-4.09 (m, 2H), 4.01-3.92 (m, 2H), 3.65-3.62 (m, 1H), 3.50-3.39 (m, 3H), 2.44-2.37 (m, 1H), 2.03-1.96 (m, 1H), 1.77-1.76 (m, 3H), 1.46-1.28 (m, 4H), 1.22 (t, *J* = 7.1 Hz, 3H), 0.87 (t, *J* = 7.1 Hz, 3H);

^{13}C NMR (100 MHz, $CDCl_3$) δ 171.1, 144.9, 142.9, 142.6, 139.5, 139.1, 136.4, 130.9, 128.4, 128.2, 126.0, 116.8, 116.4, 66.6, 60.8, 38.7, 35.4, 33.2, 30.6, 23.7, 23.2, 14.1, 13.9.

HRMS (ESI) $[C_{26}H_{34}O_3+Na]^+$ calculated mass 417.2400, measured mass 417.2391.

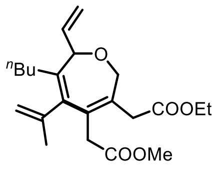

5

Yield: 78%, yellow oil, R_f = 0.75 (petroleum ether/Et_2O = 5/1).

^{1}H NMR (400 MHz, $CDCl_3$) δ 6.08-5.97 (m, 1H), 5.28-5.16 (m, 2H), 5.13-5.12 (m, 1H), 4.77-4.76 (m, 1H), 4.45 (d, *J* = 6.4 Hz, 1H), 4.12 (qd, *J* = 7.1, 0.7 Hz, 2H), 4.01-3.88 (m, 2H), 3.62 (s, 3H), 3.36-3.35 (m, 2H), 3.29-3.17 (m, 2H), 2.41-2.34 (m, 1H), 2.09-2.04 (m, 1H), 1.76-1.75 (m, 3H), 1.50-1.28 (m, 4H), 1.23 (t, *J* = 7.1 Hz, 3H), 0.87 (t, *J* = 7.1 Hz, 3H);

^{13}C NMR (100 MHz, $CDCl_3$) δ 170.9, 170.7, 144.0, 142.7, 139.6, 136.6, 136.6, 133.4, 116.6, 116.5, 76.5, 66.5, 60.9, 51.7, 38.7, 35.0, 33.1, 31.0, 23.6, 23.1, 14.1, 13.9.

HRMS (ESI) $[C_{22}H_{32}O_5+Na]^+$ calculated mass 399.2142, measured mass 399.2138.

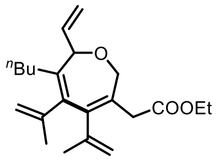

6

Yield: 79%, yellow oil, R_f = 0.8 (petroleum ether/Et_2O = 10/1).

^1H NMR (400 MHz, $CDCl_3$) δ 6.06-5.98 (m, 1H), 5.27-5.18 (m, 2H), 5.08-5.03 (m, 2H), 4.76-4.71 (m, 2H), 4.45 (d, *J* = 6.2 Hz, 1H), 4.15-4.09 (m, 2H), 4.03-3.89 (m, 2H), 3.42-3.31 (m, 2H), 2.42-2.37 (m, 1H), 2.14-2.09 (m, 1H), 1.76-1.74 (m, 6H), 1.49-1.31 (m, 4H), 1.23 (t, *J* = 7.1 Hz, 3H), 0.88 (t, *J* = 7.1 Hz, 3H);

^{13}C NMR (100 MHz, $CDCl_3$) δ ^{13}C NMR (101 MHz, $CDCl_3$) δ 171.8, 147.5, 143.7, 143.2, 142.5, 139.8, 137.1, 129.2, 116.2, 116.0, 115.2, 76.6, 65.9, 60.7, 39.1, 33.0, 31.3, 23.4, 23.1, 22.9, 14.1, 13.9.

HRMS (ESI) $[C_{22}H_{32}O_3+Na]^+$ calculated mass 367.2244, measured mass 367.2243.

6. Spectra of catalysts, starting materials and products

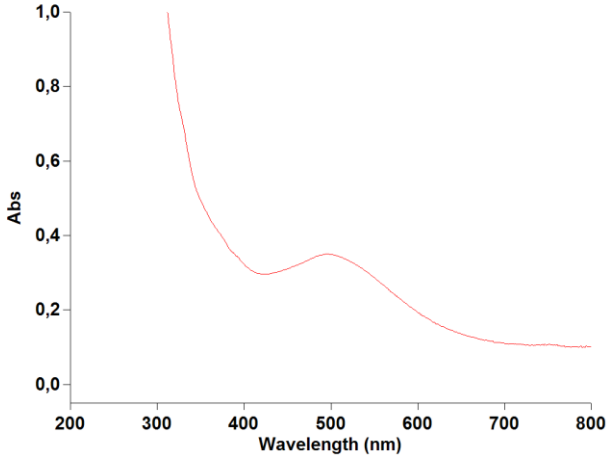

UV-visible spectrum of **Cat. 1** in MeOH

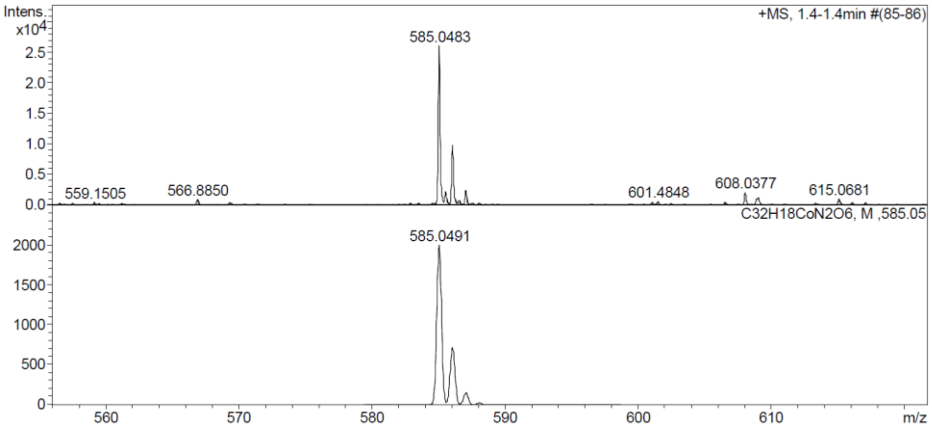

HRMS of **Cat. 1** in MeOH

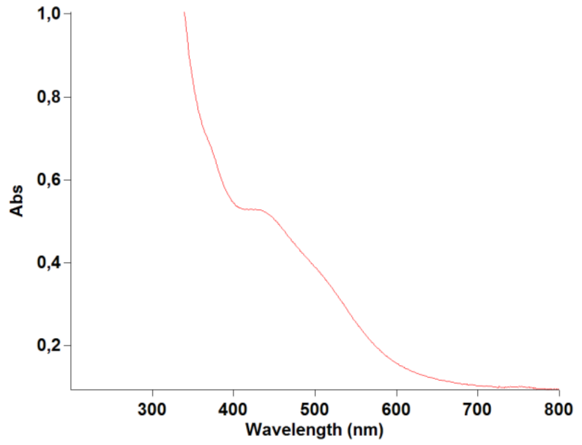

UV-visible spectrum of **Cat. 2** in MeOH

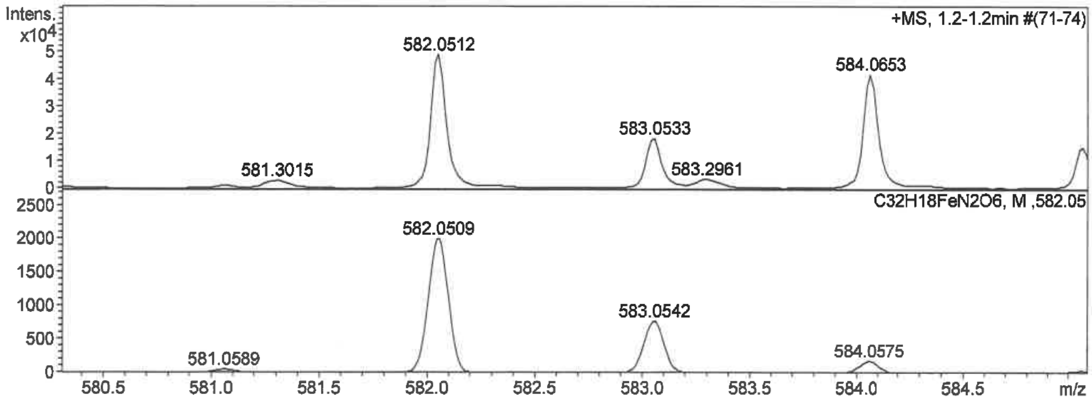

HRMS of **Cat. 2** in MeOH

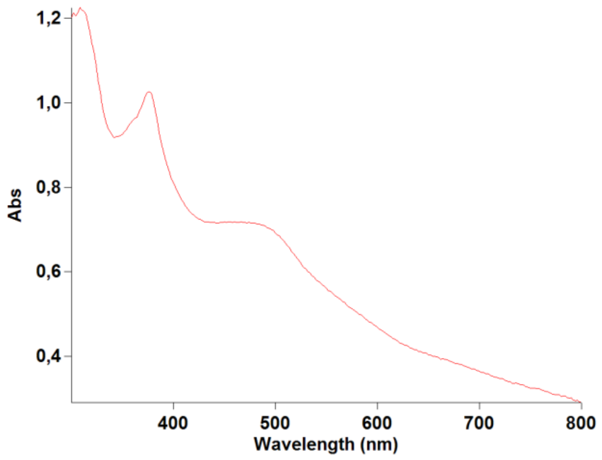

UV-visible spectrum of **Cat. 3** in MeOH

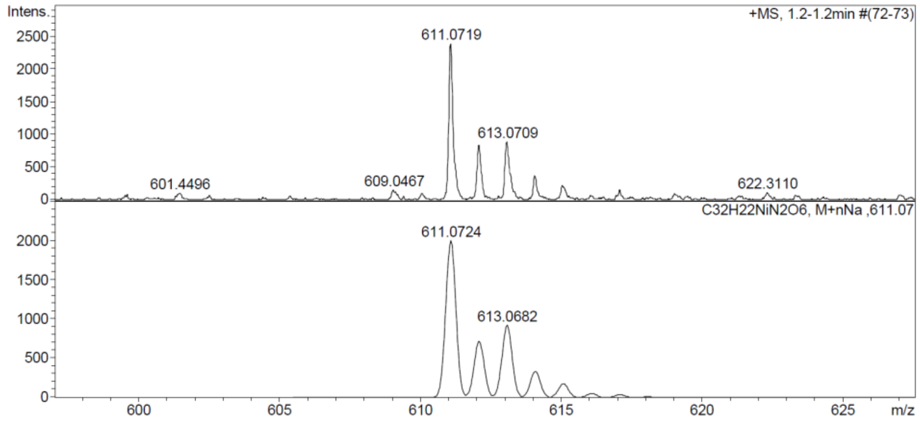

HRMS of **Cat. 3** in MeOH

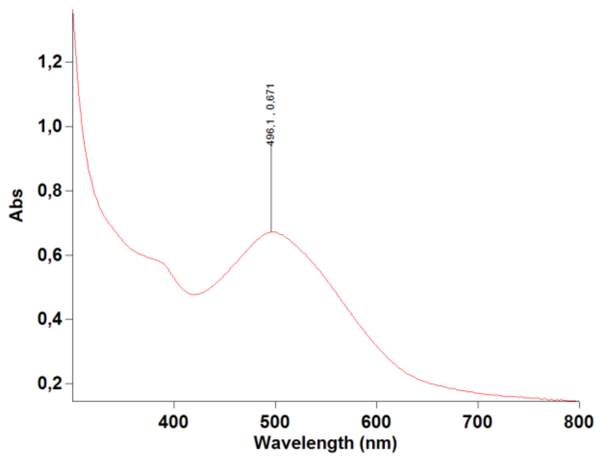

UV-visible spectrum of **Cat. 4** in MeOH

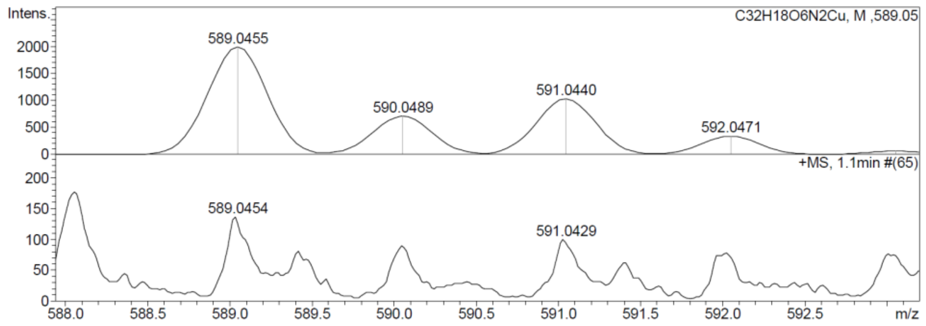

HRMS of **Cat. 4** in MeOH

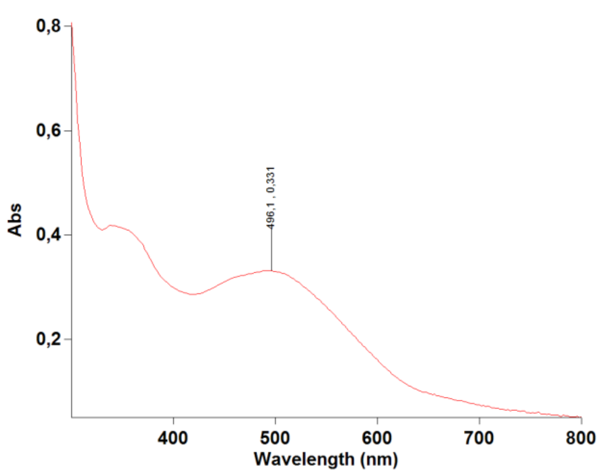

UV-visible spectrum of **Cat. 5** in MeOH

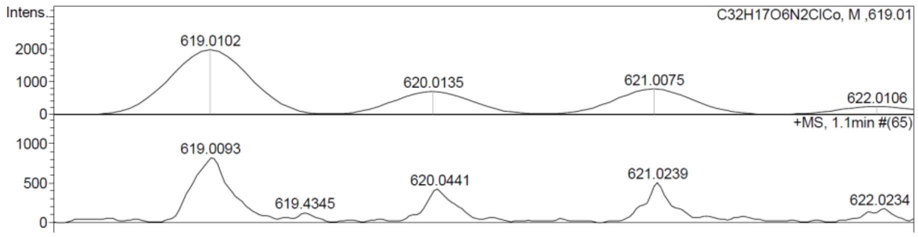

HRMS of **Cat. 5** in MeOH

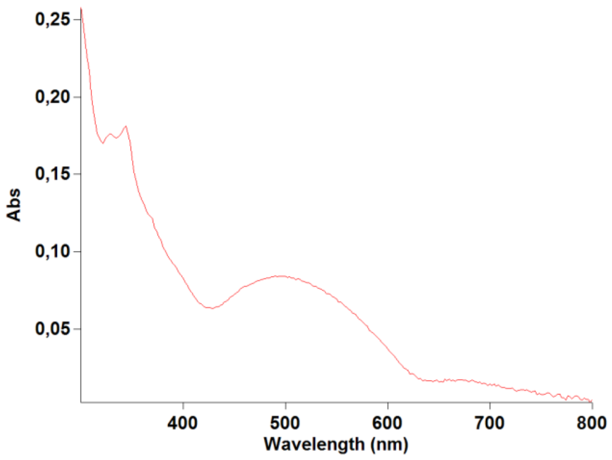

UV-visible spectrum of **Cat. 6** in MeOH

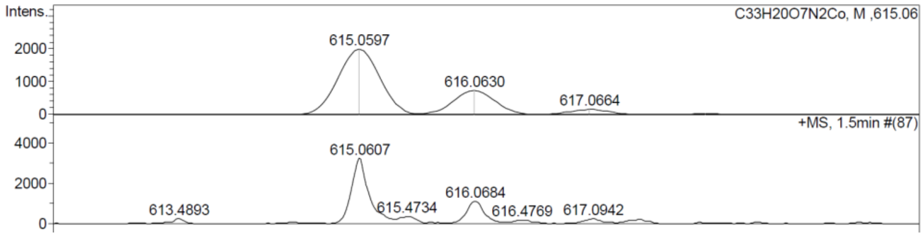

HRMS of **Cat. 6** in MeOH

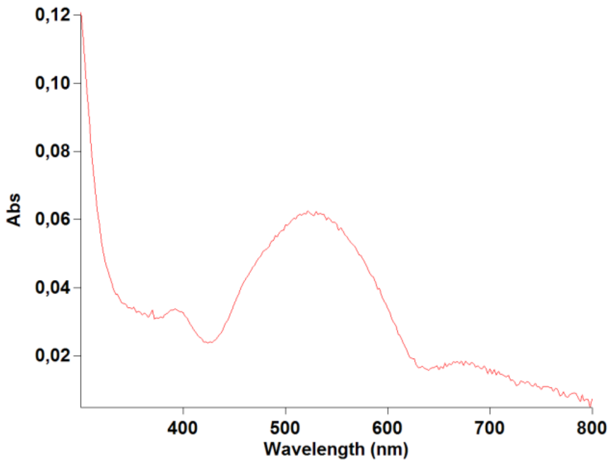

UV-visible spectrum of **Cat. 7** in MeOH

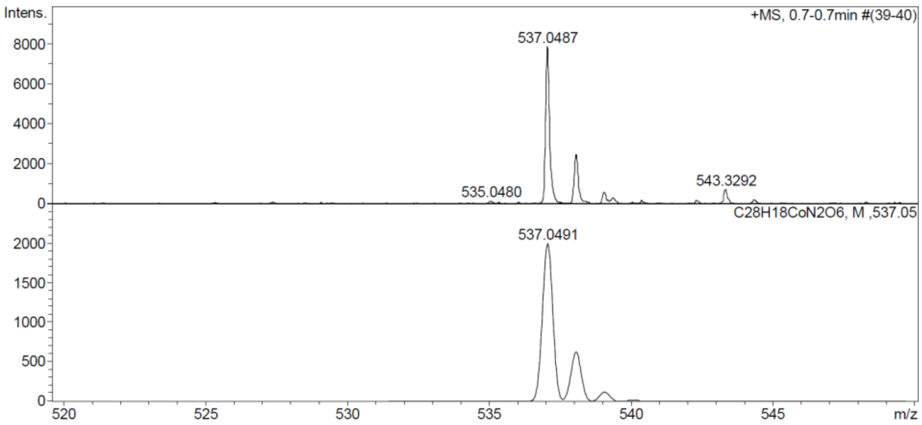

HRMS of **Cat. 7** in MeOH

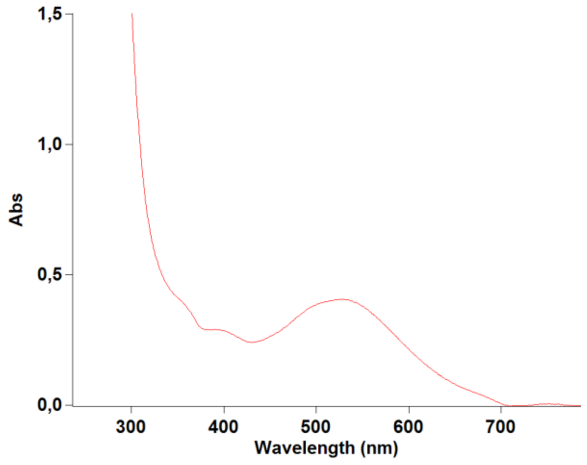

UV-visible spectrum of **Cat. 8** in MeOH

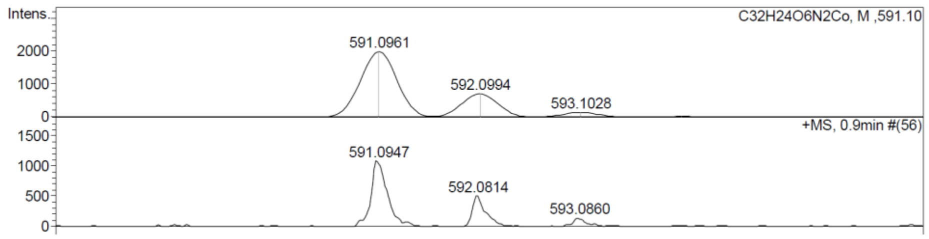

HRMS of **Cat. 8** in MeOH

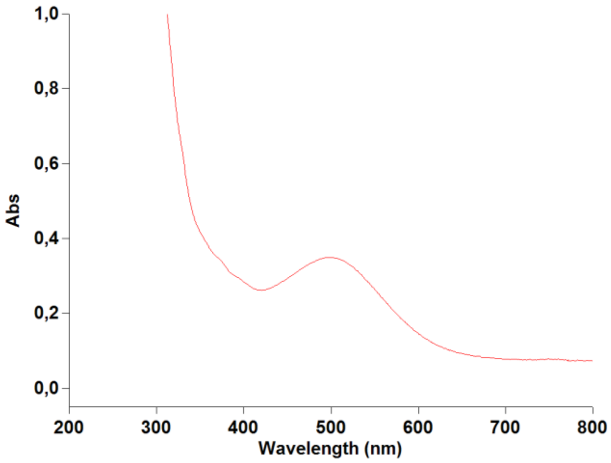

UV-visible spectrum of **Cat. 9** in MeOH

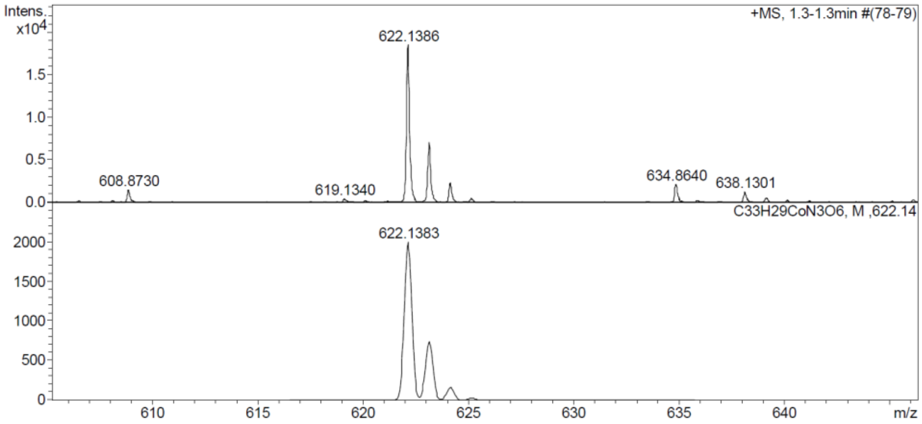

HRMS of **Cat. 9** in MeOH

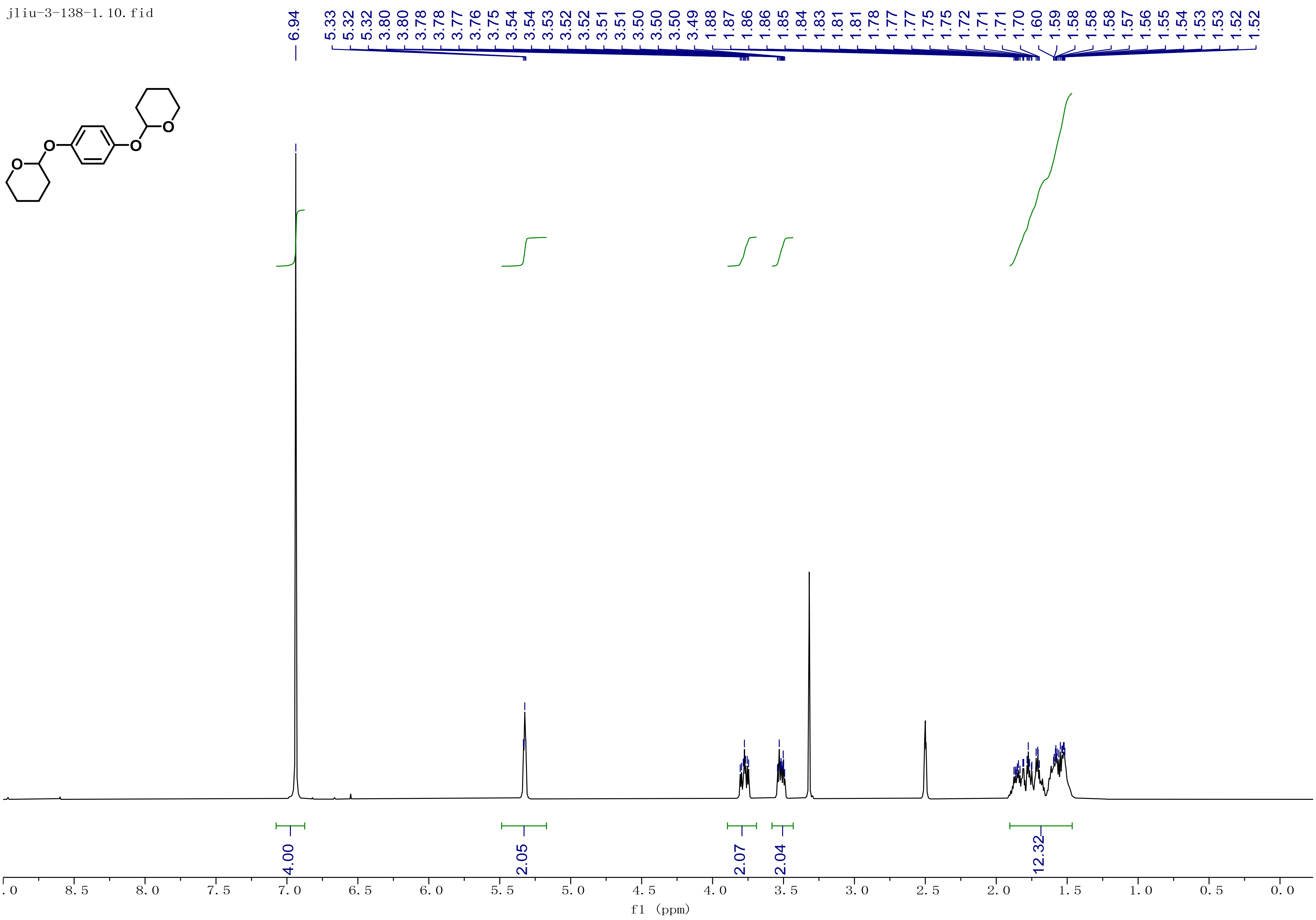

jliu-3-138-1.10.fid
6.94
5.33 5.32 5.32 3.80 3.80 3.78 3.78 3.77 3.76 3.75 3.54 3.54 3.53 3.52 3.52 3.51 3.51 3.50 3.50 3.50 3.49 1.88 1.87 1.86 1.86 1.85 1.84 1.83 1.81 1.81 1.78 1.77 1.77 1.75 1.75 1.72 1.71 1.71 1.70 1.60 1.59 1.58 1.58 1.58 1.57 1.56 1.55 1.54 1.53 1.53 1.52 1.52
4.00
2.05
2.07
2.04
12.32
f1 (ppm)

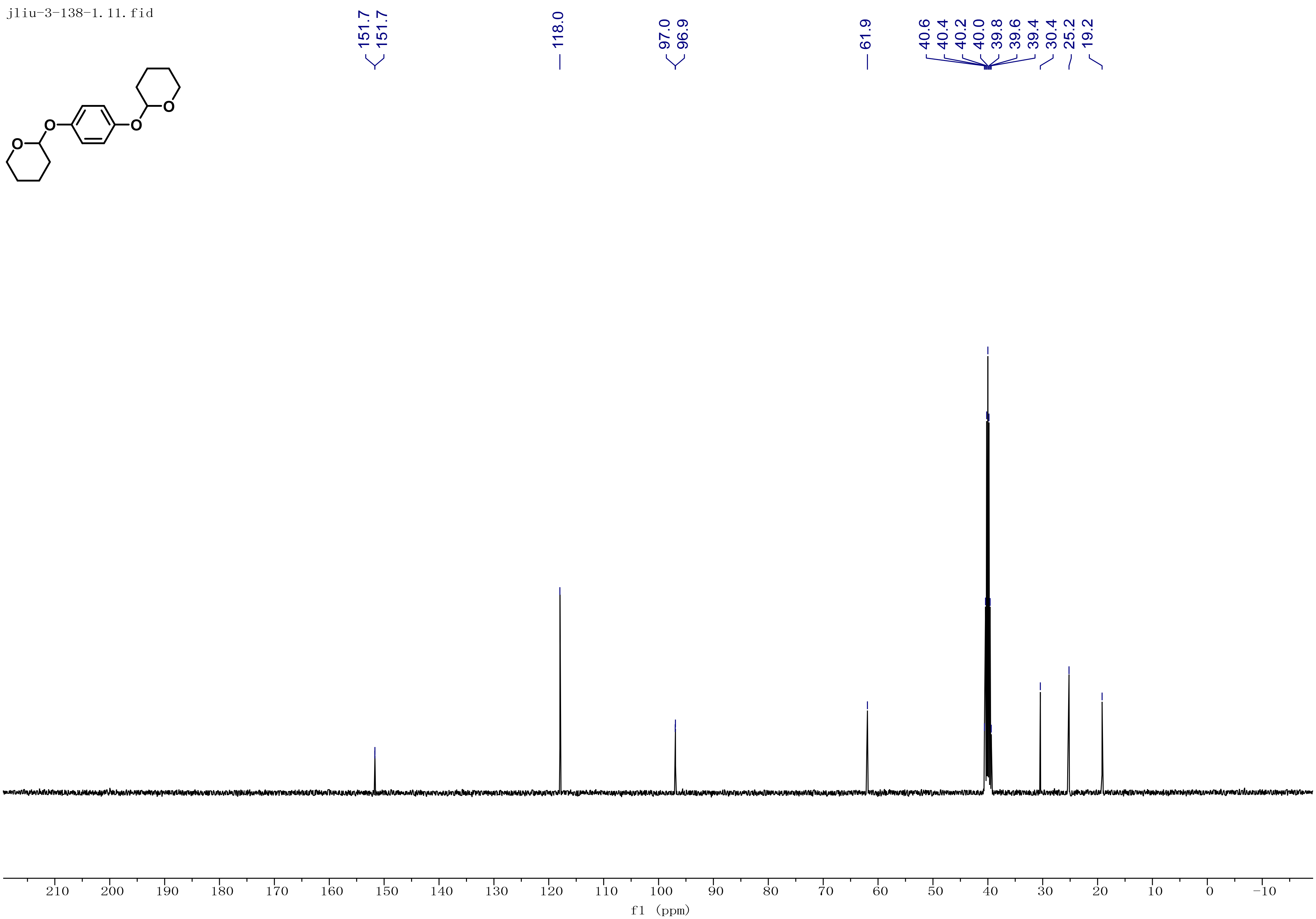
jliu-3-138-1.11.fid
151.7
151.7
118.0
97.0
96.9
61.9
40.6
40.4
40.2
40.0
39.8
39.6
39.4
30.4
25.2
19.2
210 200 190 180 170 160 150 140 130 120 110 100 90 80 70 60 50 40 30 20 10 0 −10
f1 (ppm)

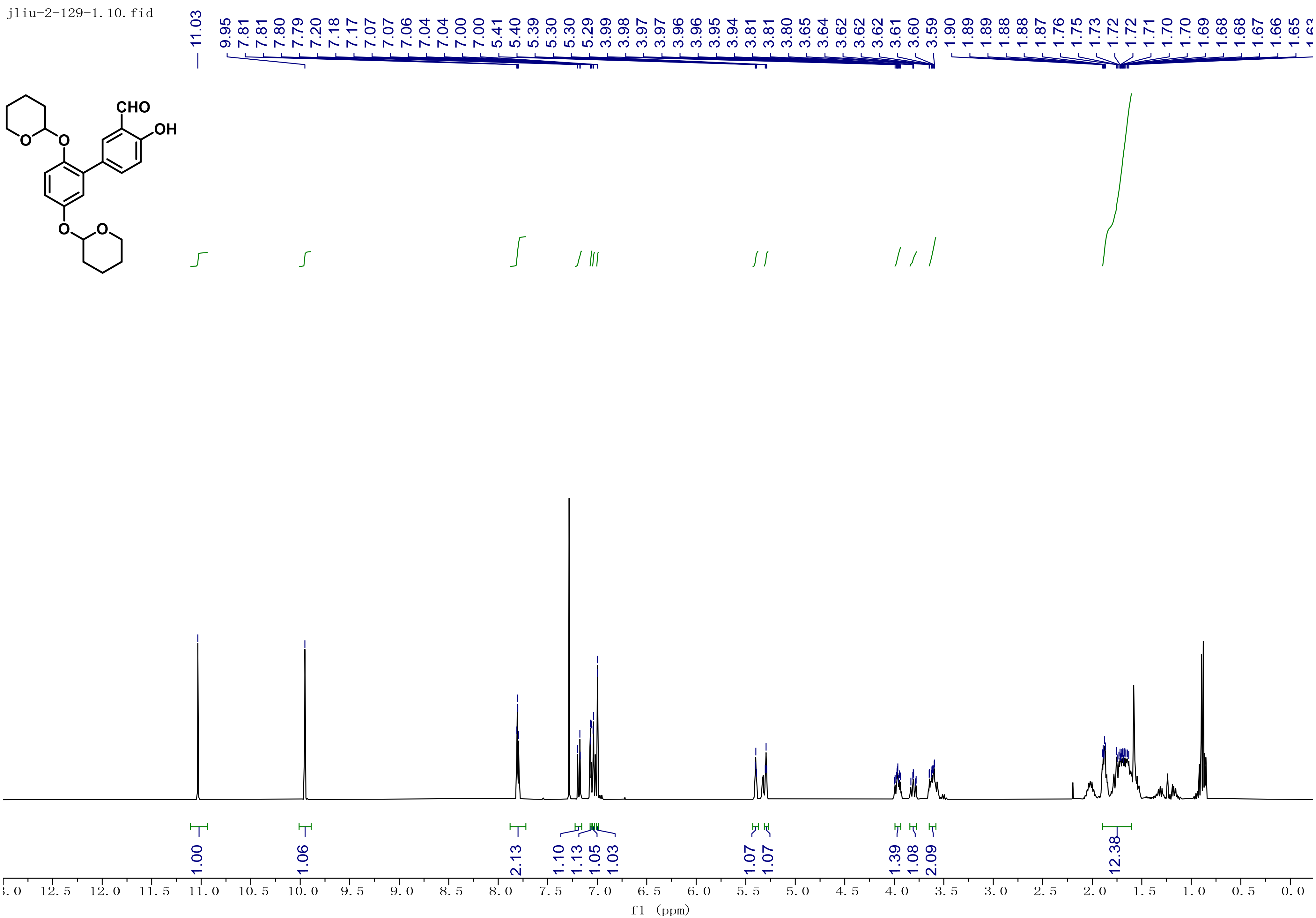
jliu-2-129-1.10.fid
CHO
OH
11.03
9.95 7.81 7.81 7.80 7.79 7.20 7.18 7.17 7.07 7.07 7.06 7.04 7.04 7.00 7.00 5.41 5.40 5.39 5.30 5.30 5.29 3.99 3.98 3.97 3.97 3.96 3.96 3.95 3.94 3.81 3.81 3.80 3.65 3.64 3.62 3.62 3.62 3.61 3.60 3.59
1.90 1.89 1.89 1.88 1.88 1.87 1.76 1.75 1.73 1.72 1.72 1.71 1.70 1.70 1.69 1.68 1.68 1.67 1.66 1.65
1.00
1.06
2.13
1.10 1.13 1.05 1.03
1.07 1.07
1.39 1.08 2.09
12.38
12.5 12.0 11.5 11.0 10.5 10.0 9.5 9.0 8.5 8.0 7.5 7.0 6.5 6.0 5.5 5.0 4.5 4.0 3.5 3.0 2.5 2.0 1.5 1.0 0.5 0.0
f1 (ppm)

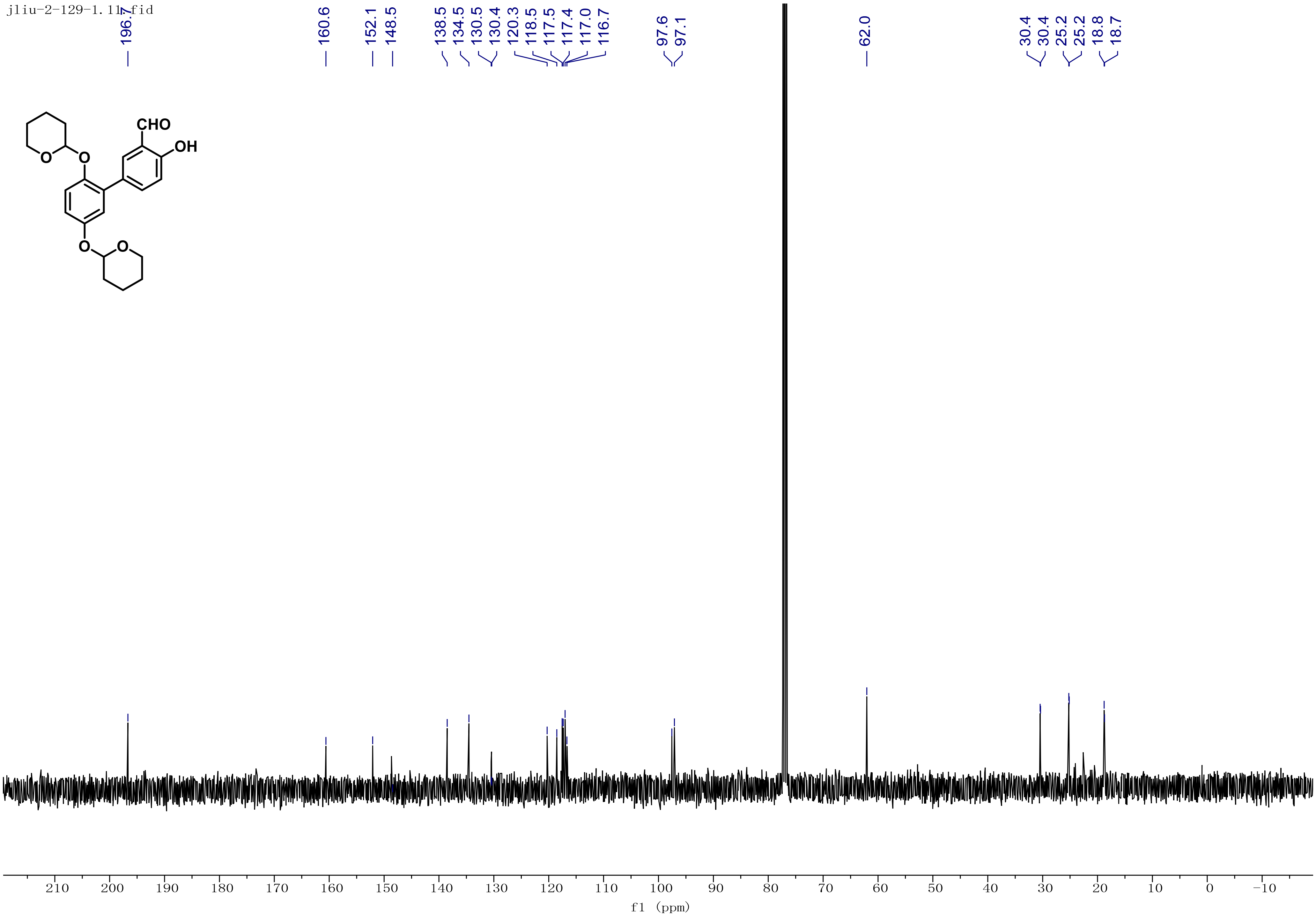
jliu-2-129-1.11.fid
196.7
160.6
152.1
148.5
138.5
134.5
130.5
130.4
120.3
118.5
117.5
117.4
117.0
116.7
97.6
97.1
62.0
30.4
30.4
25.2
25.2
18.8
18.7
CHO
OH
O
O
O
O
210 200 190 180 170 160 150 140 130 120 110 100 90 80 70 60 50 40 30 20 10 0 -10
f1 (ppm)

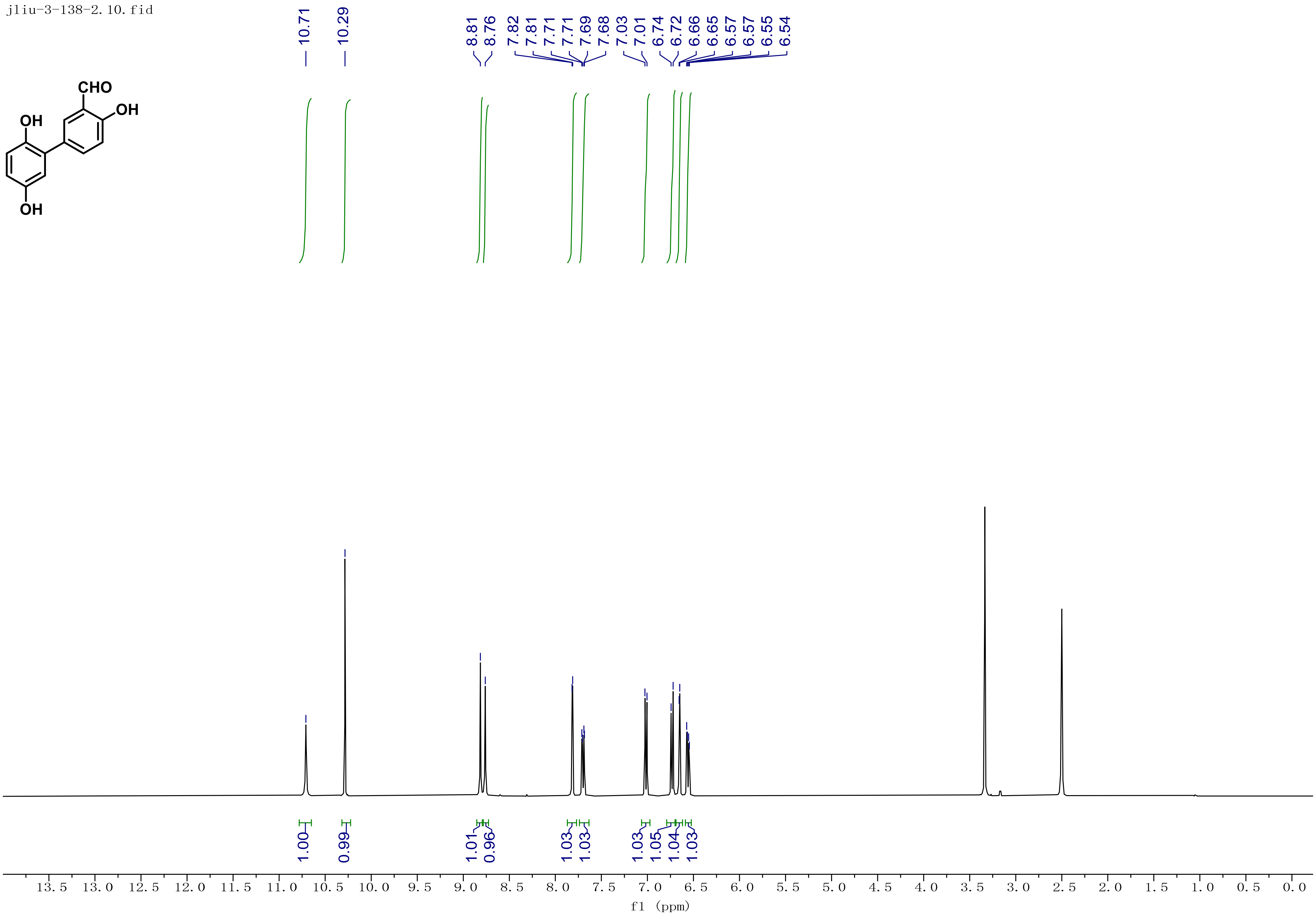
jliu-3-138-2.10.fid
CHO
OH
OH
OH
10.71
10.29
8.81
8.76
7.82
7.81
7.71
7.71
7.69
7.68
7.03
7.01
6.74
6.72
6.66
6.65
6.57
6.57
6.55
6.54
1.00
0.99
1.01
0.96
1.03
1.03
1.03
1.05
1.04
1.03
13.5 13.0 12.5 12.0 11.5 11.0 10.5 10.0 9.5 9.0 8.5 8.0 7.5 7.0 6.5 6.0 5.5 5.0 4.5 4.0 3.5 3.0 2.5 2.0 1.5 1.0 0.5 0.0
f1 (ppm)

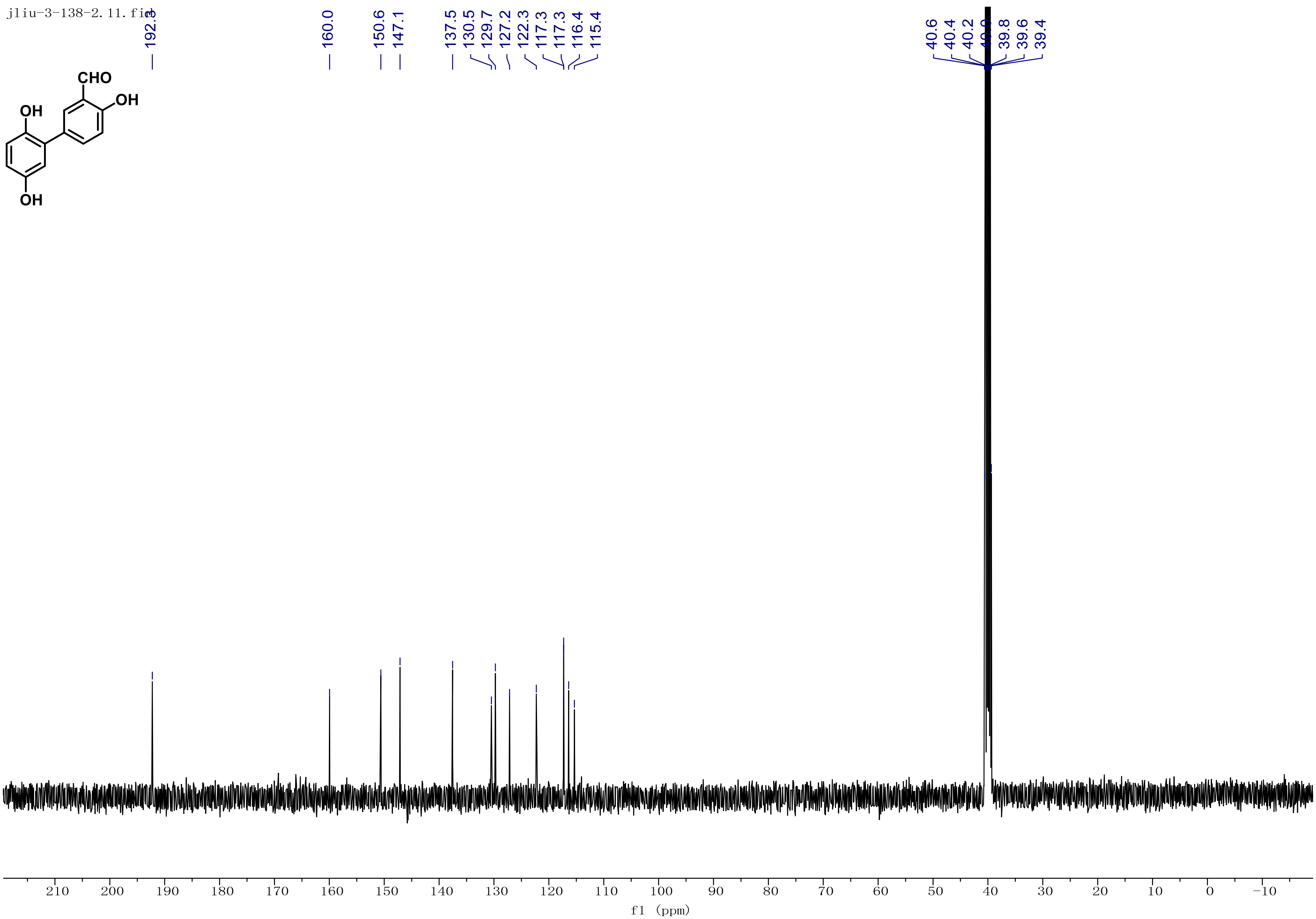
jliu-3-138-2.11.fid
192.3
160.0
150.6
147.1
137.5
130.5
129.7
127.2
122.3
117.3
117.3
116.4
115.4
40.6
40.4
40.2
40.0
39.8
39.6
39.4
CHO
OH
OH
OH
210 200 190 180 170 160 150 140 130 120 110 100 90 80 70 60 50 40 30 20 10 0 -10
f1 (ppm)

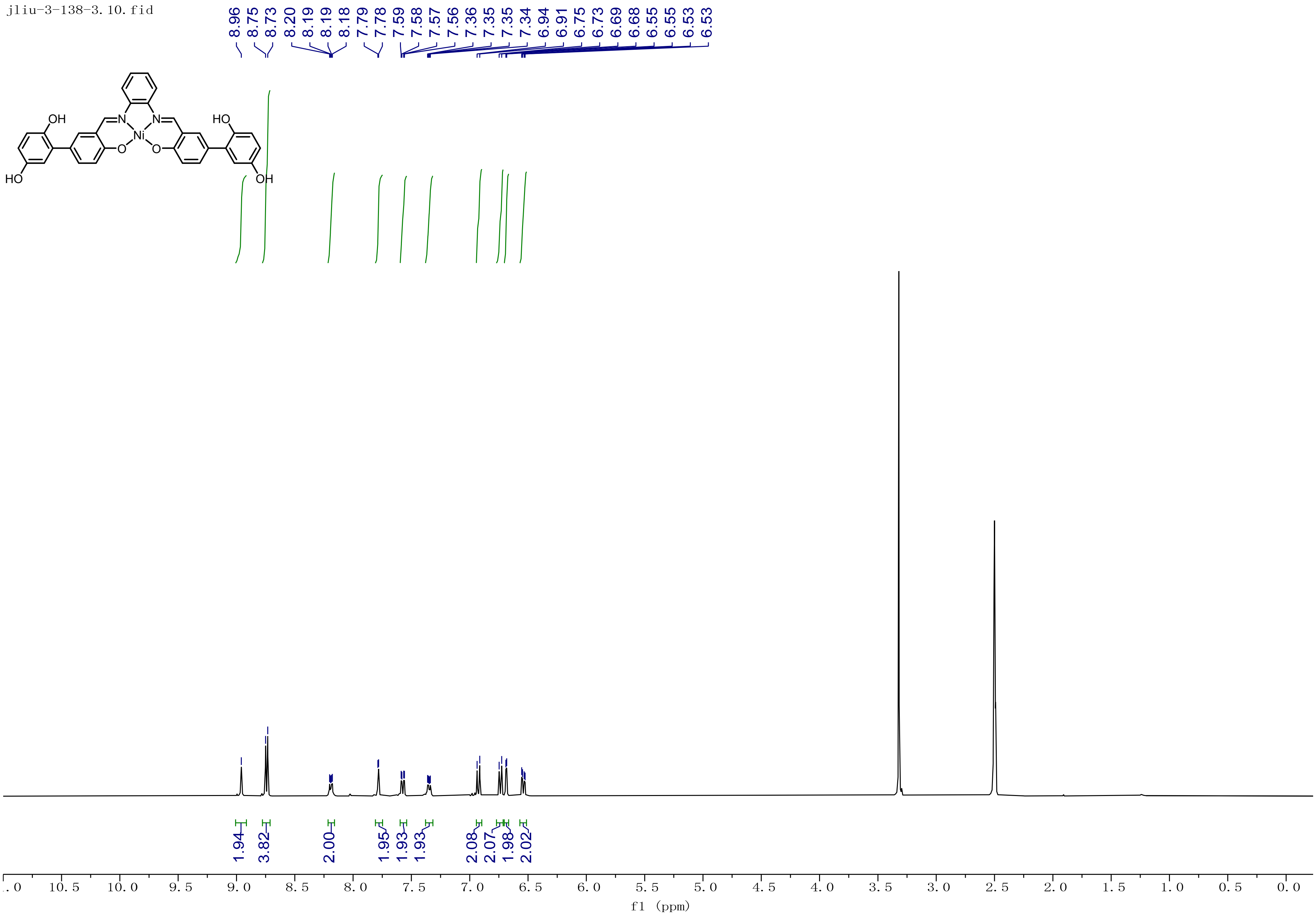
jliu-3-138-3.10.fid
8.96
8.75
8.73
8.20
8.19
8.19
8.18
7.79
7.78
7.59
7.58
7.57
7.56
7.36
7.35
7.35
7.34
6.94
6.91
6.75
6.73
6.69
6.68
6.55
6.55
6.53
6.53
OH
HO
N
Ni
O
1.94
3.82
2.00
1.95
1.93
1.93
2.08
2.07
1.98
2.02
f1 (ppm)

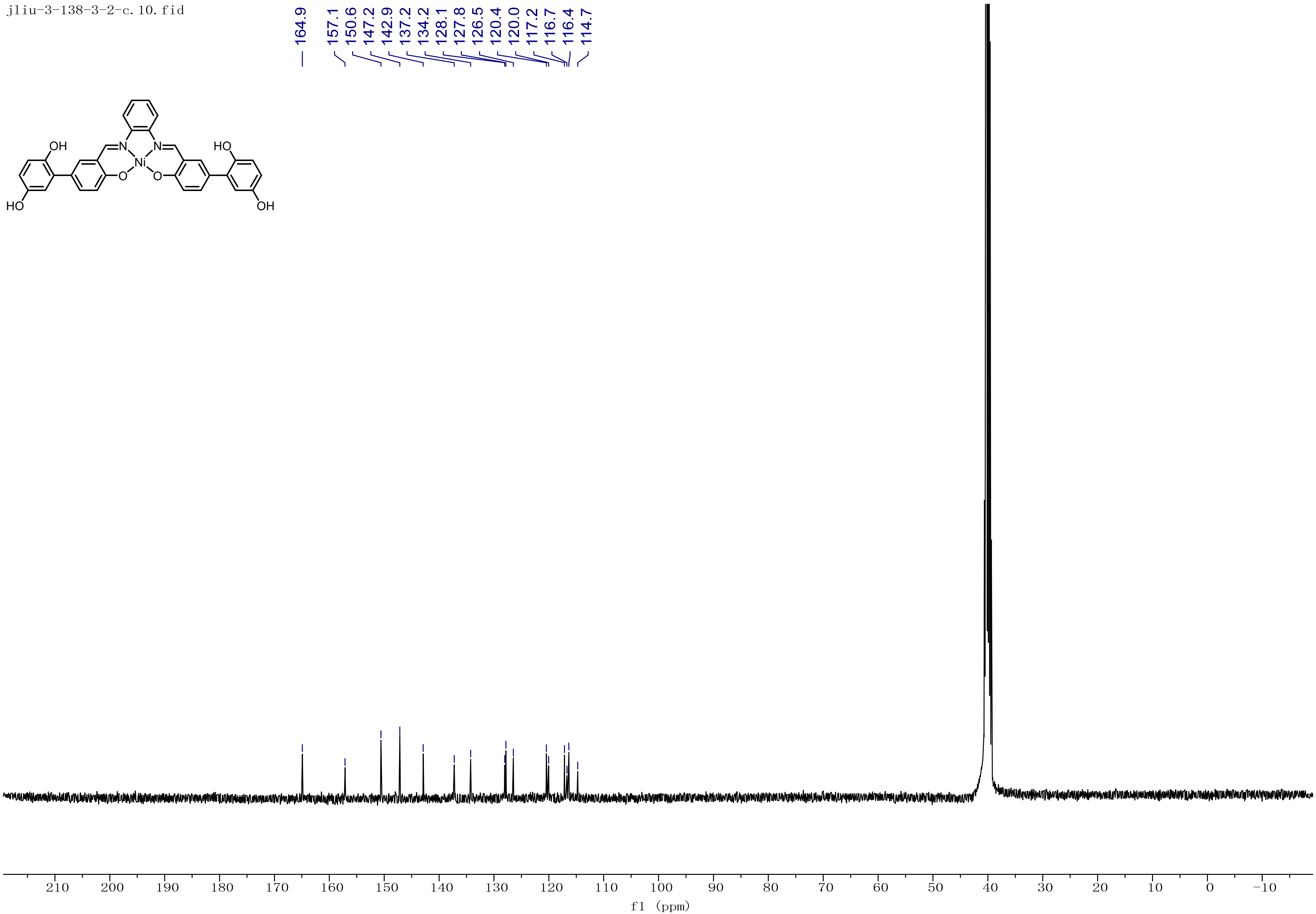
jliu-3-138-3-2-c.10.fid
164.9
157.1
150.6
147.2
142.9
137.2
134.2
128.1
127.8
126.5
120.4
120.0
117.2
116.7
116.4
114.7
OH
HO
N
N
Ni
O
O
HO
OH
210
200
190
180
170
160
150
140
130
120
110
100
90
80
70
60
50
40
30
20
10
0
−10
f1 (ppm)

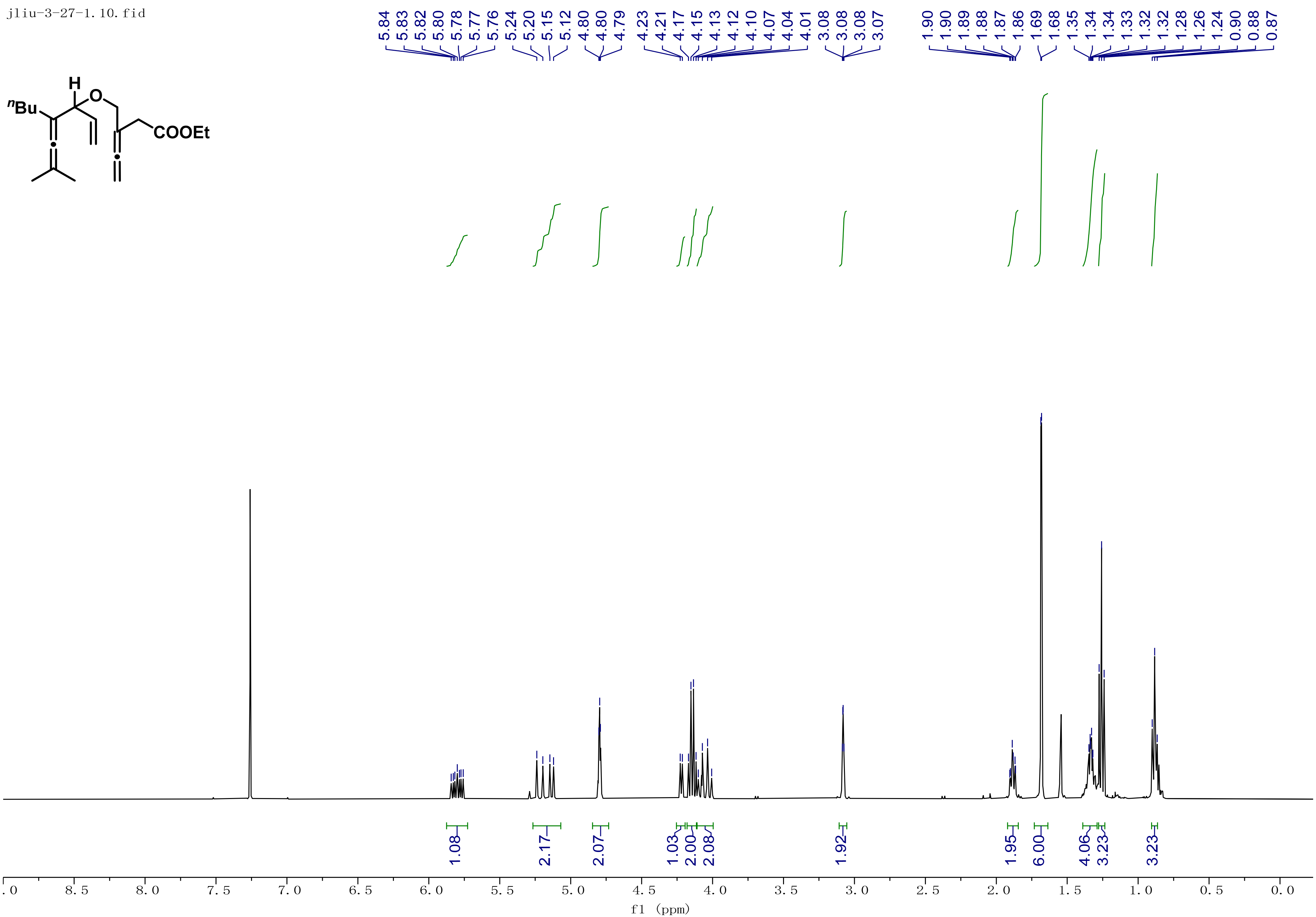

jliu-3-27-1.10.fid
5.84 5.83 5.82 5.80 5.78 5.77 5.76 5.24 5.20 5.15 5.12 4.80 4.80 4.79 4.23 4.21 4.17 4.15 4.13 4.12 4.10 4.07 4.04 4.01 3.08 3.08 3.08 3.07 1.90 1.90 1.89 1.88 1.87 1.86 1.69 1.68 1.35 1.34 1.34 1.33 1.32 1.32 1.28 1.26 1.24 0.90 0.88 0.87
H
O
^{n}Bu
COOEt
1.08 2.17 2.07 1.03 2.00 2.08 1.92 1.95 6.00 4.06 3.23 3.23
.0 8.5 8.0 7.5 7.0 6.5 6.0 5.5 5.0 4.5 4.0 3.5 3.0 2.5 2.0 1.5 1.0 0.5 0.0
f1 (ppm)

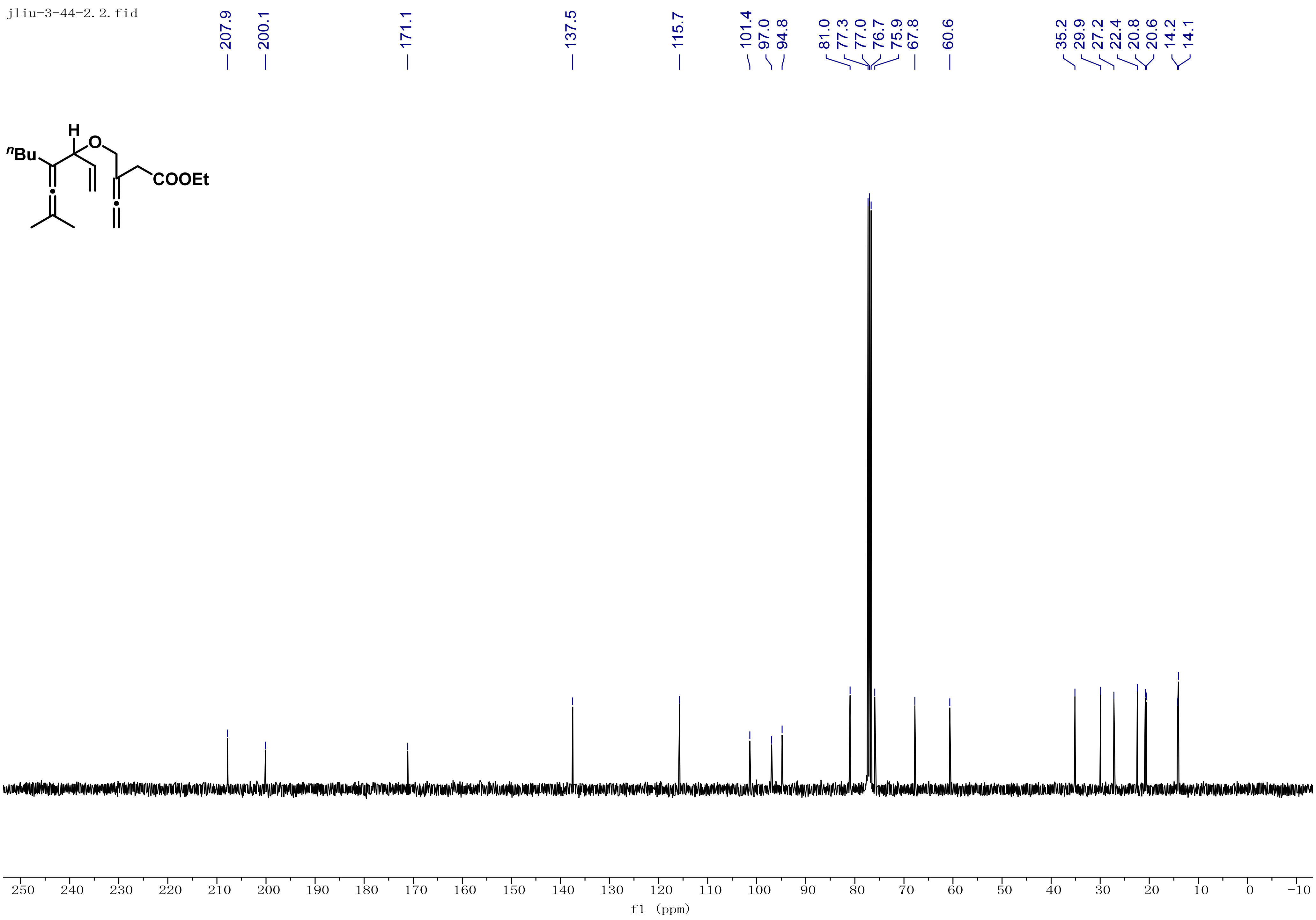
jliu-3-44-2.2.fid
207.9
200.1
171.1
137.5
115.7
101.4
97.0
94.8
81.0
77.3
77.0
76.7
75.9
67.8
60.6
35.2
29.9
27.2
22.4
20.8
20.6
14.2
14.1
H
O
^{n}Bu
COOEt
250 240 230 220 210 200 190 180 170 160 150 140 130 120 110 100 90 80 70 60 50 40 30 20 10 0 −10
f1 (ppm)

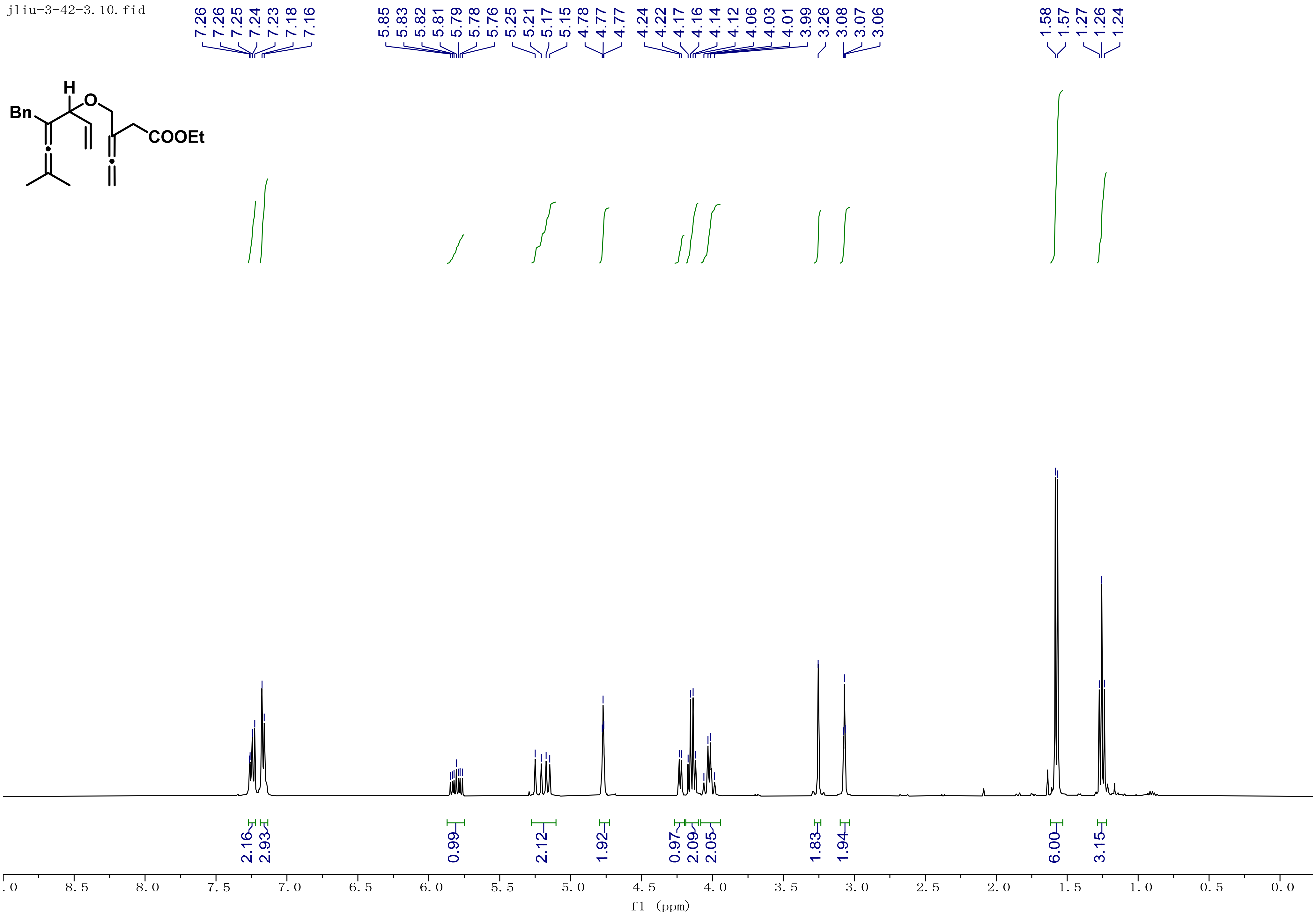
jliu-3-42-3.10.fid
7.26 7.26 7.25 7.24 7.23 7.18 7.16
5.85 5.83 5.82 5.81 5.79 5.78 5.76 5.25 5.21 5.17 5.15 4.78 4.77 4.77
4.24 4.22 4.17 4.16 4.14 4.12 4.06 4.03 4.01 3.99 3.26 3.08 3.07 3.06
1.58 1.57 1.27 1.26 1.24
Bn
H
O
COOEt
2.16 2.93 0.99 2.12 1.92 0.97 2.09 2.05 1.83 1.94 6.00 3.15
f1 (ppm)

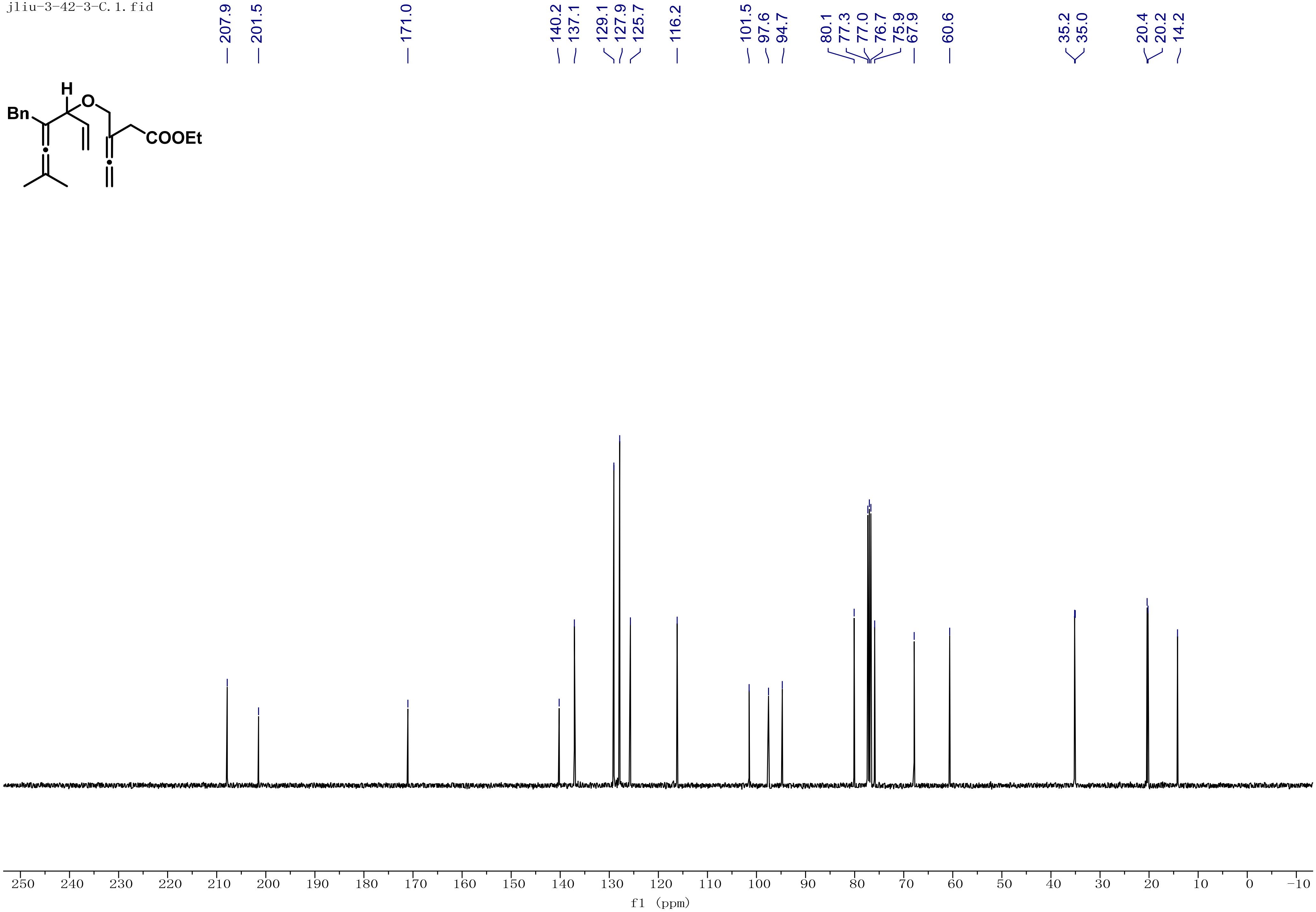
jliu-3-42-3-C.1.fid
207.9
201.5
171.0
140.2
137.1
129.1
127.9
125.7
116.2
101.5
97.6
94.7
80.1
77.3
77.0
76.7
75.9
67.9
60.6
35.2
35.0
20.4
20.2
14.2
Bn
H
O
COOEt
250 240 230 220 210 200 190 180 170 160 150 140 130 120 110 100 90 80 70 60 50 40 30 20 10 0 −10
f1 (ppm)

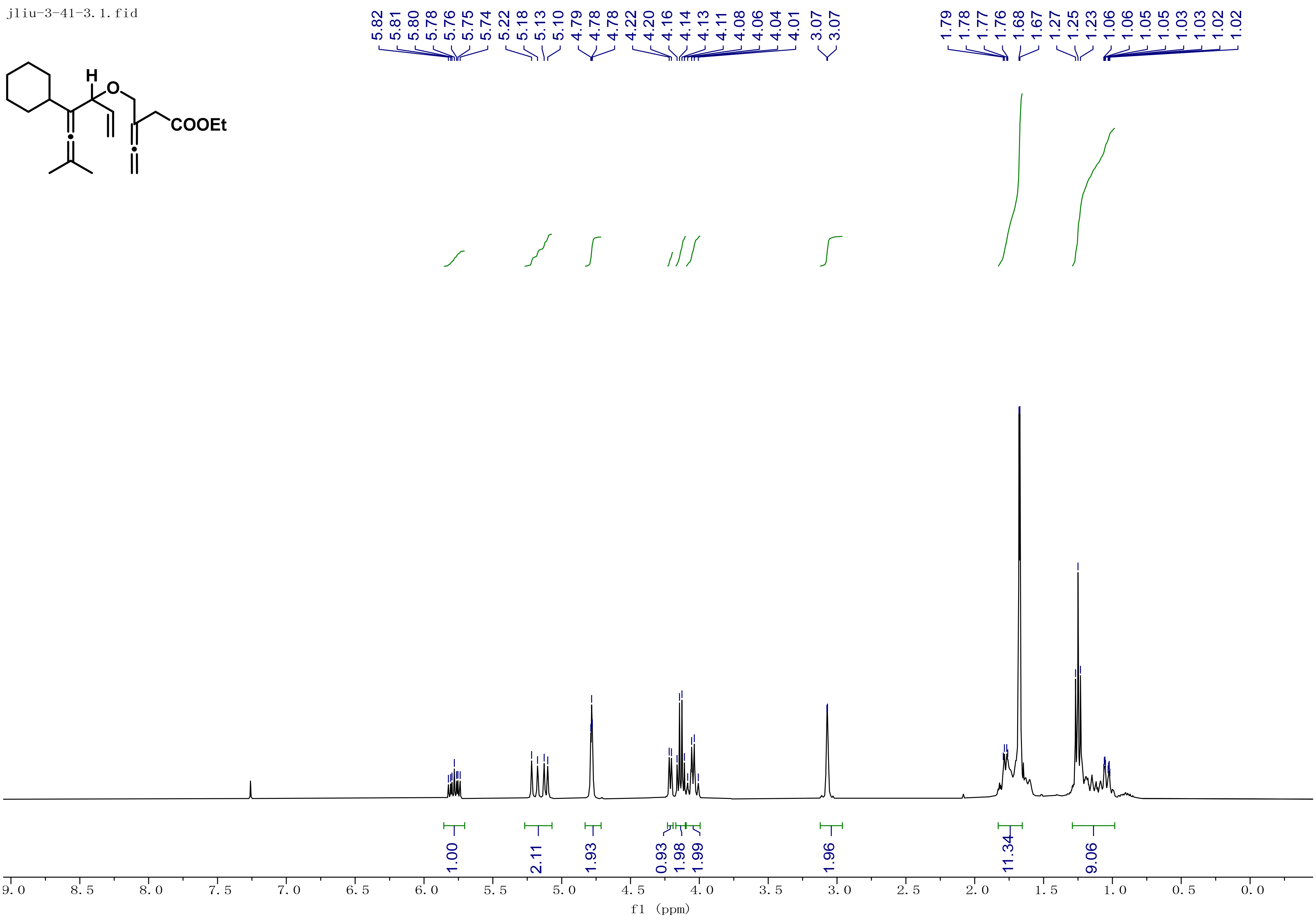
jliu-3-41-3.1.fid
COOEt
5.82 5.81 5.80 5.78 5.76 5.75 5.74 5.22 5.18 5.13 5.10 4.79 4.78 4.78 4.22 4.20 4.16 4.14 4.13 4.11 4.08 4.06 4.04 4.01 3.07 3.07
1.79 1.78 1.77 1.76 1.68 1.67 1.27 1.25 1.23 1.06 1.06 1.05 1.05 1.03 1.03 1.02 1.02
1.00 2.11 1.93 0.93 1.98 1.99 1.96 11.34 9.06
9.0 8.5 8.0 7.5 7.0 6.5 6.0 5.5 5.0 4.5 4.0 3.5 3.0 2.5 2.0 1.5 1.0 0.5 0.0
f1 (ppm)

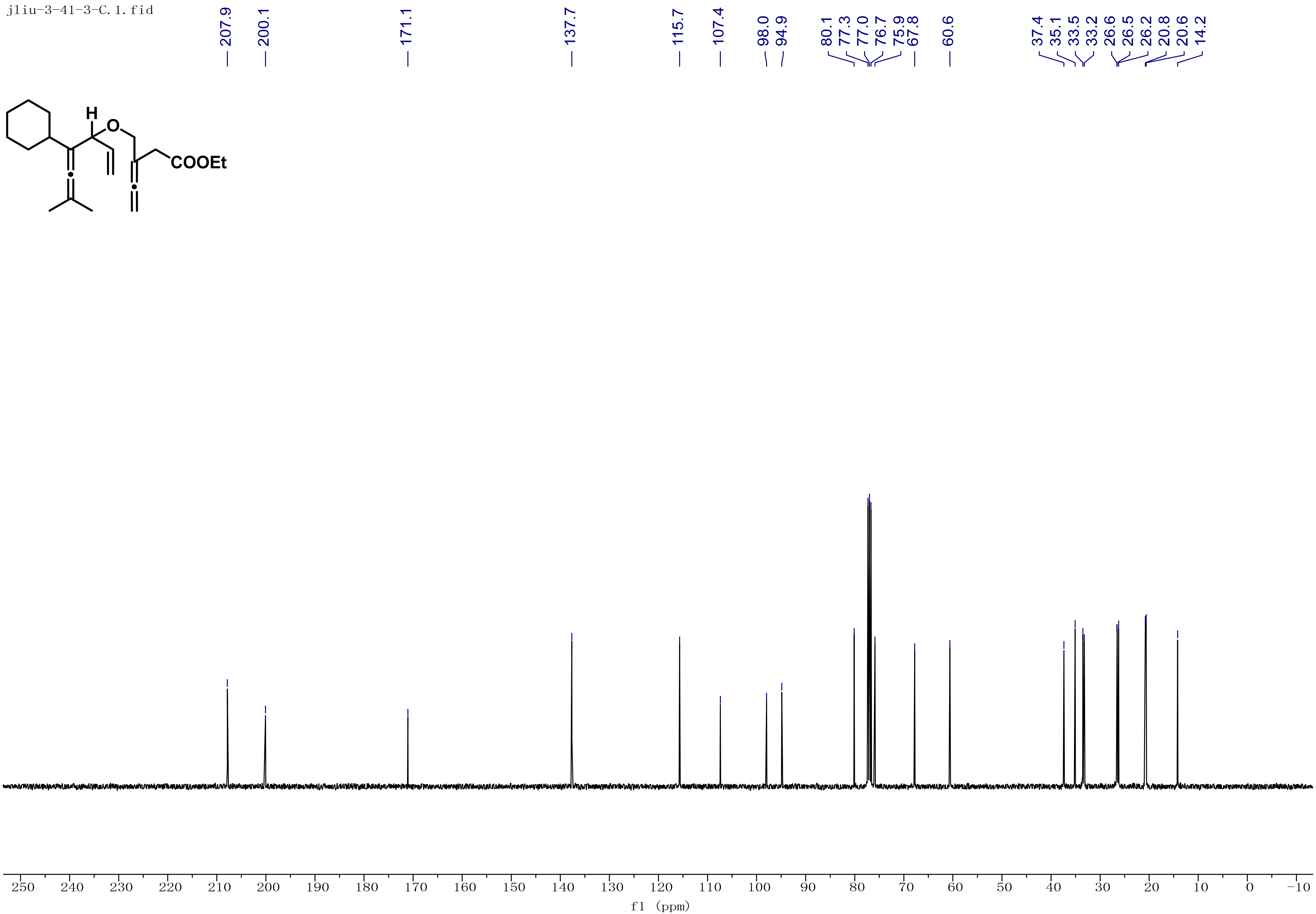
jliu-3-41-3-C.1.fid
207.9
200.1
171.1
137.7
115.7
107.4
98.0
94.9
80.1
77.3
77.0
76.7
75.9
67.8
60.6
37.4
35.1
33.5
33.2
26.6
26.5
26.2
20.8
20.6
14.2
H
O
COOEt
250 240 230 220 210 200 190 180 170 160 150 140 130 120 110 100 90 80 70 60 50 40 30 20 10 0 −10
f1 (ppm)

jliu-3-49-5.1.fid

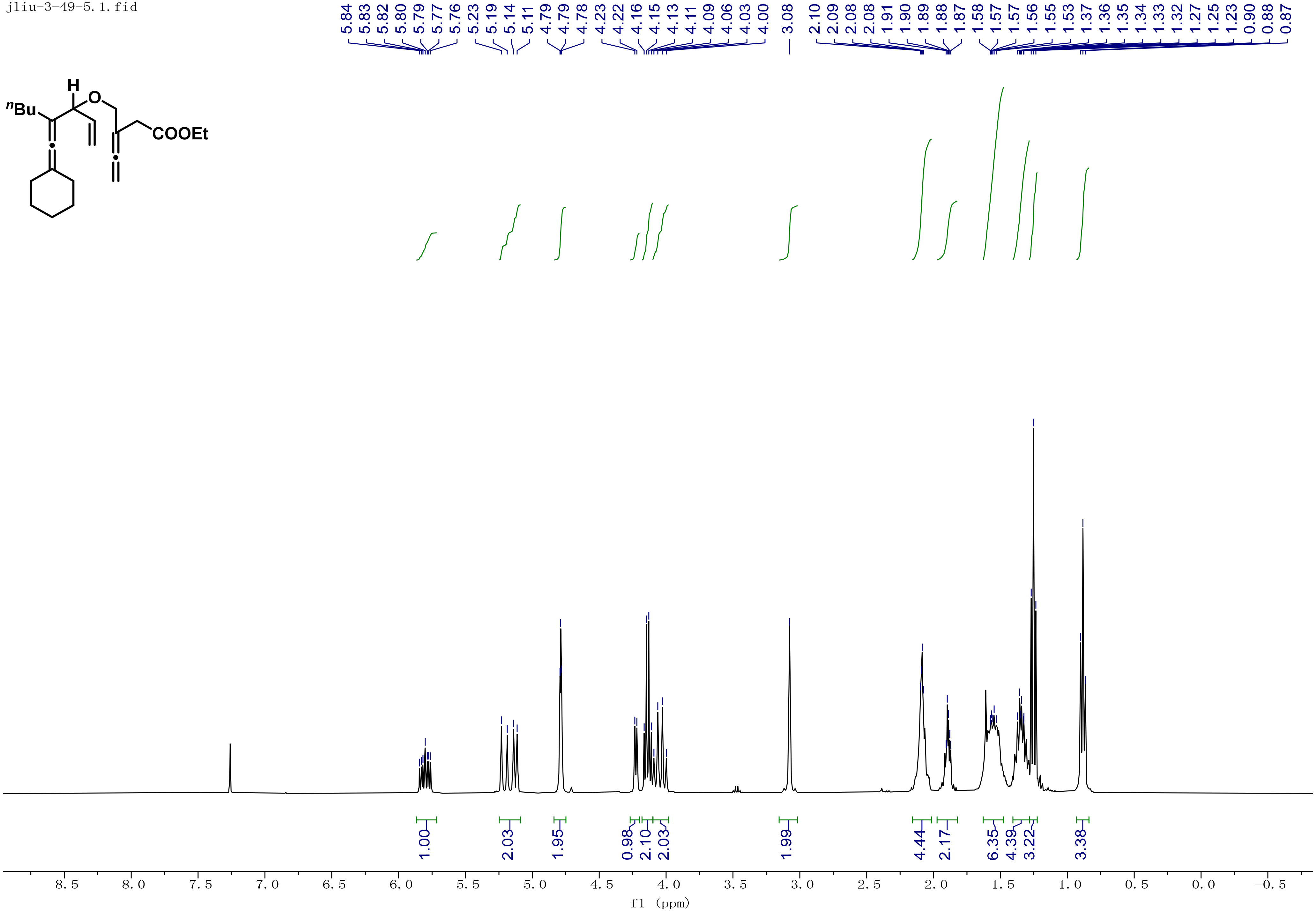

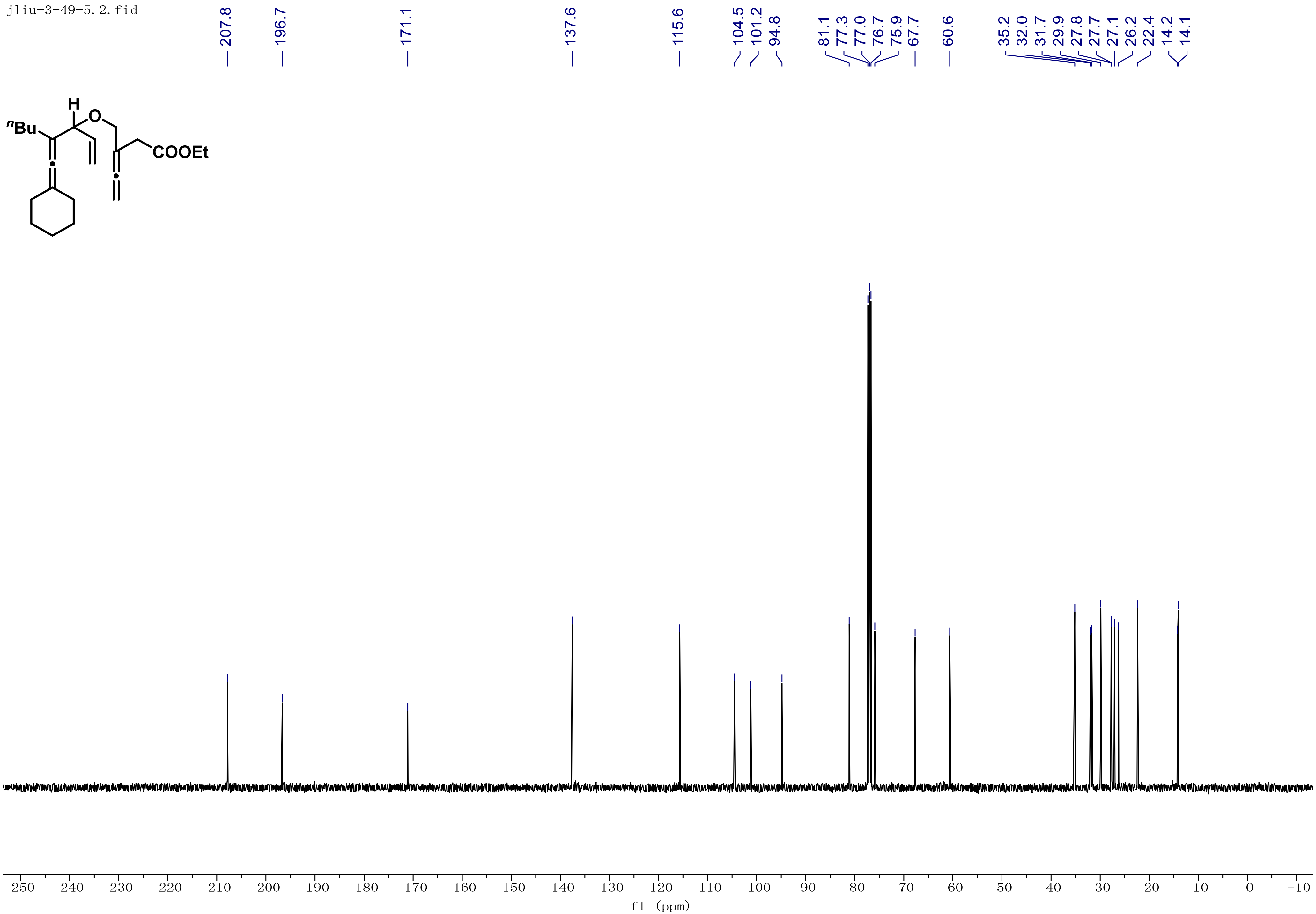
jliu-3-49-5.2.fid
207.8
196.7
171.1
137.6
115.6
104.5
101.2
94.8
81.1
77.3
77.0
76.7
75.9
67.7
60.6
35.2
32.0
31.7
29.9
27.8
27.7
27.1
26.2
22.4
14.2
14.1
nBu
H
O
COOEt
250 240 230 220 210 200 190 180 170 160 150 140 130 120 110 100 90 80 70 60 50 40 30 20 10 0 -10
f1 (ppm)

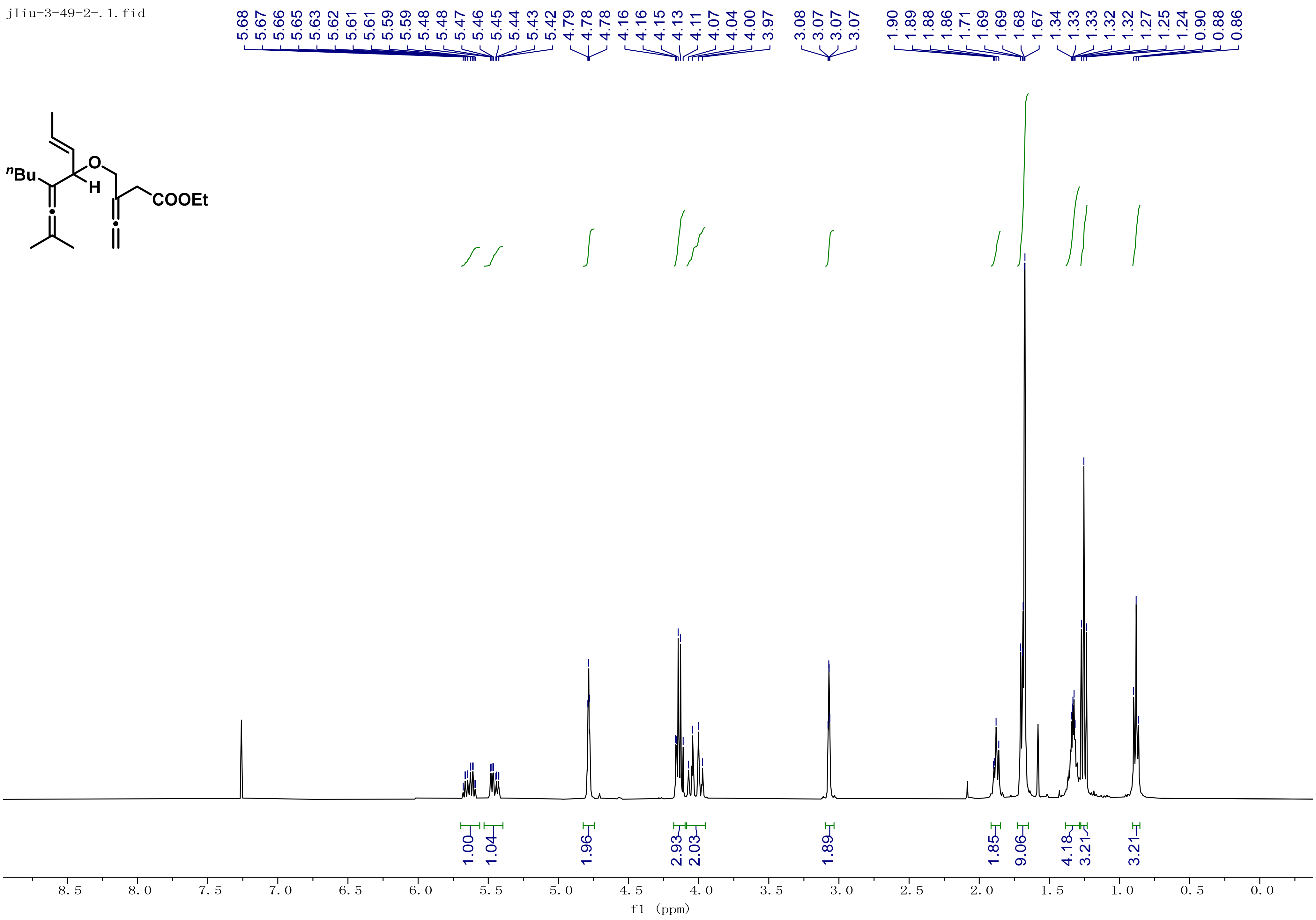
jliu-3-49-2-.1.fid
5.68 5.67 5.66 5.65 5.63 5.62 5.61 5.61 5.59 5.59 5.48 5.48 5.47 5.46 5.45 5.44 5.43 5.42 4.79 4.78 4.78 4.16 4.16 4.15 4.13 4.11 4.07 4.04 4.00 3.97 3.08 3.07 3.07 3.07 1.90 1.89 1.88 1.86 1.71 1.69 1.69 1.68 1.67 1.34 1.33 1.33 1.32 1.32 1.27 1.25 1.24 0.90 0.88 0.86
^{n}Bu
O
H
COOEt
1.00 1.04 1.96 2.93 2.03 1.89 1.85 9.06 4.18 3.21 3.21
8.5 8.0 7.5 7.0 6.5 6.0 5.5 5.0 4.5 4.0 3.5 3.0 2.5 2.0 1.5 1.0 0.5 0.0
f1 (ppm)

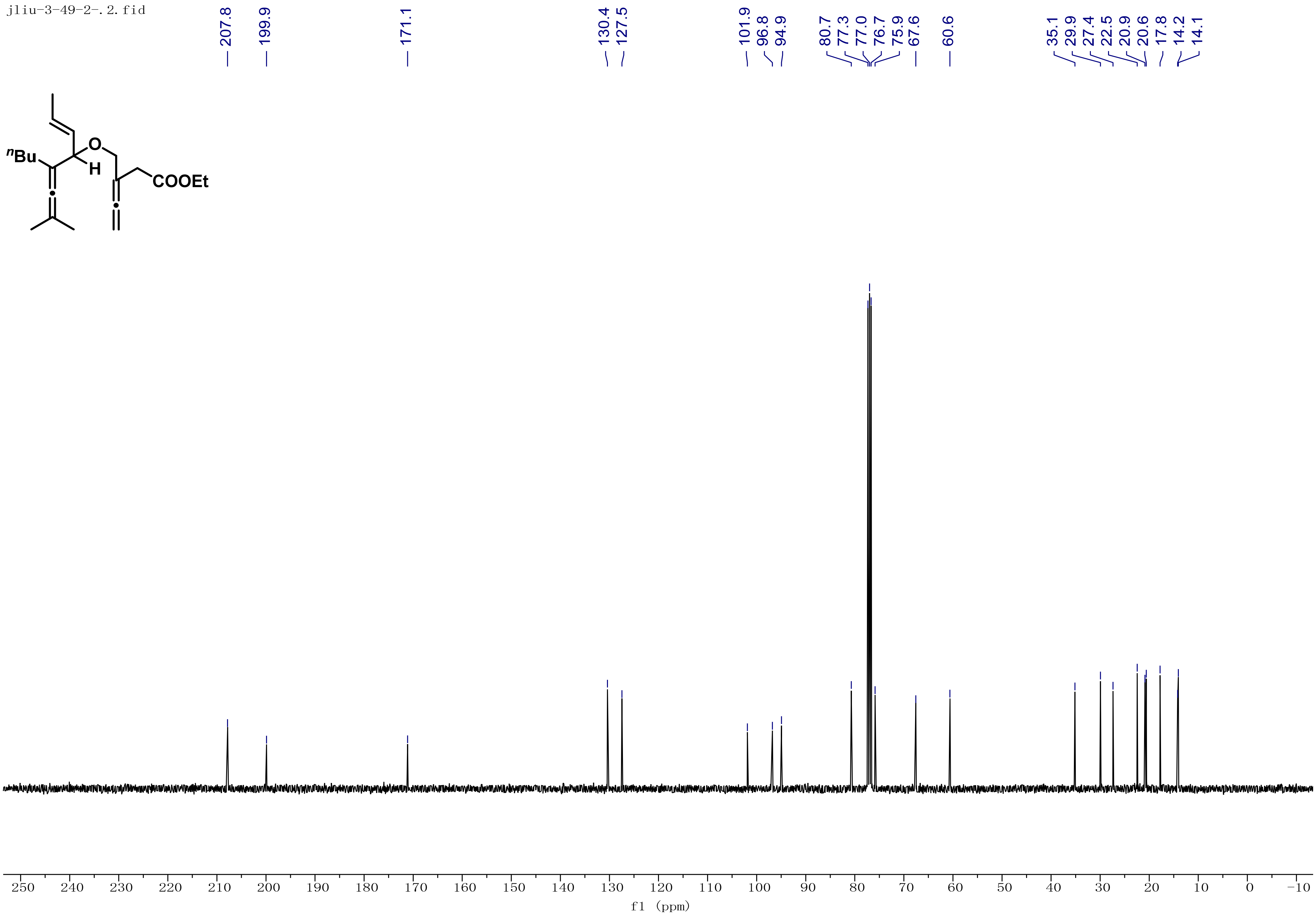
jliu-3-49-2-.2.fid
207.8
199.9
171.1
130.4
127.5
101.9
96.8
94.9
80.7
77.3
77.0
76.7
75.9
67.6
60.6
35.1
29.9
27.4
22.5
20.9
20.6
17.8
14.2
14.1
nBu
O
H
COOEt
250 240 230 220 210 200 190 180 170 160 150 140 130 120 110 100 90 80 70 60 50 40 30 20 10 0 -10
f1 (ppm)

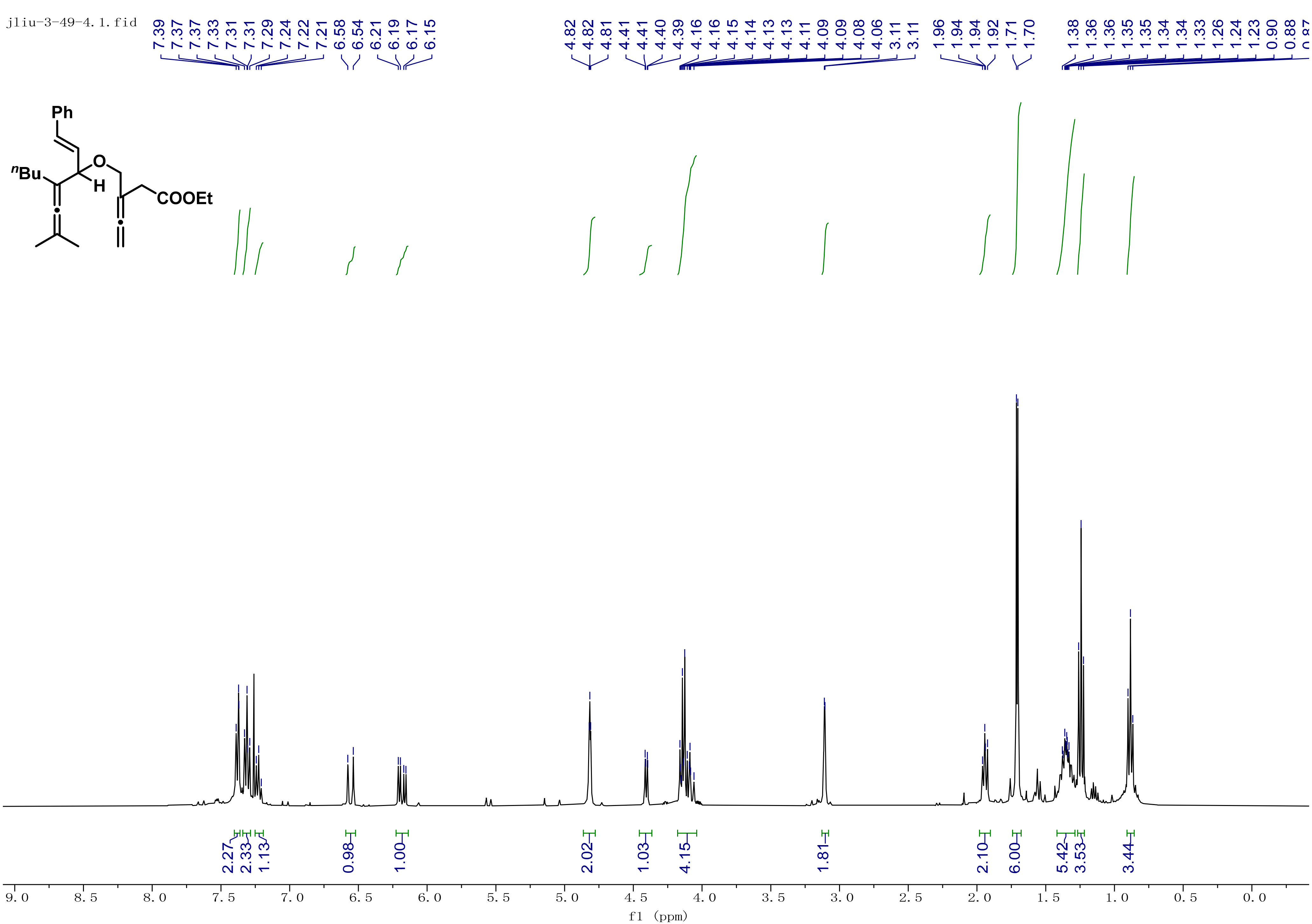

jliu-3-49-4.1.fid
7.39 7.37 7.37 7.33 7.31 7.31 7.29 7.24 7.22 7.21 6.58 6.54 6.21 6.19 6.17 6.15
4.82 4.82 4.81 4.41 4.41 4.40 4.39 4.16 4.16 4.15 4.14 4.13 4.13 4.11 4.09 4.09 4.08 4.06 3.11 3.11
1.96 1.94 1.94 1.92 1.71 1.70
1.38 1.36 1.36 1.35 1.35 1.34 1.34 1.33 1.26 1.24 1.23 0.90 0.88
Ph
O
^{n}Bu
H
COOEt
2.27
2.33
1.13
0.98
1.00
2.02
1.03
4.15
1.81
2.10
6.00
5.42
3.53
3.44
9.0 8.5 8.0 7.5 7.0 6.5 6.0 5.5 5.0 4.5 4.0 3.5 3.0 2.5 2.0 1.5 1.0 0.5 0.0
f1 (ppm)

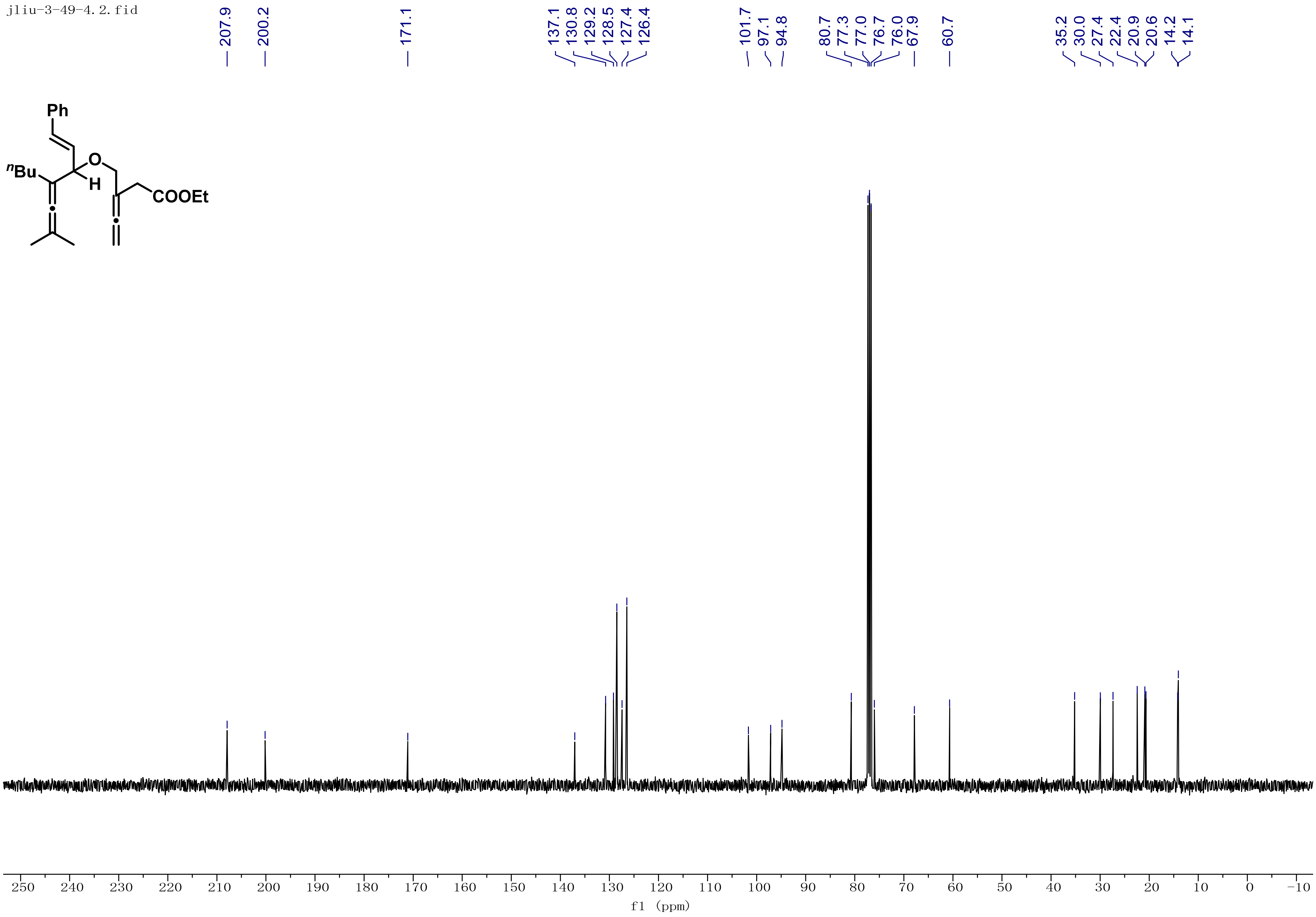
jliu-3-49-4.2.fid
207.9
200.2
171.1
137.1
130.8
129.2
128.5
127.4
126.4
101.7
97.1
94.8
80.7
77.3
77.0
76.7
76.0
67.9
60.7
35.2
30.0
27.4
22.4
20.9
20.6
14.2
14.1
Ph
O
nBu
H
COOEt
250 240 230 220 210 200 190 180 170 160 150 140 130 120 110 100 90 80 70 60 50 40 30 20 10 0 -10
f1 (ppm)

jliu-3-56-3/10

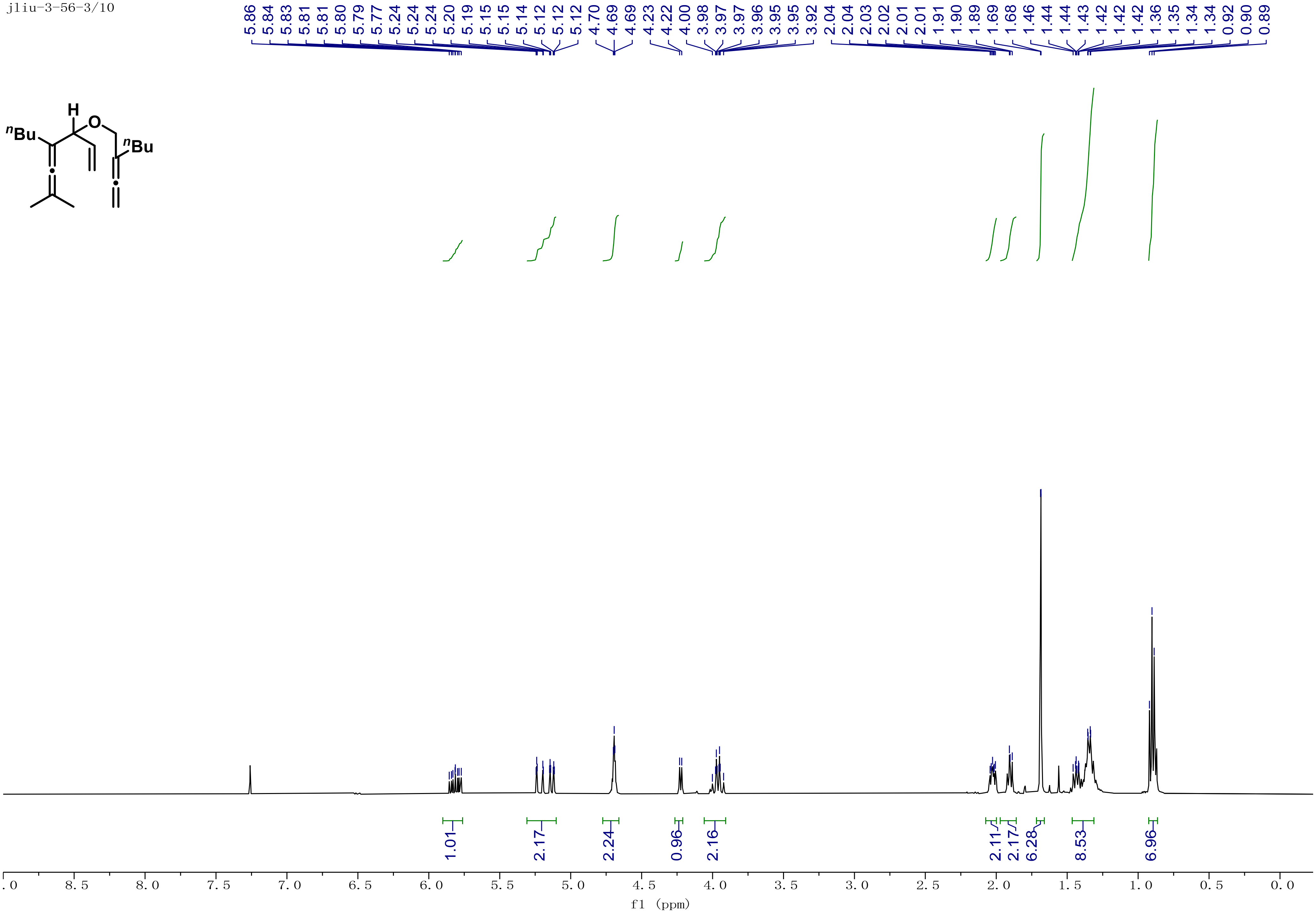
H
O
nBu
nBu
5.86 5.84 5.83 5.81 5.81 5.80 5.79 5.77 5.24 5.24 5.24 5.20 5.19 5.15 5.15 5.14 5.12 5.12 5.12 4.70 4.69 4.69 4.23 4.22 4.00 3.98 3.97 3.97 3.96 3.95 3.95 3.92 2.04 2.04 2.03 2.02 2.01 2.01 1.91 1.90 1.89 1.69 1.68 1.46 1.44 1.44 1.43 1.42 1.42 1.42 1.36 1.35 1.34 1.34 0.92 0.90 0.89
1.01
2.17
2.24
0.96
2.16
2.11
2.17
6.28
8.53
6.96
.0 8.5 8.0 7.5 7.0 6.5 6.0 5.5 5.0 4.5 4.0 3.5 3.0 2.5 2.0 1.5 1.0 0.5 0.0
f1 (ppm)

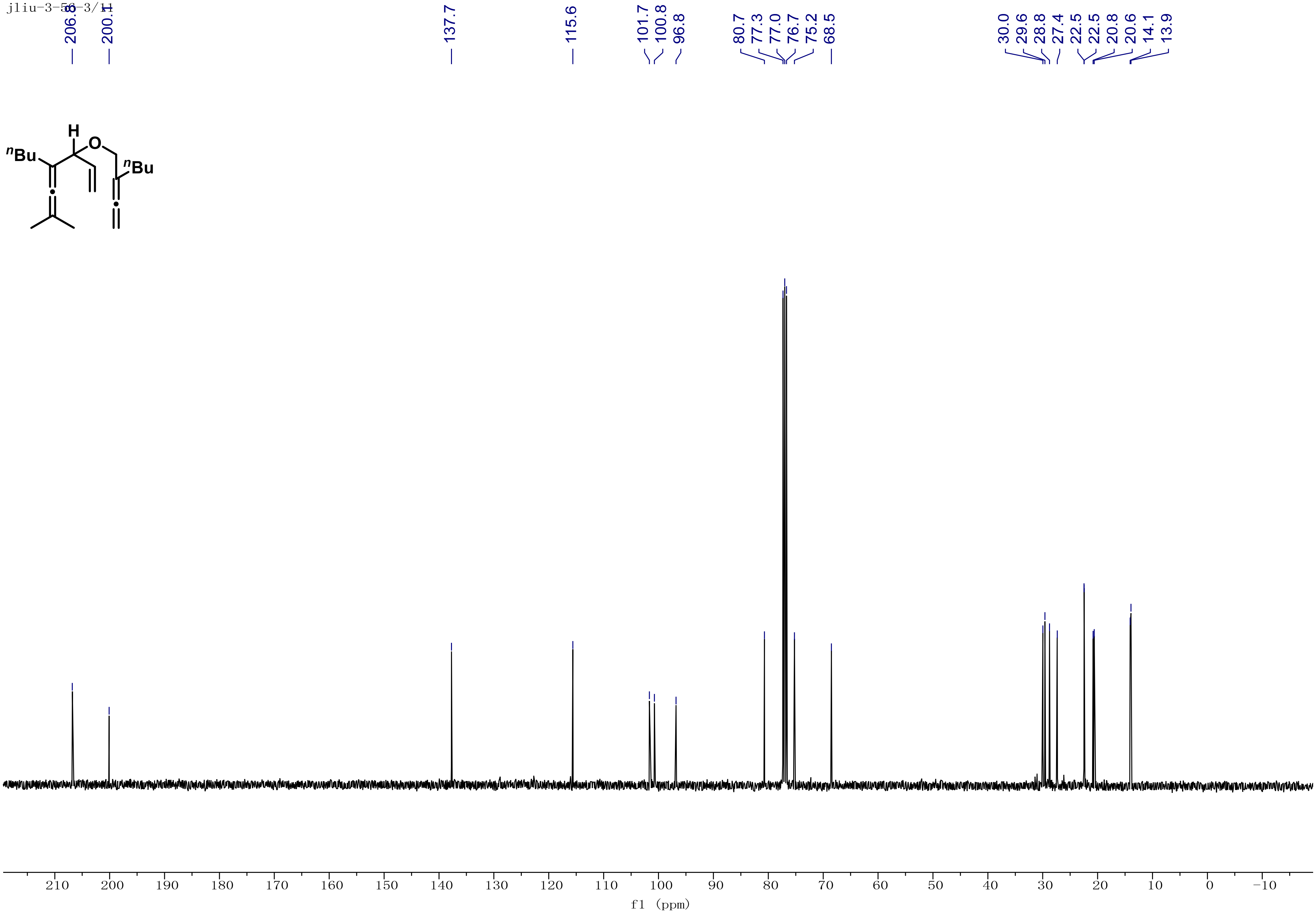
jliu-3-56-3/11
206.8
200.1
137.7
115.6
101.7
100.8
96.8
80.7
77.3
77.0
76.7
75.2
68.5
30.0
29.6
28.8
27.4
22.5
22.5
20.8
20.6
14.1
13.9
H
O
nBu
nBu
210 200 190 180 170 160 150 140 130 120 110 100 90 80 70 60 50 40 30 20 10 0 -10
f1 (ppm)

jliu-3-36-1-.10.fid

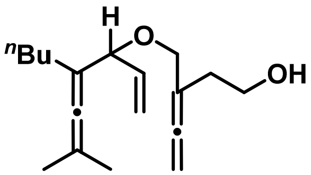

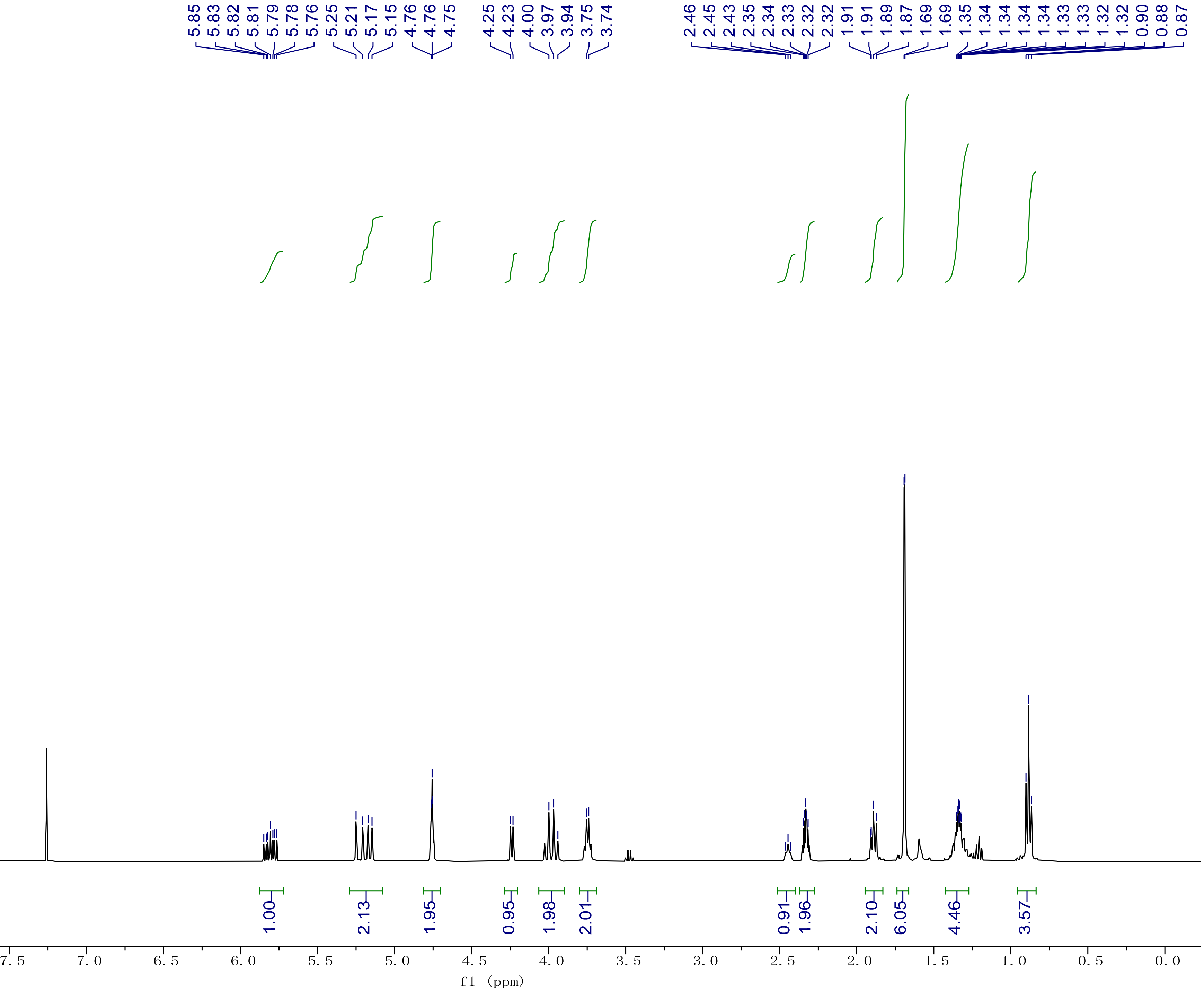

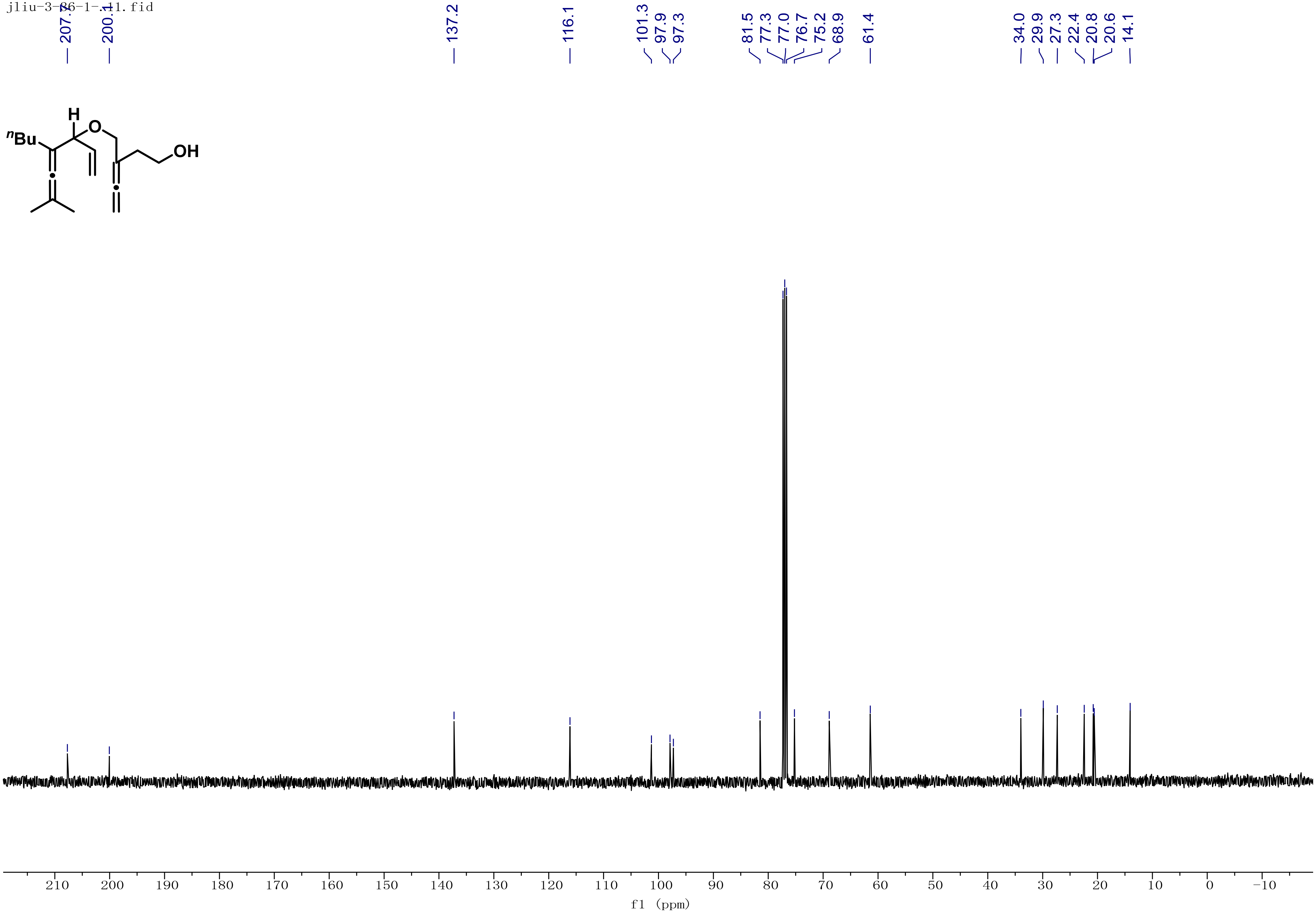
jliu-3-86-1-.11.fid
207.7
200.1
137.2
116.1
101.3
97.9
97.3
81.5
77.3
77.0
76.7
75.2
68.9
61.4
34.0
29.9
27.3
22.4
20.8
20.6
14.1
nBu
H
O
OH
210 200 190 180 170 160 150 140 130 120 110 100 90 80 70 60 50 40 30 20 10 0 −10
f1 (ppm)

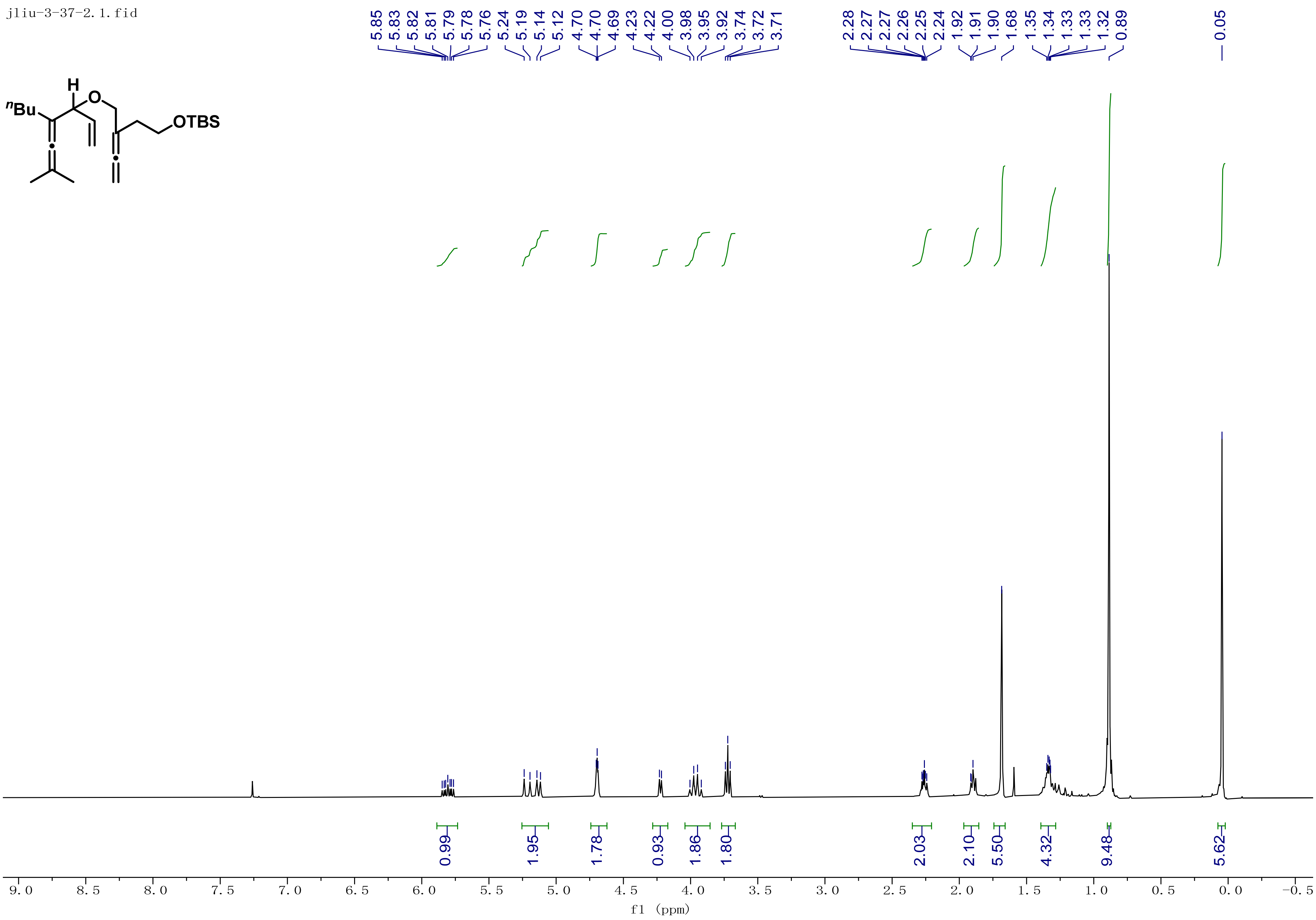

jliu-3-37-2.1.fid
nBu
H
O
OTBS
5.85
5.83
5.82
5.81
5.79
5.78
5.76
5.24
5.19
5.14
5.12
4.70
4.70
4.69
4.23
4.22
4.00
3.98
3.95
3.92
3.74
3.72
3.71
2.28
2.27
2.27
2.26
2.25
2.24
1.92
1.91
1.90
1.68
1.35
1.34
1.33
1.33
1.32
0.89
0.05
0.99
1.95
1.78
0.93
1.86
1.80
2.03
2.10
5.50
4.32
9.48
5.62
9.0
8.5
8.0
7.5
7.0
6.5
6.0
5.5
5.0
4.5
4.0
3.5
3.0
2.5
2.0
1.5
1.0
0.5
0.0
-0.5
f1 (ppm)

jliu-3-37-2.2.fid

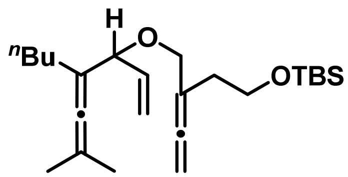

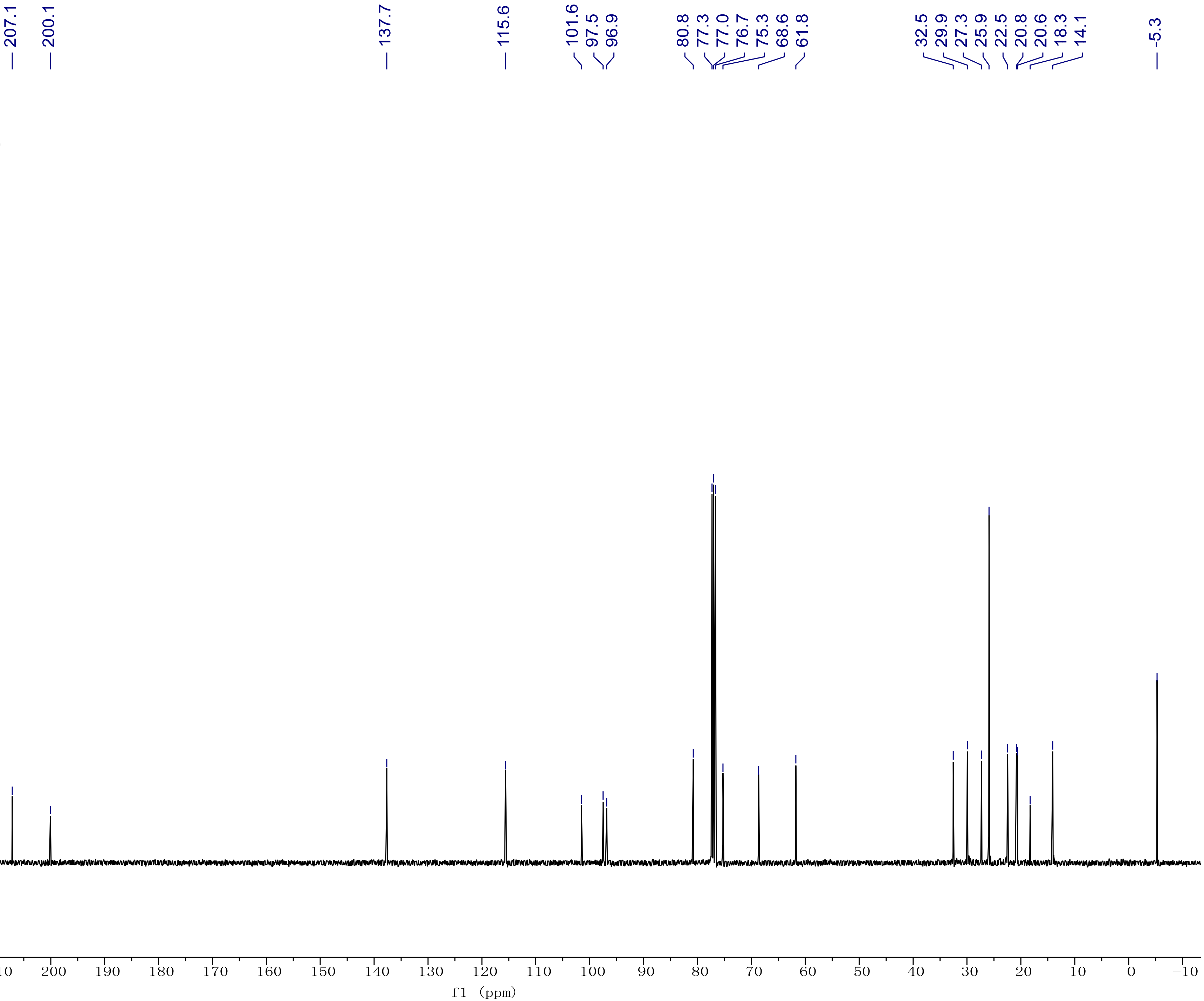

jliu-3-37-1.1.fid

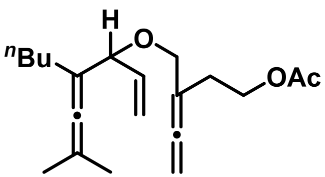

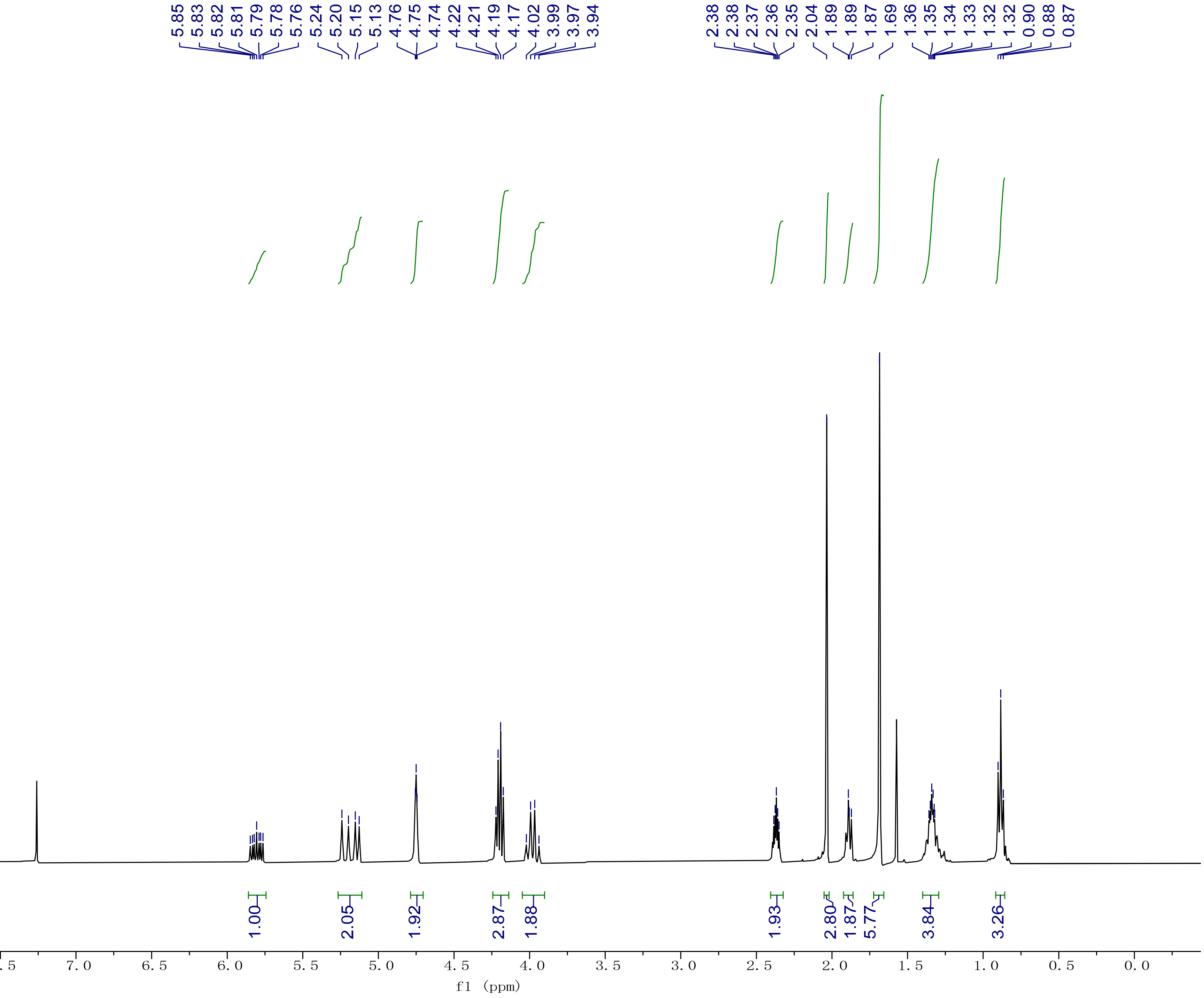

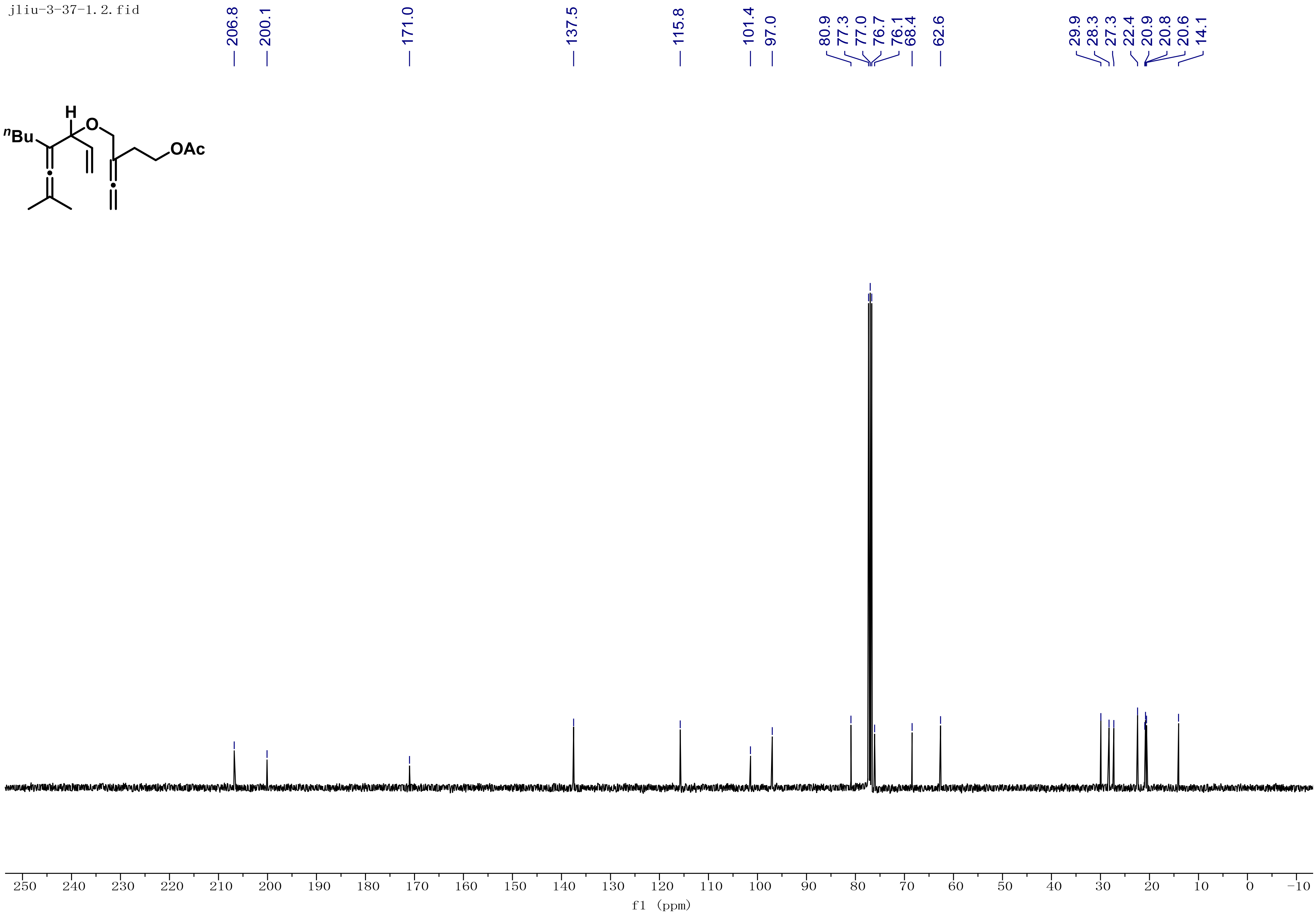

^{n}Bu
H
O
OAc
jliu-3-37-1.2.fid
206.8
200.1
171.0
137.5
115.8
101.4
97.0
80.9
77.3
77.0
76.7
76.1
68.4
62.6
29.9
28.3
27.3
22.4
20.9
20.8
20.6
14.1
250 240 230 220 210 200 190 180 170 160 150 140 130 120 110 100 90 80 70 60 50 40 30 20 10 0 -10
f1 (ppm)

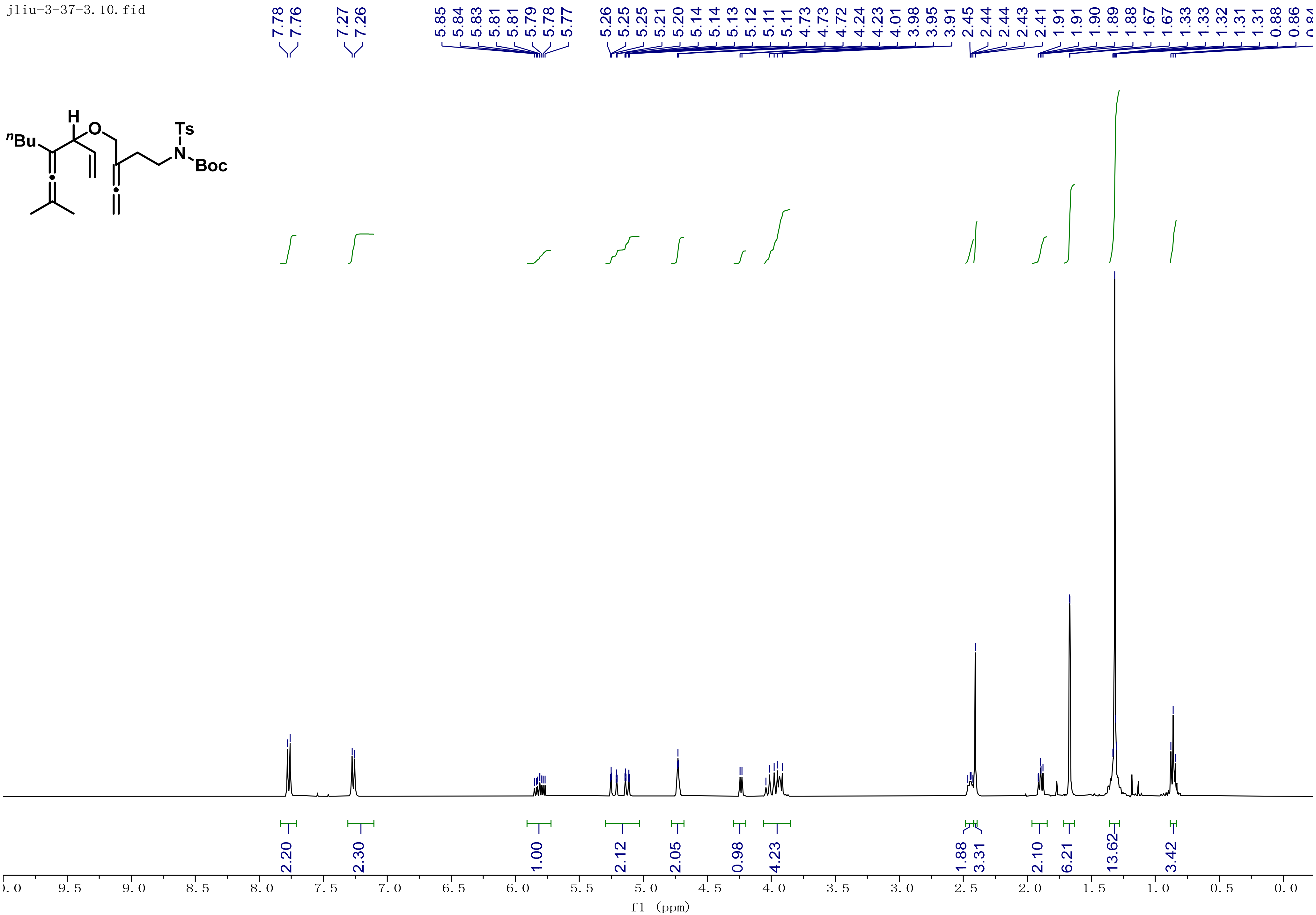
jliu-3-37-3.10.fid
7.78 7.76 7.27 7.26 5.85 5.84 5.83 5.81 5.81 5.79 5.78 5.77 5.26 5.25 5.25 5.21 5.20 5.14 5.14 5.13 5.12 5.11 5.11 4.73 4.73 4.72 4.24 4.23 4.01 3.98 3.95 3.91 2.45 2.44 2.44 2.43 2.41 1.91 1.91 1.90 1.89 1.88 1.67 1.67 1.33 1.33 1.32 1.31 1.31 0.88 0.86
nBu
H
O
Ts
N
Boc
2.20 2.30 1.00 2.12 2.05 0.98 4.23 1.88 3.31 2.10 6.21 13.62 3.42
9.5 9.0 8.5 8.0 7.5 7.0 6.5 6.0 5.5 5.0 4.5 4.0 3.5 3.0 2.5 2.0 1.5 1.0 0.5 0.0
f1 (ppm)

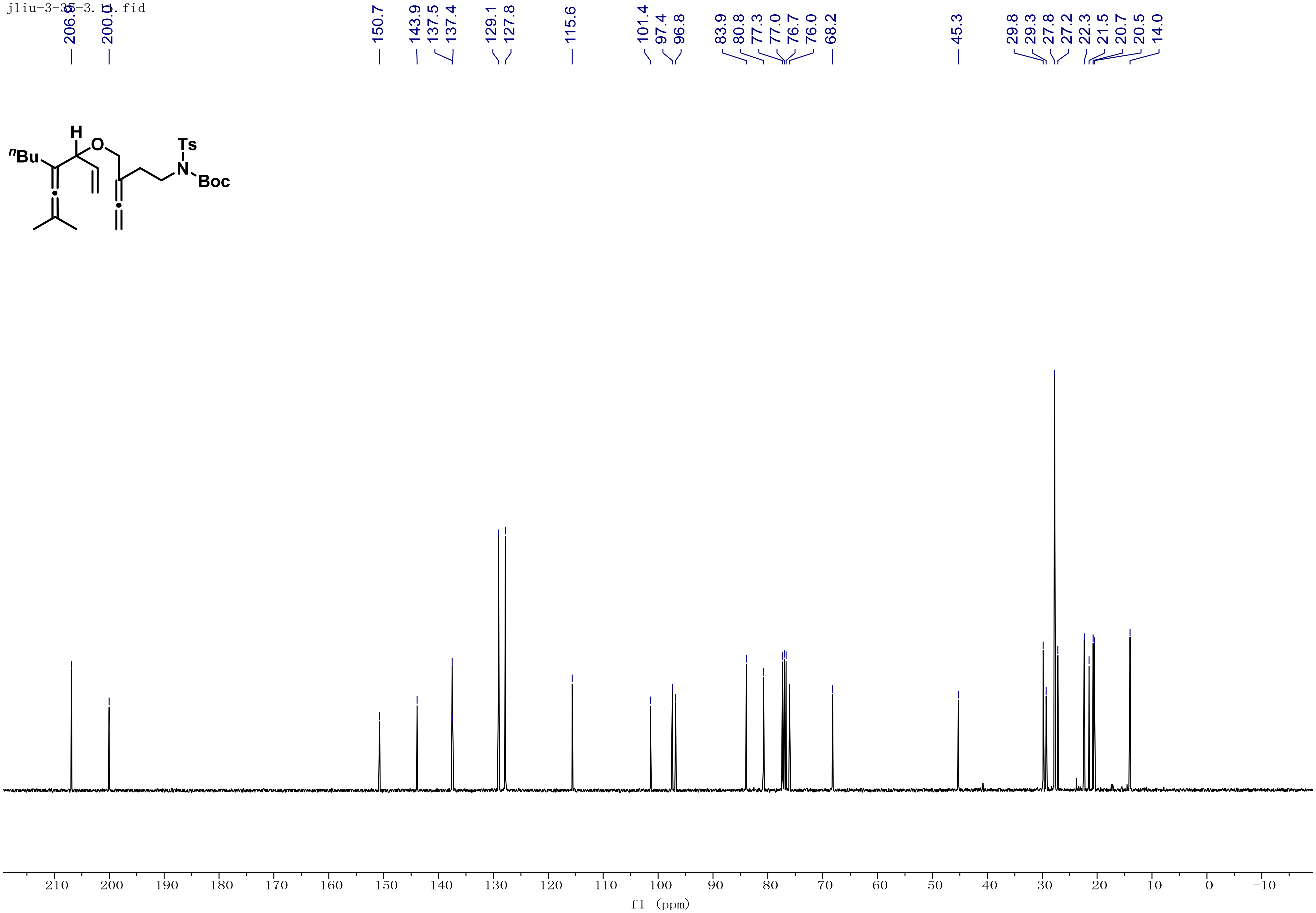
jliu-3-37-3.11.fid
206.9
200.0
150.7
143.9
137.5
137.4
129.1
127.8
115.6
101.4
97.4
96.8
83.9
80.8
77.3
77.0
76.7
76.0
68.2
45.3
29.8
29.3
27.8
27.2
22.3
21.5
20.7
20.5
14.0
H
O
^{n}Bu
Ts
N
Boc
210 200 190 180 170 160 150 140 130 120 110 100 90 80 70 60 50 40 30 20 10 0 -10
f1 (ppm)

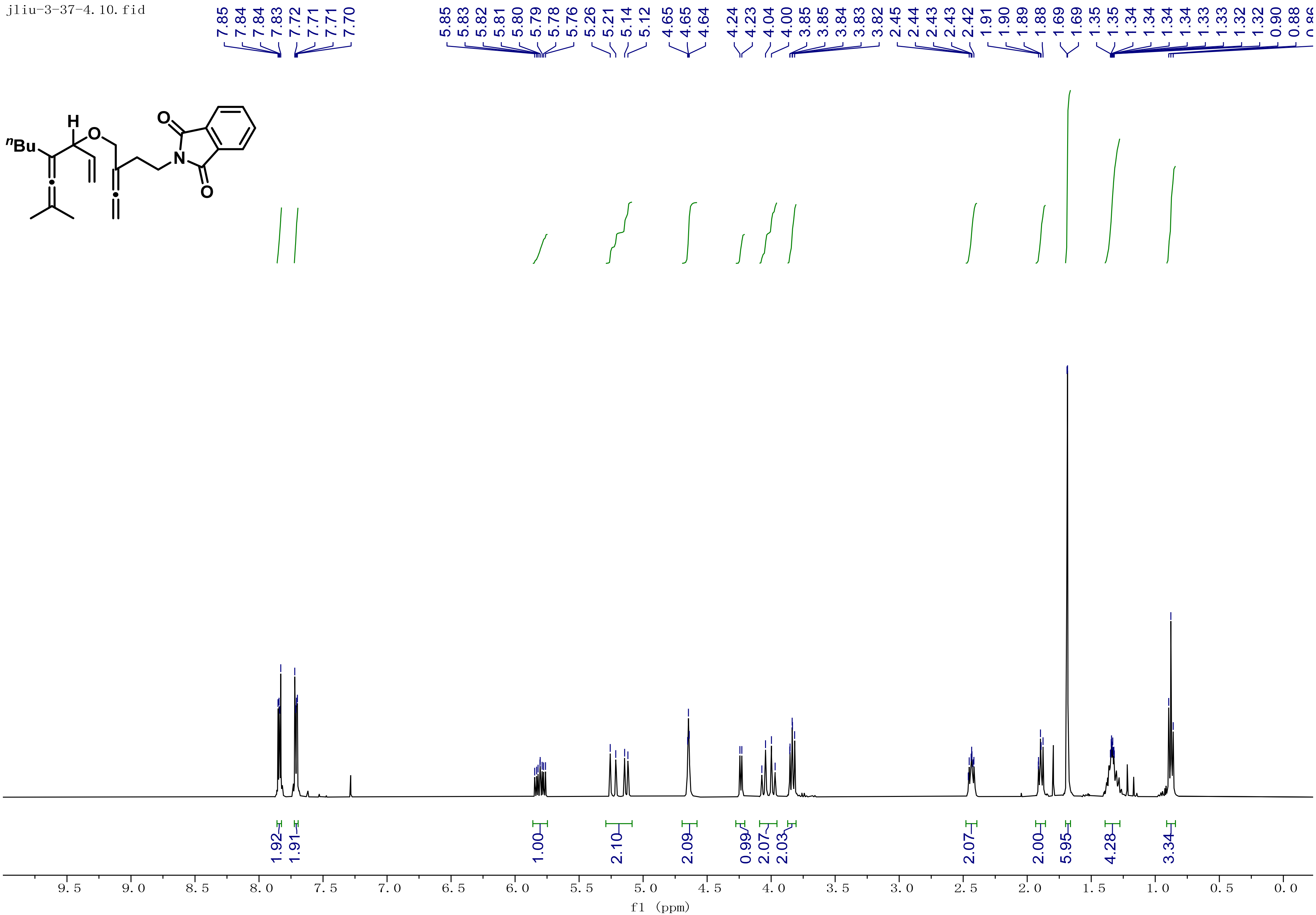
jliu-3-37-4.10.fid
7.85 7.84 7.84 7.83 7.72 7.71 7.71 7.70
5.85 5.83 5.82 5.81 5.80 5.79 5.78 5.76 5.26 5.21 5.14 5.12 4.65 4.65 4.64 4.24 4.23 4.04 4.00 3.85 3.85 3.84 3.83 3.82 2.45 2.44 2.43 2.43 2.42 1.91 1.90 1.89 1.88 1.69 1.69 1.35 1.35 1.34 1.34 1.34 1.34 1.33 1.33 1.32 1.32 0.90 0.88
ⁿBu
H
O
N
O
O
1.92
1.91
1.00
2.10
2.09
0.99
2.07
2.03
2.07
2.00
5.95
4.28
3.34
9.5 9.0 8.5 8.0 7.5 7.0 6.5 6.0 5.5 5.0 4.5 4.0 3.5 3.0 2.5 2.0 1.5 1.0 0.5 0.0
f1 (ppm)

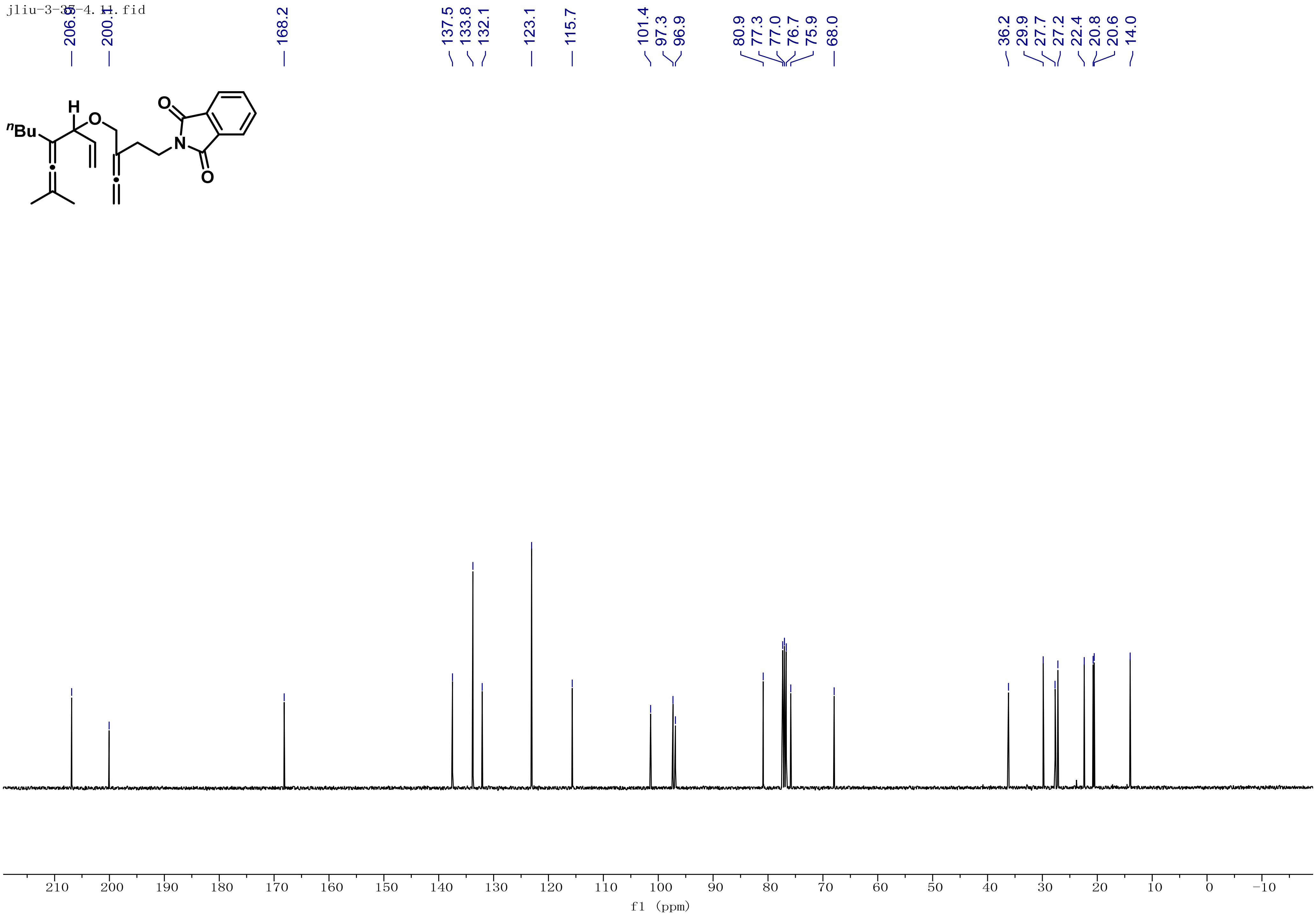
jliu-3-37-4.11.fid
206.9
200.1
168.2
137.5
133.8
132.1
123.1
115.7
101.4
97.3
96.9
80.9
77.3
77.0
76.7
75.9
68.0
36.2
29.9
27.7
27.2
22.4
20.8
20.6
14.0
ⁿBu
H
O
N
O
O
210 200 190 180 170 160 150 140 130 120 110 100 90 80 70 60 50 40 30 20 10 0 −10
f1 (ppm)

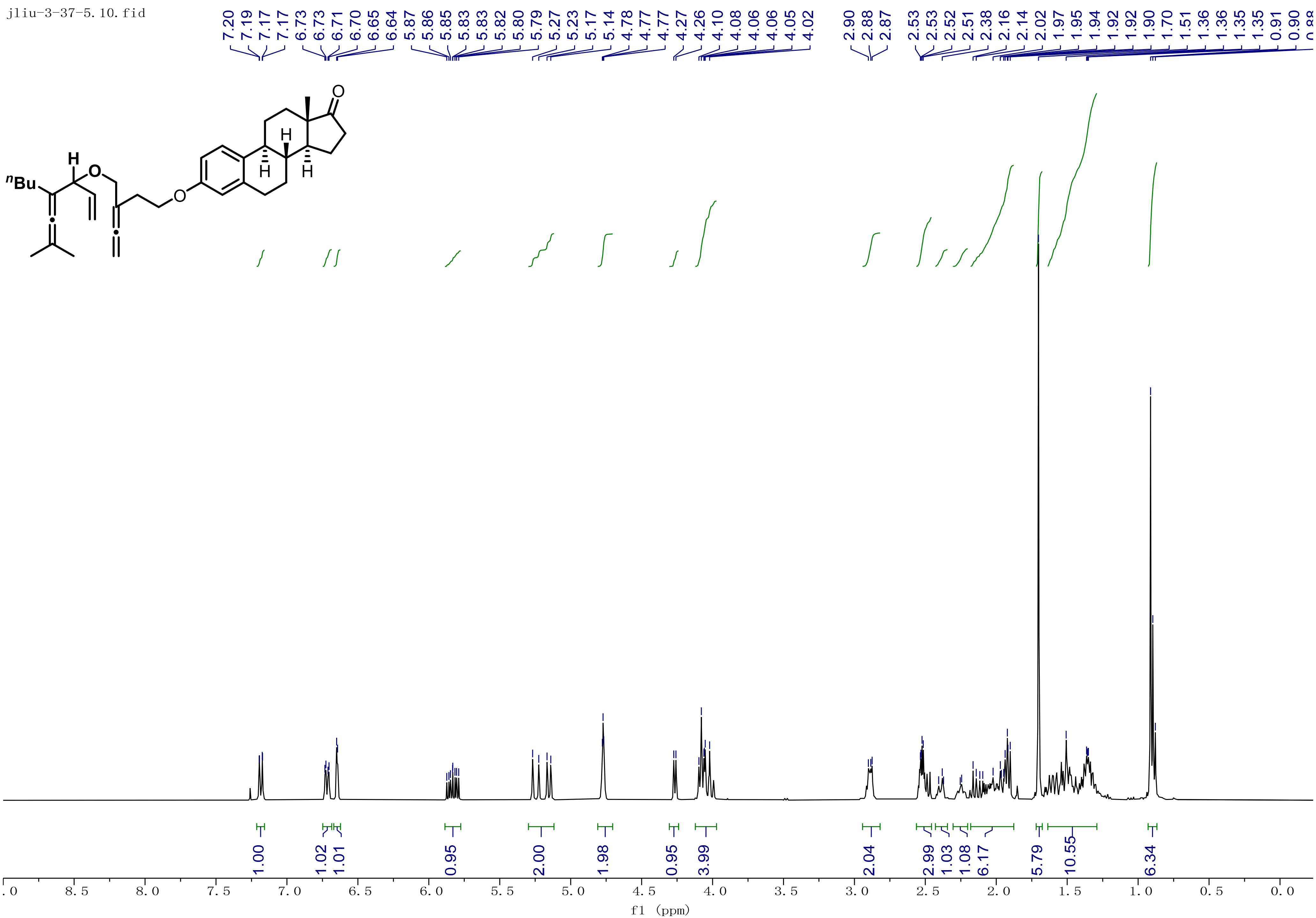
jliu-3-37-5.10.fid
7.20 7.19 7.17 7.17 6.73 6.73 6.71 6.70 6.65 6.64 5.87 5.86 5.85 5.83 5.83 5.82 5.80 5.79 5.27 5.23 5.17 5.14 4.78 4.77 4.77 4.27 4.26 4.10 4.08 4.06 4.06 4.05 4.02 2.90 2.88 2.87 2.53 2.53 2.52 2.51 2.38 2.16 2.14 2.02 1.97 1.95 1.94 1.92 1.92 1.90 1.70 1.51 1.36 1.36 1.35 1.35 0.91 0.90
nBu
H
O
O
H
H
H
O
1.00
1.02
1.01
0.95
2.00
1.98
0.95
3.99
2.04
2.99
1.03
1.08
6.17
5.79
10.55
6.34
8.5 8.0 7.5 7.0 6.5 6.0 5.5 5.0 4.5 4.0 3.5 3.0 2.5 2.0 1.5 1.0 0.5 0.0
f1 (ppm)

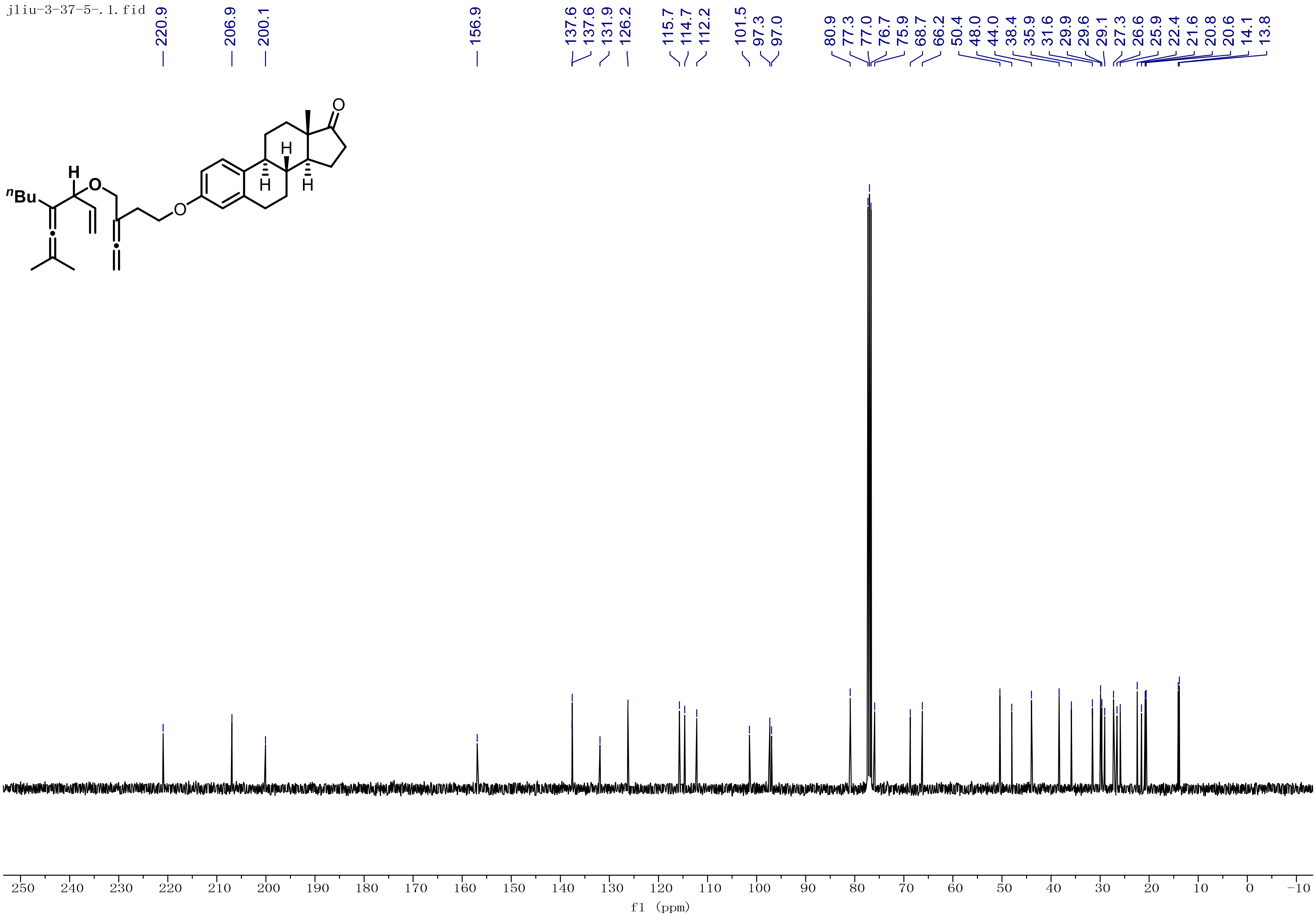
jliu-3-37-5-.1.fid
220.9
206.9
200.1
156.9
137.6
137.6
131.9
126.2
115.7
114.7
112.2
101.5
97.3
97.0
80.9
77.3
77.0
76.7
75.9
68.7
66.2
50.4
48.0
44.0
38.4
35.9
31.6
29.9
29.6
29.1
27.3
26.6
25.9
22.4
21.6
20.8
20.6
14.1
13.8
nBu
H
O
O
H
H
H
O
250 240 230 220 210 200 190 180 170 160 150 140 130 120 110 100 90 80 70 60 50 40 30 20 10 0 -10
f1 (ppm)

jliu-3-51-6.1.fid

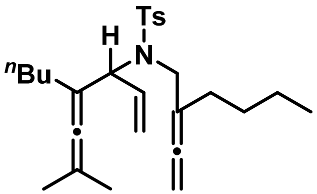

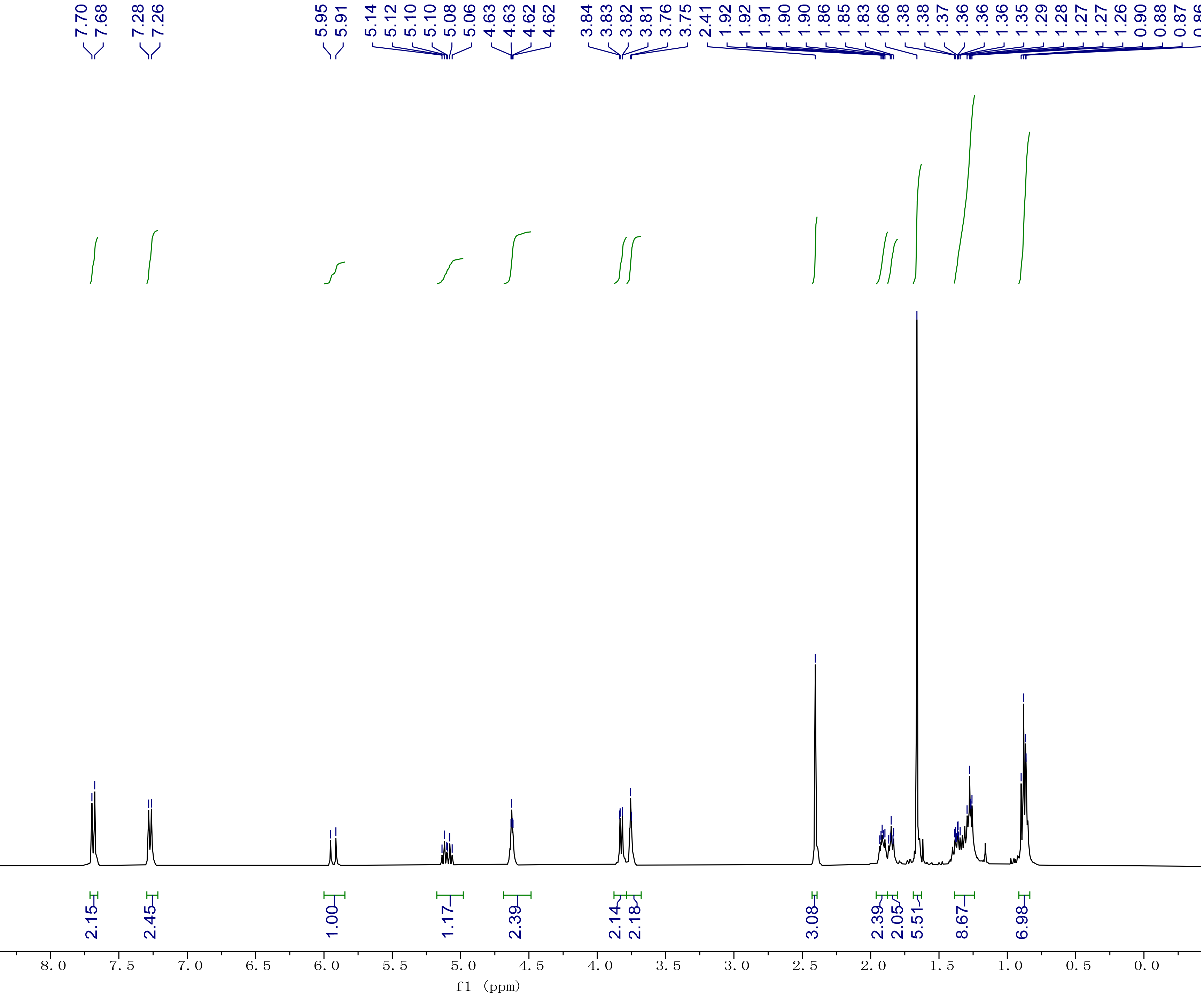

jliu-3-51-6.2.fid

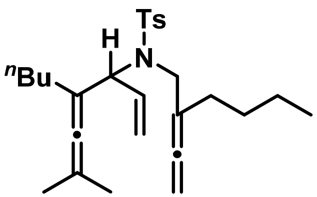

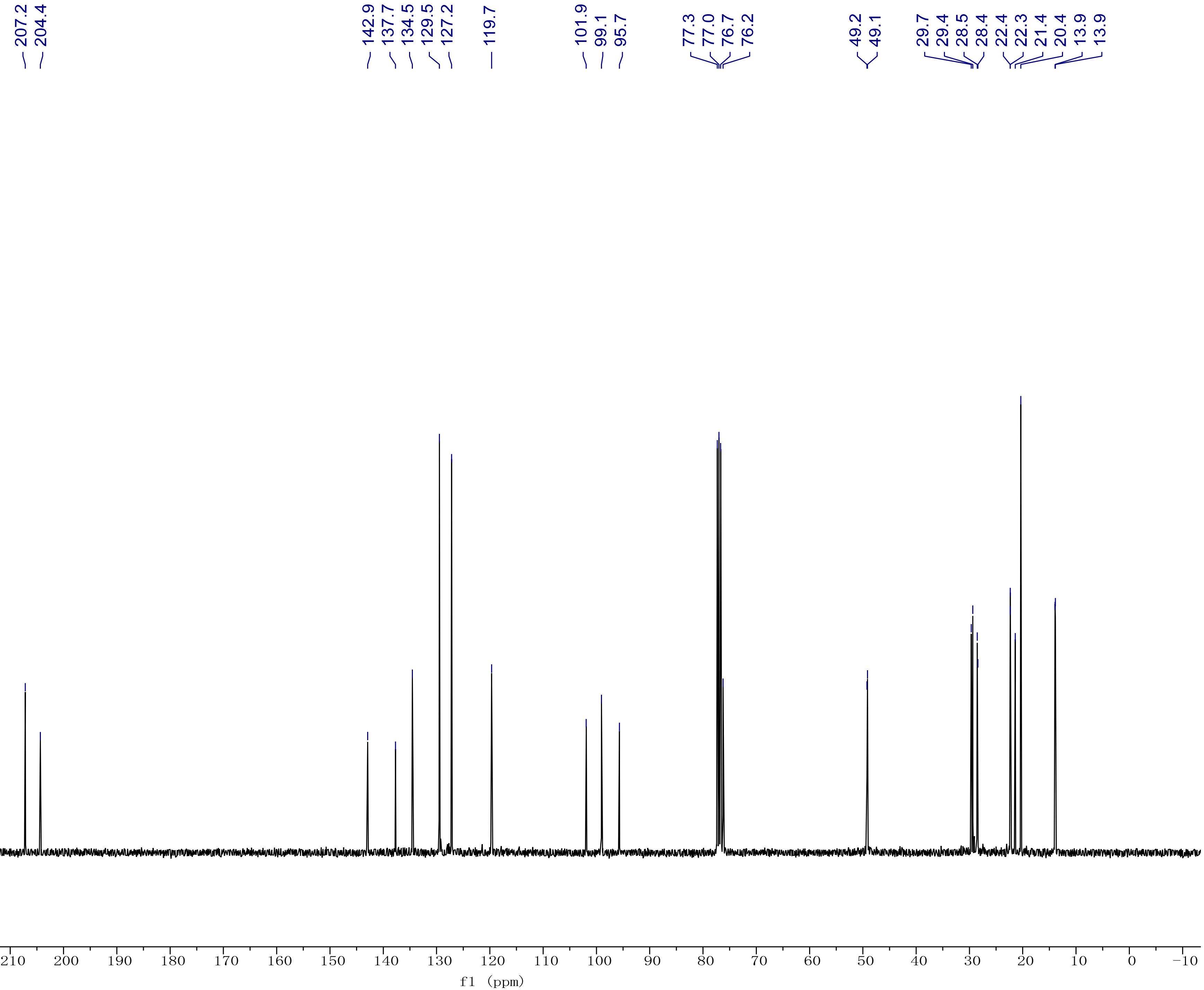

jliu-3-49-3.1.fid

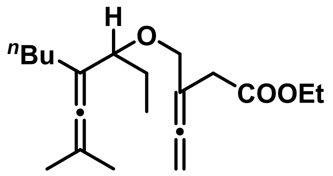

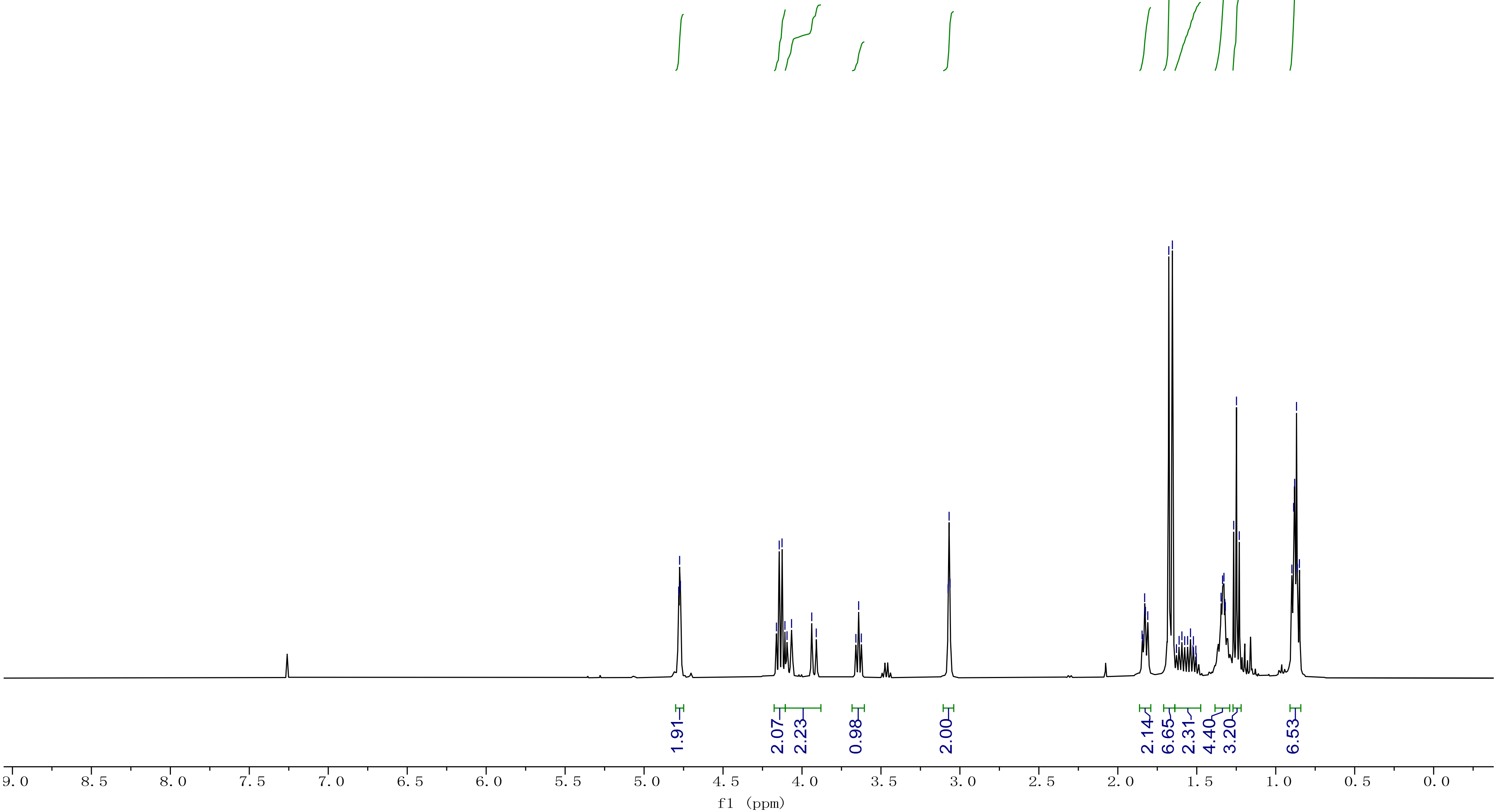

jliu-3-49-3.2.fid

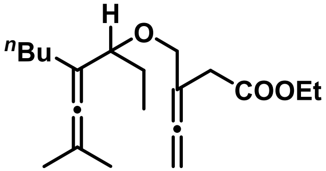

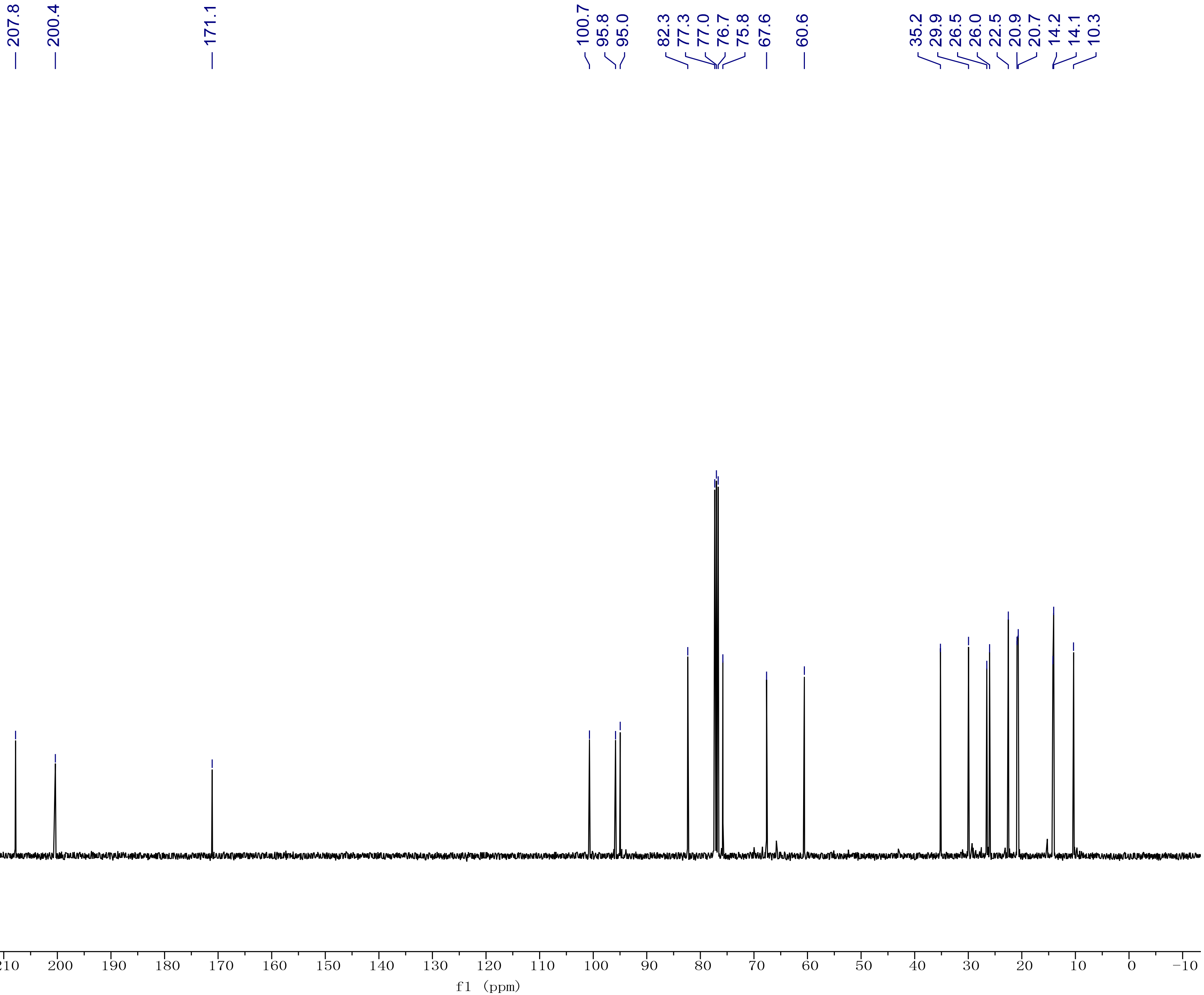

jliu-3-24-3.10.fid

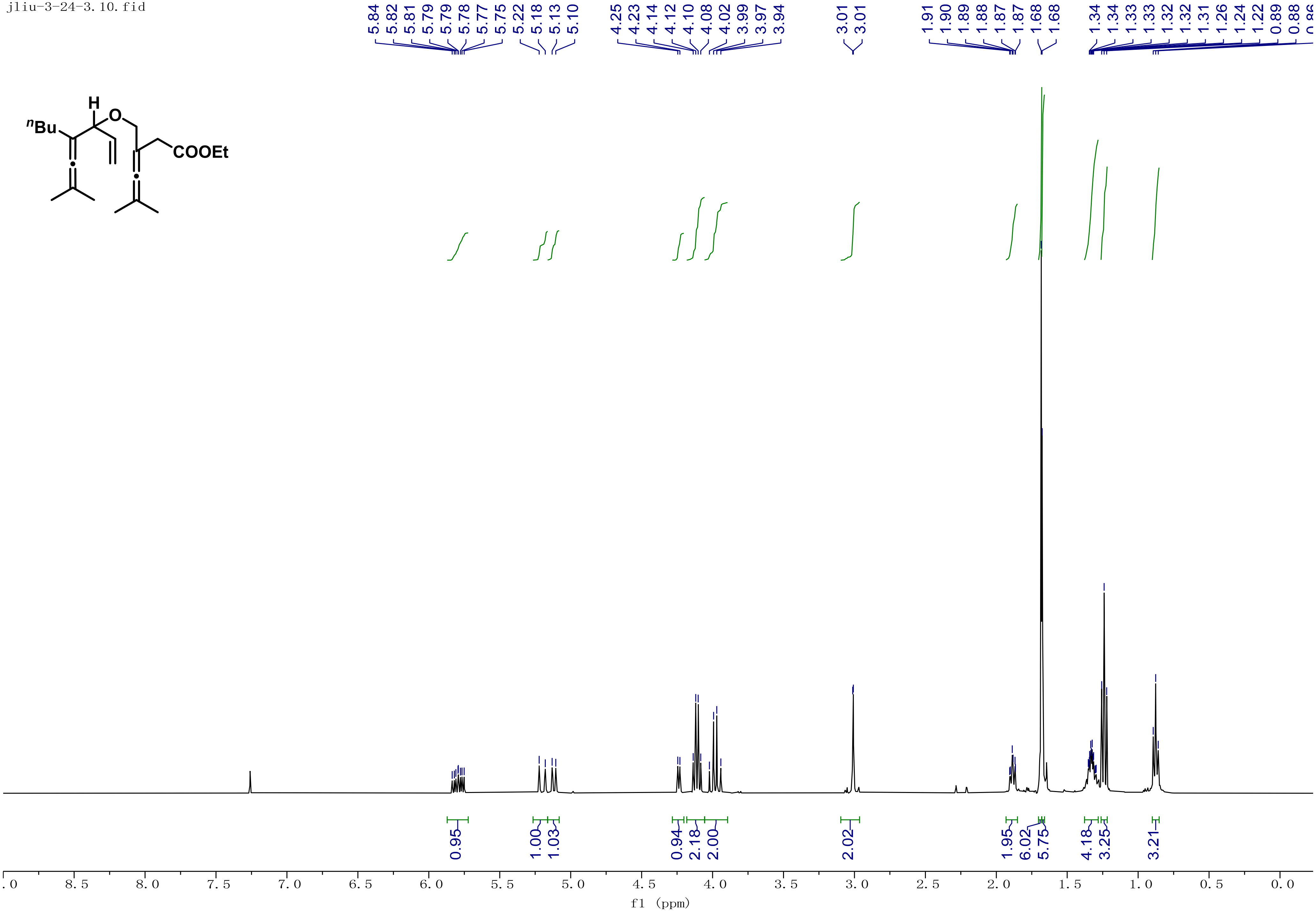

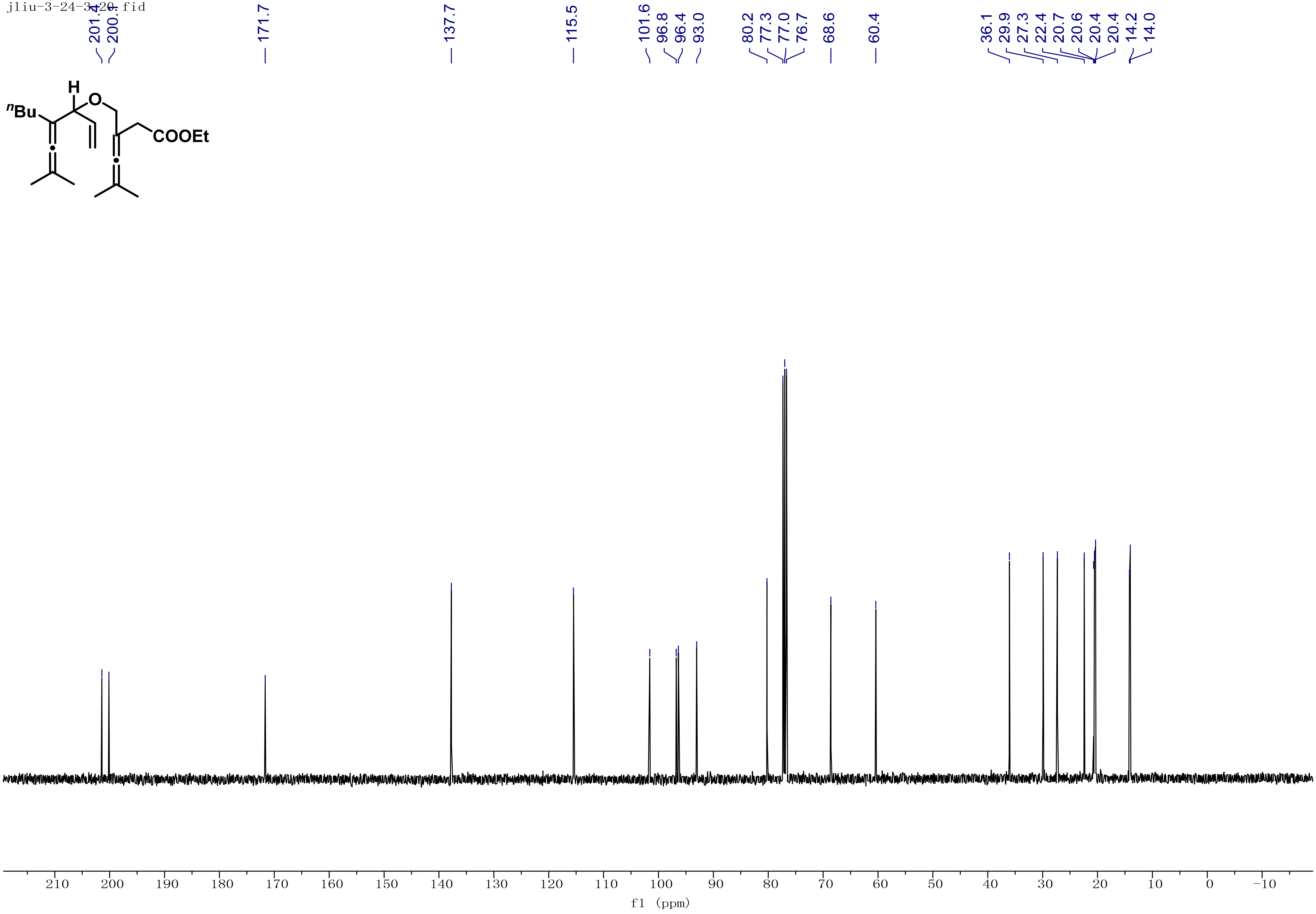

jliu-3-24-3-20.fid
201.4
200.1
171.7
137.7
115.5
101.6
96.8
96.4
93.0
80.2
77.3
77.0
76.7
68.6
60.4
36.1
29.9
27.3
22.4
20.7
20.6
20.4
20.4
14.2
14.0
H
O
nBu
COOEt
210
200
190
180
170
160
150
140
130
120
110
100
90
80
70
60
50
40
30
20
10
0
-10
f1 (ppm)

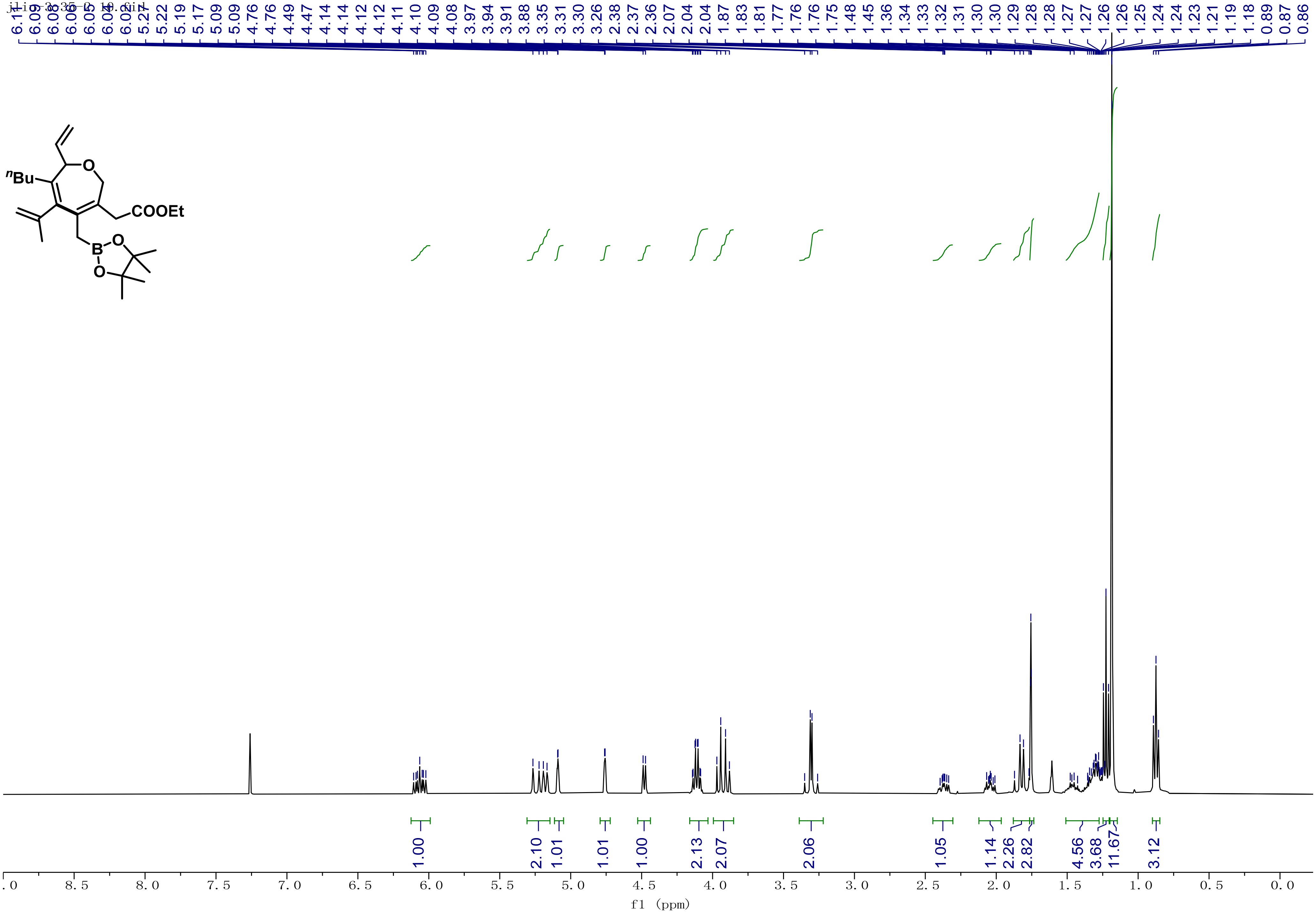

jlin-3-35-2.10.fid
6.11 6.09 6.07 6.06 6.05 6.04 6.03 5.27 5.22 5.19 5.17 5.09 5.09 4.76 4.76 4.49 4.47 4.14 4.14 4.12 4.12 4.11 4.10 4.09 4.08 3.97 3.94 3.91 3.88 3.35 3.31 3.30 3.26 2.38 2.37 2.36 2.07 2.04 2.04 1.87 1.83 1.81 1.77 1.76 1.76 1.75 1.48 1.45 1.36 1.34 1.33 1.32 1.31 1.30 1.30 1.29 1.28 1.28 1.27 1.27 1.26 1.26 1.25 1.24 1.24 1.23 1.21 1.19 1.18 0.89 0.87 0.86
nBu
O
COOEt
B
O
O
1.00
2.10
1.01
1.01
1.00
2.13
2.07
2.06
1.05
1.14
2.26
2.82
4.56
3.68
11.67
3.12
.0 8.5 8.0 7.5 7.0 6.5 6.0 5.5 5.0 4.5 4.0 3.5 3.0 2.5 2.0 1.5 1.0 0.5 0.0
f1 (ppm)

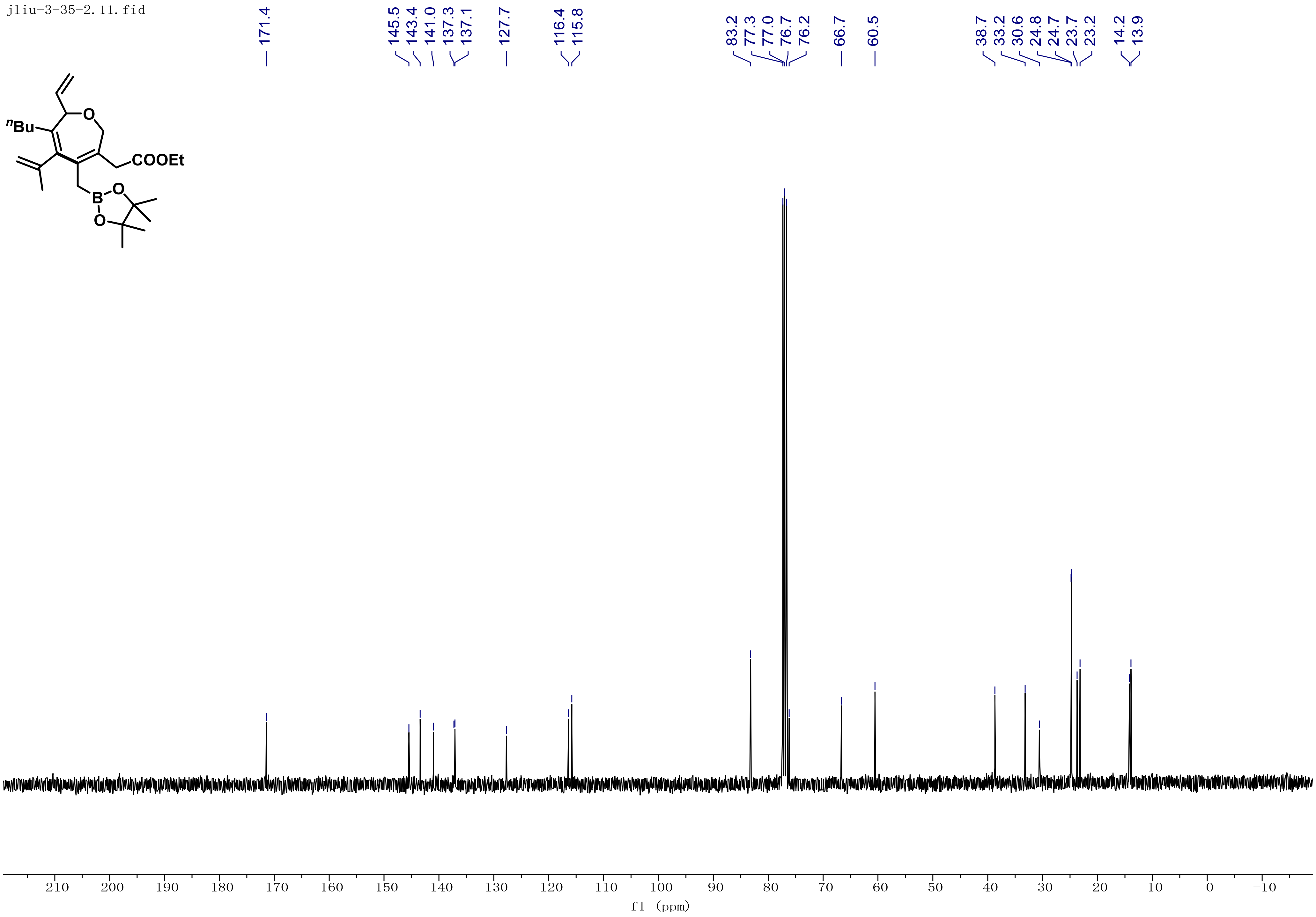
jliu-3-35-2.11.fid
171.4
145.5
143.4
141.0
137.3
137.1
127.7
116.4
115.8
83.2
77.3
77.0
76.7
76.2
66.7
60.5
38.7
33.2
30.6
24.8
24.7
23.7
23.2
14.2
13.9
nBu
O
COOEt
B
O
O
210 200 190 180 170 160 150 140 130 120 110 100 90 80 70 60 50 40 30 20 10 0 -10
f1 (ppm)

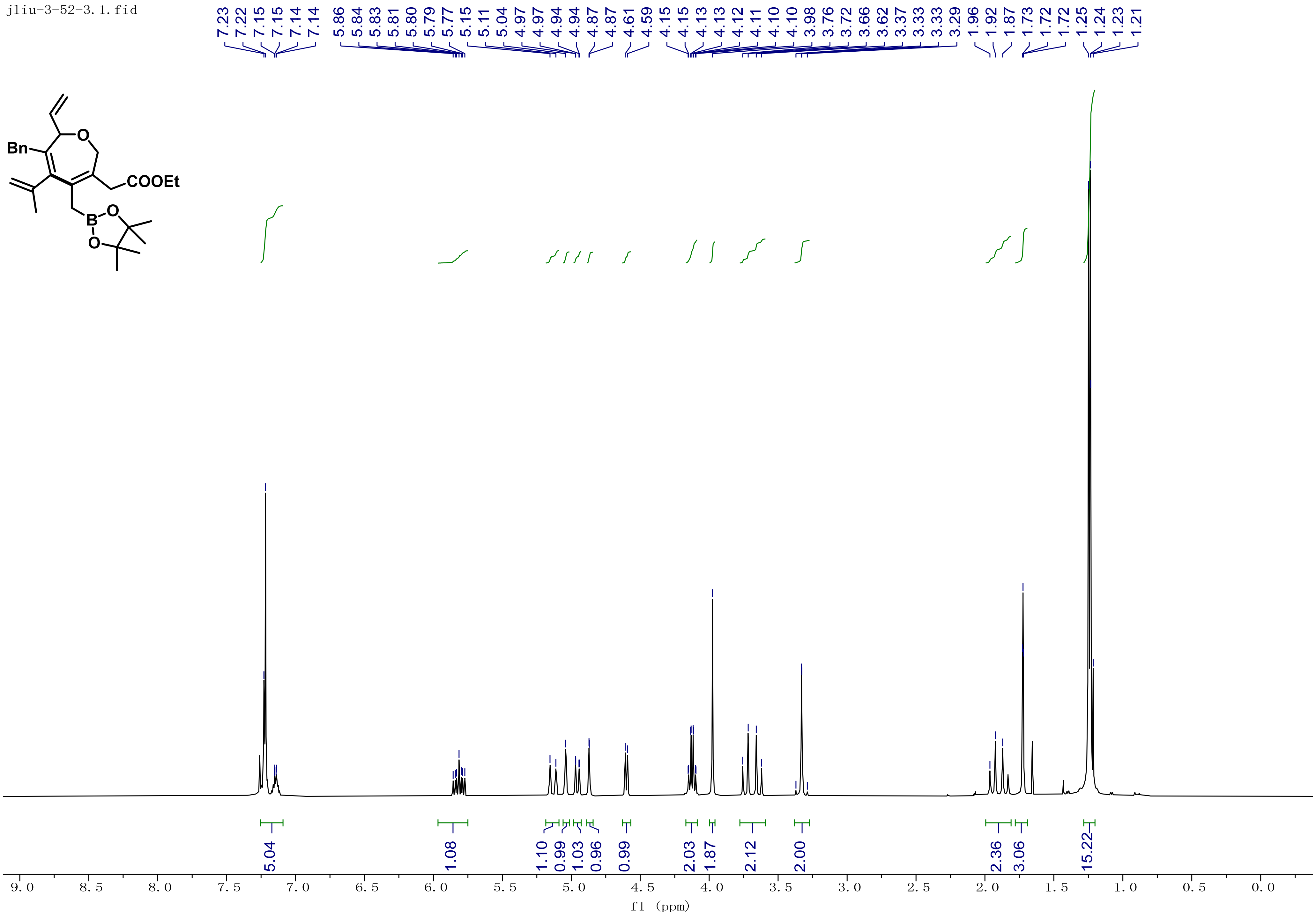
jliu-3-52-3.1.fid
7.23 7.22 7.15 7.15 7.14 7.14 5.86 5.84 5.83 5.81 5.80 5.79 5.77 5.15 5.11 5.04 4.97 4.97 4.94 4.94 4.87 4.87 4.61 4.59 4.15 4.15 4.13 4.13 4.12 4.11 4.10 4.10 3.98 3.76 3.72 3.66 3.62 3.37 3.33 3.33 3.29 1.96 1.92 1.87 1.73 1.72 1.72 1.25 1.24 1.23 1.21
Bn
O
COOEt
B
O
O
5.04 1.08 1.10 0.99 1.03 0.96 0.99 2.03 1.87 2.12 2.00 2.36 3.06 15.22
9.0 8.5 8.0 7.5 7.0 6.5 6.0 5.5 5.0 4.5 4.0 3.5 3.0 2.5 2.0 1.5 1.0 0.5 0.0
f1 (ppm)

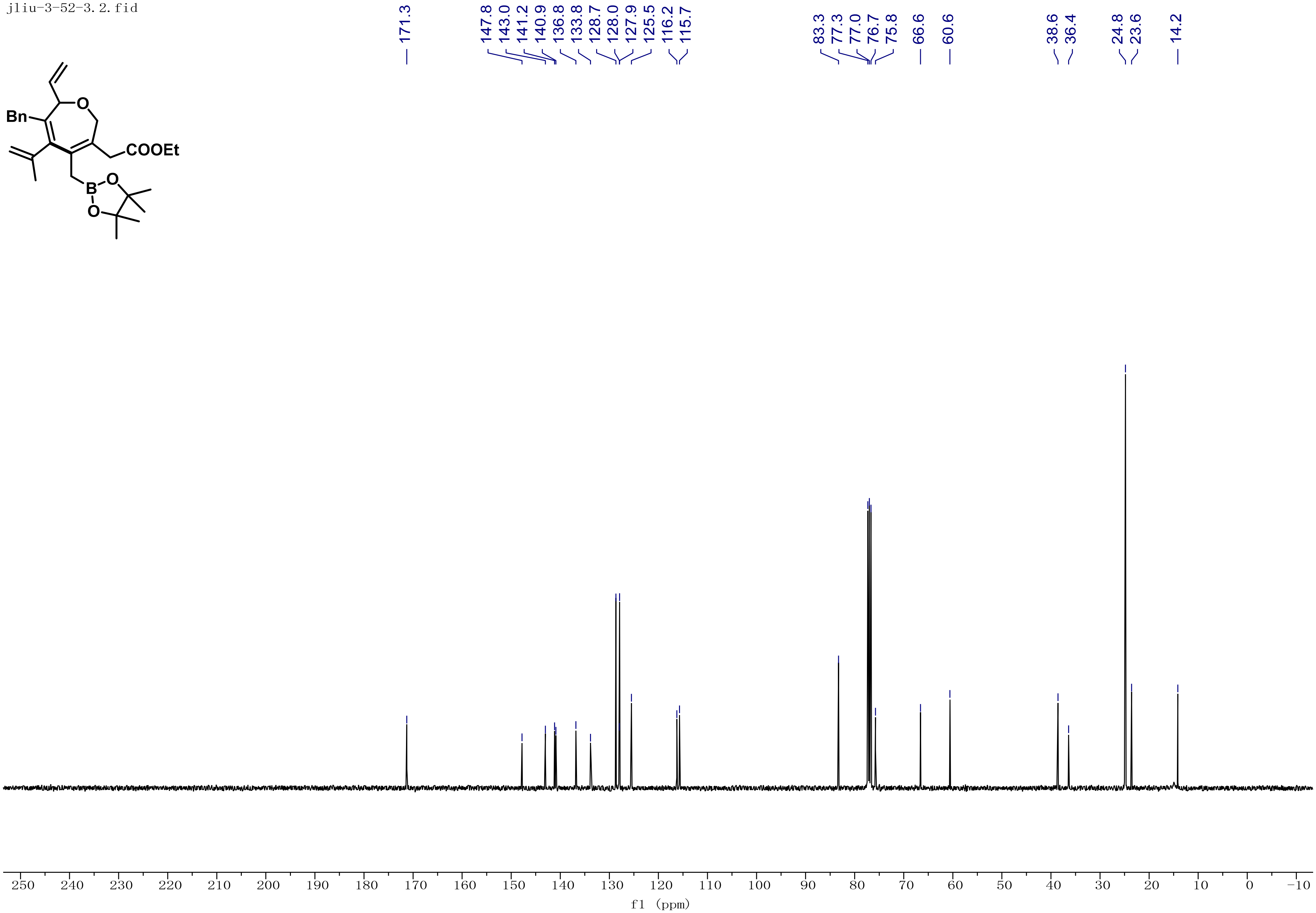
jliu-3-52-3.2.fid
171.3
147.8
143.0
141.2
140.9
136.8
133.8
128.7
128.0
127.9
125.5
116.2
115.7
83.3
77.3
77.0
76.7
75.8
66.6
60.6
38.6
36.4
24.8
23.6
14.2
Bn
O
COOEt
B
O
O
250 240 230 220 210 200 190 180 170 160 150 140 130 120 110 100 90 80 70 60 50 40 30 20 10 0 −10
f1 (ppm)

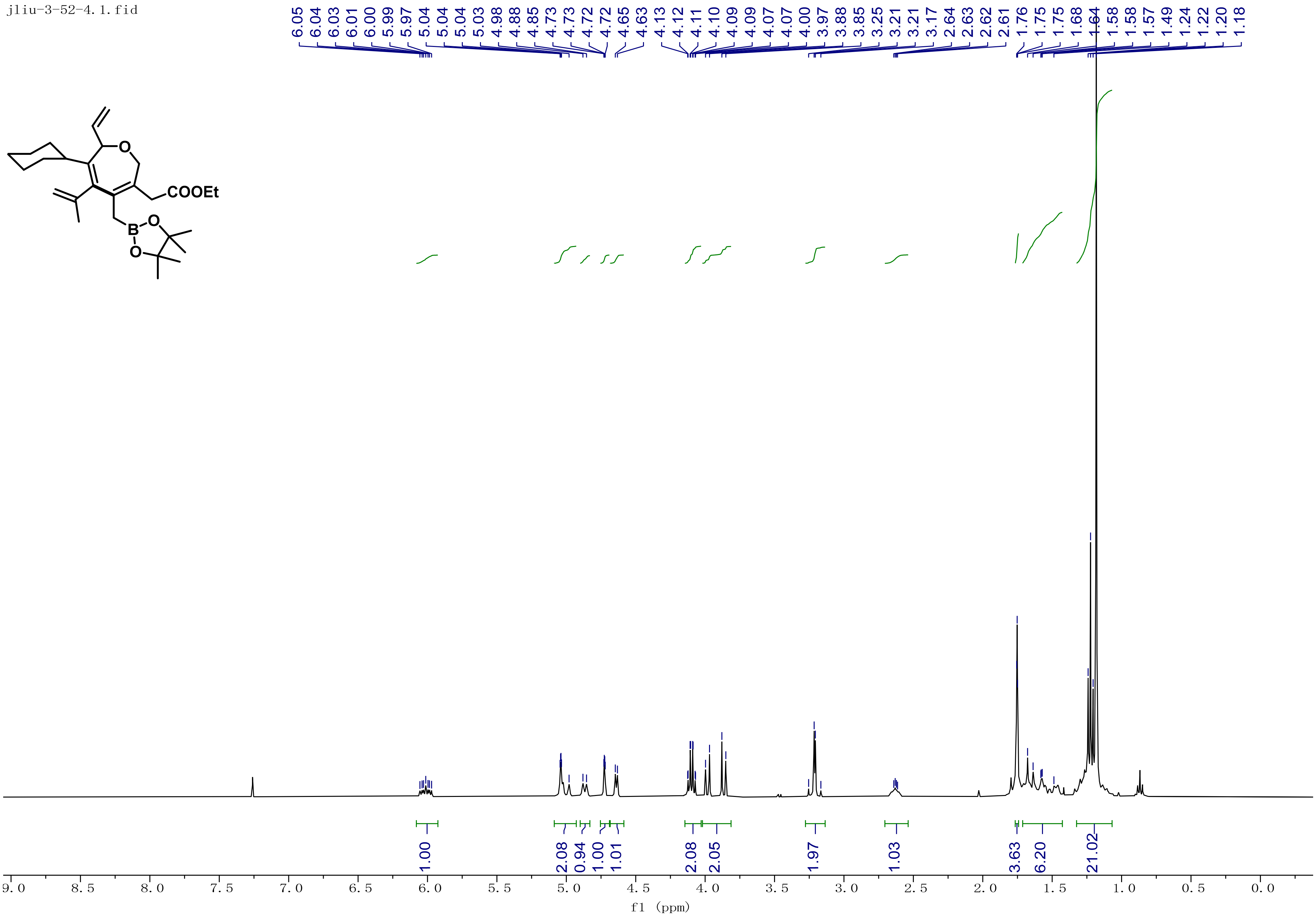
jliu-3-52-4.1.fid
6.05 6.04 6.03 6.01 6.00 5.99 5.97 5.04 5.04 5.04 5.03 4.98 4.88 4.85 4.73 4.73 4.72 4.72 4.65 4.63 4.13 4.12 4.11 4.10 4.09 4.09 4.07 4.07 4.00 3.97 3.88 3.85 3.25 3.21 3.21 3.17 2.64 2.63 2.62 2.61 1.76 1.75 1.75 1.68 1.64 1.58 1.58 1.57 1.49 1.24 1.22 1.20 1.18
O
COOEt
B
O
O
1.00
2.08
0.94
1.00
1.01
2.08
2.05
1.97
1.03
3.63
6.20
21.02
9.0 8.5 8.0 7.5 7.0 6.5 6.0 5.5 5.0 4.5 4.0 3.5 3.0 2.5 2.0 1.5 1.0 0.5 0.0
f1 (ppm)

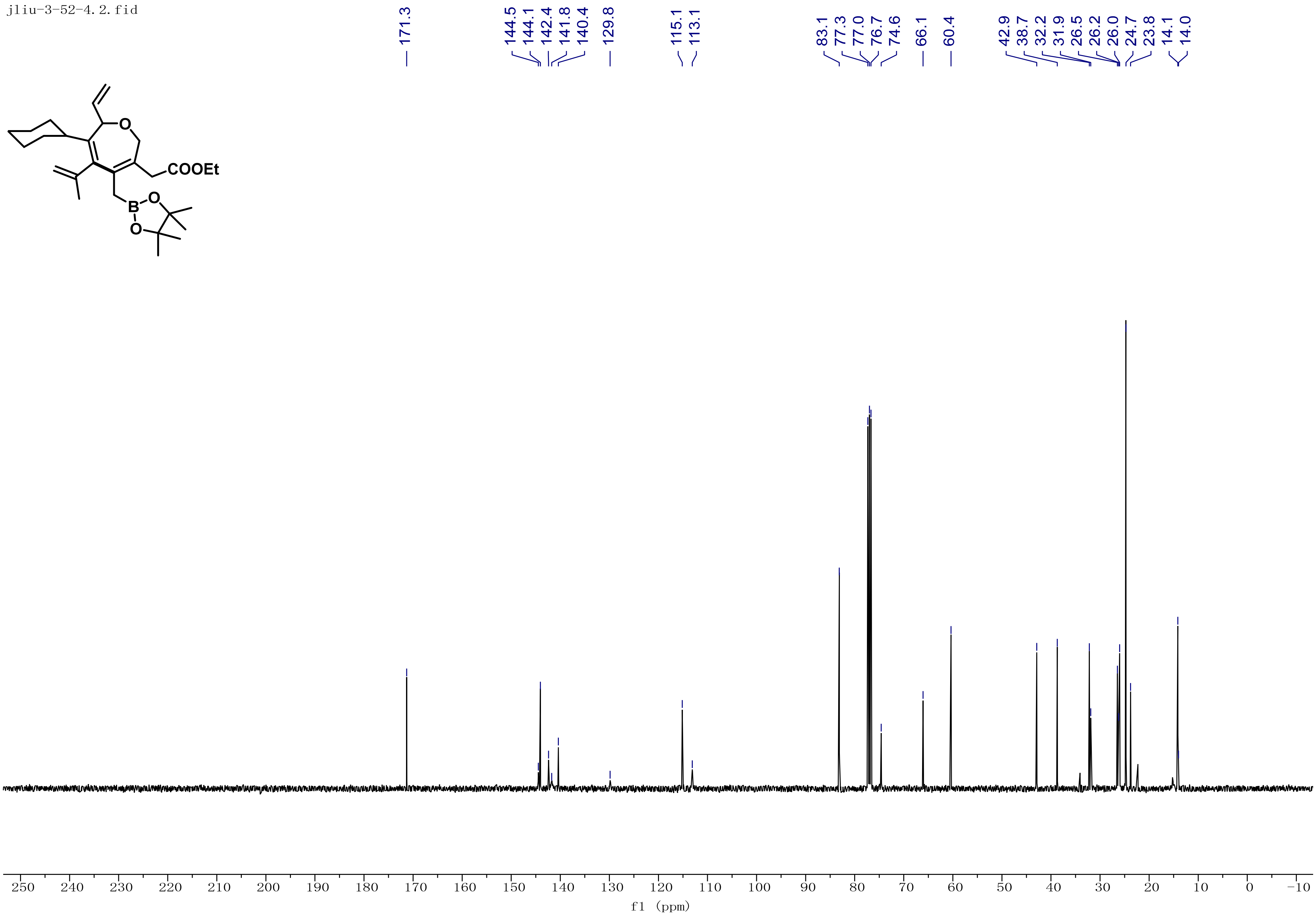
jliu-3-52-4.2.fid
171.3
144.5
144.1
142.4
141.8
140.4
129.8
115.1
113.1
83.1
77.3
77.0
76.7
74.6
66.1
60.4
42.9
38.7
32.2
31.9
26.5
26.2
26.0
24.7
23.8
14.1
14.0
O
COOEt
B
O
O
250 240 230 220 210 200 190 180 170 160 150 140 130 120 110 100 90 80 70 60 50 40 30 20 10 0 -10
f1 (ppm)

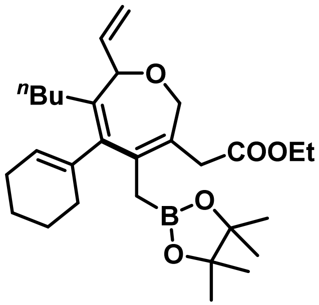
nBu
COOEt
B
O
O

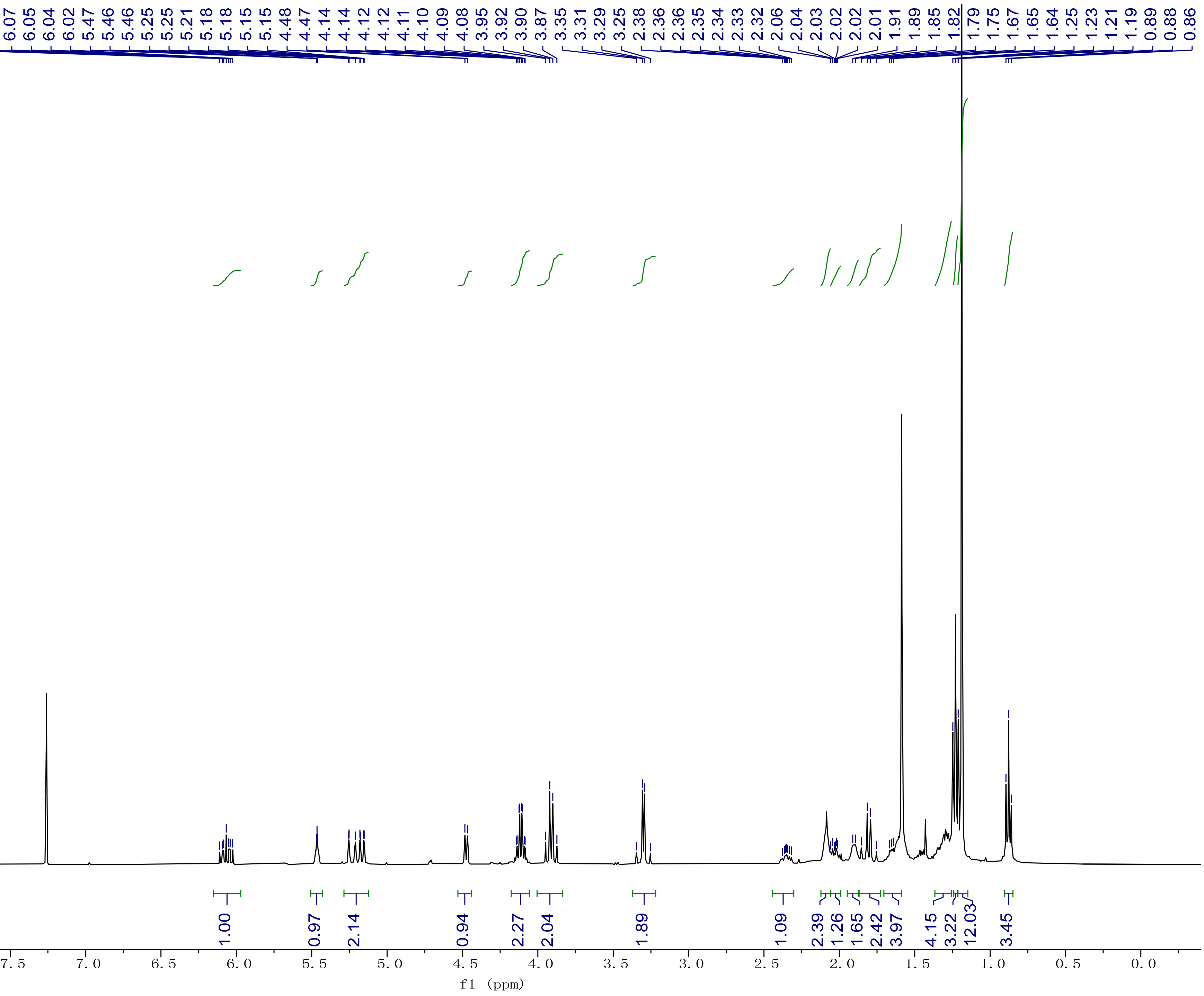
jliu-3-53-6.1.fid
6.11 6.09 6.08 6.07 6.05 6.04 6.02 5.47 5.46 5.46 5.25 5.25 5.21 5.18 5.18 5.15 5.15 4.48 4.47 4.14 4.14 4.12 4.12 4.11 4.10 4.09 4.08 3.95 3.92 3.90 3.87 3.35 3.31 3.29 3.25 2.38 2.36 2.36 2.35 2.34 2.33 2.32 2.06 2.04 2.03 2.02 2.02 2.01 1.91 1.89 1.85 1.82 1.79 1.75 1.67 1.65 1.64 1.25 1.23 1.21 1.19 0.89 0.88 0.86
1.00
0.97
2.14
0.94
2.27
2.04
1.89
1.09
2.39
1.26
1.65
2.42
3.97
4.15
3.22
12.03
3.45
8.5
8.0
7.5
7.0
6.5
6.0
5.5
5.0
4.5
4.0
3.5
3.0
2.5
2.0
1.5
1.0
0.5
0.0
f1 (ppm)

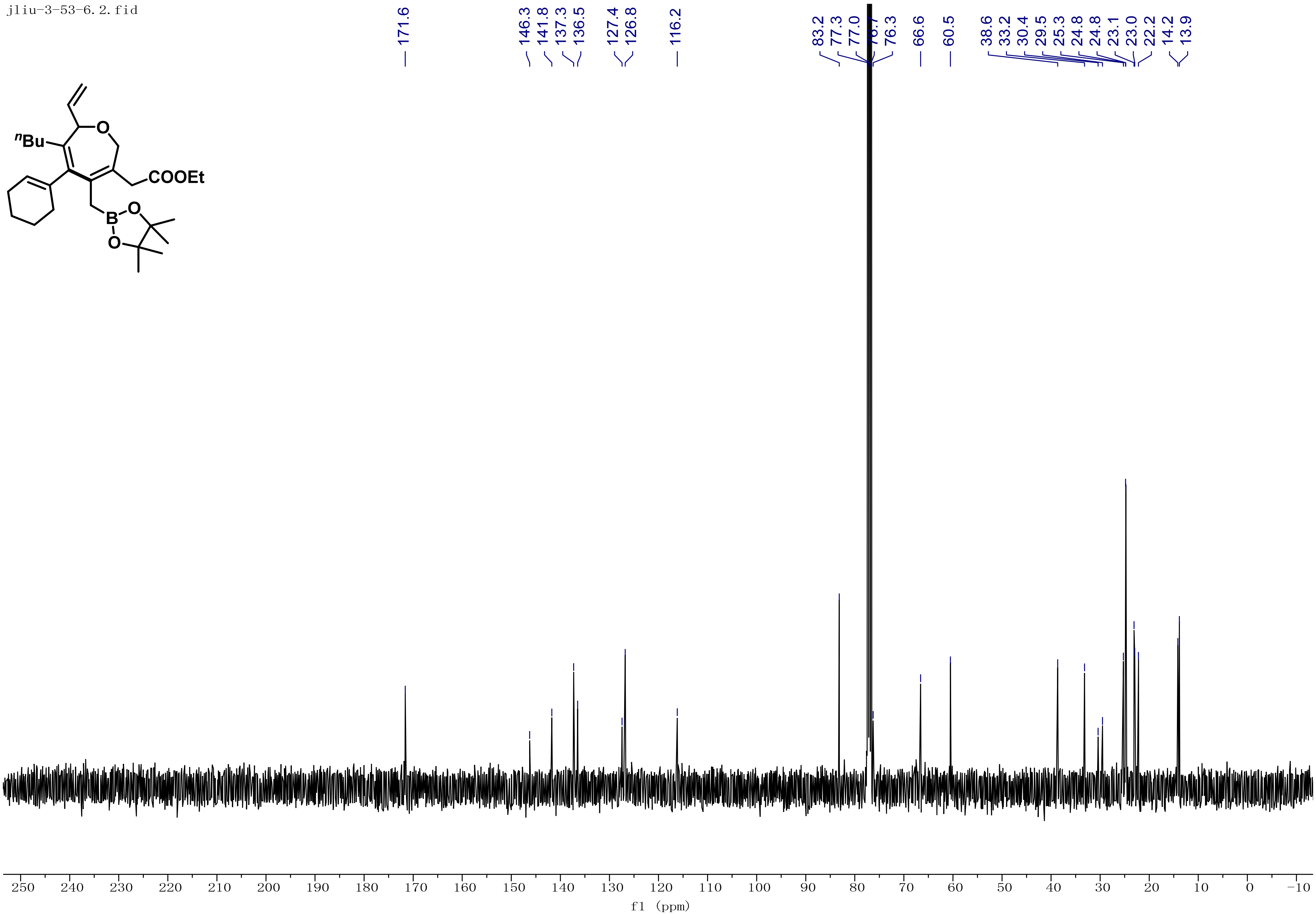
jliu-3-53-6.2.fid
171.6
146.3
141.8
137.3
136.5
127.4
126.8
116.2
83.2
77.3
77.0
76.7
76.3
66.6
60.5
38.6
33.2
30.4
29.5
25.3
24.8
24.8
23.1
23.0
22.2
14.2
13.9
^{n}Bu
O
COOEt
B
O
O
250 240 230 220 210 200 190 180 170 160 150 140 130 120 110 100 90 80 70 60 50 40 30 20 10 0 -10
f1 (ppm)

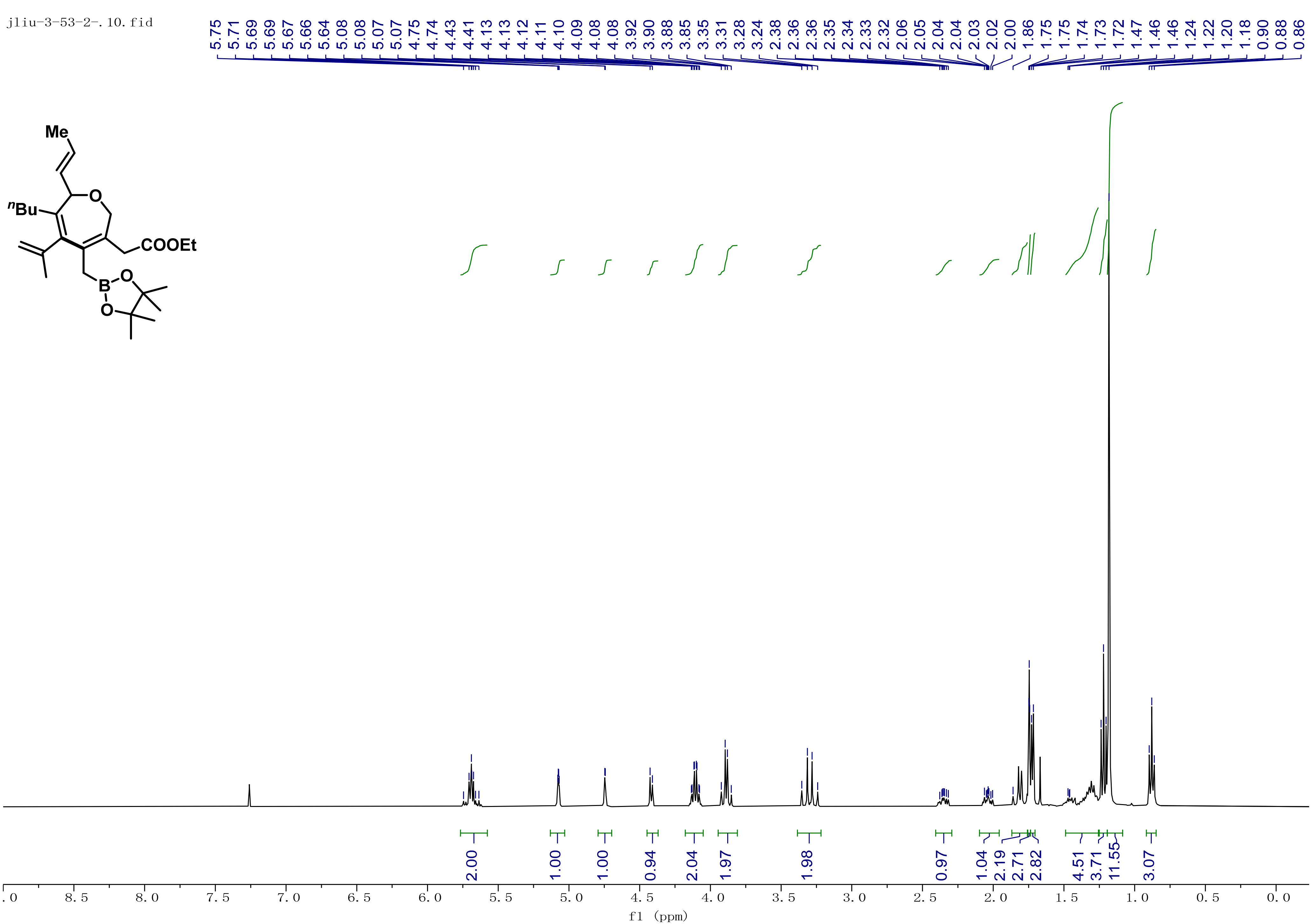
jliu-3-53-2-.10.fid
5.75 5.71 5.69 5.69 5.67 5.66 5.64 5.08 5.08 5.07 5.07 4.75 4.74 4.43 4.41 4.13 4.13 4.12 4.11 4.10 4.09 4.08 4.08 3.92 3.90 3.88 3.85 3.35 3.31 3.28 3.24 2.38 2.36 2.36 2.35 2.34 2.33 2.32 2.06 2.05 2.04 2.04 2.03 2.02 2.00 1.86 1.75 1.75 1.74 1.73 1.72 1.47 1.46 1.46 1.24 1.22 1.20 1.18 0.90 0.88 0.86
Me
nBu
O
COOEt
B
O
O
2.00
1.00
1.00
0.94
2.04
1.97
1.98
0.97
1.04
2.19
2.71
2.82
4.51
3.71
11.55
3.07
.0 8.5 8.0 7.5 7.0 6.5 6.0 5.5 5.0 4.5 4.0 3.5 3.0 2.5 2.0 1.5 1.0 0.5 0.0
f1 (ppm)

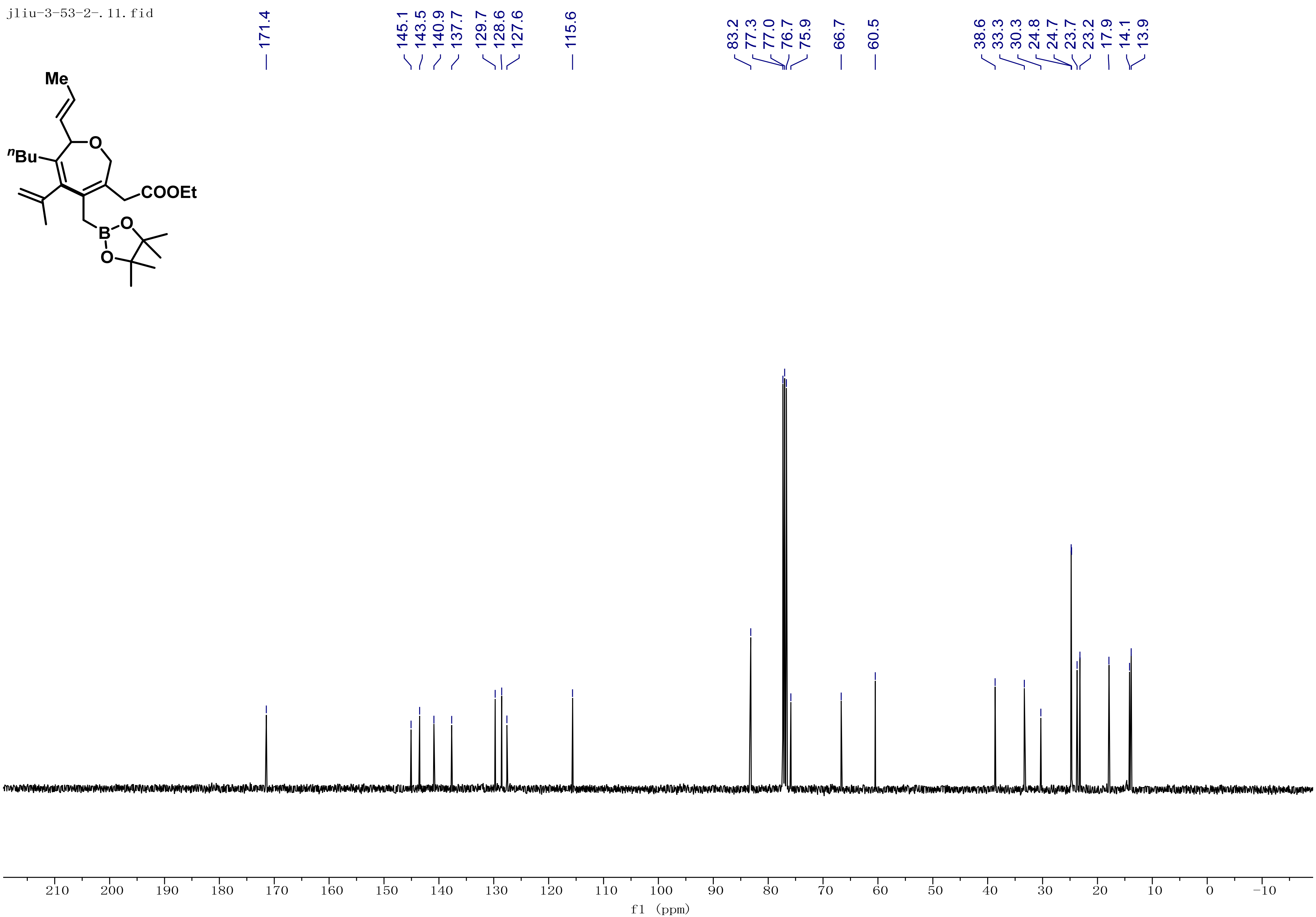
jliu-3-53-2-.11.fid
171.4
145.1
143.5
140.9
137.7
129.7
128.6
127.6
115.6
83.2
77.3
77.0
76.7
75.9
66.7
60.5
38.6
33.3
30.3
24.8
24.7
23.7
23.2
17.9
14.1
13.9
Me
^{n}Bu
O
COOEt
B
O
O
210 200 190 180 170 160 150 140 130 120 110 100 90 80 70 60 50 40 30 20 10 0 -10
f1 (ppm)

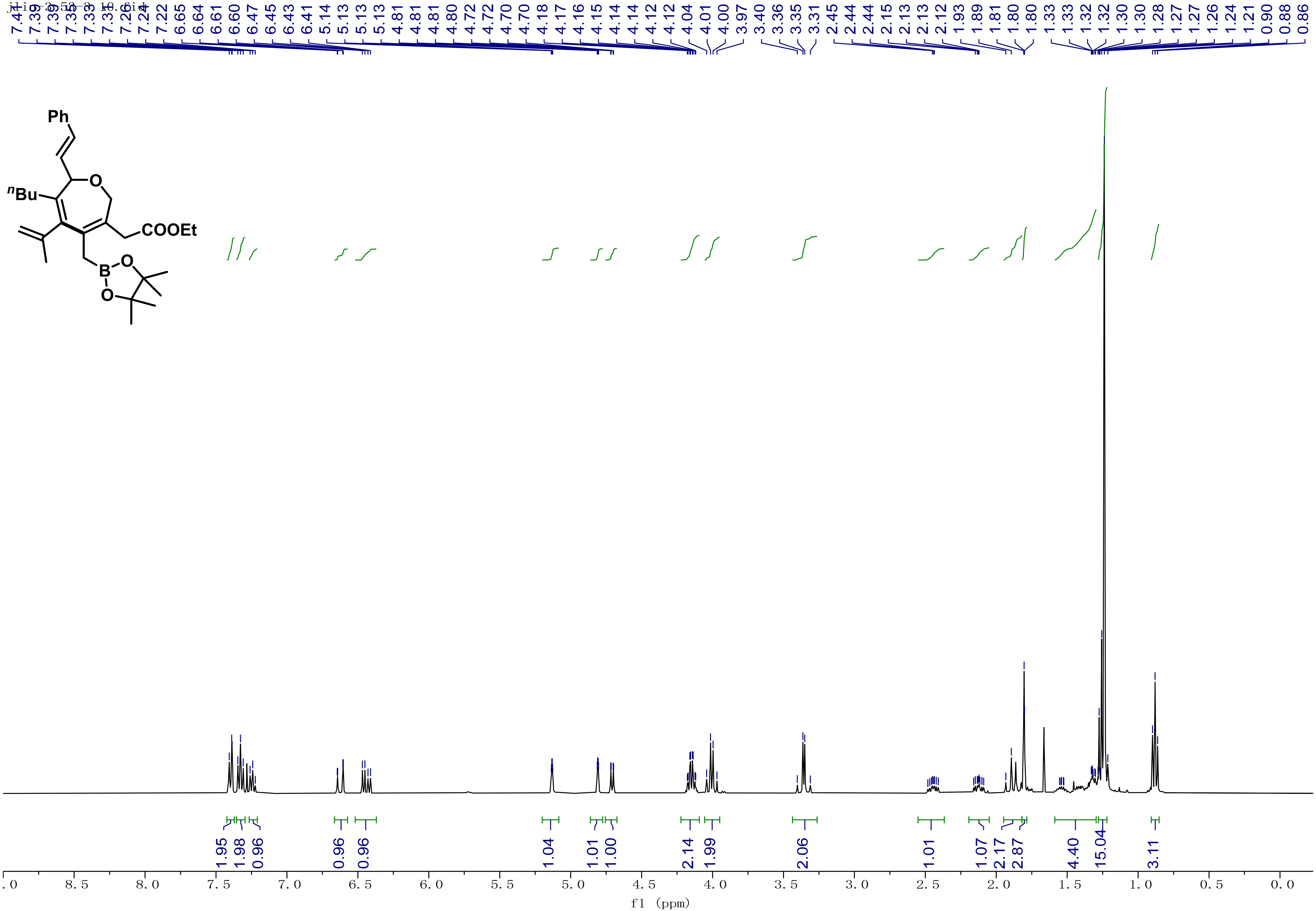
7.41
7.39
7.35
7.33
7.33
7.31
7.26
7.24
7.22
6.65
6.64
6.61
6.60
6.47
6.45
6.43
6.41
5.14
5.13
5.13
5.13
4.81
4.81
4.81
4.80
4.72
4.72
4.70
4.70
4.18
4.17
4.16
4.15
4.14
4.14
4.12
4.12
4.04
4.01
4.00
3.97
3.40
3.36
3.35
3.31
2.45
2.44
2.44
2.15
2.13
2.13
2.12
1.93
1.89
1.81
1.80
1.80
1.33
1.33
1.32
1.32
1.30
1.30
1.28
1.27
1.27
1.26
1.24
1.21
0.90
0.88
0.86
Ph
nBu
O
COOEt
B
O
O
1.95
1.98
0.96
0.96
0.96
1.04
1.01
1.00
2.14
1.99
2.06
1.01
1.07
2.17
2.87
4.40
15.04
3.11
8.5
8.0
7.5
7.0
6.5
6.0
5.5
5.0
4.5
4.0
3.5
3.0
2.5
2.0
1.5
1.0
0.5
0.0
f1 (ppm)

jliu-3-53-3.11.fid

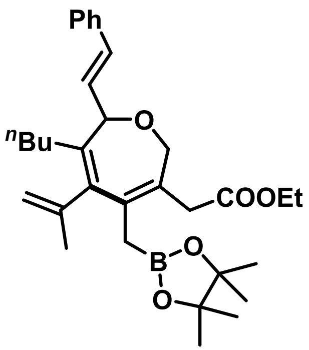

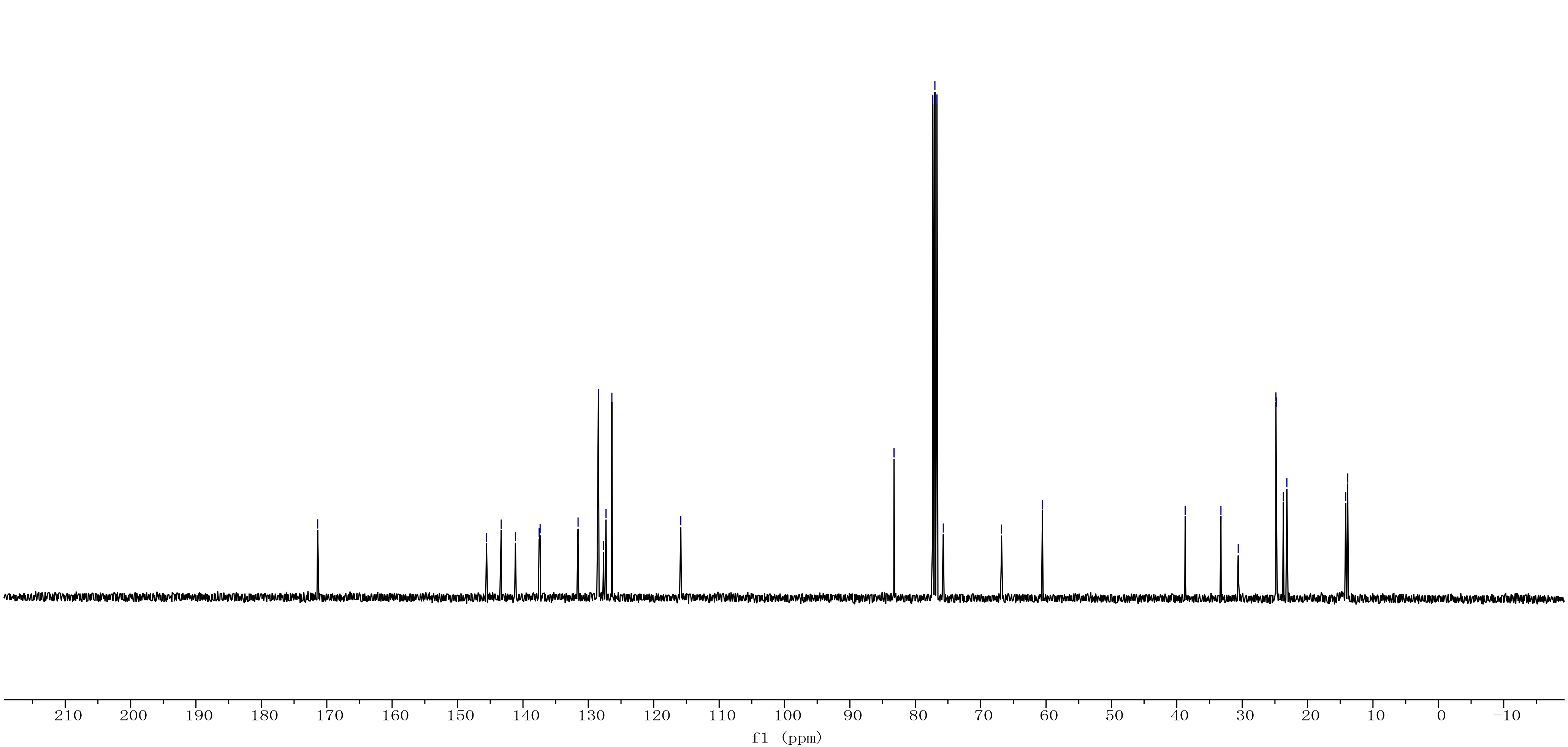

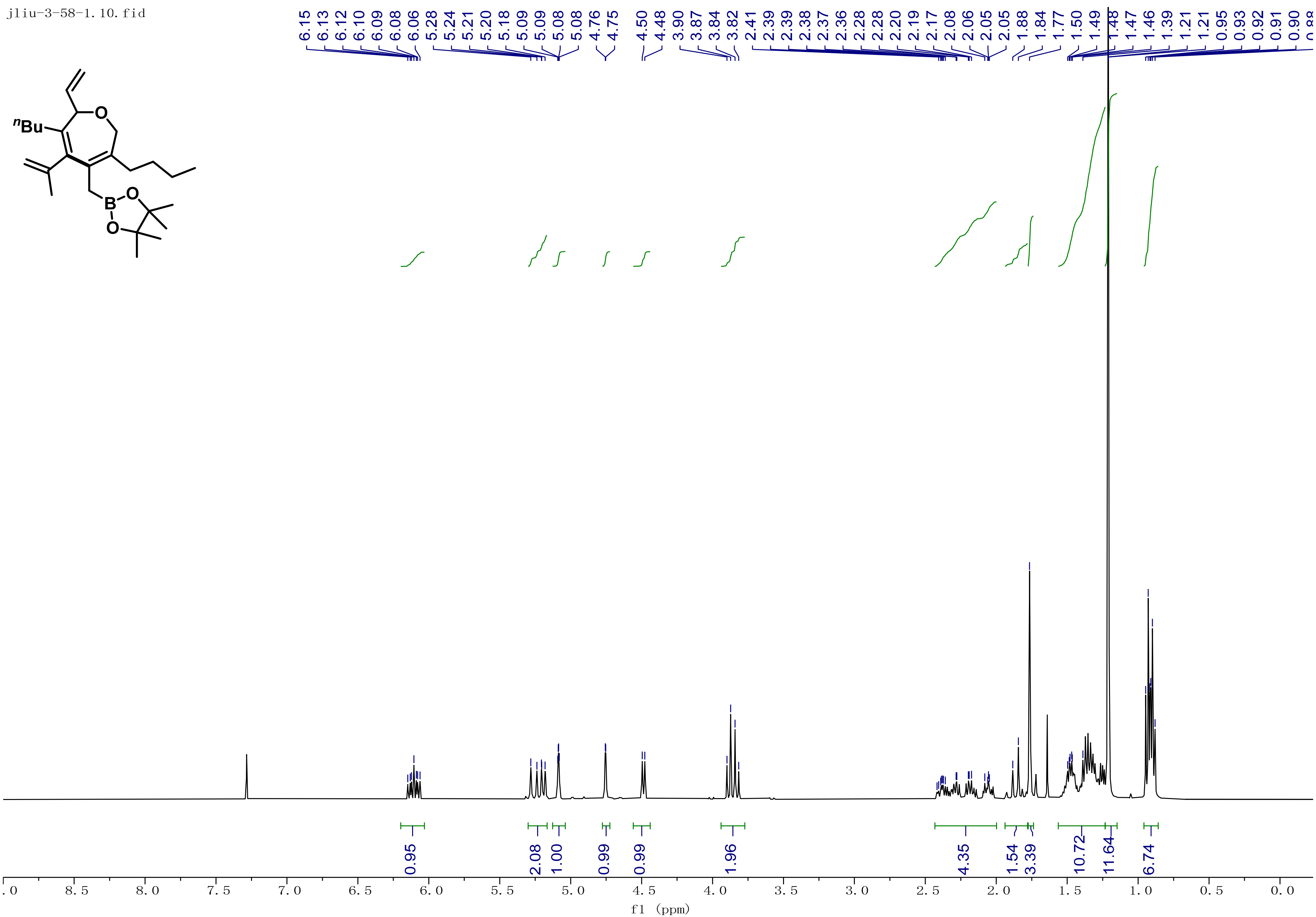

jliu-3-58-1.10.fid
6.15 6.13 6.12 6.10 6.09 6.08 6.06 5.28 5.24 5.21 5.20 5.18 5.09 5.09 5.08 5.08 4.76 4.75 4.50 4.48 3.90 3.87 3.84 3.82 2.41 2.39 2.39 2.38 2.37 2.36 2.28 2.28 2.20 2.19 2.17 2.08 2.06 2.05 2.05 1.88 1.84 1.77 1.50 1.49 1.48 1.47 1.46 1.39 1.21 1.21 0.95 0.93 0.92 0.91 0.90
O
^{n}Bu
B
O
O
0.95
2.08
1.00
0.99
0.99
1.96
4.35
1.54
3.39
10.72
11.64
6.74
8.5 8.0 7.5 7.0 6.5 6.0 5.5 5.0 4.5 4.0 3.5 3.0 2.5 2.0 1.5 1.0 0.5 0.0
f1 (ppm)

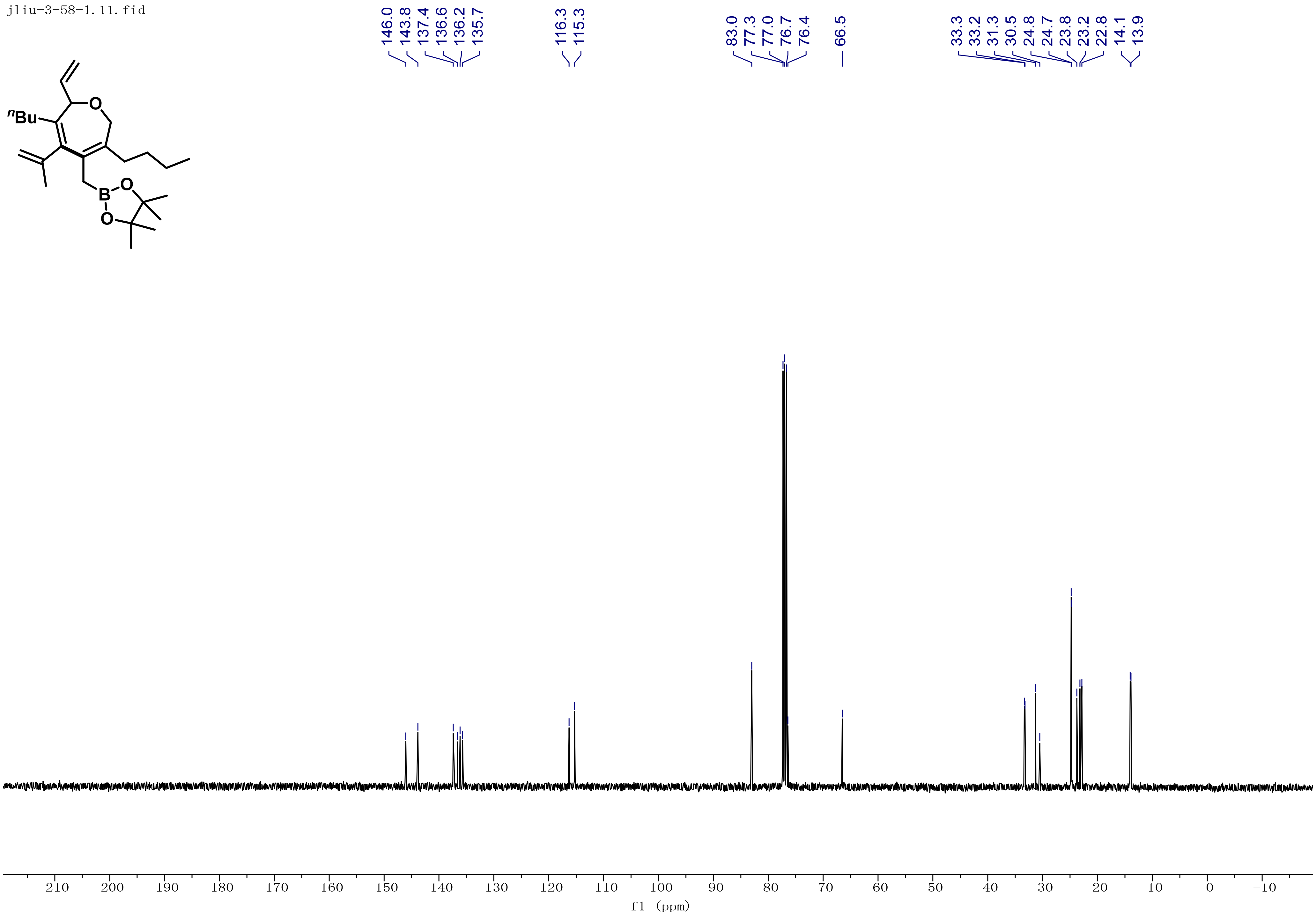

jliu-3-58-1.11.fid
146.0
143.8
137.4
136.6
136.2
135.7
116.3
115.3
83.0
77.3
77.0
76.7
76.4
66.5
33.3
33.2
31.3
30.5
24.8
24.7
23.8
23.2
22.8
14.1
13.9
ⁿBu
O
B
O
O
210 200 190 180 170 160 150 140 130 120 110 100 90 80 70 60 50 40 30 20 10 0 -10
f1 (ppm)

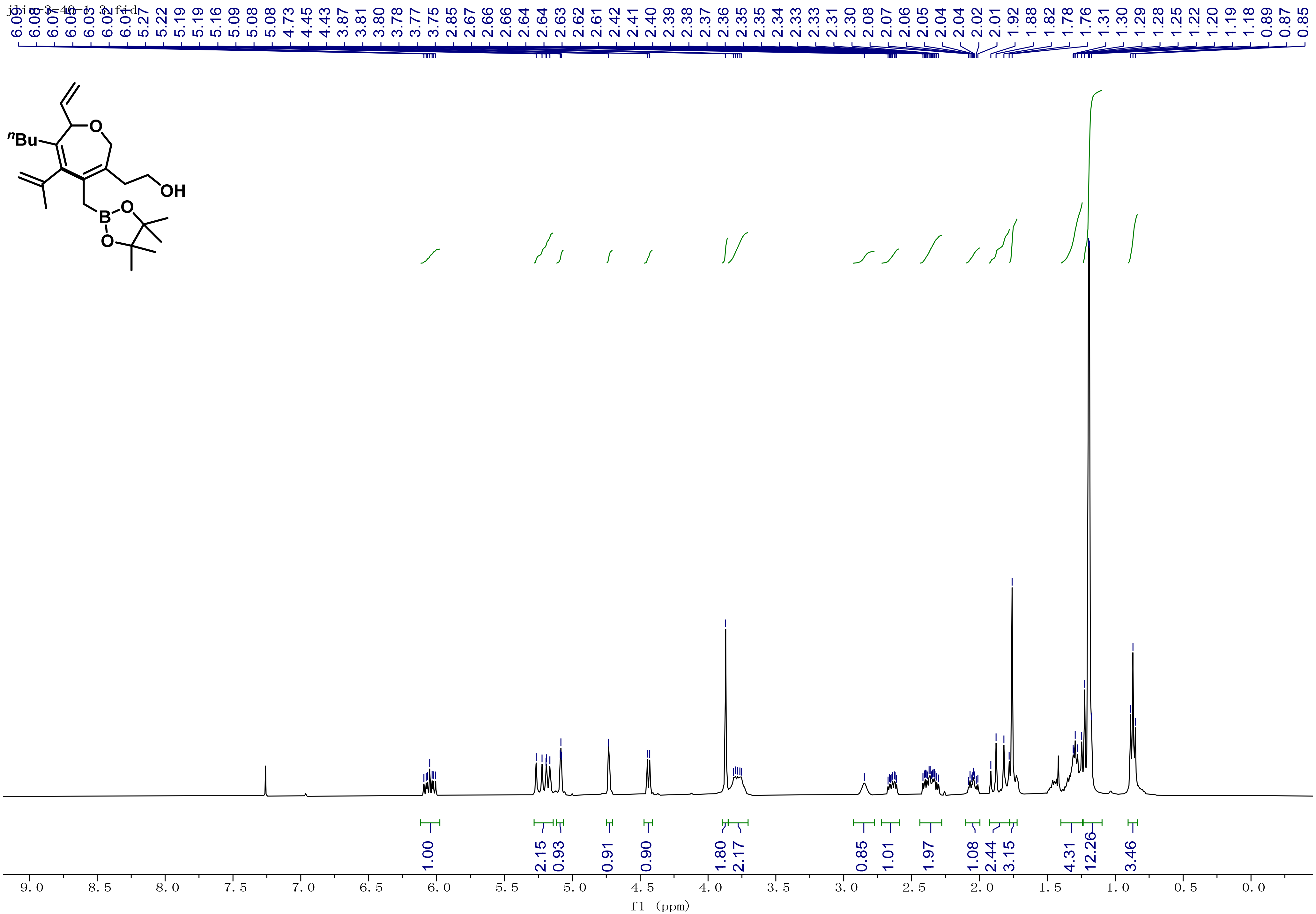
jbin-3-40-1.3.fid
6.09 6.08 6.07 6.05 6.03 6.02 6.01 5.27 5.22 5.19 5.19 5.16 5.09 5.08 5.08 4.73 4.45 4.43 3.87 3.81 3.80 3.78 3.77 3.75 2.85 2.67 2.66 2.66 2.64 2.64 2.63 2.62 2.61 2.42 2.41 2.40 2.39 2.38 2.37 2.36 2.35 2.35 2.34 2.33 2.33 2.31 2.30 2.08 2.07 2.06 2.05 2.04 2.04 2.02 2.01 1.92 1.88 1.82 1.78 1.76 1.31 1.30 1.29 1.28 1.25 1.22 1.20 1.19 1.18 0.89 0.87 0.85
ⁿBu
O
OH
B
O
O
1.00
2.15
0.93
0.91
0.90
1.80
2.17
0.85
1.01
1.97
1.08
2.44
3.15
4.31
12.26
3.46
9.0 8.5 8.0 7.5 7.0 6.5 6.0 5.5 5.0 4.5 4.0 3.5 3.0 2.5 2.0 1.5 1.0 0.5 0.0
f1 (ppm)

jliu-3-40-1-C.1.fid

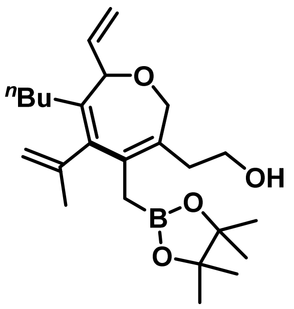

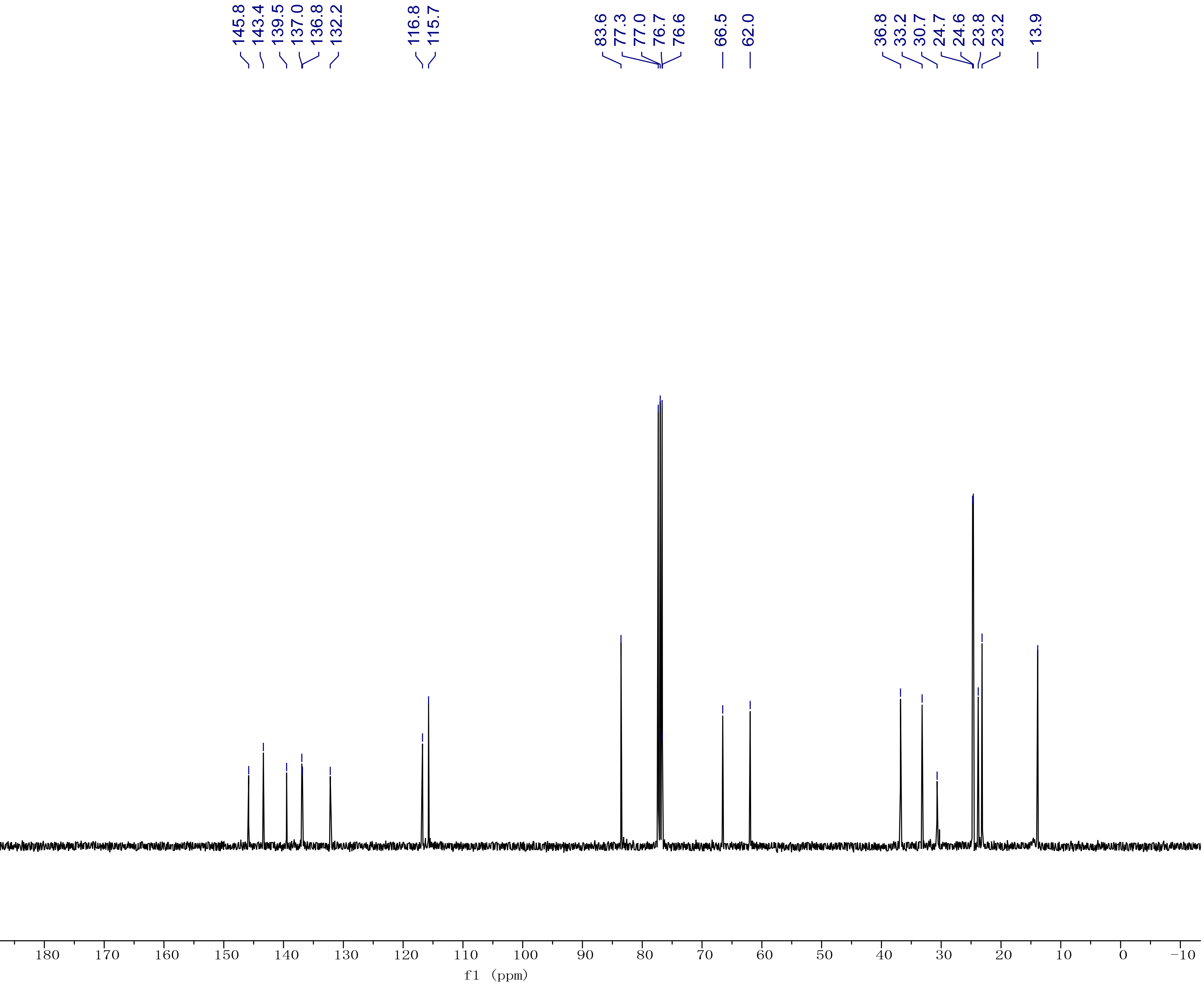

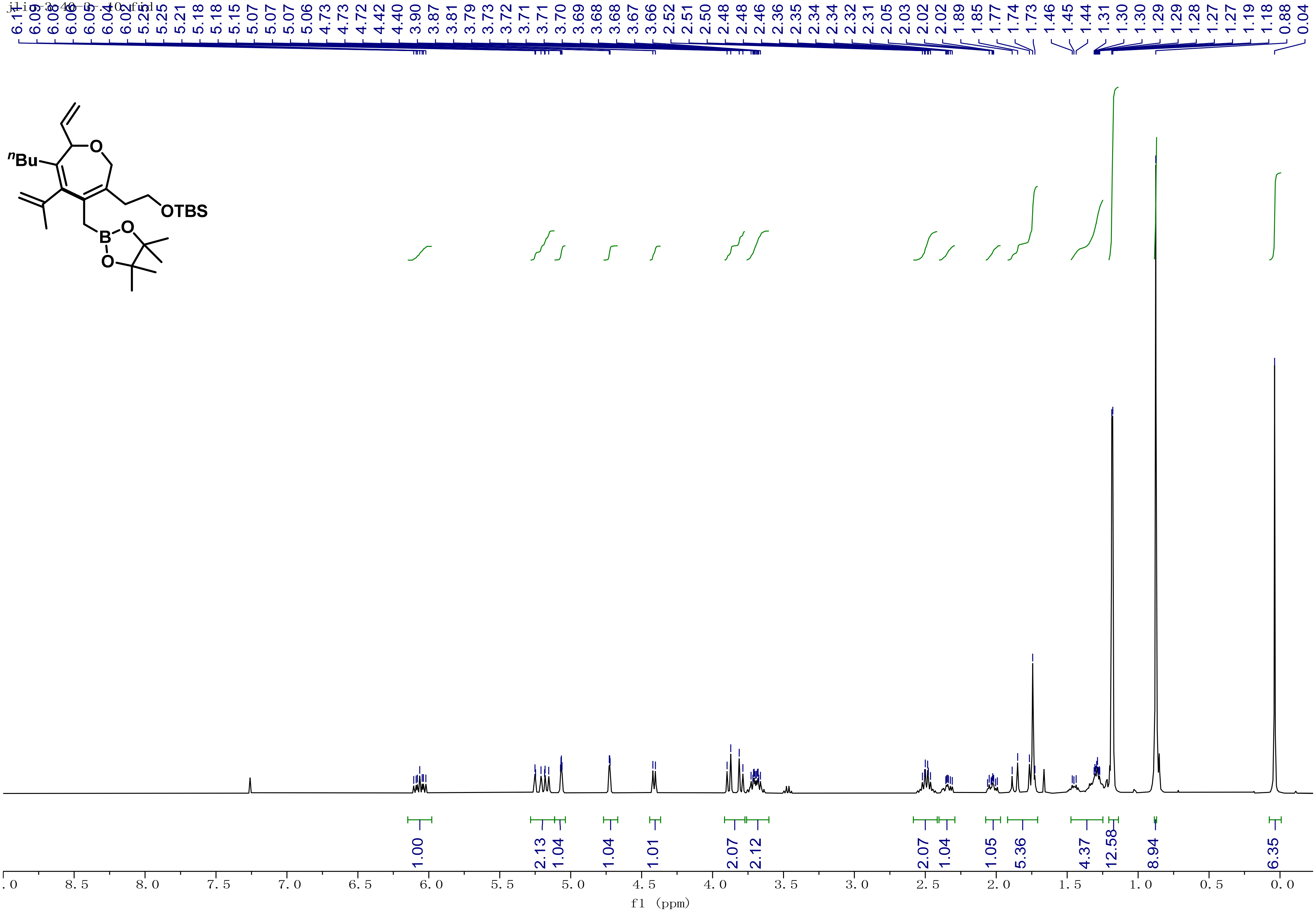

6.11 6.09 6.08 6.06 6.05 6.04 6.02 5.25 5.25 5.21 5.18 5.18 5.15 5.07 5.07 5.07 5.06 4.73 4.73 4.72 4.42 4.40 3.90 3.87 3.81 3.79 3.73 3.72 3.71 3.71 3.70 3.69 3.68 3.68 3.67 3.66 2.52 2.51 2.50 2.48 2.48 2.46 2.36 2.35 2.34 2.34 2.32 2.31 2.05 2.03 2.02 2.02 1.89 1.85 1.77 1.74 1.73 1.46 1.45 1.44 1.31 1.30 1.30 1.29 1.29 1.28 1.27 1.27 1.19 1.18 0.88 0.04
nBu
O
OTBS
B
O
O
1.00 2.13 1.04 1.04 1.01 2.07 2.12 2.07 1.04 1.05 5.36 4.37 12.58 8.94 6.35
8.5 8.0 7.5 7.0 6.5 6.0 5.5 5.0 4.5 4.0 3.5 3.0 2.5 2.0 1.5 1.0 0.5 0.0
f1 (ppm)

jliu-3-40-3.2.fid

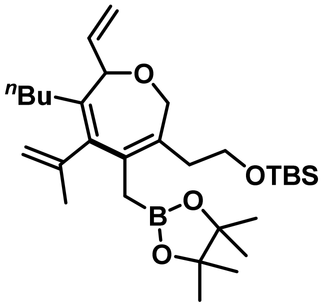

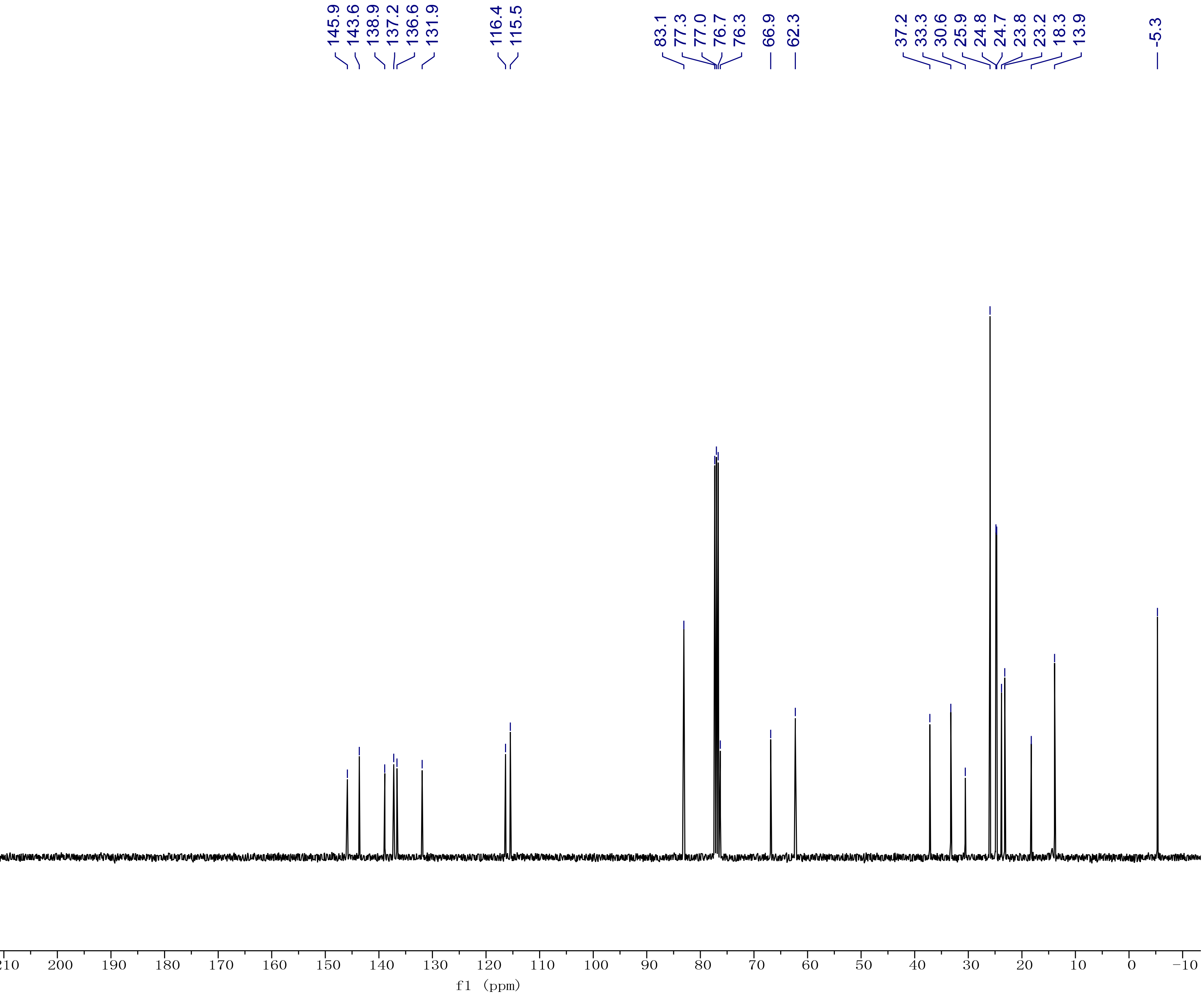

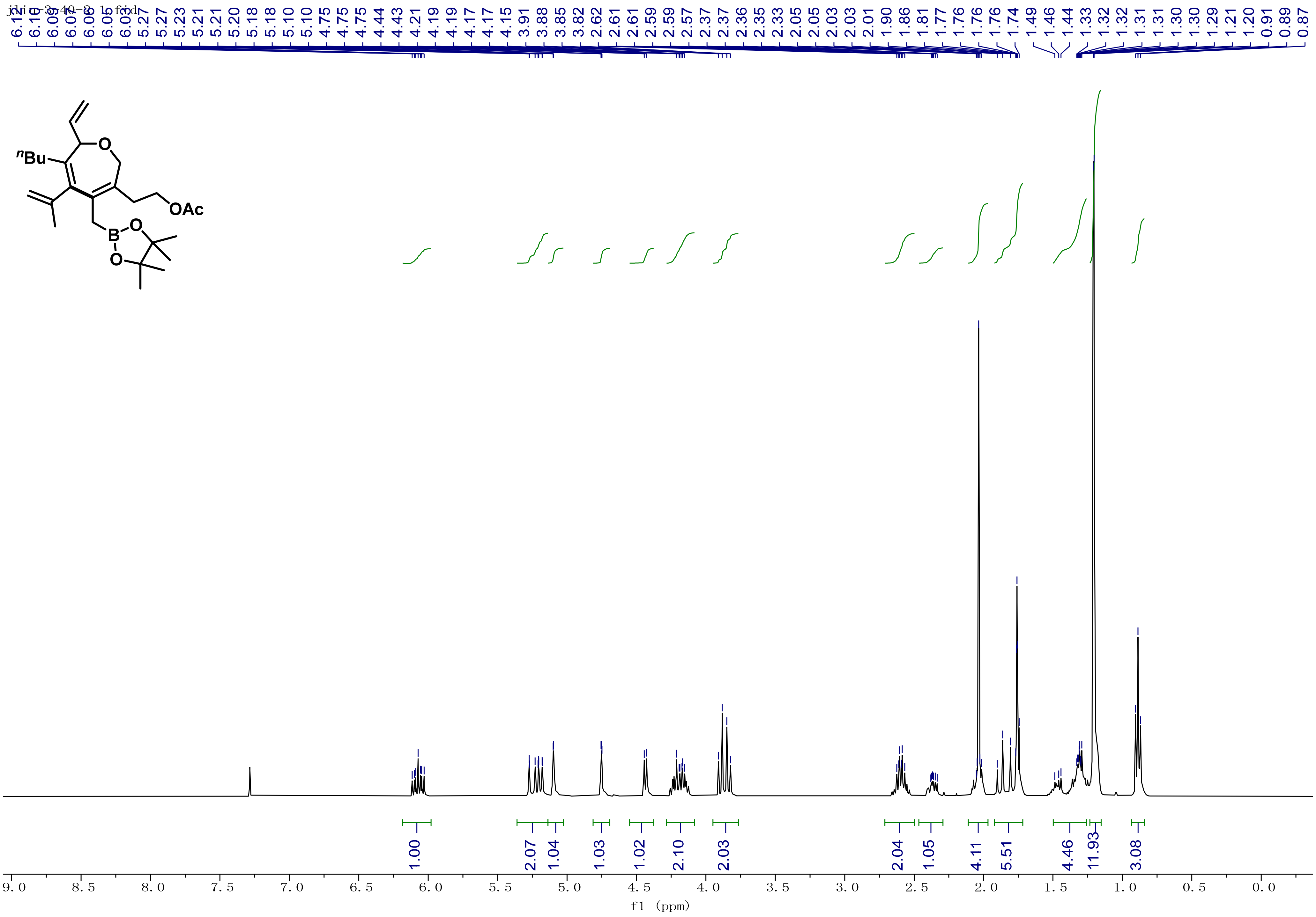
6.11 6.10 6.09 6.07 6.06 6.05 6.03 5.27 5.27 5.23 5.21 5.21 5.20 5.18 5.18 5.10 5.10 4.75 4.75 4.75 4.44 4.43 4.21 4.19 4.19 4.17 4.17 4.15 3.91 3.88 3.85 3.82 2.62 2.61 2.61 2.59 2.59 2.57 2.37 2.37 2.36 2.35 2.33 2.05 2.05 2.03 2.03 2.01 1.90 1.86 1.81 1.77 1.76 1.76 1.76 1.74 1.49 1.46 1.44 1.33 1.32 1.32 1.31 1.31 1.30 1.30 1.29 1.21 1.20 0.91 0.89 0.87
nBu
O
OAc
B
O
O
1.00
2.07
1.04
1.03
1.02
2.10
2.03
2.04
1.05
4.11
5.51
4.46
11.93
3.08
9.0 8.5 8.0 7.5 7.0 6.5 6.0 5.5 5.0 4.5 4.0 3.5 3.0 2.5 2.0 1.5 1.0 0.5 0.0
f1 (ppm)

jliu-3-40-2.2.fid

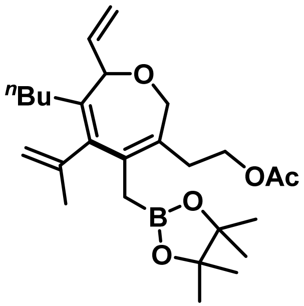

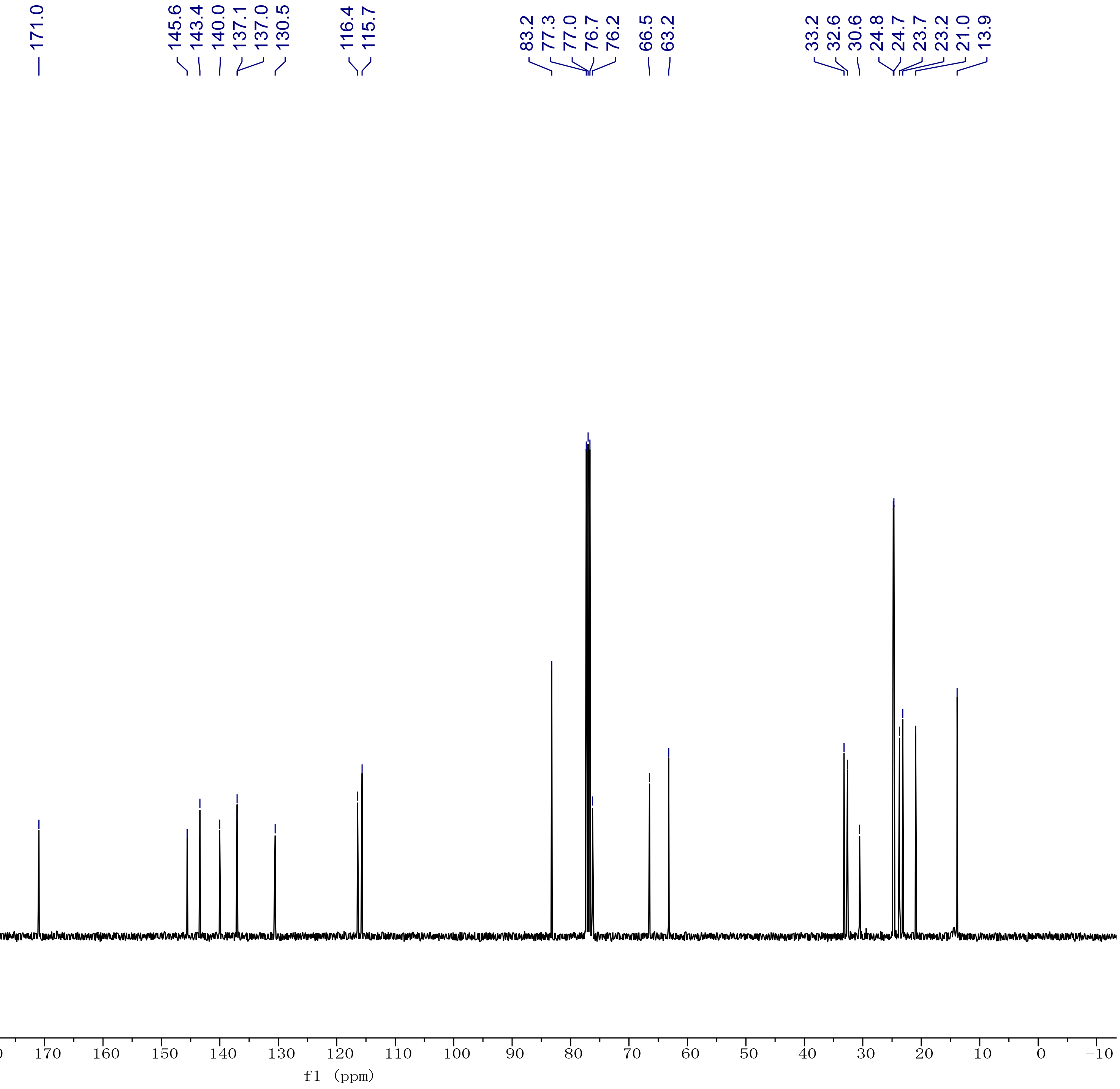

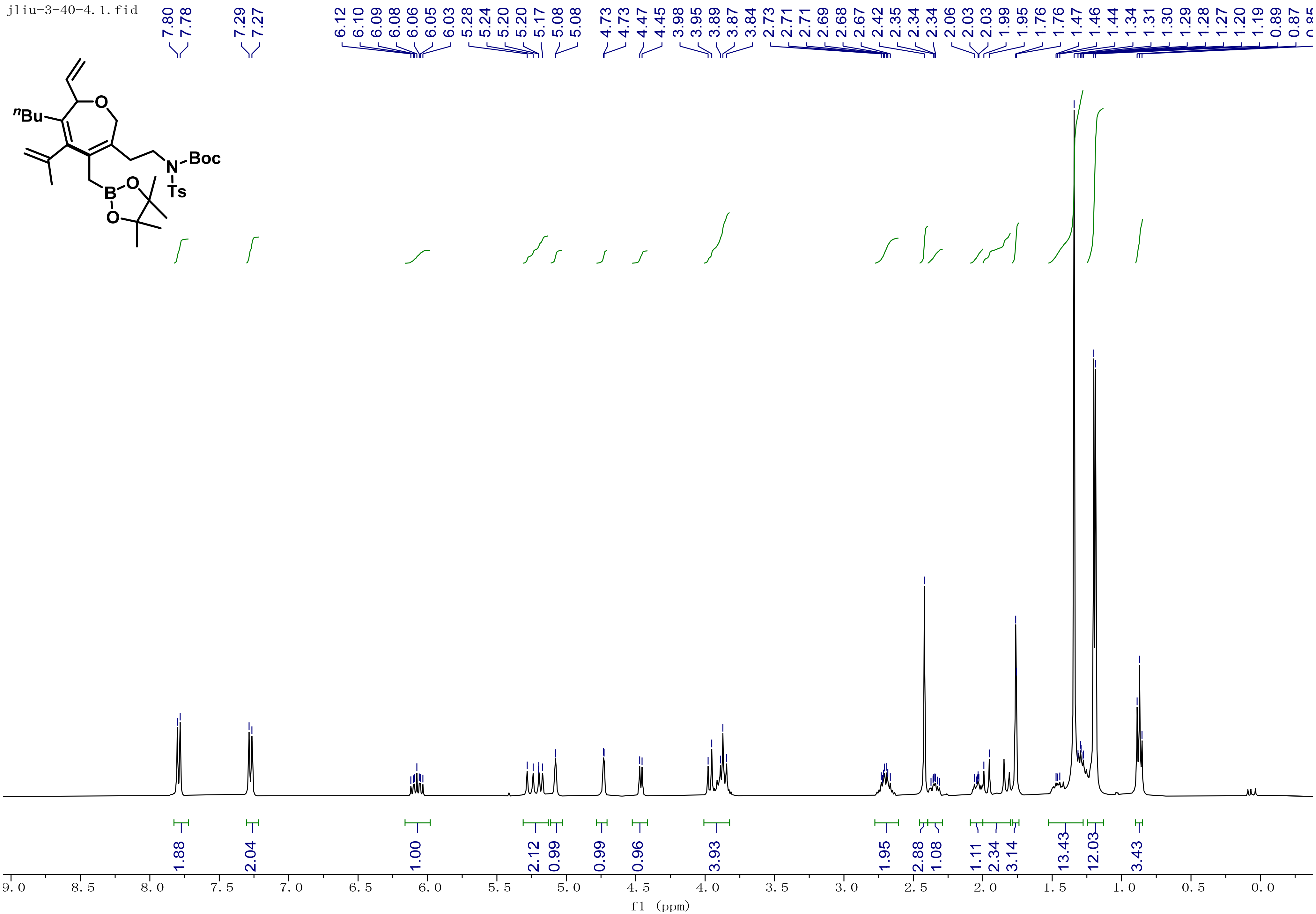
jliu-3-40-4.1.fid
7.80 7.78 7.29 7.27 6.12 6.10 6.09 6.08 6.06 6.05 6.03 5.28 5.24 5.20 5.20 5.17 5.08 5.08 4.73 4.73 4.47 4.45 3.98 3.95 3.89 3.87 3.84 2.73 2.71 2.71 2.69 2.68 2.67 2.42 2.35 2.34 2.34 2.06 2.03 2.03 1.99 1.95 1.76 1.76 1.47 1.46 1.44 1.34 1.31 1.30 1.29 1.28 1.27 1.20 1.19 0.89 0.87
nBu
O
N
Boc
Ts
B
O
O
1.88 2.04 1.00 2.12 0.99 0.99 0.96 3.93 1.95 2.88 1.08 1.11 2.34 3.14 13.43 12.03 3.43
9.0 8.5 8.0 7.5 7.0 6.5 6.0 5.5 5.0 4.5 4.0 3.5 3.0 2.5 2.0 1.5 1.0 0.5 0.0
f1 (ppm)

jliu-3-40-4.3.fid

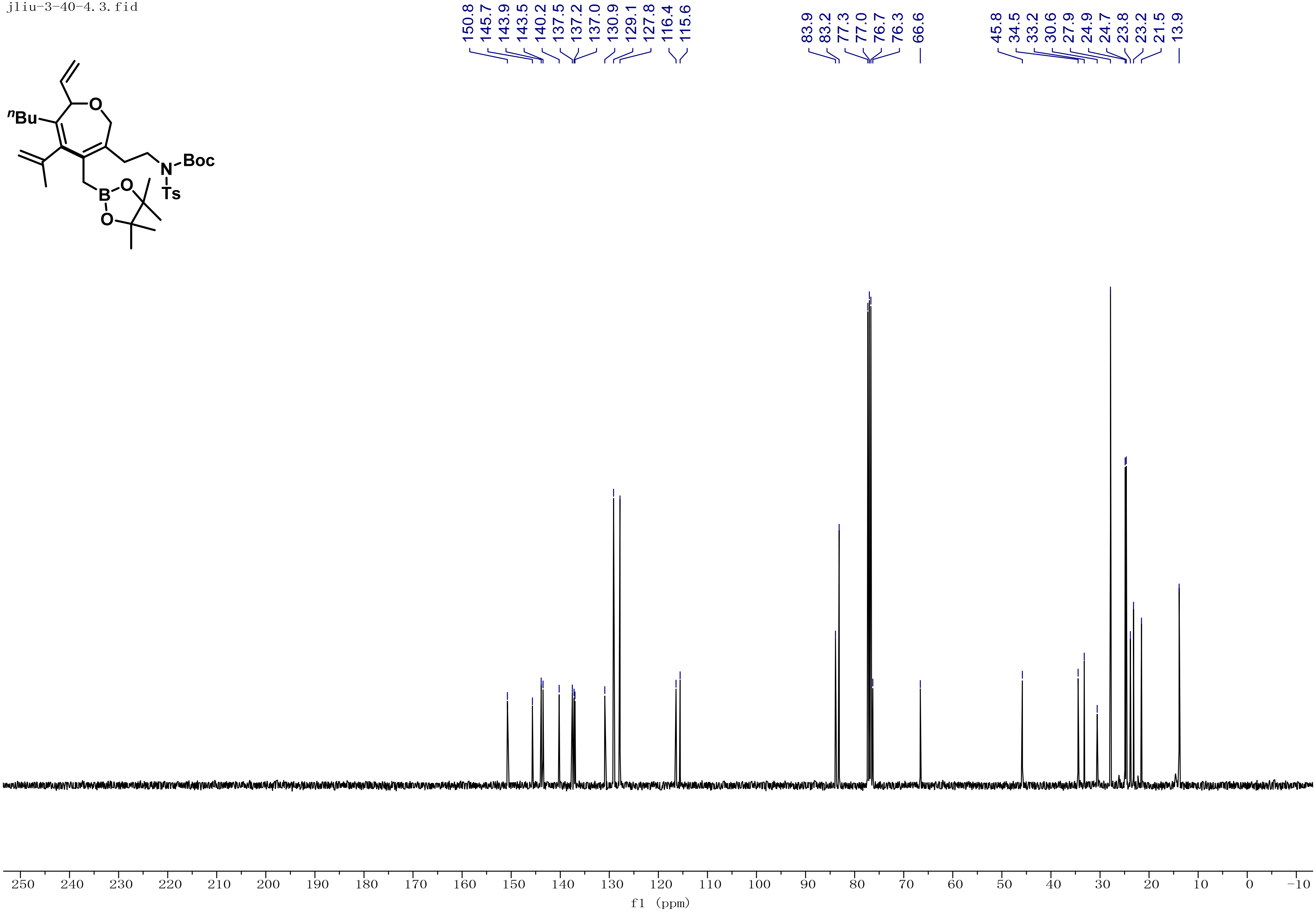

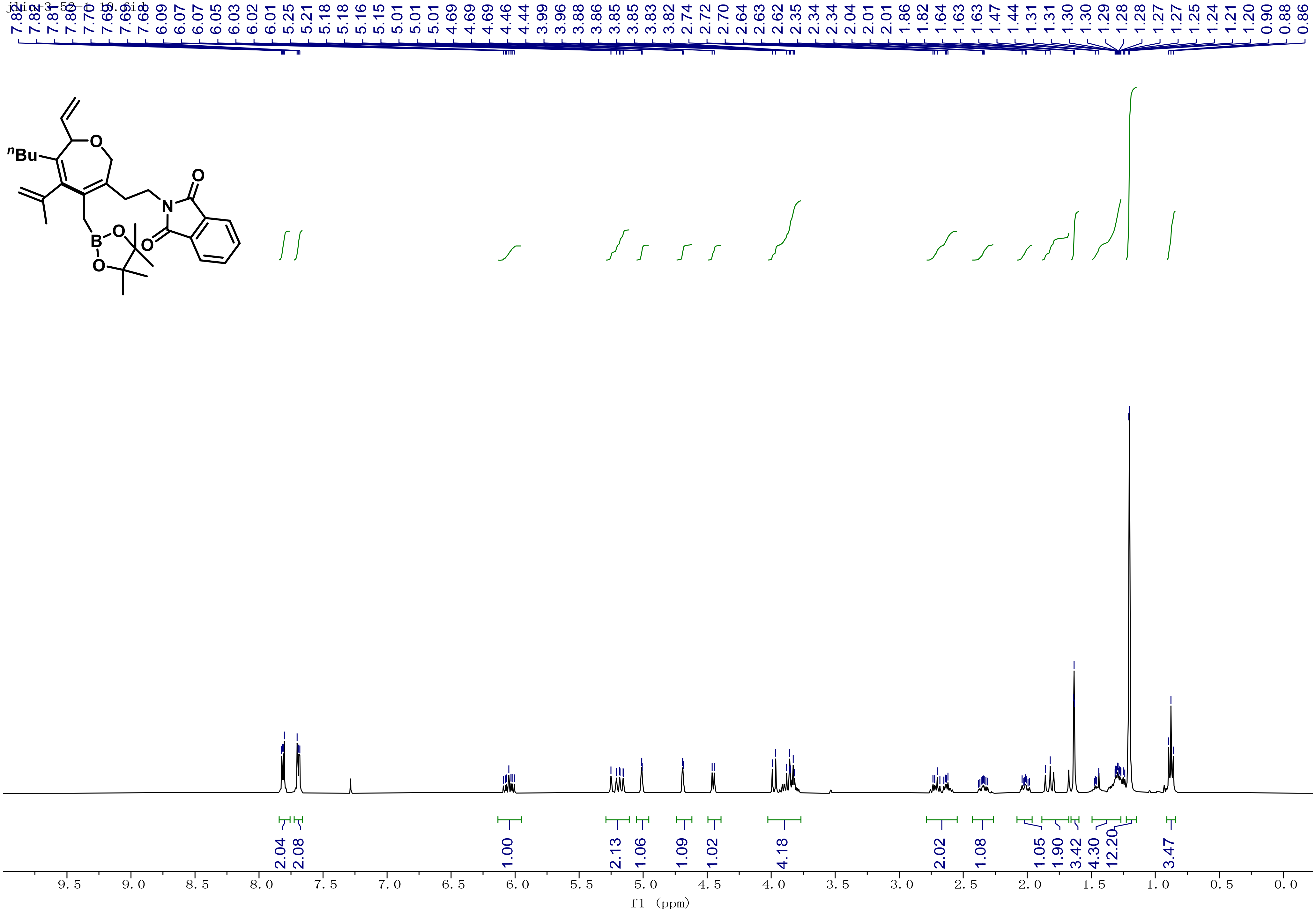
7.82 7.81 7.80 7.70 7.69 7.69 7.68 6.09 6.07 6.07 6.05 6.03 6.02 6.01 5.25 5.21 5.18 5.18 5.16 5.15 5.01 5.01 5.01 4.69 4.69 4.69 4.46 4.44 3.99 3.96 3.88 3.86 3.85 3.85 3.83 3.82 2.74 2.72 2.70 2.64 2.63 2.62 2.35 2.34 2.34 2.04 2.01 2.01 1.86 1.82 1.64 1.63 1.63 1.47 1.44 1.31 1.31 1.30 1.30 1.29 1.28 1.28 1.27 1.27 1.25 1.24 1.21 1.20 0.90 0.88 0.86
nBu
O
N
B
2.04
2.08
1.00
2.13
1.06
1.09
1.02
4.18
2.02
1.08
1.05
1.90
3.42
4.30
12.20
3.47
9.5 9.0 8.5 8.0 7.5 7.0 6.5 6.0 5.5 5.0 4.5 4.0 3.5 3.0 2.5 2.0 1.5 1.0 0.5 0.0
f1 (ppm)

jliu-3-52-1.11.fid

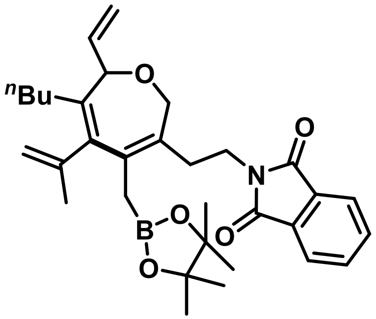

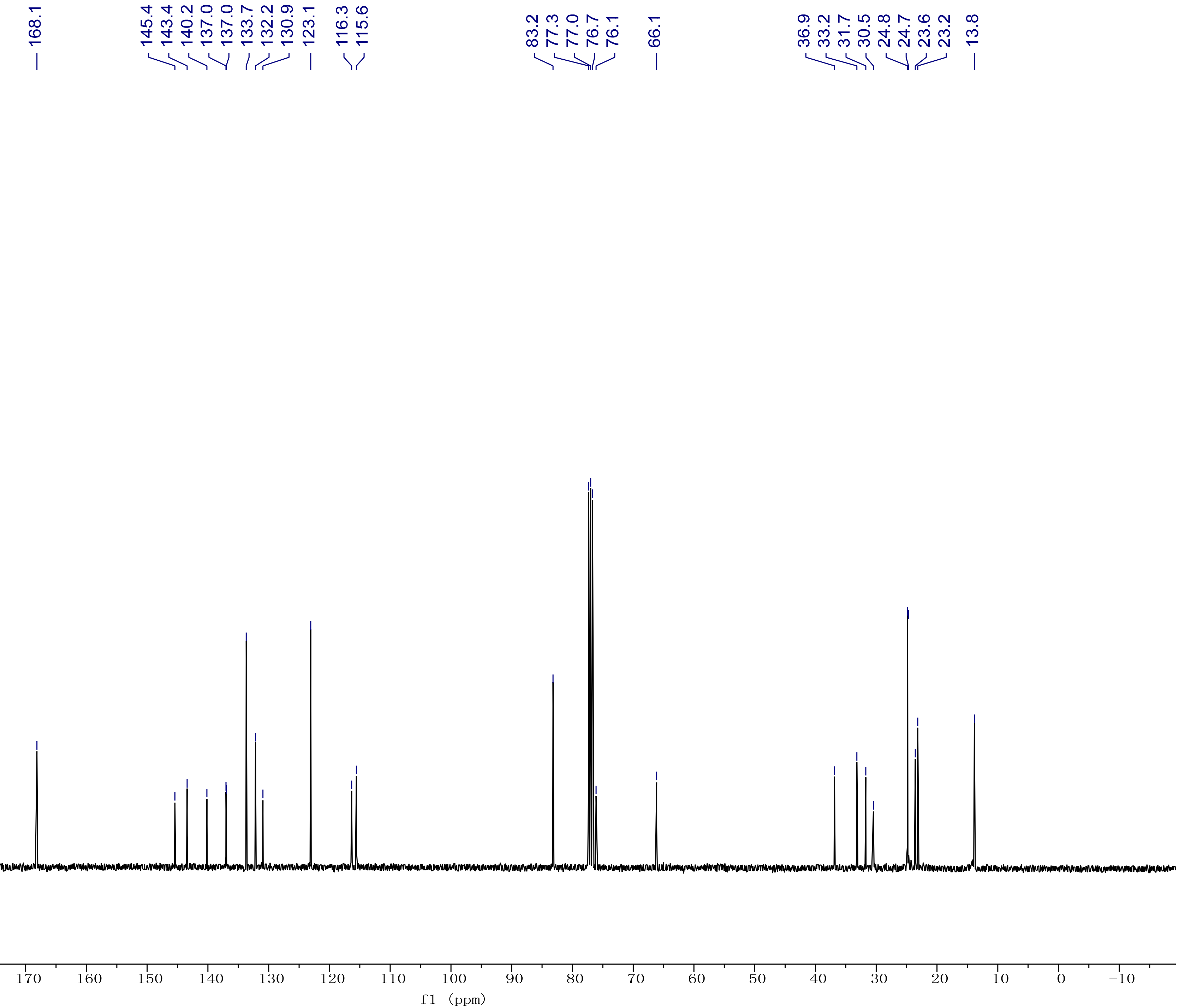

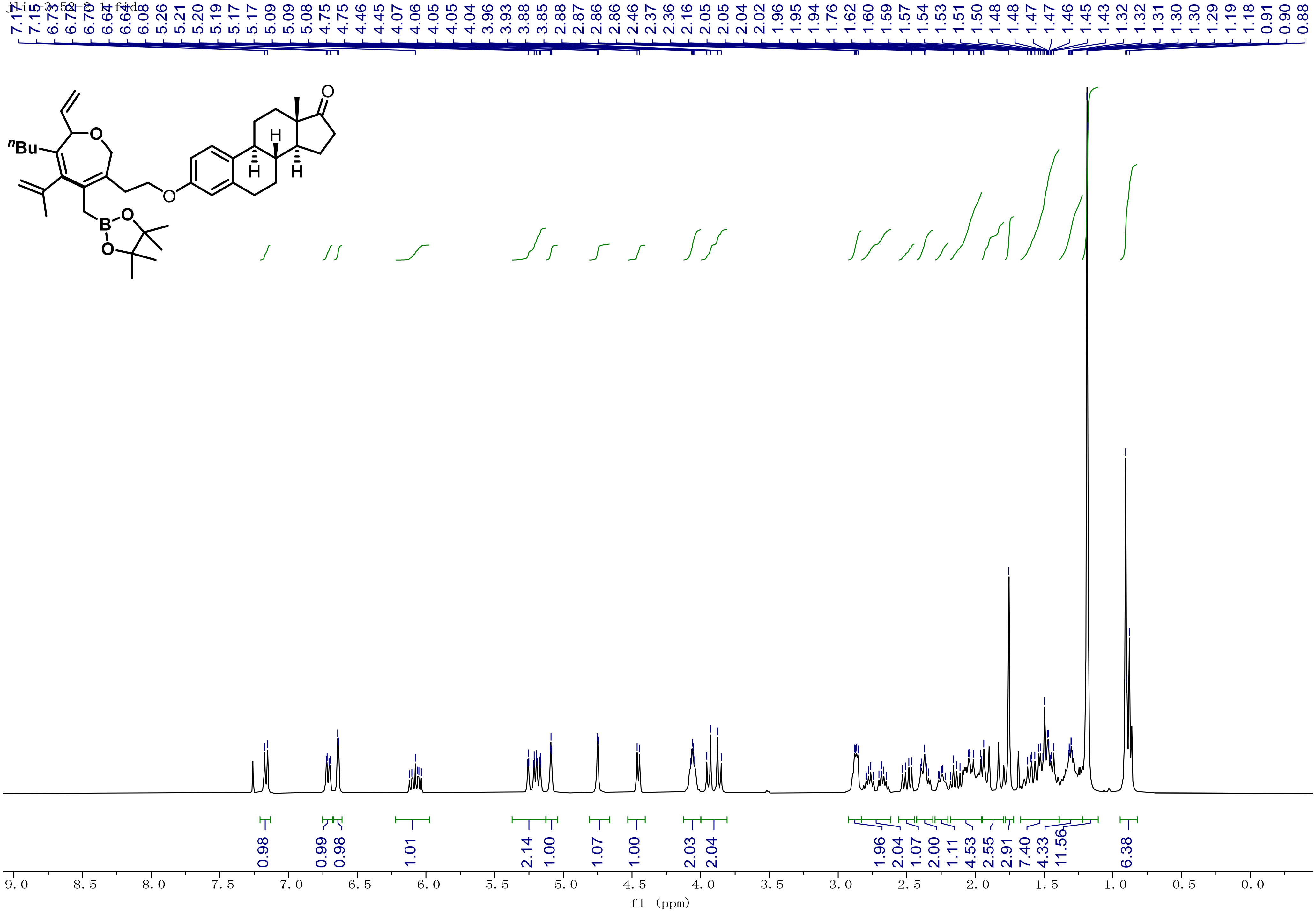

jliu-3-52-2-1.fid
7.15 7.15 6.73 6.72 6.70 6.64 6.64 6.08 5.26 5.21 5.20 5.19 5.17 5.17 5.09 5.09 5.08 4.75 4.75 4.46 4.45 4.07 4.06 4.05 4.05 4.04 3.96 3.93 3.88 3.85 2.88 2.87 2.86 2.86 2.46 2.37 2.36 2.16 2.05 2.05 2.04 2.02 1.96 1.95 1.94 1.76 1.62 1.60 1.59 1.57 1.54 1.53 1.51 1.50 1.48 1.48 1.47 1.47 1.47 1.46 1.45 1.43 1.32 1.32 1.31 1.30 1.30 1.29 1.19 1.18 0.91 0.90 0.88
nBu
O
B
0.98 0.99 0.98 1.01 2.14 1.00 1.07 1.00 2.03 2.04 1.96 2.04 1.07 2.00 1.11 4.53 2.55 2.91 7.40 4.33 11.56 6.38
9.0 8.5 8.0 7.5 7.0 6.5 6.0 5.5 5.0 4.5 4.0 3.5 3.0 2.5 2.0 1.5 1.0 0.5 0.0
f1 (ppm)

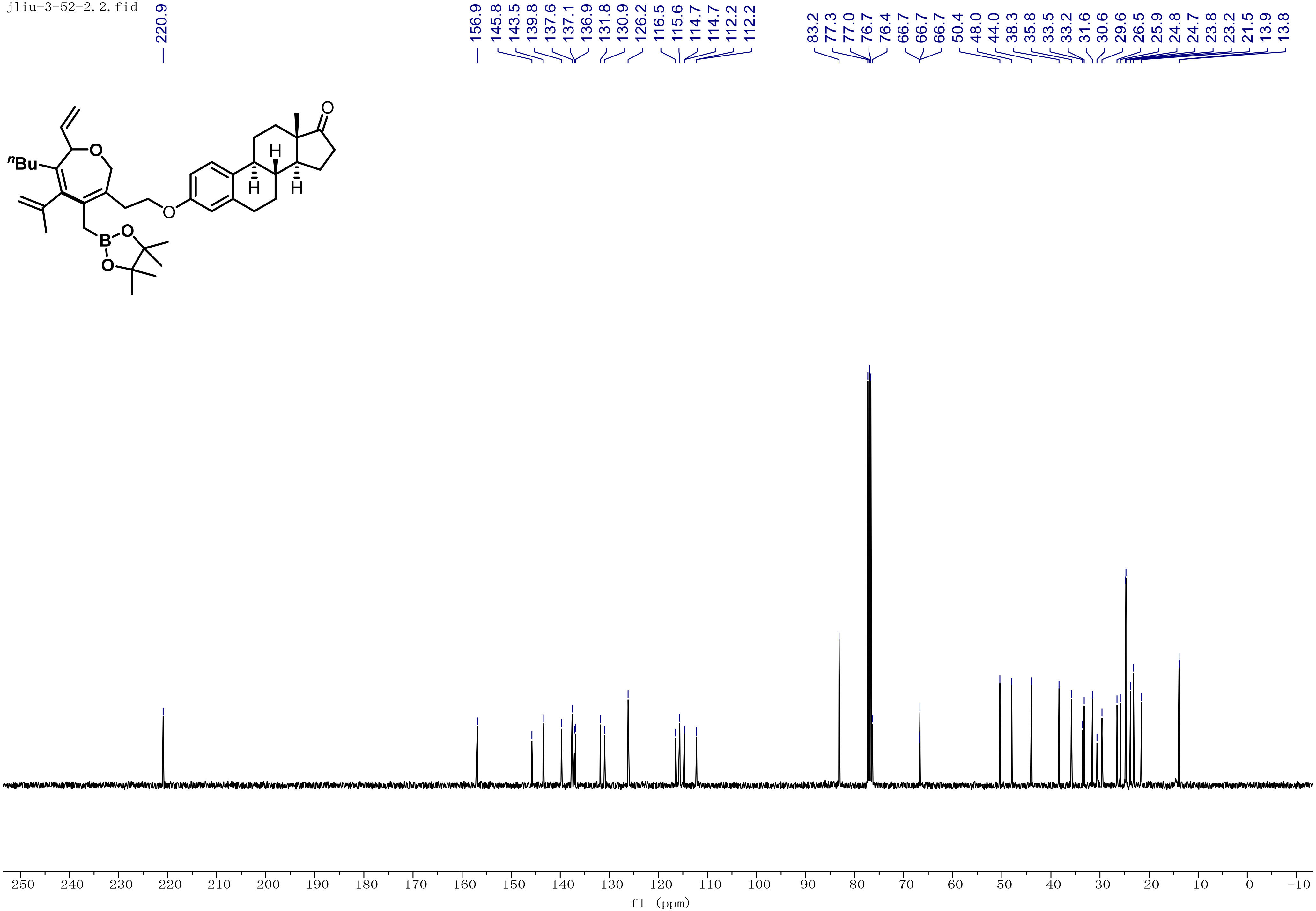
jliu-3-52-2.2.fid
220.9
156.9
145.8
143.5
139.8
137.6
137.1
136.9
131.8
130.9
126.2
116.5
115.6
114.7
114.7
112.2
112.2
83.2
77.3
77.0
76.7
76.4
66.7
66.7
66.7
50.4
48.0
44.0
38.3
35.8
33.5
33.2
31.6
30.6
29.6
26.5
25.9
24.8
24.7
23.8
23.2
21.5
13.9
13.8
nBu
250 240 230 220 210 200 190 180 170 160 150 140 130 120 110 100 90 80 70 60 50 40 30 20 10 0 −10
f1 (ppm)

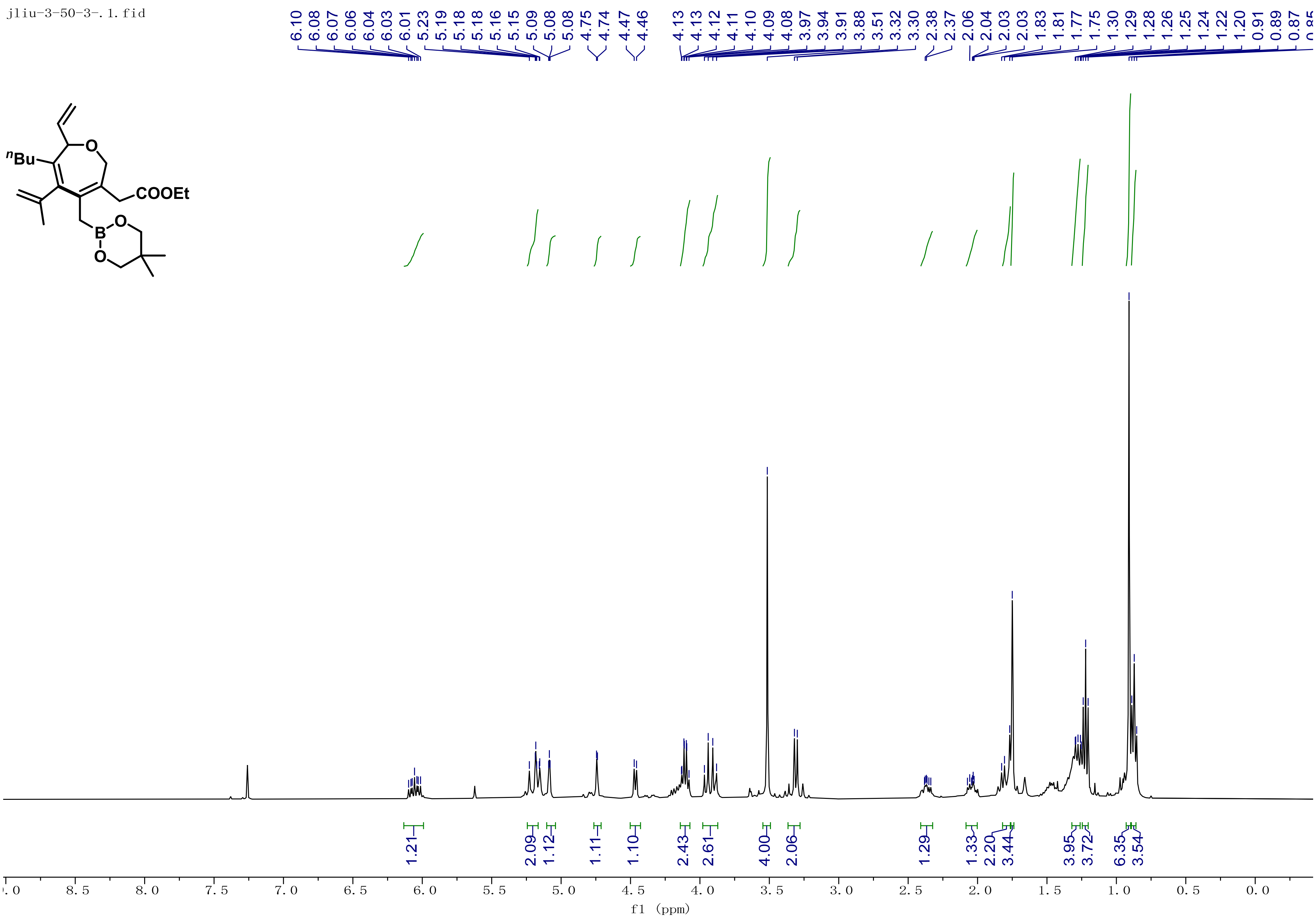
jliu-3-50-3-.1.fid
6.10 6.08 6.07 6.06 6.04 6.03 6.01 5.23 5.19 5.18 5.18 5.16 5.15 5.09 5.08 5.08 4.75 4.74 4.47 4.46 4.13 4.13 4.12 4.11 4.10 4.09 4.08 3.97 3.94 3.91 3.88 3.51 3.32 3.30 2.38 2.37 2.06 2.04 2.03 2.03 1.83 1.81 1.77 1.75 1.30 1.29 1.28 1.26 1.25 1.24 1.22 1.20 0.91 0.89 0.87
nBu
O
COOEt
B
O
O
1.21 2.09 1.12 1.11 1.10 2.43 2.61 4.00 2.06 1.29 1.33 2.20 3.44 3.95 3.72 6.35 3.54
8.5 8.0 7.5 7.0 6.5 6.0 5.5 5.0 4.5 4.0 3.5 3.0 2.5 2.0 1.5 1.0 0.5 0.0
f1 (ppm)

jliu-3-50-3-C.1.fid

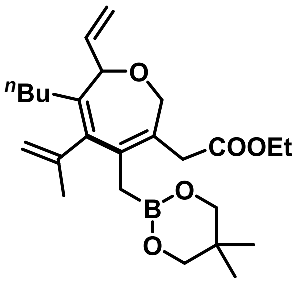

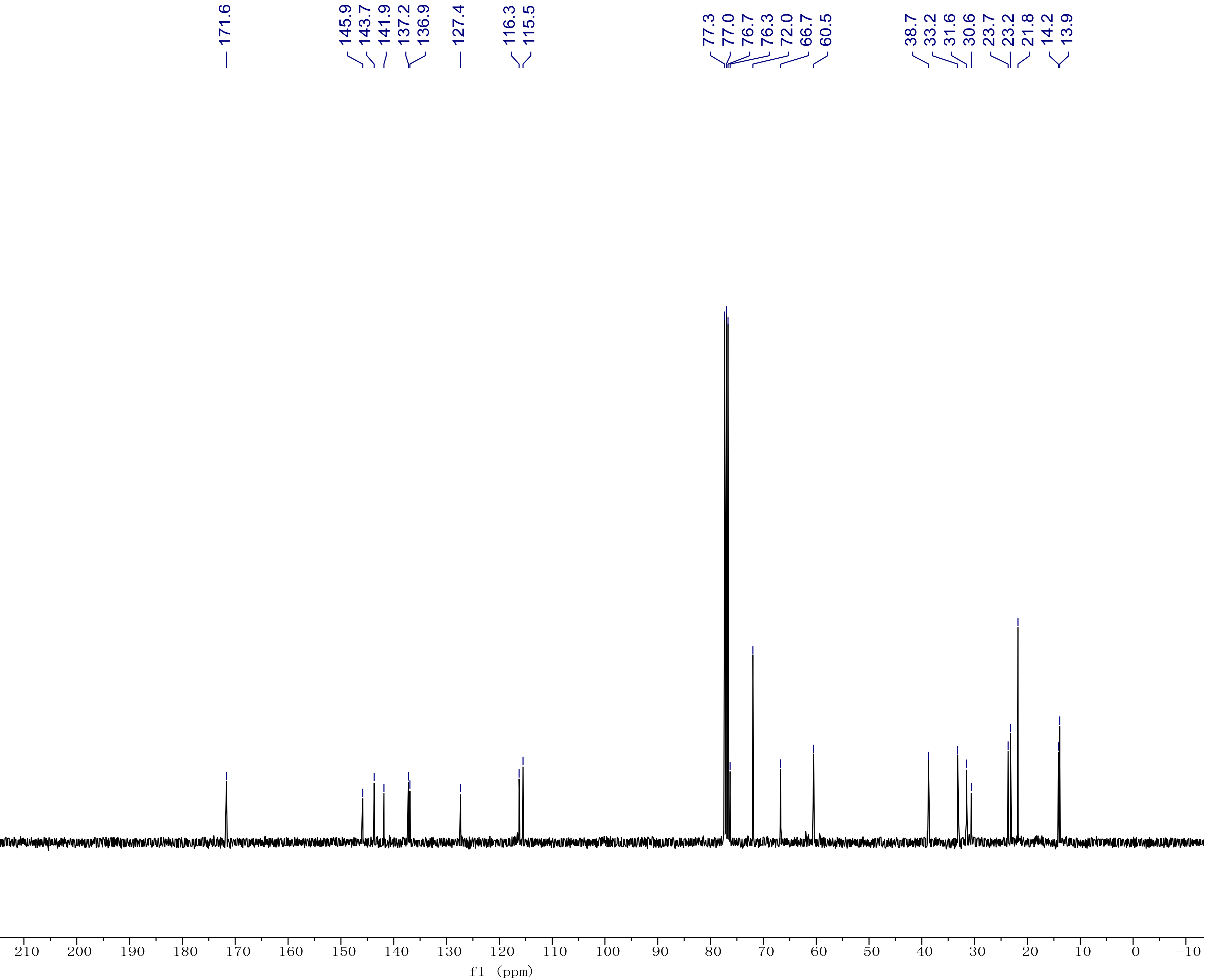

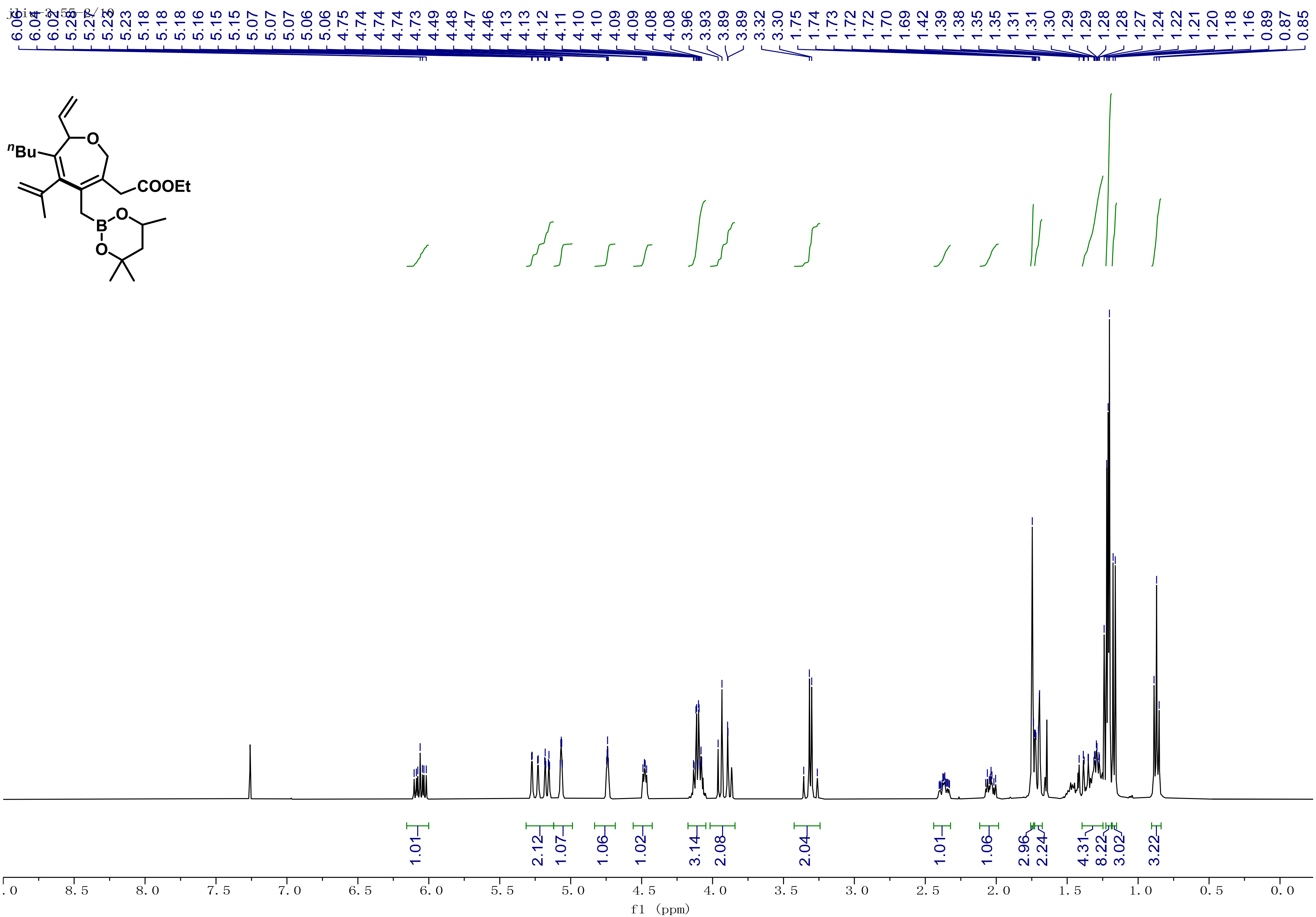
6.04 6.02 5.28 5.27 5.23 5.23 5.18 5.18 5.18 5.16 5.15 5.15 5.07 5.07 5.07 5.07 5.06 5.06 4.75 4.74 4.74 4.74 4.73 4.49 4.48 4.47 4.46 4.13 4.13 4.12 4.11 4.10 4.10 4.09 4.09 4.08 4.08 3.96 3.93 3.89 3.89 3.32 3.30 1.75 1.74 1.73 1.72 1.72 1.70 1.69 1.42 1.39 1.38 1.35 1.35 1.31 1.31 1.30 1.29 1.29 1.28 1.28 1.28 1.27 1.24 1.22 1.21 1.20 1.18 1.16 0.89 0.87 0.85
nBu
COOEt
O
B
1.01
2.12
1.07
1.06
1.02
3.14
2.08
2.04
1.01
1.06
2.96
2.24
4.31
8.22
3.02
3.22
8.5 8.0 7.5 7.0 6.5 6.0 5.5 5.0 4.5 4.0 3.5 3.0 2.5 2.0 1.5 1.0 0.5 0.0
f1 (ppm)

jliu-3-55-2/11

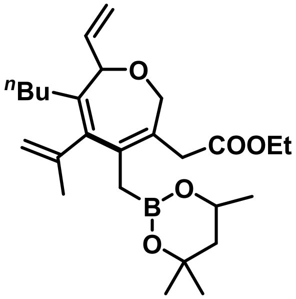

nBu
O
COOEt
B
O
O

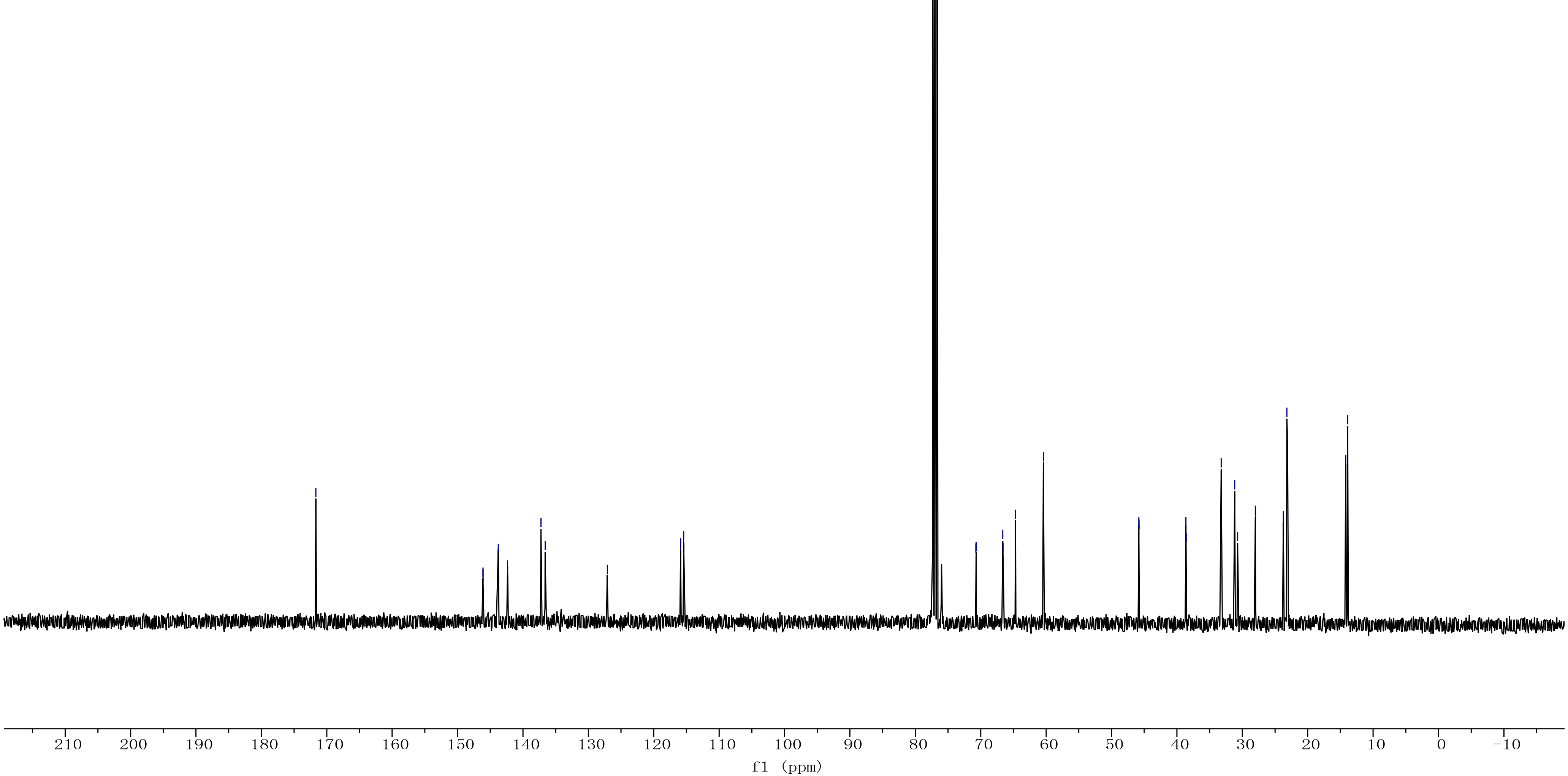

171.7
146.1
146.1
143.8
142.4
142.3
137.2
136.6
127.1
115.9
115.9
115.5
115.4
77.3
77.0
76.7
76.0
70.7
70.7
66.6
66.6
64.7
60.4
45.8
45.8
38.6
38.6
33.2
31.2
30.7
28.0
28.0
23.7
23.7
23.2
23.1
14.2
13.9
210
200
190
180
170
160
150
140
130
120
110
100
90
80
70
60
50
40
30
20
10
0
-10
f1 (ppm)

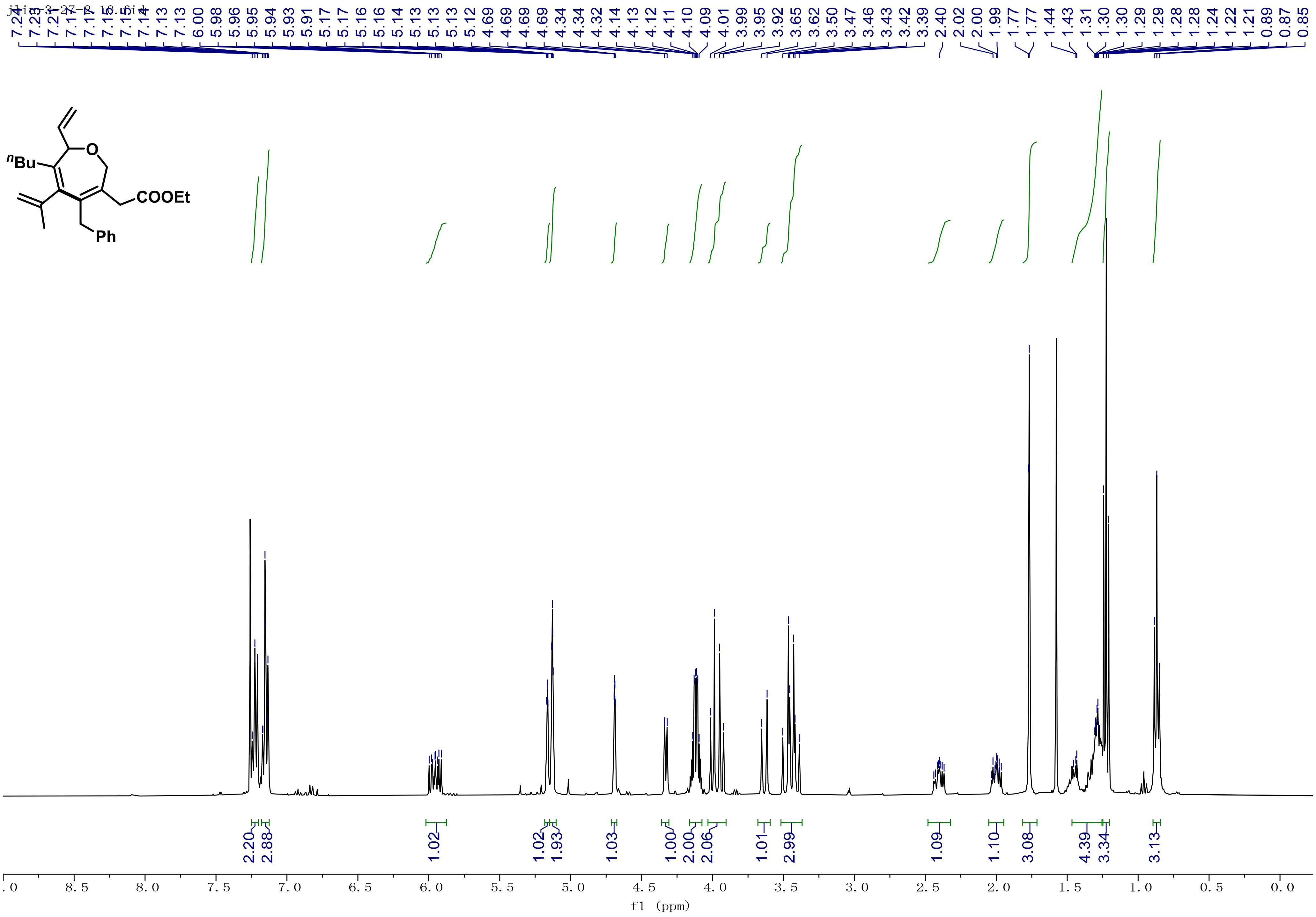
7.24 7.23 7.21 7.17 7.15 7.15 7.14 7.13 7.13 6.00 5.98 5.96 5.95 5.94 5.93 5.91 5.17 5.17 5.16 5.16 5.14 5.13 5.13 5.13 5.12 4.69 4.69 4.69 4.69 4.34 4.34 4.32 4.14 4.13 4.12 4.11 4.10 4.09 4.01 3.99 3.95 3.92 3.65 3.62 3.50 3.47 3.46 3.43 3.42 3.39 2.40 2.02 2.00 1.99 1.77 1.77 1.44 1.43 1.31 1.30 1.30 1.29 1.29 1.28 1.28 1.24 1.22 1.21 0.89 0.87 0.85
^{n}Bu
O
COOEt
Ph
2.20 2.88 1.02 1.02 1.93 1.03 1.00 2.00 2.06 1.01 2.99 1.09 1.10 3.08 4.39 3.34 3.13
8.5 8.0 7.5 7.0 6.5 6.0 5.5 5.0 4.5 4.0 3.5 3.0 2.5 2.0 1.5 1.0 0.5 0.0
f1 (ppm)

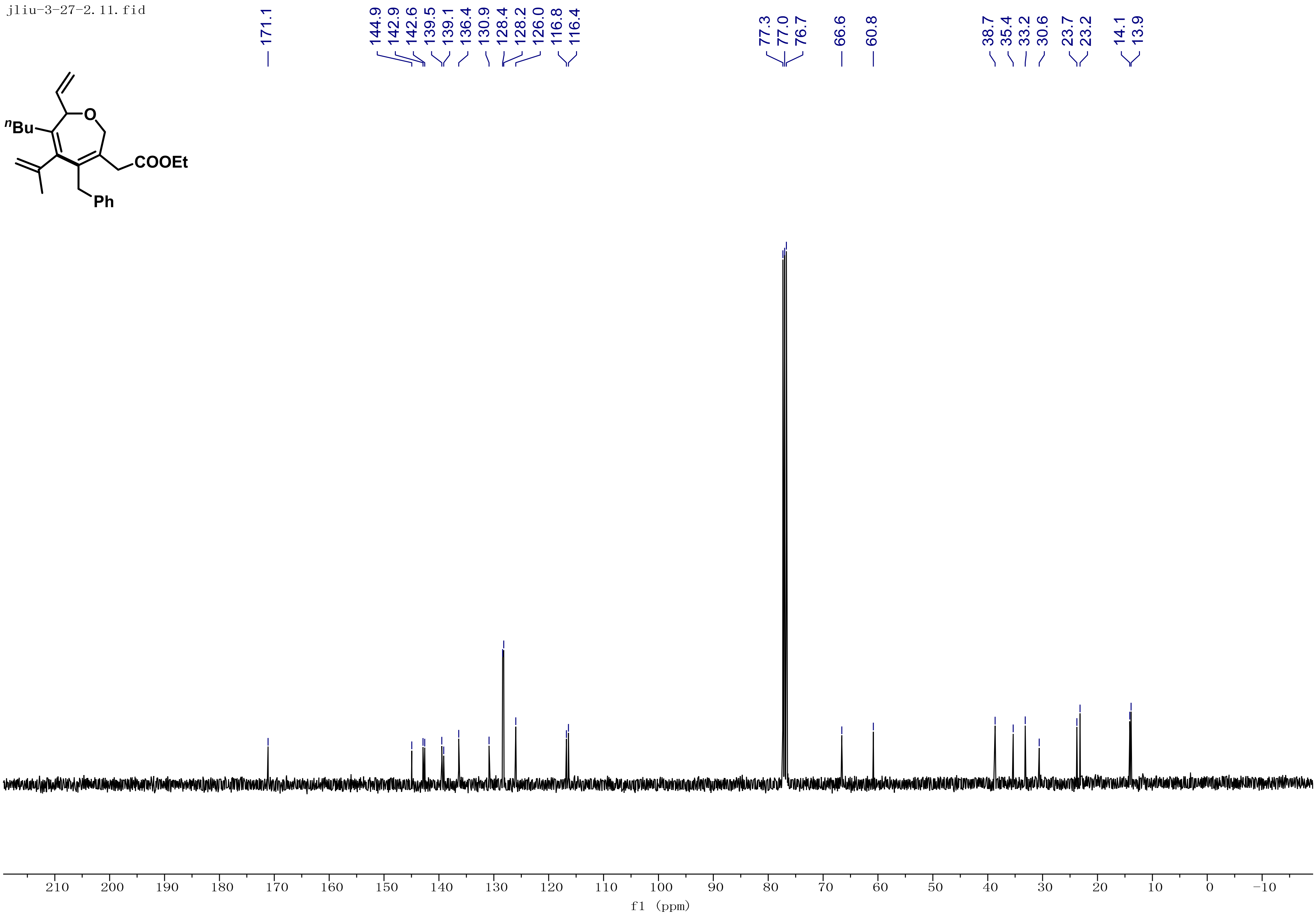
jliu-3-27-2.11.fid
171.1
144.9
142.9
142.6
139.5
139.1
136.4
130.9
128.4
128.2
126.0
116.8
116.4
77.3
77.0
76.7
66.6
60.8
38.7
35.4
33.2
30.6
23.7
23.2
14.1
13.9
nBu
O
COOEt
Ph
210 200 190 180 170 160 150 140 130 120 110 100 90 80 70 60 50 40 30 20 10 0 -10
f1 (ppm)

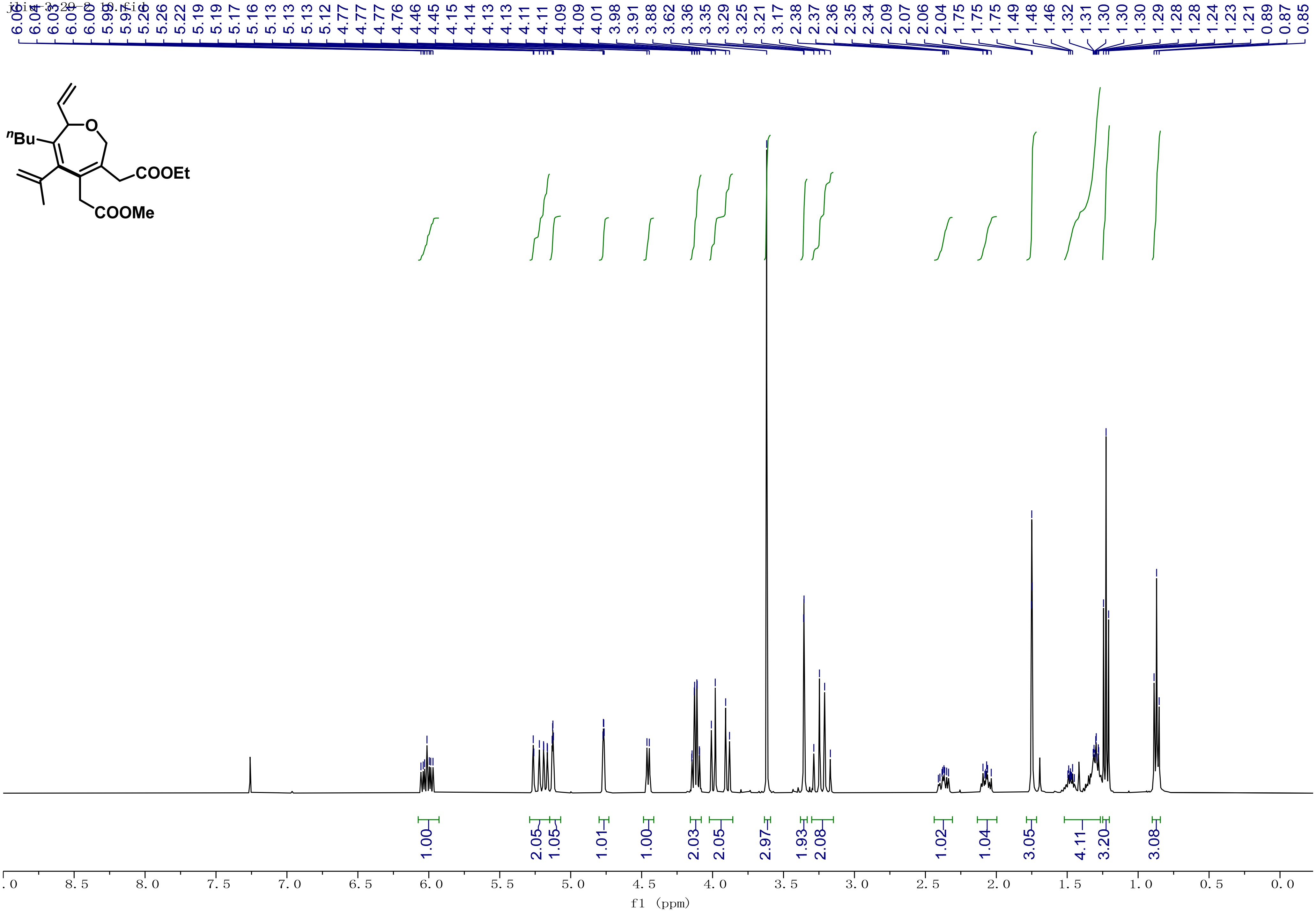

6.04 6.03 6.01 6.00 5.99 5.97 5.26 5.26 5.22 5.19 5.19 5.17 5.16 5.13 5.13 5.13 5.12 4.77 4.77 4.77 4.76 4.46 4.45 4.15 4.14 4.13 4.13 4.11 4.11 4.09 4.09 4.01 3.98 3.91 3.88 3.62 3.36 3.35 3.29 3.25 3.21 3.17 2.38 2.37 2.36 2.35 2.34 2.09 2.07 2.06 2.04 1.75 1.75 1.75 1.49 1.48 1.46 1.32 1.31 1.30 1.30 1.30 1.29 1.28 1.28 1.24 1.23 1.21 0.89 0.87 0.85
^{n}Bu
O
COOEt
COOMe
1.00 2.05 1.05 1.01 1.00 2.03 2.05 2.97 1.93 2.08 1.02 1.04 3.05 4.11 3.20 3.08
8.5 8.0 7.5 7.0 6.5 6.0 5.5 5.0 4.5 4.0 3.5 3.0 2.5 2.0 1.5 1.0 0.5 0.0
f1 (ppm)

jliu-3-29-2.11.fid

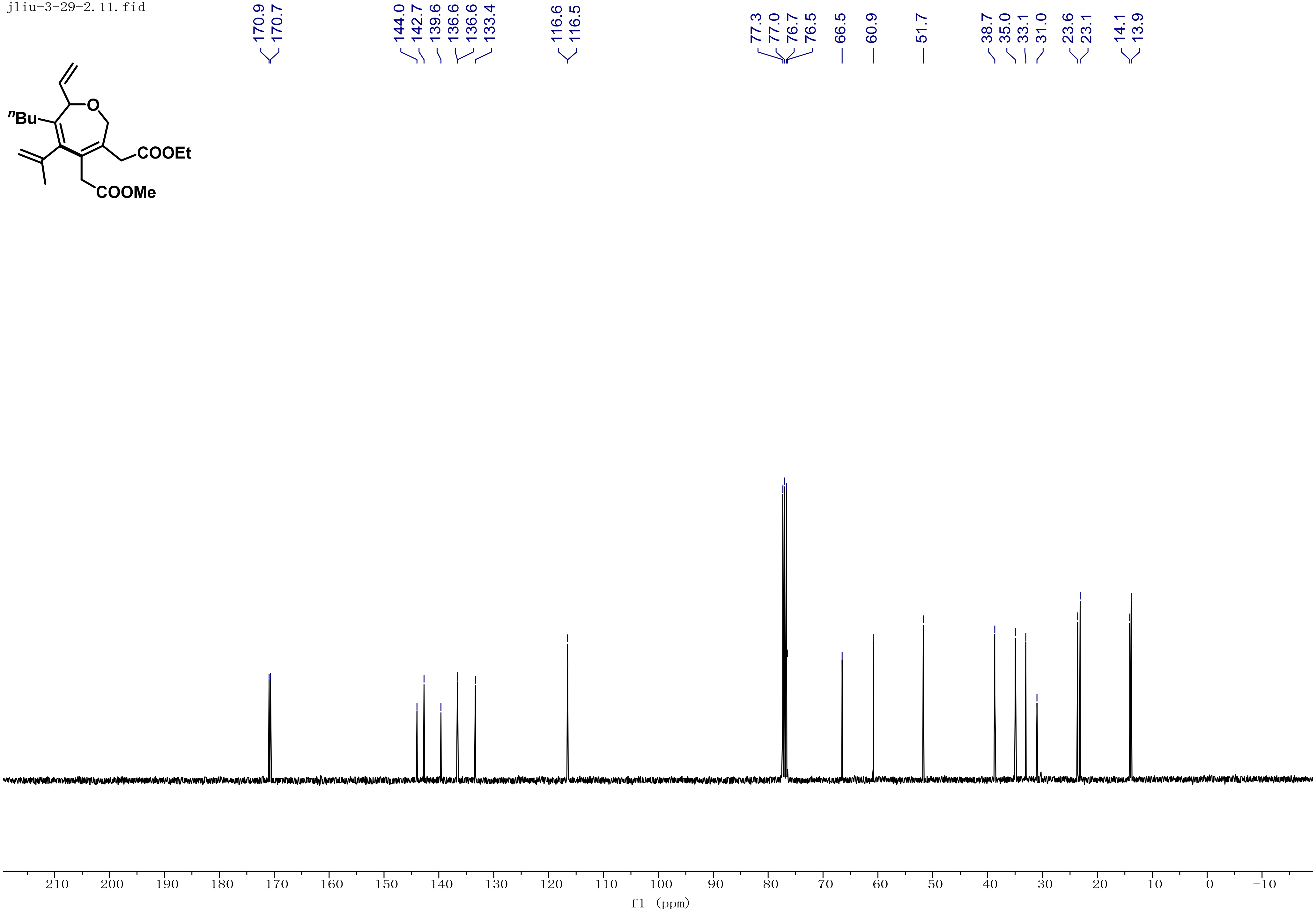

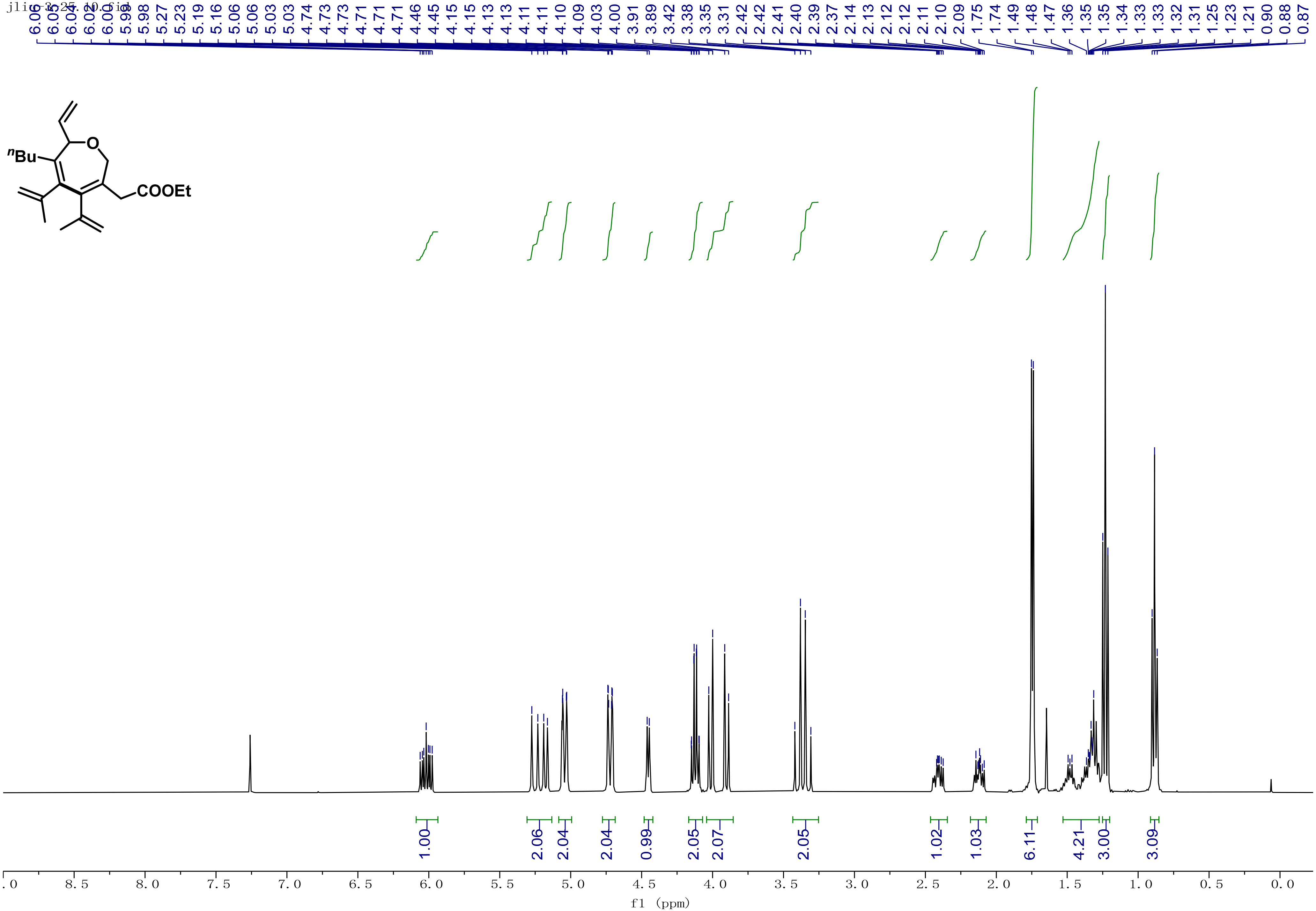

jlic-3-25.10.fid
6.06 6.05 6.04 6.02 6.00 5.99 5.98 5.27 5.23 5.19 5.16 5.06 5.06 5.03 5.03 4.74 4.73 4.73 4.71 4.71 4.71 4.46 4.45 4.15 4.15 4.13 4.13 4.11 4.11 4.10 4.09 4.03 4.00 3.91 3.89 3.42 3.38 3.35 3.31 2.42 2.42 2.41 2.40 2.39 2.37 2.14 2.13 2.12 2.12 2.11 2.10 2.09 1.75 1.74 1.49 1.48 1.47 1.36 1.35 1.35 1.34 1.33 1.33 1.32 1.31 1.25 1.23 1.21 0.90 0.88 0.87
^{n}Bu
O
COOEt
1.00
2.06
2.04
2.04
0.99
2.05
2.07
2.05
1.02
1.03
6.11
4.21
3.00
3.09
9.0 8.5 8.0 7.5 7.0 6.5 6.0 5.5 5.0 4.5 4.0 3.5 3.0 2.5 2.0 1.5 1.0 0.5 0.0
f1 (ppm)

jliu-3-25.11.fid

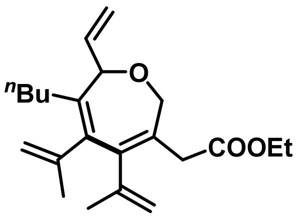

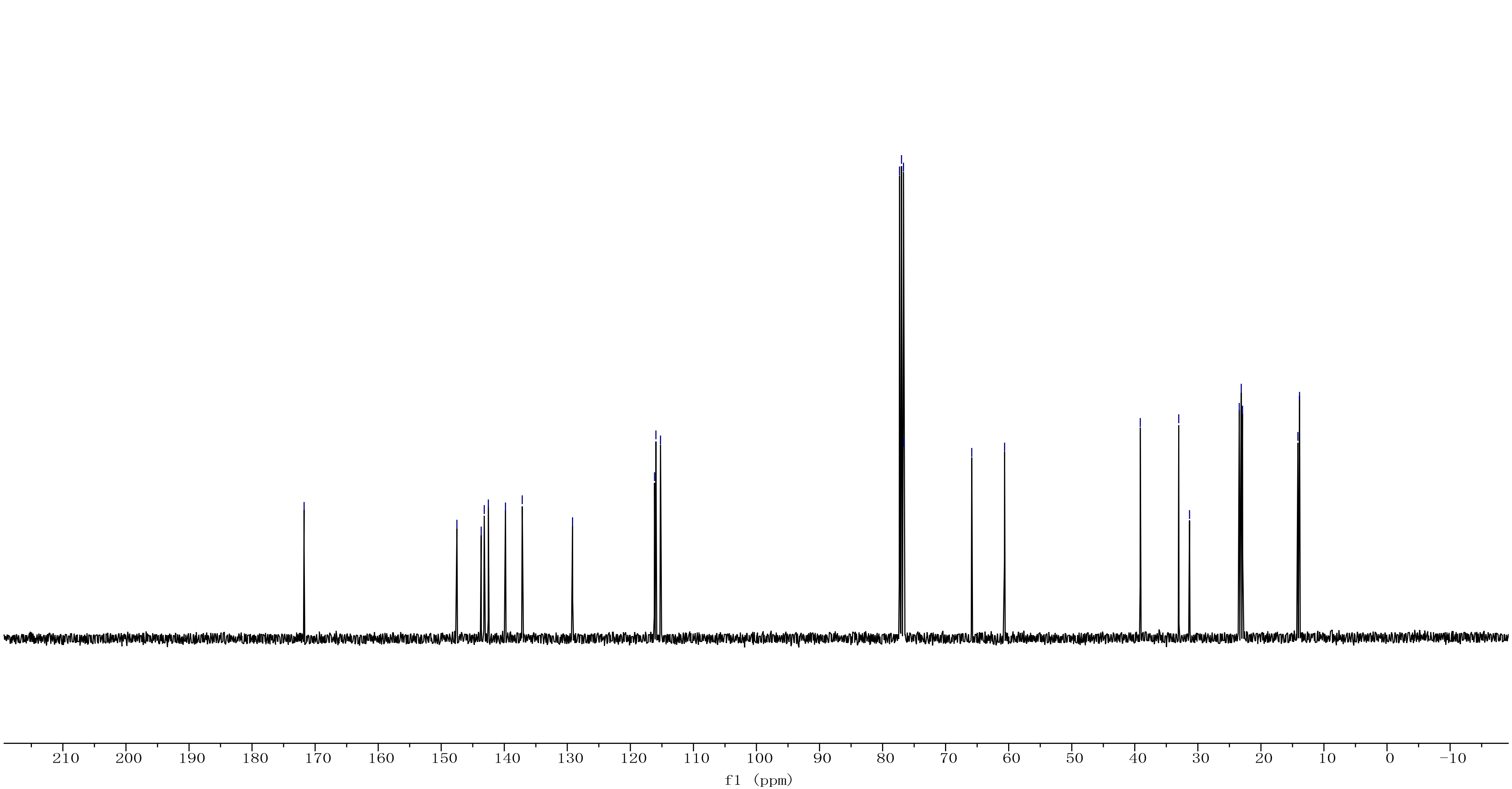

Supplement: Supplementary file 1 — Supplementary [file CHEM-26-15513-s001.pdf]
